# Positive Charge in an Antimalarial Compound Unlocks Broad-Spectrum Antibacterial Activity

**DOI:** 10.1021/jacsau.4c00935

**Published:** 2025-02-21

**Authors:** Maria Braun-Cornejo, Mitchell Platteschorre, Vincent de Vries, Patricia Bravo, Vidhisha Sonawane, Mostafa M. Hamed, Jörg Haupenthal, Norbert Reiling, Matthias Rottmann, Dennis Piet, Peter Maas, Eleonora Diamanti, Anna K. H. Hirsch

**Affiliations:** † Specs Compound Handling, B.V., Bleiswijkseweg 55, Zoetermeer 2712 PB, The Netherlands; ‡ Department of Pharmacy, Saarland University, Campus Building E8.1, Saarbrücken 66123, Germany; § Helmholtz Institute for Pharmaceutical Research Saarland (HIPS)—Helmholtz Centre for Infection Research (HZI), Campus Building E8.1, Saarbrücken 66123, Germany; ∥ Swiss Tropical and Public Health Institute, Kreuzstrasse 2, Allschwil 4123, Switzerland; ⊥ Universität Basel, Petersplatz 1, Basel 4003, Switzerland; # Microbial Interface Biology, Research Center Borstel, Leibniz Lung Center, Borstel 23845, Germany; ¶ German Center for Infection Research (DZIF), Partner Site Hamburg-Lübeck-Borstel-Riems, Borstel 23845, Germany

**Keywords:** antimicrobial resistance, eNTRy rules, antimalarial, broad-spectrum antibiotic, antitubercular, Gram-negative accumulation

## Abstract

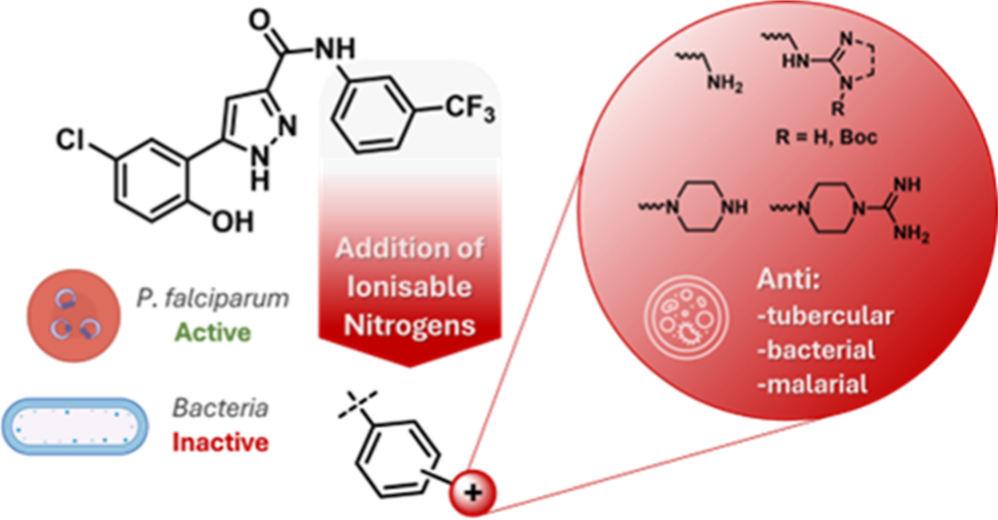

In this study, we synthesized a library of eNTRy-rule-compliant compounds by introducing ionizable nitrogen atoms to an antimalarial compound. These positively charged derivatives gained activity against both Gram-negative and -positive bacteria, *Mycobacterium tuberculosis*, and boosted *Plasmodium falciparum* inhibition to the double-digit nanomolar range. Overcoming and remaining inside the cell envelope of Gram-negative bacteria (GNB) is one of the major difficulties in antibacterial drug discovery and development. The eNTRy rules (N = ionizable nitrogen, T = low three-dimensionality, R = rigidity) can be a useful structural guideline to improve accumulation of small molecules in GNB. With the aim of unlocking Gram-negative activity, we added amines and (cyclic) *N*-alkyl guanidines to an already flat and rigid pyrazole-amide class as a representative example for our investigation. To test their performance, we compared these eNTRy-rule-compliant compounds to closely related noncompliant ones through phenotypic screening of various pathogens (*P. falciparum*, *Escherichia coli*, *Acinetobacter baumannii*, *Pseudomonas aeruginosa*, *Staphylococcus aureus*, *Streptococcus pneumoniae*, and *M. tuberculosis*), obtaining a handful of broad-spectrum hits. The results support the working hypothesis and even extend its applicability. The studied pyrazole-amide class adheres to the eNTRy rules; noncompliant compounds do not kill any of the bacteria tested, while compliant compounds largely showed growth inhibition of Gram-negative, -positive, and *M. tuberculosis* bacteria in the single-digit micromolar range.

## Introduction

Antimicrobial resistance is increasing rapidly and has become a major global health threat.^1^ The World Health Organization (WHO) highlights the urgency for novel treatments against Gram-negative bacteria (GNB).^2^ Over the past five decades, few new antibiotic classes have been approved, with Gram-negative active ones being vastly underrepresented.^3^ Therefore, research and development of antibacterial drug candidates should focus more on targeting GNB, ideally designing novel chemical classes with unprecedented modes of action.^4^

The difficulty of small compounds to permeate and remain inside GNB’s cell is the main reason why many antibiotics are active only against Gram-positive bacteria (GPB).^5−7^ Many statistical studies to understand the physicochemical properties that promote compound uptake in GNB have been completed since 1968.^8^ However, correlation of molecular properties and their bacterial activity give skewed results for two main reasons: (1) limited number of antibiotic compound classes causes lack of structural diversity and (2) in general, it is not possible to separate the properties of a molecule that affect its antibacterial activity from the ones that affect its bacterial bioavailability.^9^ A fundamentally different approach was taken in 2017 by developing a biological assay that quantifies the compound concentration inside *Escherichia coli* cells, effectively measuring compound bioavailability.^10^ Applying this assay to a diverse set of nearly 200 compounds and using computational methods to analyze the results, the Hergenrother group developed the so-called “eNTRy rules” (N = ionizable nitrogen, T = low three-dimensionality, R = rigidity).^10,11^ According to these guidelines, compounds containing an ionizable nitrogen atom, with low globularity and high rigidity, are more likely to accumulate inside *E. coli* cells. The group’s initial work identified primary amines as the most effective ionizable nitrogen-containing functional group, outperforming secondary and tertiary amines. Since these rules were introduced, many successes of their application to Gram-positive-only starting points to achieve GNB inhibition have been published.^12−15^ The most advanced compounds show in vivo efficacy and inhibition of critical GNB pathogens like *Klebsiella pneumoniae* and *Acinetobacter baumannii*, indicating that eNTRy rules have a promising broad applicability.^16−18^ In 2021, Hergenrother’s team broadened their investigation to other functional groups and revealed that *N*-alkyl guanidiniums perform similarly to primary amines, regarding enhanced accumulation in *E. coli*.^19^ This finding aligns with the previous work of Masci et al., who observed that the inclusion of an amine or guanidine, into their new antibiotic class, was essential to overcome the GNB outer membrane, obtaining enhanced activity against *E. coli*, *K. pneumoniae*, and *A. baumannii*.^20^ Given that GNB’s membrane composition differs between species and individual strains, with *E. coli*’s membrane generally being easier to cross, applying the eNTRy rules to other Gram-negative species needs caution.^21−23^ For instance, Andrews et al. enhanced the polarity of a hit compound to overcome efflux problems in *E. coli* by introducing various ionizable groups, achieving a significant improvement with primary amine derivatives.^24^ However, this approach did not translate to *A. baumannii* or *Pseudomonas aeruginosa*. Recently, an extensive investigation across different strains of *E. coli*, *A. baumannii*, and *P. aeruginosa* using a carefully designed library of 80 oxazolidinones revealed that small structural changes can heavily influence the accumulation and efflux of this class in different GNB.^25^ This study suggests that *E. coli* and *A. baumannii* have a more comparable membrane composition than *P. aeruginosa*, which generally proved to be more difficult to target.

These important findings on structural features and properties of small molecules and their relationship with GNB uptake mark a crucial starting point for the rational design of anti-Gram-negative antibiotics. The relevance of these rules for compounds that do not show previous antibiotic activity needs to be assessed, as it would be especially useful and important for accessing novel antibacterial classes and thereby delay the emergence of cross-resistance.^3^ Recently, we filtered a screening library for an in silico hit-identification study according to the eNTRy guidelines with the aim of increasing *E. coli* bioavailability.^26^ This approach led to the identification of several *E. coli* inhibitors, indicating that eNTRy rules are beneficial for the selection of antibacterial compound libraries. Optimization of the hits, however, demonstrated the challenges of balancing antibacterial activity with target engagement while minimizing toxicity. In a previous study, we introduced primary amine moieties through amino-acid-based residues to an antimalarial chemical class, obtaining compounds compliant with the eNTRy rules.^27^ These derivatives, however, did not show significant efficacy against *E. coli*, showcasing that the addition of an ionizable nitrogen atom is not always enough to gain GNB uptake.

**Figure 1:**
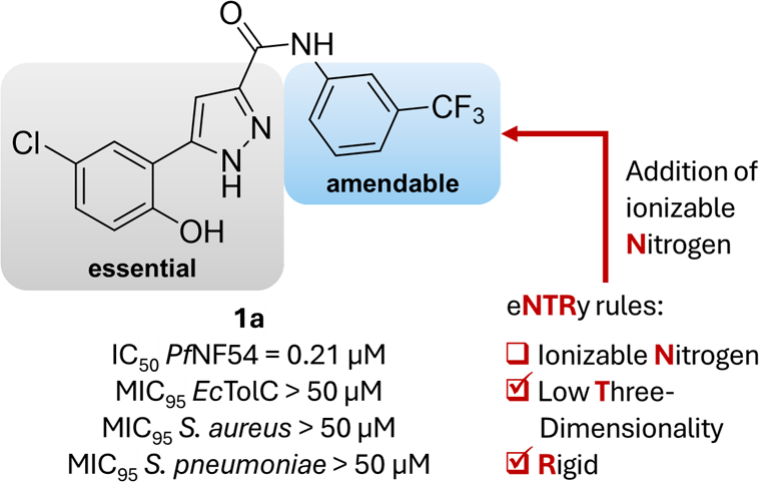
Illustration of our design strategy: use of compound **1a** as an antimalarial starting point to incorporate ionizable nitrogen functionalities. Biological Activity of **1a** in *Plasmodium falciparum* (*Pf*NF54), *Escherichia coli* (*Ec*Δ*tolC* ), *Staphylococcus aureus* (*Sa*), and *Streptococcus pneumoniae* (*Sp*).

In this study, we further investigate the applicability of Hergenrother’s guidelines to antimalarial compounds to expand their anti-infective scope. We achieved this by introducing a variety of ionizable nitrogen functionalities to a flat and rigid antimalarial structure (Figure 1). The functional groups comprise various amine motifs and *N*-alkyl guanidines including novel cyclized forms not previously explored in this context. A concise synthesis yielded 48 derivatives, including neutral controls. The compounds with ionizable nitrogen atoms display broad-spectrum activity against a wide variety of pathogens. In addition to boosting activity against the parasite *Plasmodium falciparum*, many compounds demonstrate antibacterial activity against *E. coli*, *A. baumannii*, *P. aeruginosa*, *Staphylococcus aureus*, *Streptococcus pneumoniae*, and *Mycobacterium tuberculosis*, indicating successful membrane permeation of these pathogens.

## Results and Discussion

### Molecular Design

**Figure 2:**
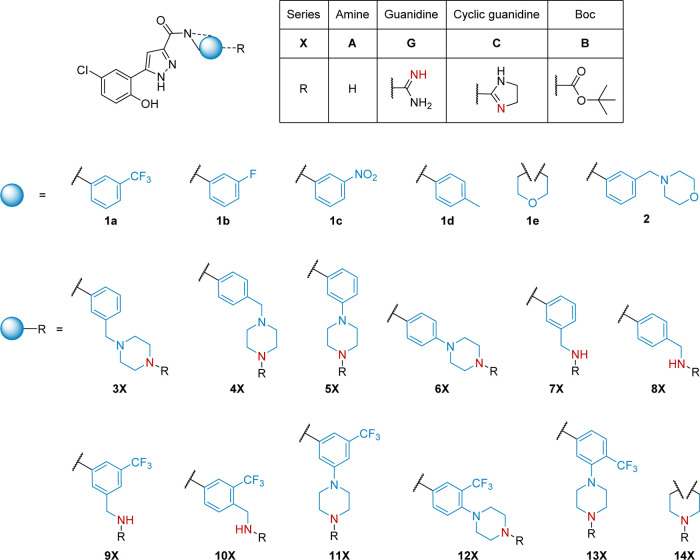
Focused library of a pyrazole-amide class, including uncharged compounds (**1a**–**e, 2,** and **3**–**14B**), amine (**3**–**14A**: p*K*_a_ = 8.2–9.4), guanidine (**5**–**14G**: p*K*_a_ = 11.1–12.1), and cyclic-guanidine (**7**–**11C**: p*K*_a_ = 9.3–11.3) derivatives. Potential ionizable nitrogen atoms are in red, and p*K*_a_ values were computationally determined using ACD/Percepta.

Our antimalarial starting point **1a** originates from previous unpublished work, and its structure comprises three aromatic ring systems: a phenol directly connected to a pyrazole with an amide linking to a trifluoromethyl-substituted phenyl ring (Figure 1). The analysis of our hit molecule with the eNTRy rules revealed that it already complies with two out of the three structural properties from Hergenrother’s findings. Specifically, it is rigid (less than five rotatable bonds), and the scaffold of three connected aromatic rings is extremely flat (low globularity), but it does not contain ionizable nitrogen atoms.^10,11^ Compound **1a** displays antimalarial activity by inhibiting *P. falciparum* in the submicromolar range but shows no antibacterial activity. Our previous unpublished work suggests that the phenol and pyrazole moieties are crucial for antimalarial activity, whereas the amide-linked phenyl is amenable to changes. Modifications on this part of the molecule are easily accessible synthetically, via amide couplings. Therefore, we rationally designed a focused library (Figure 2) of 32 compounds containing ionizable nitrogen atoms while preserving the essential phenol and pyrazole moieties, with the aim of obtaining anti-Gram-negative activity. The introduced positively charged nitrogen-containing functional groups are amines (**A**-series) and *N*-alkyl guanidines (**G**-series). More specifically, amine moieties include methylamines, piperazines, and morpholine. We derived the guanidines from the primary and secondary amines for a direct comparison of the anti-infective profile, with some analogues featuring cyclized guanidines (**C**-series) for increased lipophilicity (Figure 2). Additionally, to gain further insights, we included uncharged compounds that do not comply with the eNTRy rules, namely, *N*-Boc (**B**-series)-protected analogues of the amines and compounds that contain alternative electron-withdrawing substituents to the trifluoromethyl of **1a**, namely, fluorine **1b** and nitro **1c**. To assess the influence of an electron-donating substituent, we included methyl-derivative **1d** and to evaluate the influence of the aromatic ring we removed it in structures **1e** and **14**.

### Synthesis

**Scheme 1:**
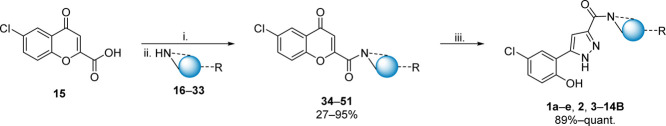
General Synthetic Scheme of Pyrazole-Amide Compounds **1a**–**e**, **2**, and **3**–**14B** *Reagents and conditions*: (i) DIPEA, HATU, DMF, 0 °C, 30 min; (ii) r.t., 2–24 h;^28^ and (iii) Hydrazine Hydrate, EtOH, Reflux, 2–18 h.^29^

**Scheme 2:**
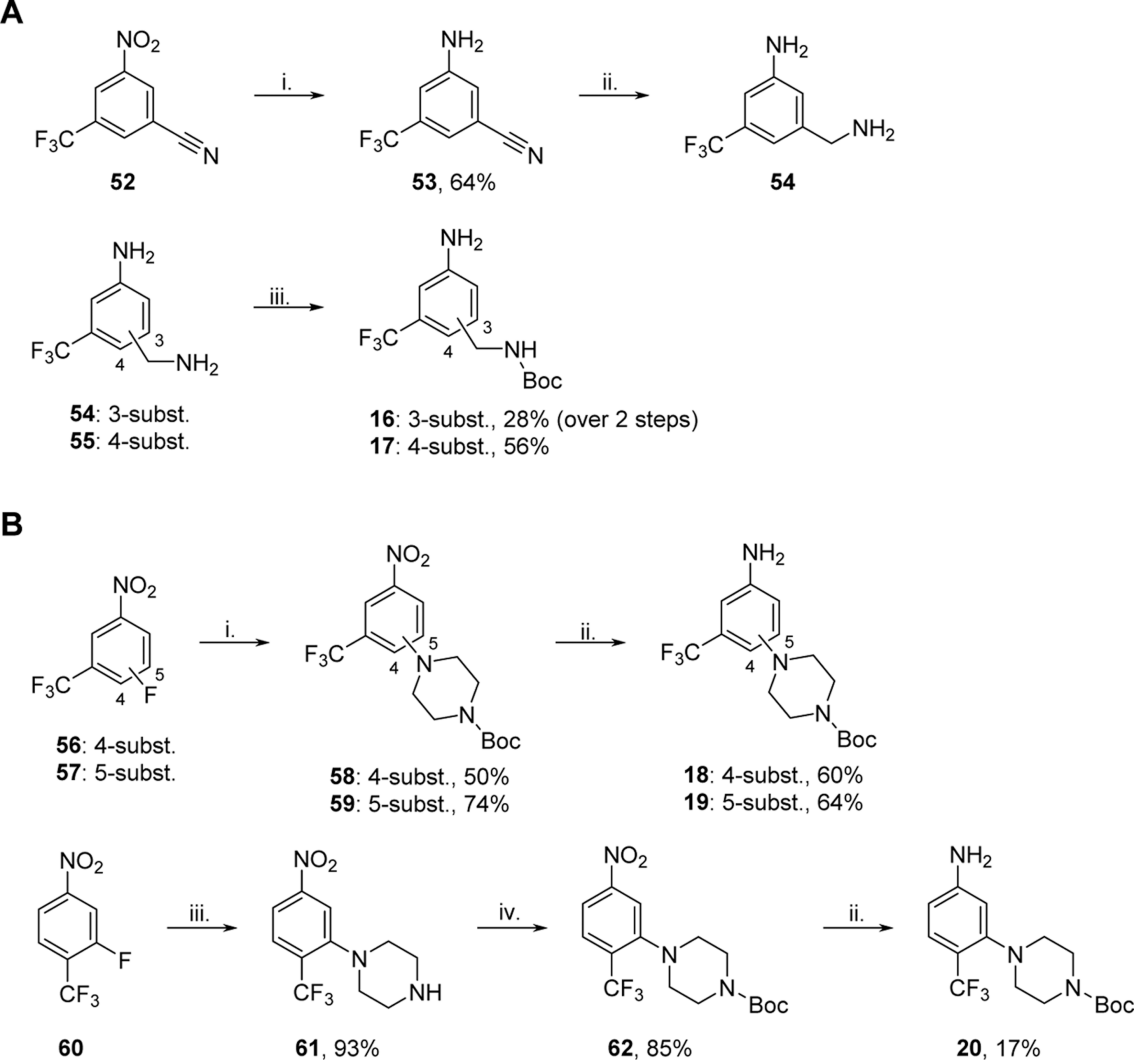
Synthesis of Anilines **16**–**20** (A) Methylamine-substituted (subst.) anilines, *reagents and conditions:* (i) Fe, NH_4_Cl, EtOH/H_2_O (2:1), reflux, 24 h; (ii) LiAlH_4_, THF, reflux, 4 h; and (iii) Boc_2_O, NEt_3_, DCM, 0 °C–r.t., 6–24 h.^30^ (B) Piperazine-substituted anilines, *reagents and conditions*: (i) 1-Boc piperazine, K_2_CO_3_, DMSO, 100 °C, 18–20 h;^31^ (ii) Na_2_S_2_O_4_, EtOH, reflux, 6 h; (iii) piperazine, K_2_CO_3_, 100 °C, DMSO, 24 h;^31^ and (iv) Boc_2_O, DMAP, DCM, r.t., 72 h.^32^

We optimized the synthesis of the designed library using key chromene amide intermediates **34**–**51**. Initially, we investigated amide couplings of pyrazole-carboxylic acid derivatives with anilines. This procedure was hampered by low yields, and purification and isolation of the products proved to be difficult. Alternatively, we used commercially available 6-chlorochromene-2-carboxylic acid (**15**) for amide coupling. Subsequent reaction with hydrazine hydrate formed pyrazole-amide products **1a**–**e**, **2**, and **3**–**14B** in quantitative yield (Scheme 1). The amines **16**–**33** used in the amide coupling were largely commercially available, however, maintaining the trifluoromethyl substituent of parent compound **1a** in addition to the ionizable nitrogen functionality, required synthesis of **16**–**20** (Scheme 2).

To minimize the formation of byproducts in the amide coupling, the methylamine- and piperazine-substituted anilines needed *N*-Boc protection, which, at the same time allowed to obtain the **B**-series (**3**–**14B**) as control compounds. To obtain aniline **16** and **17**, we selectively *N*-Boc protected methylamine analogues **54** and **55**. Analogue **54** was prepared by reducing the nitro and nitrile groups of **52**, and **55** was commercially available (Scheme 2A). We obtained piperazine-substituted anilines **18** and **19** in a two-step synthesis starting with the fluorine displacement of derivatives **56** and **57** using 1-Boc piperazine. Subsequent reduction of the nitro group using sodium dithionite gave anilines **18** and **19** in good to moderate yield. The synthesis of aniline **20** required an additional step, because the direct fluorine displacement of **60** using 1-Boc piperazine was unsuccessful. Using an excess of unsubstituted piperazine, however, followed by *N*-Boc protection afforded **62** in a good yield. Lastly, reduction of the nitro group afforded aniline **20** in a modest yield (Scheme 2B).

**Scheme 3:**
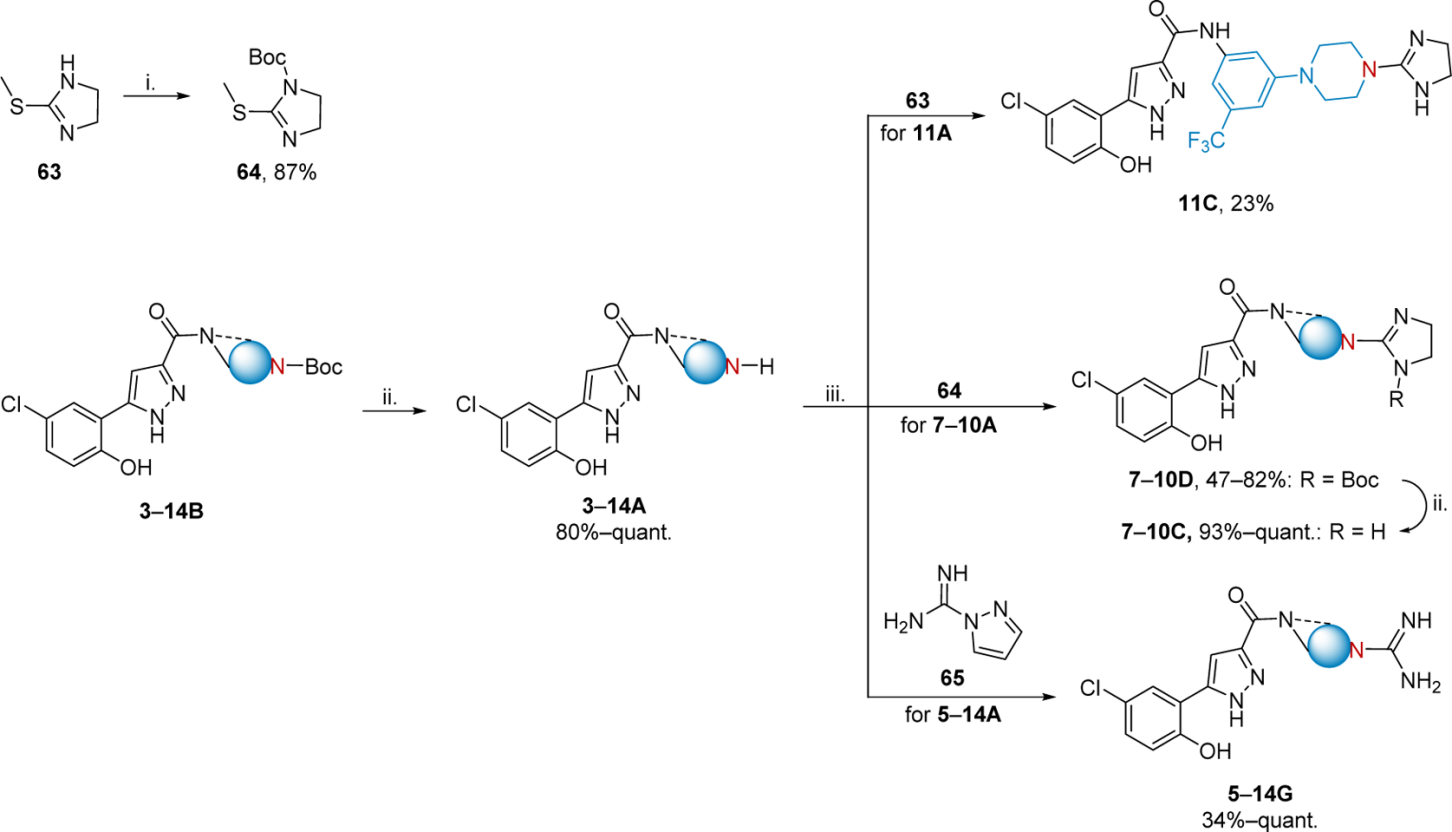
General Synthetic Scheme of Pyrazole-Amide Containing Ionizable Nitrogens: Amine (**A**), Guanidine (**G**), Cyclic-Guanidine (**C**), and *N*-Boc Cyclic-Guanidine (**D**) *Reagents and conditions*: (i) Boc_2_O, NEt_3_, DCM, r.t., 24 h;^30^ (ii) TFA, DCM, r.t., o.n.;^33^ and (iii) DIPEA, DMF, 50 °C, 2 h–6 days.^34−36^

The obtained compounds of the Boc-series (**3**–**14B**) served as intermediates providing the desired amine series as TFA salts in excellent yields (**3**–**14A**). Following a similar approach, the **A**-series was used to obtain guanidine series **C** and **G**. The initial guanidinylation strategy for the five-membered-ring guanidine series **C** yielded undesired double-guanylated products. Controlling the reaction rate for selective guanidinylation using 2-methylthio-2-imidazolin (**63**) proved challenging, leading to difficult purifications and a low yield of product **11C** (Scheme 3). To address this issue, we *N*-Boc protected **63**, obtaining the alternative guanidinylation agent **64**. This modification facilitated the synthesis of the remaining cyclic guanidines (**5**–**10C**) via their corresponding Boc analogues (**7**–**10D**) in good yield. As piperazines are more lipophilic than methylamines, we opted to exclude piperazine derivatives from the **C** and **D** series. The *N*-alkyl guanidine series **G** was accessed by employing a guanidinylation agent **65**, resulting in moderate to excellent yields (Scheme 3).

### Overview of the Anti-Infective Activity

**Figure 3:**
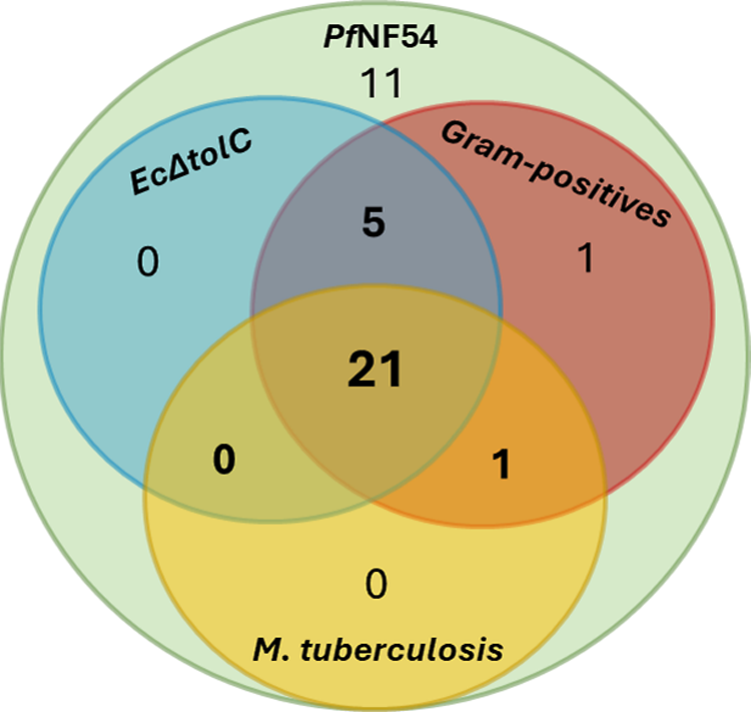
Venn diagram of the active compounds in *Pf*NF54, *Ec*Δ*tolC*, GPB, and *M. tuberculosis*, indicating the broad-spectrum anti-infective nature of our pyrazole-amide class.

To showcase how the introduction of positively charged nitrogen-containing functional groups translates into cellular activity, we assessed the anti-infective profile of our newly synthesized library. We tested the library against the parasite *P. falciparum* (strain *Pf*NF54) and various bacterial strains both Gram-negative and -positive, as well as *M. tuberculosis*. The vast majority of compounds largely retained antimalarial activity compared to the parent compound **1a**, indicating that anti-infective properties were not affected by the addition of a positive charge (Table 1). This finding gave a good foundation to determine the antibacterial efficacy of the library and evaluate the applicability of the eNTRy rules. Notably, given that the target of this compound class is unknown, negative results may reflect a lack of on-target affinity. In the case of GNB, first we tested all compounds against the efflux-pump deficient *E. coli* strain *Ec*Δ*tolC*. As the majority of positively charged compounds showed at least moderate *Ec*Δ*tolC* inhibition, we extended the panel and included the *E. coli* wild-type *Ec*K12, *A. baumannii* and *P. aeruginosa* strain PA14. Approximately half of the compounds are active against *Ec*K12, however, with a significant loss in potency compared to *Ec*Δ*tolC*, indicating efflux liabilities. Many of the *E. coli* inhibitors were also active against *A. baumannii* and PA14. Interestingly, the addition of ionizable nitrogen atoms to this class also yielded excellent activities against *M. tuberculosis* strain *Mtb*H37Rv and GPB. None of our neutral compounds presented antibacterial activity. These findings confirm that the eNTRy rules are applicable to our pyrazole-amide class. In addition, for the first time, we showed that introduction of ionizable nitrogen atoms not only affects the activity against GNB but also can be successfully expanded to GPB and *M. tuberculosis*. Excitingly, this approach yielded a new broad-spectrum anti-infective class, with many compounds being active across species. It certainly holds the potential to be expanded to other chemical classes. We illustrated the big overlap of active compounds across *Pf*NF54, *Ec*Δ*tolC*, *Mtb*H37Rv, and GPB (*S. pneumoniae* or *S. aureus*) in a Venn diagram (Figure 3). In addition, two examples (**7D**, **10G**) inhibit all eight tested pathogens, and an additional 11 compounds (**3G**, **5A**, **6G**, **9**–**11C**, **9G**, **10**–**11A**, **11G**, **13G**) inhibit all pathogens except for *P. aeruginosa*, which is known to be a particularly challenging pathogen (Table 1).

**Table 1:** Biological Activity of the Pyrazole-Amide Class in *Plasmodium falciparum* (*Pf*NF54), *Escherichia coli* (*Ec*Δ*tolC* and *Ec*K12), *Acinetobacter baumannii* (*Ab*), *Pseudomonas aeruginosa* (PA14), *Streptococcus pneumoniae* (*Sp*), *Staphylococcus aureus* (*Sa*), *Mycobacterium tuberculosis* (*Mtb*H37Rv), and Human Liver Cells (HepG2)^a^

		Gram-negative				Gram-positive			
Cmp	*Pf*NF54 IC_50_	*Ec*Δ*tolC* MIC_95_	*Ec*K12 inh. at 50 μM	*Ab* inh. at 50 μM	PA14 inh. at 50 μM	*Sp* MIC_95_	*Sa* MIC_95_	*Mtb*H37Rv MIC_90_	HepG2 CC_50_
**1a**	0.21	>50	<10%	<10%	<10%	>50	>50	n.d.	>50
**1b**	0.7	>50	n.d.	n.d.	n.d.	>50	>50	n.d.	n.d.
**1c**	0.51	>50	n.d.	n.d.	n.d.	>50	>50	>32^b^	n.d.
**1d**	2.40	>50	n.d.	n.d.	n.d.	>50	>50	n.d.	>50
**1e**	>5	>50	n.d.	n.d.	n.d.	>50	>50	>16	>50
**2**	1.1	>50	n.d.	n.d.	n.d.	>50	>50	>16^b^	∼50
**3A**	0.62	45	28%	21%	50%	40	>50	>64	12
**3B**	1.0	>50	n.d.	n.d.	n.d.	>50	>50	n.d.	25
**4A**	0.27	40	34%	24%	62%	40	>50	64	13
**4B**	0.2	>50	n.d.	n.d.	n.d.	>50	>50	n.d.	7
**5A**	0.13	21	83%	34%	60%	26	37	64	9
**5B**	0.9	>50	n.d.	n.d.	n.d.	>50	>50	n.d.	>50
**5G**	0.93	11	32%	<10%	31%	48	23.1	64	>50
**6A**	0.14	22.5	49%	37%	63%	45	>50	>16^b^	11.8
**6B**	1.61	>50	n.d.	n.d.	n.d.	>50	>50	n.d.	>50
**6G**	0.44	9	45%	32%	55%	25	26	64	>50
**7A**	0.30	47	27%	15%	44%	43	>50	>64	28.4
**7B**	1.1	>50	n.d.	n.d.	n.d.	>50	>50	n.d.	>50
**7C**	0.39	13	29%	33%	41%	48.2	22.3	32	>50
**7D**	0.36	14	56%	77%	55%	31	24.0	64	19
**7G**	0.21	13	61%	24%	56%	49.0	22	64	>50
**8A**	1.8	>50	n.d.	n.d.	n.d.	>50	>50	>16^b^	30
**8B**	1.7	>50	n.d.	n.d.	n.d.	>50	>50	n.d.	>50
**8C**	0.67	21.5	<10%	12%	10%	>50	49	>64	>50
**8D**	>5	>50	n.d.	n.d.	n.d.	>50	>50	>64	>50
**8G**	0.42	47.5	29%	19.9%	41%	>50	22	>64	>50
**9A**	0.082	8	<10%	MIC_95_ = 49	<10%	11	12.1	32	7
**9B**	0.2033	>50	n.d.	n.d.	n.d.	30	>50	>64	5.0
**9C**	0.517	7	<10%	47%	18%	15	9	16	>50
**9D**	0.15	24.0	<10%	82%	21%	21	12	16	14
**9G**	0.078	5	MIC_95_ = 46	59%	50%	>50	8	32	>50
**10A**	0.15	22.9	61%	86%	<10%	23	29	64	13
**10B**	0.19	>50	n.d.	n.d.	n.d.	>50	>50	n.d.	>50
**10C**	0.404	5.5	77%	47%	29%	14	8	16	>50
**10D**	0.14	18	17%	<10%	<10%	>50	11.6	16	11
**10G**	0.25	3.5	86%	49%	55%	16	5	8	>50
**11A**	0.05	7	72%	MIC_95_ = 22	<10%	5	6	32	9
**11B**	0.13	>50	n.d.	n.d.	n.d.	>50	>50	n.d.	4.0
**11C**	0.59	7	63%	53%	<10%	7	8	8	>50
**11G**	0.5	4	MIC_95_ = 48	MIC_95_ = 17	25%	28	3.2	8	>25
**12A**	0.06	18.9	12%	29%	<10%	8	14	16	6
**12B**	0.56	>50	n.d.	n.d.	n.d.	>50	>50	n.d.	>50
**12G**	0.2	2.8	51%	33%	<10%	31	2.4	4	30
**13A**	0.160	>50	n.d.	n.d.	n.d.	29	>50	32	8
**13B**	0.3418	>50	n.d.	n.d.	n.d.	>50	>50	n.d.	2.8
**13G**	0.5	5	59%	46%	17%	16	2.5	8	>25
**14A**	3.3	>50	n.d.	n.d.	n.d.	>50	>50	>16	>50
**14G**	>5	>50	n.d.	n.d.	n.d.	>50	>50	>64	>50

a IC_50_, MIC, and CC_50_ values are in μM.

b Not active at maximum solubility; n.d.: not determined.

### Structure–Activity Relationships

Our library was designed to investigate various functional groups, mostly containing nitrogens, and their effect on anti-infective properties through phenotypic screening. In total, we synthesized 48 compounds, 28 of which contain ionizable nitrogen atoms, consisting of 13 amines (**2**, **3**–**14A**), ten *N*-alkyl guanidines (**5**–**14G**), and five cyclic guanidines (**7**–**11C**). In addition, we tested four Boc-protected analogues (**7**–**10D**) of the cyclic guanidines (**7**–**10C**), and these functionalities are likely not ionizable in physiological conditions based on computational evaluation (p*K*_a_: ∼5.1, Figure S1). The 16 remaining compounds are also uncharged, consisting mainly of Boc-protected analogues (**3**–**14B**) of the amines (**3**–**14A**), as well as compounds **1a**–**d**, which lack ionizable nitrogen atoms altogether. Besides the nature of the functional groups, the main differences between these compounds are the motifs that contain said groups, consisting of piperazines, methylamines, and morpholine. Additionally, the substitution pattern of the motifs changes, with some examples (**9**–**13**) including the trifluoromethyl substituent present in parent compound **1a**.

#### *P. falciparum*

Our antimalarial starting point **1a** has an inhibitory concentration in the submicromolar range (*Pf*NF54 IC_50_ = 0.21 μM) which was largely retained in the dedicated library (Table 1). Fourteen compounds (**5**–**6A**, **9**–**13A**, **9**–**11B**, **9**–**10D**, **9G**, and **12G**) showed an increase in activity against *Pf*NF54, and all of them except for **5A** and **6A** retain the *meta*-CF_3_ substitution on the aromatic ring of **1a**. The two most active compounds **11A** and **12A** (IC_50_ ≤ 0.06 μM) contain a piperazine substituent, respectively, on *meta* and *para* positions. In contrast, the compounds without an aromatic ring linked to the nitrogen of the amide (**1e**, **14A**, and **14G**) are inactive, suggesting that the aromatic moiety is essential. When it comes to the aromatic ring, *para*-methylene substitution seems detrimental, methyl derivative **1d**, methylamine **8A**, and Boc-protected cyclic guanidine **8D** have a 10-fold decrease in activity compared to **1a** (IC_50_ > 2 μM). Similarly, Boc-protected amine derivatives without an additional CF_3_ substituent suffer from a significant loss in activity (**5**–**8B**: IC_50_ = 0.9–1.7 μM). Exchanging the trifluoromethyl substituent of **1a** with other electron-withdrawing groups led to a loss in activity (**1b**–**c**: IC_50_ = 0.5–0.7 μM). These findings reveal that the combination of trifluoromethyl substitution and ionizable nitrogen atom can be highly favorable for activity in *Pf*NF54 and that bigger substituents such as piperazine and *N*-Boc piperazine are well-tolerated.

#### Gram-Negative Bacteria

The *E. coli* inhibition of all 48 compounds was investigated using the efflux-pump deficient *Ec*Δ*tolC* strain. We obtained 26 hits with minimum inhibitory concentrations (MIC) in the micromolar range (MIC_95_: 2.8–47.5 μM, Table 1). None of the neutral compounds significantly affected the growth of *Ec*Δ*tolC* (Table S1), indicating that a positive charge is essential for *E. coli* activity. One possible reason supported by the eNTRy rules could be the low bioavailability of the uncharged compounds. Due to the lack of on-target activity, however, we cannot confirm this. The 11 most potent hits have a single-digit micromolar MIC and consist of nine (cyclic) guanidines (**6G**, **9**–**13G**, and **9**–**11C**) and two amine derivatives (**9A** and **11A**). Similar to *Pf*NF54, only one of the top hits does not contain an *m*-CF_3_ substituent (**6G**). In addition, the compounds with no antimalarial activity also lack activity against *E. coli*. Only two positively charged antimalarial hits are inactive against *Ec*Δ*tolC* (**2** and **13A**). These findings suggest that the engagement with its possible anti-infective target is largely consistent across these two species. In the case of *Ec*Δ*tolC* inhibition, there is a clear trend indicating that guanidine-type groups enhance potency. When comparing the different positively charged groups of identical scaffolds, the amine derivatives (**A**-series) have the lowest potencies, with one exception (MIC_95_: **9A** = 8 μM vs **9D** = 24 μM). Within the various types of guanidine functionalities (**C**-, **D**-, and **G**-series), the *N*-Boc-protected cyclic guanidine derivatives (**D**-series) are less active, with the most significant difference observed for scaffold **10** (MIC_95_: **10D** = 18 μM vs **10C** = 5.5 μM vs **10G** = 3.5 μM).

**Figure 4:**
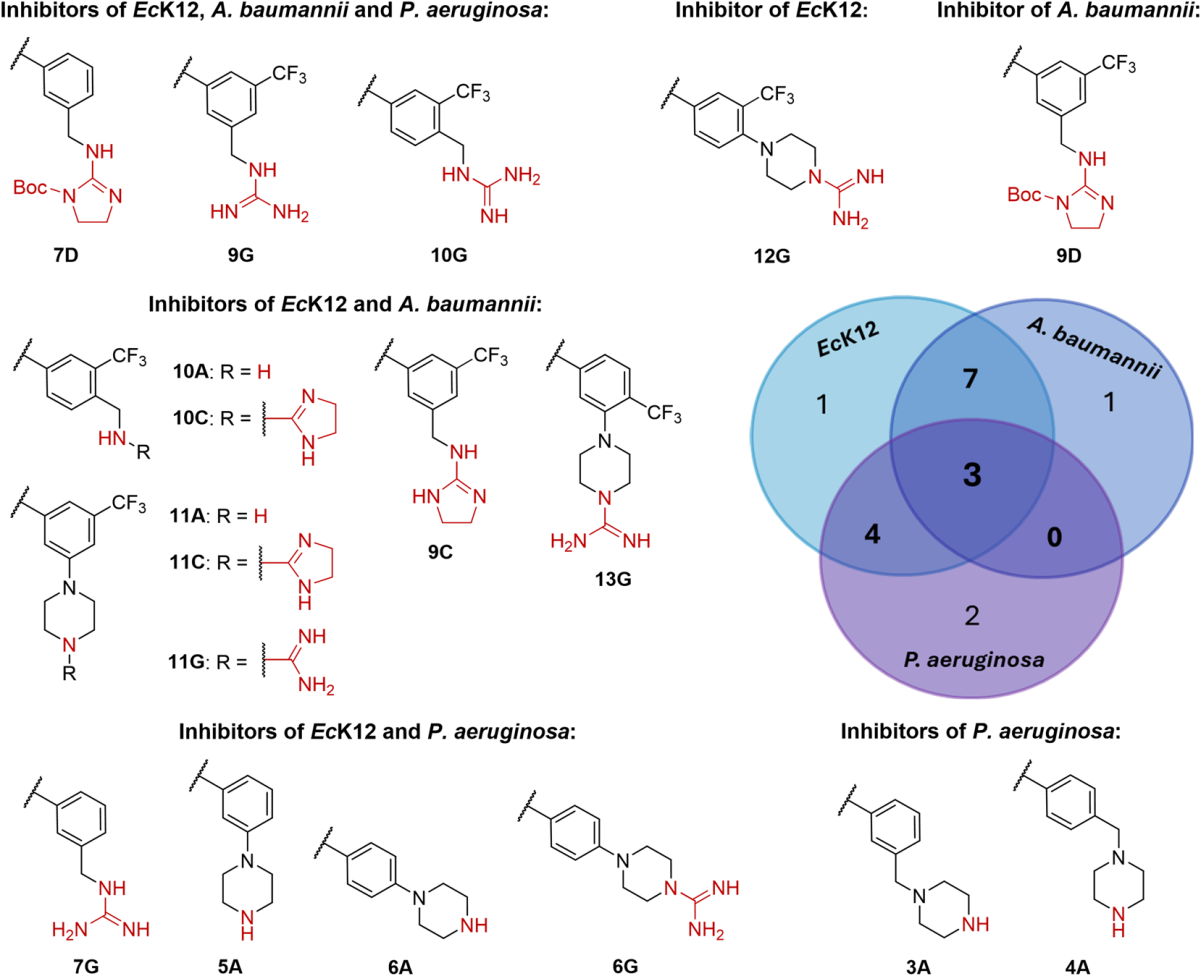
Chemical structures and Venn diagram of the active compounds in *Escherichia coli* K12 (*Ec*K12), *Acinetobacter baumannii*, and *Pseudomonas aeruginosa* (*PA14*) (≥45% inhibition at 50 μM). Potential ionizable nitrogen moieties are in red.

To further investigate the antibacterial profile and assess the applicability of the eNTRy rules, the 26 *Ec*Δ*tolC* hits (MIC_95_ < 50 μM) were tested against the *E. coli* wild-type K12, *A. baumannii* and *P. aeruginosa*. As expected, these pathogenic strains were harder to target; nevertheless, 15 hits (**5**–**6A**, **10**–**11A, 6G**, **7G, 9**–**11G, 13G**, **7D**, **9D**, and **9**–**11C**) were identified with moderate inhibition (≥45%) at 50 μM compound concentration against *Ec*K12. Structurally, we confirm once again that the guanidine functionality is beneficial for *E. coli* activity, with the two most active compounds being **9G** and **11G**. These structures have *Ec*K12 MIC_95_ values just below 50 μM (**9G** = 46 μM; **11G** = 48 μM), which indicates a 10-fold decrease in activity compared to *Ec*Δ*tolC* (**9G** = 5 μM; **11G** = 4 μM), making efflux a main concern for the activity of this class. Noteworthy, the most significant loss of activity is observed for methylamine **9A**, one of the top *Ec*Δ*tolC* inhibitors that did not show any effect on *Ec*K12 growth (**9A** Δ*tolC* MIC_95_ = 8 μM vs **9A** K12 < 10% inh. at 50 μM). A similar trend also applies to compound **9D** where the good activity against *Ec*Δ*tolC* did not translate to *Ec*K12 (**9D** Δ*tolC* MIC_95_ = 24 μM vs **9D** K12 < 10% inh. at 50 μM). These findings led us to speculate that the structural makeup of compound **9** seems to be especially prone to *tolC* efflux. In the case of *A. baumannii*, 11 compounds (**7D**, **9D**, **9**–**11C**, **9**–**11G, 13G**, and **10**–**11A**) showed a moderate (≥45% inh. at 50 μM) to good (MIC_95_ < 25 μM) activity, with **11A** and **11G** as the best hits having an MIC_95_ of 22 μM and 17 μM, respectively. Interestingly, these doubly *meta*-substituted structures are also among the best *E. coli* hits. The nine remaining *A. baumannii* inhibitors also largely contain a CF_3_ substituent and (cyclic) guanidines, with all of them except for **9D** being active against *Ec*K12. This big overlap in their inhibitory profile suggests that the bioavailability and target engagement of our pyrazole-amide class are similar in *A. baumannii* and *Ec*K12. When comparing to *P. aeruginosa*, however, the species have fewer hits in common as illustrated in the Venn diagram (Figure 4). We identified nine compounds (**3**–**6A**, **6**–**7G**, **9**–**10G**, and **7D**) with a moderate effect (≥45% inh. at 50 μM) on the growth of *P. aeruginosa* strain PA14. Three of the PA14 hits (**7D** and **9**–**10G**) are also active against the other two GNB wild-types *Ec*K12 and *A. baumannii*, and an additional four compounds (**5**–**6A** and **6**–**7G**) share activity with *Ec*K12 (Figure 4). Methylamine-derived guanidines seem to be a privileged scaffold for targeting GNB, and they appear in the three common hits across all tested GNB and in several other shared hit scaffolds of *E. coli* and *A. baumannii* (**9**–**10C**) or PA14 (**3G**). Overall, the potencies of the PA14 hits are the lowest we obtained across all pathogens (Table 1). The CF_3_ substituent and the guanidine moieties seem to be significantly less effective in targeting PA14 compared to the other GNB. In contrast, amines with (methyl)piperazine motifs yielded better results. These findings align with the structure–uptake study on oxazolidinones where they identified a CF_3_-substituted phenyl motif as a liability to *P. aeruginosa* outer membrane permeation and also concluded that *P. aeruginosa* is more divergent compared to *E. coli* and *A. baumannii*.^25^

#### Gram-Positive Bacteria

The use of Hergenrother’s eNTRy rules has mostly been reported by modifying a Gram-positive antibacterial class to comply with the three structural indications in order to obtain anti-Gram-negative activity. Therefore, we wanted to assess the GPB inhibition of our pyrazole-amide class and tested all 48 compounds against *S. aureus* and *S. pneumoniae*. Half of the compounds were active against at least one of the species, but only one example of a neutral compound (**9B**) inhibits *S. pneumoniae* (**9B**: MIC_95_ = 30 μM, Table 1). This finding suggests that the bacterial permeability of our neutral compounds is very low; however, once again, we cannot be certain due to lack of on-target affinity information. Eighteen of the 24 anti-Gram-positive hits inhibit both *S. aureus* and *S. pneumoniae*, with potencies against *S. aureus* being generally higher and guanidines being particularly favorable. Guanidines **9**–**10C** and **9**–**13G** have excellent single digit micromolar MIC_95_ values against *S. aureus*. In addition, **11A** and **11C** are among the best hits against both species and amine **12A** in *S. pneumoniae*. These findings align with previous trends and highlight the favorable combination of trifluoromethyl and positively charged motifs for antibacterial activity. In the case of *S. pneumoniae*, piperazine substituents are especially advantageous.

#### *M. tuberculosis*

To obtain an even wider scope of the anti-infective profile of our chemical class, we assessed its antitubercular activity using the *M. tuberculosis* strain *Mtb*H37Rv and obtained 22 hits. The five best *Mtb*H37Rv inhibitors (**10**–**13G** and **11C**) have comparable potencies to the best Gram-positive and *Ec*Δ*tolC* hits, with an MIC_90_ value of 4 μM, and **12G** is the most potent *Mtb*H37Rv inhibitor (Table 1). The tendency of CF_3_- and guanidine-containing structures to be especially active persists, and overall, there is a big overlap in hit compounds shared between *Mtb*H37Rv, GPB, and *Ec*Δ*tolC* (Figure 3). Our neutral compounds were not sufficiently soluble in the *M. tuberculosis* growth medium and could not be evaluated (Table S2). Similarly, four amines had low solubilities (16–32 μM: **2**, **4A**, **6A**, and **12A**) and did not show an effect on the growth of *Mtb*H37Rv at the testable concentrations (Table 1). To the best of our knowledge, this is the first record of applying the eNTRy rules to gain antitubercular activity, which was encouraged in a review on the hurdles of antitubercular drug development from 2020.^37^ However, similarly to the other tested pathogens, we cannot rule out that the ionizable nitrogen functionalities give rise to the antibacterial effect and not only to the bacterial uptake, especially considering that the cell wall of *M. tuberculosis* is particularly lipophilic and hard to permeate for drug-like compounds.^38^

#### Cytotoxicity

To gain insight into the toxicity of the class, the impact on the viability of the human liver cell line HepG2 was evaluated for all compounds. Generally, our most potent hits were nontoxic with cytotoxic concentrations (CC_50_) > 50 μM (Table 1). However, we did identify a major cytotoxic liability. All amine derivatives had a toxic effect on liver cells; in the worst cases, the CC_50_ values reached the single-digit micromolar range. Interestingly, the majority of Boc and guanidine analogues were not toxic, suggesting that the liability stems directly from the amine functional groups. This is exemplified when comparing the toxicities of structures **5**–**8**. Compounds **5** and **6** contain a piperazinyl-substituted phenyl, in *meta* and *para* position. In the closely related structures **7** and **8**, a methylene linker separates the piperazinyl from the aromatic ring, which results in both piperazine nitrogen atoms being aliphatic amines. In this case, both amine (**7**–**8A**) and Boc (**7**–**8B**) derivatives are toxic. In contrast, Boc and guanidine derivatives **5**–**6B** and **5**–**6G** are nontoxic, whereas amine analogues **5**–**6A** are toxic, indicating that aniline-like nitrogen atoms are devoid of hepatoxicity.

We investigated the toxicity of our best *Mtb*H37Rv inhibitors further by testing their effect on human monocyte-derived macrophages (**10**–**13G**, Table S2). None of the tested compounds were of major concern, and solely **12G** exhibits a CC_90_ of 32 μM, which is manageable given that it is an 8-fold difference in activity compared to *Mtb*H37Rv.

## Conclusions

We report the design, synthesis, and evaluation of a targeted library of positively charged pyrazole amides against *P. falciparum*, *E. coli*, *A. baumannii*, *P. aeruginosa*, *S. pneumoniae*, *S. aureus*, and *M. tuberculosis*. Through phenotypic screenings, we identified broad-spectrum anti-infective activity of the new pyrazole-amide class, indicating its diverse membrane permeability. We successfully implemented or enlarged the eNTRy rules to an antimalarial compound as a model example and we proved that our newly synthesized derivatives are active not only against GNB but also GPB and *M. tuberculosis*. Specifically, the best ionizable nitrogen-containing functional group for our chemical class was *N*-alkyl guanidines. Furthermore, for the first time, we showed that cyclized guanidines can also aid in bacterial uptake, opening the door to a novel and easily accessible chemical moiety that could improve anti-infective activity. We identified three compounds (**3D** and **9**–**10G**) with activity in all of the tested GNB. Guanidine **12G** is the most potent *Mtb*H37Rv, *Ec*Δ*tolC*, and *S. aureus* hit with low single-digit micromolar activities in all three species while maintaining the antimalarial potency of the parent compound **1a**. We observed the biggest SAR variations in *P. aeruginosa*, where guanidine or trifluoromethyl substitution seemed detrimental to activity as opposed to the rest of the pathogens. Nevertheless, further evaluation of molecular properties that dictate compounds’ bioavailabilities across different pathogens is needed to better understand the applicability and limitations of existing guidelines and expand them. At the same time, the target identification and mode of action of the pyrazole-amide class is necessary for future hit optimization and better rationalization of the SAR.

## Experimental Section

### General Procedure for the Synthesis of Pyrazole-Amide Inhibitors

#### General Procedure for Pyrazole Formation (GP-1)

The synthesis of the pyrazoles was prepared following a similar procedure reported in the literature.^29^

The respective chromene amide (1 equiv) was suspended in EtOH (0.1 M), and hydrazine hydrate (8 equiv) was added dropwise. The reaction mixture was heated to reflux, and after the reaction was completed, the mixture was allowed to cool to room temperature (r.t.). The solvent was removed under reduced pressure to obtain the pure product, without purification unless stated otherwise, in excellent yields (>95%).

#### General Procedure for Boc Deprotection (GP-2)

The *N*-Boc deprotections were completed following a similar procedure reported in the literature.^33^

The respective *N*-Boc-protected product (1 equiv) was dissolved in a mixture of trifluoroacetic acid (TFA) and dichloromethane (DCM) (1:4, 0.1 M) and cooled to 0 °C in an ice–water bath. The reaction mixture was stirred while allowing to reach r.t. After the reaction was completed, the solvents were removed under reduced pressure to obtain the pure products as TFA salts in excellent yield (>95%).

#### General Procedure for Guanidinylation (GP-3)

The guanidinylation of amines was completed following similar procedures reported in the literature.^34−36^

The respective amine TFA salt (1 equiv) was stirred in DMF (0.1 M) and DIPEA (1.5–8.1 equiv), and the respective guanidinylation agent (1.4–3.0 equiv) was added. The reaction mixture was heated to 50 °C and, after completion, was allowed to cool to r.t. The excess solvent was removed under reduced pressure, and ice-cold water (5–20 mL) was added to the mixture. The resulting precipitate was filtered to obtain the pure product as TFA salt, without purification unless stated otherwise, in good to excellent yield (46%—quantitative).

#### General Procedure for Amide Coupling (GP-4)

The synthesis of the chromene amides was prepared following a similar procedure reported in the literature.^28^

6-Chloro-4-oxo-4*H*-chromene-2-carboxylic acid **15** (1.05 equiv) was suspended in DMF (0.1 M) and DIPEA was added (1.2 equiv). The mixture was cooled to 0 °C in an ice–water bath, and 2-(3*H*-[1,2,3]triazolo[4,5-*b*]pyridin-3-yl)-1,1,3,3-tetramethylisouronium (HATU, 1.2 equiv) was added. The yellow solution was stirred for 30 min, and the corresponding aniline (1 equiv) was added. The reaction mixture was stirred while allowing to reach r.t. After the reaction was completed, the mixture was added to water (25–100 mL), and the resulting precipitate was filtered and washed with solvent. When necessary, the crude was purified by flash column chromatography. The respective chromene amides were obtained in low to excellent yields (27–95%).

Details of the synthesis and characterization of pyrazole-amide inhibitors can be found in the Supporting Information.

## Supporting Information Available

Detailed synthetic and biological assay procedures, HRMS, LCMS, H^1^ and C^13^ NMR spectra, growth inhibition, and standard deviation (PDF)

## Notes

This article is based on Chapter 2 of M. Braun-Cornejo’s Ph.D. Thesis.^39^
