# Supplementary material for: Positive Charge in an Antimalarial Compound Unlocks Broad-Spectrum Antibacterial Activity
Source: JACS Au. 2025 Feb 21;5(3):1146–56. doi: 10.1021/jacsau.4c00935 (PMC11938010; doi:10.1021/jacsau.4c00935)
Supplement: Supplementary file 1 — au4c00935_si_001.pdf [file au4c00935_si_001.pdf]

Supporting Information

A Positive Charge in an Antimalarial Compound Unlocks Broad-spectrum Antibacterial Activity

Maria Braun-Cornejo,[1,2,3] Mitchell Platteschorre,[1] Vincent de Vries,[1] Patricia Bravo,[4,5] Vidhisha Sonawane,[6] Mostafa M. Hamed,[3] Jörg Haupenthal,[3] Norbert Reiling,[6,7] Matthias Rottmann,[4] Dennis Piet,[1] Peter Maas,[1] Eleonora Diamanti,[3] and Anna K. H. Hirsch.[2,3*]

[1] Specs Compound Handling, B.V., Bleiswijkseweg 55, 2712 PB Zoetermeer, The Netherlands.

[2] Department of Pharmacy, Saarland University, Campus Building E8.1, 66123 Saarbrücken, Germany.

[3] Helmholtz Institute for Pharmaceutical Research Saarland (HIPS) – Helmholtz Centre for Infection Research (HZI), Campus Building E8.1, 66123 Saarbrücken, Germany.

[4] Swiss Tropical and Public Health Institute Kreuzstrasse 2, 4123 Allschwil, Switzerland.

[5] Universität Basel, Petersplatz 1, 4003 Basel, Switzerland.

[6] Microbial Interface Biology, Research Center Borstel, Leibniz Lung Center, 23845 Borstel, Germany.

[7] German Center for Infection Research (DZIF), Partner Site Hamburg-Lübeck-Borstel-Riems, 23845 Borstel, Germany.

*Corresponding author: Anna.Hirsch@helmholtz-hips.de

Table of Contents

Supplementary Methods 3
- General information 3
- Chemicals 3
- General procedure for Synthesis 3
- Characterisation of products 4
  - Final Products **1–14** 4
  - Intermediates **16–62** 18
- *P. falciparum* culturing and *in vitro* drug sensitivity assay on asexual blood stage parasites 26
- Determination of *in vitro* antibacterial activity 26
- Determination of *in vitro* anti-tubercular activity and solubility in 7H9 medium 26
- Cytotoxicity assay 26
- Measurement of *in vitro* cytotoxicity by XTT assay 27

Supplementary Tables and Figures 27
- Computational evaluation of pKa 27
- Biological evaluation of compounds 28
- NMR, HRMS, and LCMS spectra 30
  - Compounds **1a–e** 30
  - Compound **2** 39
  - Compounds **3A–B** 40
  - Compounds **4A–B** 43

Compounds **5A–G** 46
Compounds **6A–G** 51
Compounds **7A–G** 55
Compounds **8A –G** 63
Compounds **9A–G** 71
Compounds **10A–G** 82
Compounds **11A–G** 92
Compounds **12A–G** 100
Compounds **13A–G** 106
Compounds **14A–G** 112
Supplementary References 115

Supplementary Methods

General information

All reactions were conducted under nitrogen atmosphere using oven-dried glassware. The reaction progress was monitored on thin layer chromatography (TLC) on silica gel-coated aluminum (silica gel F254, SiliCycle). Purification of the final products, when necessary, was performed by flash column chromatography using using silica gel (Screening Devices 60-200 µm) or by preparative HPLC (Dionex UltiMate 3000 UHPLC+ focused, Thermo Scientific) on a reversed-phase column (C18 column, 5 µM, Macherey-Nagel, Germany). The solvents used for the chromatography were water (0.1% formic acid) and MeCN (0.1% formic acid). High-resolution mass (HRMS) of final products was determined by HPLC-MS/MS using a Thermo Scientific Q Exactive Focus Orbitrap LC-MS/MS system. All compounds were analysed for purity by LCMS on Acquity UPLC-SQD system from Waters with a gradient elution of Water (Formic acid 0.1%)/Acetonitrile on an HSS-T3 column (2.1 x 50 mm, Waters), 1.8 µm, at 30 °C, PDA detection between 240-320 nm, and MS detection by simultaneous ES+/ES- ionization in a mass range of 150-800. The flow was set to 0.9 ml/min, and the gradient time is 1.5 min. NMR spectra were recorded on an Agilent 400 MHz or a Bruker Avance Neo 500 MHz. Chemical shifts (δ) are reported in ppm relative to residual solvent signals. The following abbreviations are used to describe peak patterns when appropriate: s (singlet), d (doublet), t (triplet), q (quartet), quint (quintet), sex (sextet), sept (septuplet), m (multiplet), br (broad). Coupling constants (J) are reported in Hertz (Hz). Reactions were monitored with thin layer chromatography (TLC) on silica gel-coated aluminum (silica gel F254, SiliCycle).

Chemicals

All reagents and solvents were purchased from Sigma-Aldrich, Specs, Fluorochem, or Acros Organics, were reagent grade, and used without purification unless indicated otherwise. Reagents and substrates were purchased from commercial sources and used as received. Solvents not required to be dry were purchased as technical grade and used as received. Dry solvents were purchased from commercial sources in Sure/SealTM bottles and used as received and stored under a dry inert gas (N_2 or Ar). All new compounds were fully characterised by ^1H and ^{13}C NMR and HRMS techniques. The purity of the final products was determined by HPLC-MS and found to be >95%.

General procedure for Synthesis

General procedure for pyrazole formation (GP-1)

The synthesis of the pyrazoles was prepared following a similar procedure reported in the literature.[1]
The respective chromene amide (1 eq.) was suspended in EtOH (0.1 M), and hydrazine hydrate (8 eq.) was added dropwise. The reaction mixture was heated to reflux, after the reaction was completed the mixture was allowed to cool to room temperature (r.t.). The solvent was removed under reduced pressure to obtain the pure product, without purification unless stated otherwise, in excellent yields (>95%).

General procedure for Boc deprotection (GP-2)

The *N*-Boc deprotections were completed following a similar procedure reported in the literature.[2]
The respective *N*-Boc protected product (1 eq.) was dissolved in a mixture of trifluoroacetic acid (TFA) and dichloromethane (DCM) (1:4, 0.1 M) and cooled to 0 °C in an ice-water bath. The reaction mixture stirred while allowing to reach r.t. After the reaction was completed, the solvents were removed under reduced pressure to obtain the pure products as TFA salts in excellent yields (>95%).

General procedure for guanidinylation (GP-3)

The guanidinylation of amines were completed following similar procedures reported in the literature.[3–5]
The respective amine TFA salt (1 eq.) was stirred in DMF (0.1 M), DIPEA (1.5–8.1 eq.) and the respective guanidinylation agent (1.4–3.0 eq.) was added. The reaction mixture was heated to 50 °C and after completion allowed to cool to r.t. The excess solvent was removed under reduced pressure and ice-cold water (5–20 mL) was added to the mixture. The resulting precipitate was filtered to obtain the pure product as TFA salt, without purification unless stated otherwise, in good to excellent yields (46%–quantitative).

General procedure for amide coupling (GP-4)

The synthesis of the chromene amides was prepared following a similar procedure reported in the literature.[6]
6-Chloro-4-oxo-4*H*-chromene-2-carboxylic acid **15** (1.05 eq.) was suspended in DMF (0.1 M) and DIPEA was added (1.2 eq.). The mixture was cooled to 0 °C in an ice-water bath, and 2-(3*H*-[1,2,3]triazolo[4,5-*b*]pyridin-3-yl)-1,1,3,3-tetramethylisouronium (HATU,1.2 eq.) was added. The yellow solution stirred for 30 min and the corresponding aniline (1 eq.) was added. The reaction mixture stirred while allowing to

reach r.t. After the reaction was completed, the mixture was added to water (25–100 mL), and the resulting precipitate was filtered and washed with solvent. When necessary, the crude was purified by flash column chromatography. The respective chromene amides were obtained in low to excellent yields (27–95%).

Characterisation of products

Final Products 1–14

5-(5-Chloro-2-hydroxyphenyl)-*N*-(3-(trifluoromethyl)phenyl)-1*H*-pyrazole-3-carboxamide (**1a**):

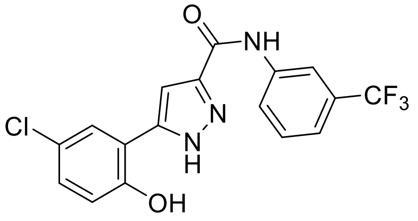

Compound **1a** was prepared following GP-1, chromene amide **34** (76 mg, 0.21 mmol) was reacted with hydrazine hydrate (83 mg, 1.7 mmol) and the reaction mixture stirred for 1.5 h at reflux. The solvent was removed under reduced pressure to obtain **1a** as white solid in quantitative yield (80 mg, 0.21 mmol).

^{1}H NMR (400 MHz, DMSO-*d*$_6$) δ = 10.50 (br s, 1H), 8.30 (br s, 1H), 8.11 (br d, *J* = 7.8 Hz, 1H), 7.81 (d, *J* = 2.0 Hz, 1H), 7.59 (br t, *J* = 7.8 Hz, 1H), 7.44 (br d, *J* = 7.8 Hz, 1H), 7.41 (br s, 1H), 7.25 (br dd, *J* = 8.6, 2.0 Hz, 1H), 7.02 (br d, *J* = 8.6 Hz, 1H) ppm.

^{13}C NMR (101 MHz, DMSO-*d*$_6$) δ = 160.9, 153.2, 146.8, 139.7, 129.8, 129.5, 129.2, 128.2, 126.6, 125.5, 123.7, 122.9, 119.6, 118.1, 117.1, 116.3, 106.2 ppm.

^{19}F NMR (470 MHz, DMSO-*d*$_6$) δ = -61.25 ppm.

HRMS (ESI$^+$): *m*/z calcd. for $C_{17}H_{12}ClF_3N_3O_2^+$ ([*M*+H]$^+$) 382.0565, measured 382.0553.

5-(5-Chloro-2-hydroxyphenyl)-*N*-(3-fluorophenyl)-1*H*-pyrazole-3-carboxamide (**1b**):

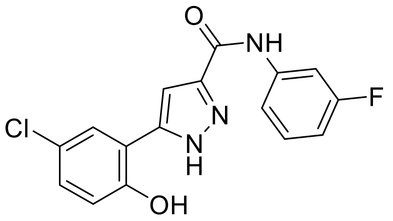

Compound **1b** was prepared following GP-1, chromene amide **35** (335 mg, 1.05 mmol) was reacted with hydrazine hydrate (588 mg, 8.5 mmol) and the reaction mixture stirred for 2 h at reflux. The solvent was removed under reduced pressure to obtain **1b** as off-white solid (345 mg, 1.04 mmol, 99%).

^{1}H NMR (500 MHz, DMSO-*d*$_6$) δ = 10.36 (br s, 1H), 7.81 (d, *J* = 2.4 Hz, 1H), 7.79 (s, 1H), 7.64 (br d, *J* = 7.8 Hz, 1H), 7.41 (br s, 1H), 7.38 (q, *J* = 7.8 Hz, 1H), 7.25 (dd, *J* = 8.7, 2.4 Hz, 1H), 7.01 (d, *J* = 8.7 Hz, 1H), 6.92 (br t, *J* = 7.8 Hz, 1H) ppm.

^{13}C NMR (101 MHz, DMSO-*d*$_6$) δ = 163.2, 160.8, 153.3, 140.6, 140.5, 130.2, 130.1, 128.9, 126.5, 122.9, 118.1, 115.9, 109.9, 107.0, 106.8, 106.0 ppm.

^{19}F NMR (470 MHz, DMSO-*d*$_6$) δ = -112.27 ppm.

HRMS (ESI$^+$): *m*/z calcd. for $C_{16}H_{12}ClFN_3O_2^+$ ([*M*+H]$^+$) 332.0597, measured 332.0580.

5-(5-Chloro-2-hydroxyphenyl)-*N*-(3-nitrophenyl)-1*H*-pyrazole-3-carboxamide (**1c**):

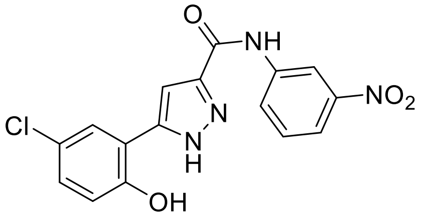

Compound **1c** was prepared following GP-1, chromene amide **36** (74 mg, 0.21 mmol) was reacted with hydrazine hydrate (86 mg, 1.72 mmol). The reaction mixture stirred for 1.5 h at reflux. The solvent was removed under reduced pressure obtaining **1c** as yellow powder (76 mg, 0.21 mmol, 99%).

^{1}H NMR (400 MHz, DMSO-*d*$_6$) δ = 10.64 (br s, 1H), 8.88 (br s, 1H), 8.25 (br s, 1H), 7.93 (br s, 1H), 7.80 (br s, 1H), 7.64 (br s, 1H), 7.42 (br s, 1H), 7.22 (br s, 1H), 7.01 (br s, 1H) ppm.

^{13}C NMR (101 MHz, DMSO-*d*$_6$) δ = 160.3, 153.7, 147.9, 144.2, 142.4, 140.1, 130.0, 128.7, 126.4, 126.2, 122.6, 118.13, 118.06, 117.9, 114.3, 105.8 ppm.

HRMS (ESI$^+$): *m*/z calcd. for $C_{16}H_{12}ClN_4O_4^+$ ([*M*+H]$^+$) 359.0542, measured 359.0532.

5-(5-Chloro-2-hydroxyphenyl)-*N*-(p-tolyl)-1*H*-pyrazole-3-carboxamide (**1d**):

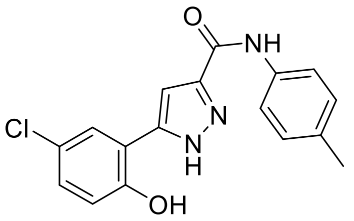

Compound **1d** was prepared following GP-1 with adaptations, chromene amide **37** (218 mg, 0.70 mmol) was reacted with hydrazine hydrate (278 mg, 3.48 mmol), and the reaction mixture stirred for 1 h at reflux. A precipitate appeared, which was filtered and washed with ice-cold water (1.5 mL) to obtain **1d** as white powder (203 mg, 0.62 mmol, 89%).

^{1}H NMR (400 MHz, DMSO-d_6) δ = 10.05 (br s, 1H), 7.80 (d, J = 2.3 Hz, 1H), 7.69 (br d, J = 8.2 Hz, 2H), 7.44 (br s, 1H), 7.24 (dd, J = 8.6, 2.3 Hz, 1H), 7.15 (br d, J = 8.2 Hz, 2H), 7.01 (d, J = 8.6 Hz, 1H), 2.28 (s, 3H) ppm.
^{13}C NMR (101 MHz, DMSO-d_6) δ = 153.8, 136.6, 133.0, 129.5, 129.2, 126.9, 123.4, 123.3, 120.7, 118.5, 106.1, 20.9 ppm. Quaternary carbon peaks missing.
HRMS (ESI$^+$): m/z calcd. for $C_{17}H_{15}ClN_3O_2^+$ ([M+H]$^+$) 328.0847, measured 328.0830.

(5-(5-Chloro-2-hydroxyphenyl)-1*H*-pyrazol-3-yl)(morpholino)methanone (**1e**):

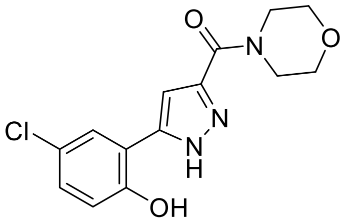

Compound **1e** was prepared following GP-1 with adaptations, chromene amide **38** (64 mg, 0.22 mmol) was reacted with hydrazine hydrate (95 mg, 1.9 mmol), and the reaction mixture stirred for 2 h at reflux. A precipitate appeared, which was filtered and washed with ice-cold water (1 mL) to obtain **1e** as white powder (65 mg, 0.21 mmol, 97%).
^{1}H NMR (400 MHz, DMSO-d_6) δ = 7.78 (s, 1H), 7.22 (br d, J = 8.6 Hz, 1H), 7.14 (s, 1H), 6.98 (d, J = 8.6 Hz, 1H), 3.92 (br s, 4H), 3.34 (br s, 4H) ppm.
^{13}C NMR (151 MHz, DMSO-d_6) δ = 161.4, 153.3, 146.1, 139.4, 128.8, 126.5, 122.9, 118.0,117.8 106.6, 66.4, 66.2, 47.1, 42.3 ppm.
HRMS (ESI+): m/z calcd. for $C_{14}H_{15}ClN_3O_3^+$ ([M+H]$^+$) 308.0796, measured 308.0776.

5-(5-Chloro-2-hydroxyphenyl)-*N*-(3-(morpholinomethyl)phenyl)-1*H*-pyrazole-3-carboxamide (**2**):

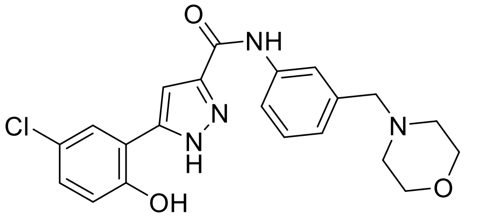

Compound **2** was prepared following GP-1, chromene amide **39** (277 mg, 0.69 mmol) was reacted with hydrazine hydrate (275 mg, 5.5 mmol) and the reaction mixture stirred at reflux overnight. The solvent was removed under reduced pressure to obtain **2** as light pink solid in quantitative yield (284 mg, 0.69 mmol).
^{1}H NMR (400 MHz, DMSO-d_6) δ = 10.22 (br s, 1H), 7.79 (br s, 1H), 7.76 (br s, 1H), 7.70 (d, J = 7.6 Hz, 1H), 7.44 (br s, 1H), 7.25 (t, J = 7.6 Hz, 1H), 7.18 (br d, J = 8.2 Hz, 1H), 7.10 (br d, J = 8.2 Hz, 1H), 6.99 (d, J = 7.6 Hz, 1H), 3.54 (br s, 4H), 3.42 (br s, 2H), 2.34 (br s, 4H) ppm.
^{13}C NMR (101 MHz, DMSO-d_6): δ = 161.5, 154.0, 139.1, 138.6, 129.0, 128.8, 126.8, 124.7, 123.2, 121.2, 119.4, 118.6, 106.5, 66.6, 62.9, 53.6 ppm. Quaternary carbon peaks missing.
HRMS (ESI+): m/z calcd. for $C_{21}H_{22}ClN_4O_3^+$ ([M+H]$^+$) 413.1375, measured 413.1359.

5-(5-Chloro-2-hydroxyphenyl)-*N*-(3-(piperazin-1-ylmethyl)phenyl)-1*H*-pyrazole-3-carboxamide (**3A**):

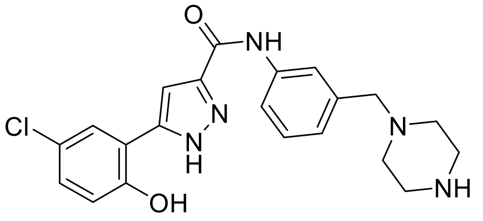

Compound **3A** was prepared following GP-2, *N*-Boc amine **3B** (214 mg, 0.42 mmol) was reacted with TFA (6.3 mmol) and the reaction mixture stirred for 2 h. Compound **3A** was obtained as white powder in form of a TFA salt (257 mg, 0.40 mmol, 96%).
^{1}H NMR (400 MHz, DMSO-d_6) δ = 10.64 (br s, 1H), 10.26 (s, 1H), 8.95 (br s, 1H), 7.98 (br s, 1H), 7.81 (d, J = 2.3 Hz, 1H), 7.78 (s, 1H), 7.43 (br s, 1H), 7.37-7.42 (m, 1H), 7.25 (dd, J = 9.0, 2.3 Hz, 1H), 7.18 (br d, J = 7.0 Hz, 1H), 7.03 (d, J = 9.0 Hz, 1H), 4.10 (br s, 2H), 3.28 (br s, 4H), 3.09 (br s, 4H) ppm.
^{13}C NMR (101 MHz, DMSO-d_6) δ = 159.0, 153.3, 139.1, 132.5, 131.1, 129.1, 128.9, 126.5, 125.8, 123.0, 122.3, 120.8, 118.1, 105.9, 60.0, 48.2, 41.4 ppm. Quaternary carbon peaks missing.
HRMS (ESI$^+$): m/z calcd. for $C_{21}H_{23}ClN_5O_2^+$ ([M+H]$^+$) 412.1535, measured 412.1529.

tert-Butyl 4-(3-(5-(5-chloro-2-hydroxyphenyl)-1*H*-pyrazole-3-carboxamido)benzyl)piperazine-1-carboxylate (**3B**):

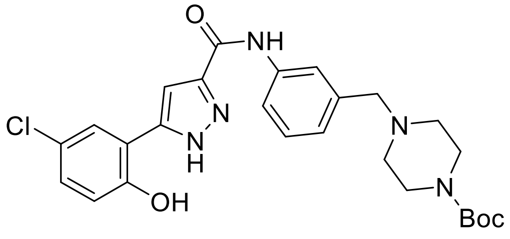

Compound **3B** was prepared following GP-1, chromene amide **40** (114 mg, 0.23 mmol) was reacted with hydrazine hydrate (92 mg, 1.83 mmol). The reaction mixture stirred for 15 min at reflux. The solvent was removed under reduced pressure to obtain **3B** as off-white powder in quantitative yield (125 mg, 0.23 mmol).

^{1}H NMR (400 MHz, DMSO-d_6) δ = 10.10 (br s, 1H), 7.70-7.81 (m, 3H), 7.42 (s, 1H), 7.29 (t, J = 7.8 Hz, 1H), 7.21 (br d, J = 7.8 Hz, 1H), 6.96-7.06 (m, 2H), 3.47 (s, 2H), 3.31 (br s, 4H), 2.32 (br s, 4H), 1.38 (br s, 9H) ppm.
^{13}C NMR (101 MHz, DMSO-d_6) δ = 159.5, 154.0, 153.9, 143.9, 143.3, 138.7, 138.4, 128.6, 128.4, 126.3, 124.2, 122.5, 120.6, 119.0, 118.5, 118.2, 105.2, 78.8, 62.1, 52.4, 28.1 ppm.
HRMS (ESI$^+$): m/z calcd. for $C_{26}H_{31}ClN_5O_4^+$ ([M+H]$^+$) 512.2059, measured 512.2050.

5-(5-Chloro-2-hydroxyphenyl)-*N*-(4-(piperazin-1-ylmethyl)phenyl)-1*H*-pyrazole-3-carboxamide (**4A**):

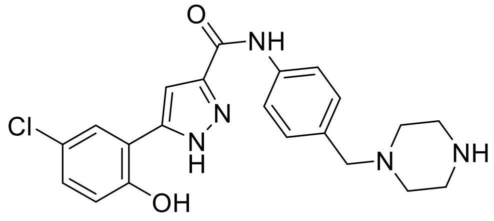

Compound **4A** was prepared following GP-2, *N*-Boc amine **4B** (153 mg, 0.30 mmol) was reacted with TFA (4.8 mmol) and the reaction mixture stirred for 1 h. Compound **4A** was obtained as beige powder in form of a TFA salt in quantitative yield (194 mg, 0.30 mmol).
^{1}H NMR (400 MHz, DMSO-d_6) δ = 10.72 (br s, 1H), 10.31 (br s, 1H), 9.19 (br s, 1H), 7.89 (d, J = 8.2 Hz, 2H), 7.81 (d, J = 2.4 Hz, 1H), 7.47 (br s, 1H), 7.44 (br d, J = 8.2 Hz, 2H), 7.24 (dd, J = 8.8, 2.4 Hz, 1H), 7.03 (d, J = 8.8 Hz, 1H), 4.12 (br s, 2H), 3.32 (br s, 4H), 3.13 (br s, 4H) ppm.
^{13}C NMR (101 MHz, DMSO-d_6) δ = 159.5, 153.8, 139.9, 131.6, 129.3, 126.9, 123.3, 123.3, 120.6, 118.5, 106.4, 59.7, 48.3, 41.5 ppm. Quaternary carbon peaks missing.
HRMS (ESI$^+$): *m*/*z* calcd. for $C_{21}H_{23}ClN_5O_2^+$ ([*M*+H]$^+$) 412.1535, measured 412.1509.

tert-Butyl 4-(4-(5-(5-chloro-2-hydroxyphenyl)-1*H*-pyrazole-3-carboxamido)benzyl)piperazine-1-carboxylate (**4B**):

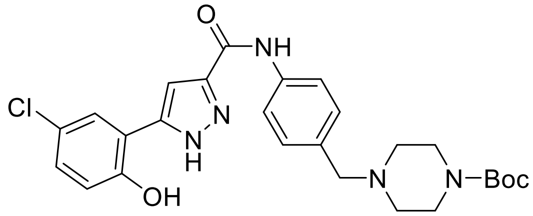

Compound **4B** was prepared following GP-1, chromene amide **41** (178 mg, 0.36 mmol) was reacted with hydrazine hydrate (143 mg, 2.86 mmol). The solvent was removed under reduced pressure to obtain **4B** as off-white powder in quantitative yield (183 mg, 0.36 mmol).
^{1}H NMR (400 MHz, DMSO-d_6) δ = 10.13 (br s, 1H), 7.80 (d, J = 2.0 Hz, 1H), 7.76 (d, J = 7.8 Hz, 2H), 7.34-7.59 (m, 1H), 7.26 (d, J = 7.8 Hz, 2H), 7.21-7.25 (m, 1H), 7.01 (br d, J = 8.6 Hz, 1H), 3.44 (br s, 2H), 3.31 (br s, 4H), 2.30 (br s, 4H), 1.38 (s, 9H) ppm.
^{13}C NMR (101 MHz, DMSO-d_6) δ = 154.3, 153.8, 138.0, 133.4, 129.6, 129.2, 126.9, 123.4, 120.5, 118.5, 106.1, 79.2, 62.0, 52.7, 28.5 ppm.
HRMS (ESI$^+$): *m*/*z* calcd. for $C_{26}H_{31}ClN_5O_4^+$ ([*M*+H]$^+$) 512.2059, measured 512.2051.

5-(5-Chloro-2-hydroxyphenyl)-*N*-(3-(piperazin-1-yl)phenyl)-1*H*-pyrazole-3-carboxamide (**5A**):

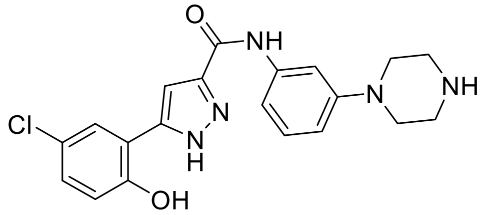

Compound **5A** was prepared following GP-2, *N*-Boc amine **5B** (254 mg, 0.51 mmol) was reacted with TFA (8.1 mmol) and the reaction mixture stirred for 24 h. Compound **5A** was obtained as white powder in form of a TFA salt in quantitative yield (258 mg, 0.51 mmol).
^{1}H NMR (400 MHz, DMSO-d_6) δ = 10.68 (br s, 1H), 10.03 (br s, 1H), 9.00 (br s, 2H), 7.81 (br s, 1H), 7.51 (br s, 1H), 7.39 (br d, J = 7.8 Hz, 1H), 7.44 (br s, 1H), 7.23 (br t, J = 8.6 Hz, 2H), 7.03 (br d, J = 8.6 Hz, 1H), 6.75 (br d, J = 7.8 Hz, 1H), 3.35 (br s, 4H), 3.27 (br s, 4H) ppm.
^{13}C NMR (101 MHz, DMSO-d_6) δ = 158.6, 153.4, 150.4, 139.6, 129.2, 128.8, 126.5, 122.9, 118.1, 112.1, 111.8, 108.0, 105.8, 45.7, 42.7 ppm. Quaternary carbon peaks missing.
HRMS (ESI$^+$): *m*/*z* calcd. for $C_{20}H_{21}ClN_5O_2^+$ ([*M*+H]$^+$) 398.1378, measured 398.1366.

tert-Butyl 4-(3-(5-(5-chloro-2-hydroxyphenyl)-1*H*-pyrazole-3-carboxamido)phenyl)piperazine-1-carboxylate (**5B**):

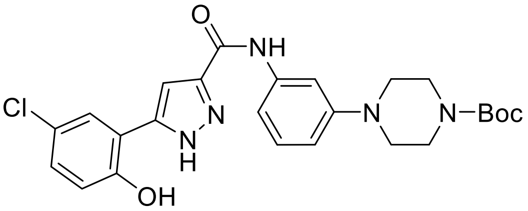

Compound **5B** was prepared following GP-1, chromene amide **42** (224 mg, 0.46 mmol) was reacted with hydrazine hydrate (185 mg, 3.71 mmol). The solvent was removed under reduced pressure to obtain **5B** as off-white powder (228 mg, 0.45 mmol, 99%).

^{1}H NMR (400 MHz, DMSO-d_6) δ = 9.93 (br s, 1H), 7.80 (d, J = 2.0 Hz, 1H), 7.44 (br s, 1H), 7.34 (br d, J = 7.8 Hz, 1H), 7.24 (dd, J = 8.6, 2.0 Hz, 1H), 7.19 (br t, J = 7.8 Hz, 1H), 7.01 (d, J = 8.6 Hz, 1H), 6.71 (br d, J = 7.8 Hz, 1H), 3.47 (br s, 4H), 3.10 (br s, 4H), 1.42 (s, 9H) ppm. Pyrazole C–H is not a clear peak, likely very broad at 7.4 ppm due to annular tautomerism.

^{13}C NMR (101 MHz, DMSO-d_6) δ = 181.3, 153.9, 153.3, 151.2, 139.4, 129.1, 128.8, 126.5, 122.9, 122.1, 118.1, 111.6, 111.5, 107.9, 79.0, 48.4, 43.6, 28.1 ppm. Quaternary carbons missing.

HRMS (ESI$^-$): *m*/*z* calcd. for $C_{25}H_{27}ClN_5O_4^-$ ([*M*-H]$^-$) 496.1757, measured 496.1745.

N-(3-(4-Carbamimidoylpiperazin-1-yl)phenyl)-5-(5-chloro-2-hydroxyphenyl)-1*H*-pyrazole-3-carboxamide (**5G**):

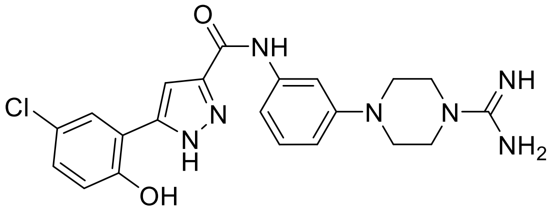

Compound **5G** was prepared following GP-3, amine **5A** (97 mg, 0.19 mmol) was reacted with DIPEA (156 mg, 1.2 mmol) and guanidinylation agent **65** (45 mg, 0.31 mmol). The reaction mixture stirred for 24 h and **5G** was obtained as grey solid in form of a TFA salt in quantitative yield (106 mg, 0.19 mmol).

^{1}H NMR (400 MHz, DMSO-d_6, 120 °C): δ = 7.46 (br s, 1H), 7.43 (br s, 1H), 7.26 (br d, J = 7.6 Hz, 1H), 7.17 (br t, J = 7.6 Hz, 1H), 6.99 (br s, 1H), 6.89 (br s, 1H), 6.72 (br d, J = 8.2 Hz, 1H), 6.64 (br d, J = 7.4 Hz, 1H), 3.63 (br s, 4H), 3.31 (br s, 4H) ppm.

^{13}C NMR (101 MHz, DMSO-d_6, 120 °C): δ = 161.3, 157.1, 156.2, 150.1, 150.0, 145.5, 139.8, 128.4, 125.1, 123.5, 119.9, 119.4, 117.5, 110.3, 109.6, 106.3, 99.1, 46.9, 44.3 ppm.

HRMS (ESI+): *m*/*z* calcd. for $C_{21}H_{23}ClN_7O_2^+$ ([*M*+H]$^+$) 440.1596, measured 440.1587.

5-(5-Chloro-2-hydroxyphenyl)-*N*-(4-(piperazin-1-yl)phenyl)-1*H*-pyrazole-3-carboxamide (**6A**):

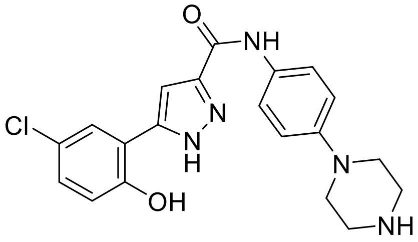

Compound **6A** was prepared following GP-2, *N*-Boc amine **6B** (150 mg, 0.30 mmol) was reacted with TFA (9.6 mmol) and the reaction mixture stirred for 2.5 h. Compound **6A** was obtained as off-white solid in form of a TFA salt in quantitative yield (153 mg, 0.30 mmol).

^{1}H NMR (400 MHz, DMSO-d_6, 120 °C): δ = 9.64 (br s, 1H), 7.73 (d, J = 2.2 Hz, 1H), 7.67 (d, J = 8.6 Hz, 2H), 7.37 (s, 1H), 7.21 (br dd, J = 8.8, 2.2 Hz, 1H), 7.02 (d, J = 8.8 Hz, 1H), 6.99 (d, J = 8.6 Hz, 2H), 3.38 (s, 4H), 3.27 (s, 4H) ppm.

^{13}C NMR (101 MHz, DMSO-d_6, 120 °C): δ = 158.1, 153.0, 145.9, 131.1, 128.1, 127.9, 126.1, 125.9, 122.7, 121.2, 121.0, 118.1, 117.8, 116.0, 115.9, 104.5, 104.3, 45.6, 42.4 ppm.

HRMS (ESI$^+$): *m*/*z* calcd. for $C_{20}H_{21}ClN_5O_2^+$ ([*M*+H]$^+$) 398.1378, measured 398.1366.

tert-Butyl 4-(4-(5-(5-chloro-2-hydroxyphenyl)-1*H*-pyrazole-3-carboxamido)phenyl)piperazine-1-carboxylate (**6B**):

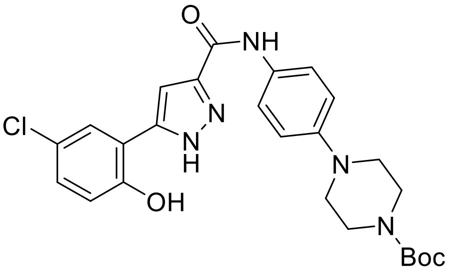

Compound **6B** was prepared following GP-1, chromene amide **43** (212 mg, 0.44 mmol) was reacted with hydrazine hydrate (175 mg, 3.5 mmol). The mixture stirred for 2 h and the solvent was removed under reduced pressure to obtain **6B** as white solid in quantitative yield (220 mg, 0.44 mmol).

^{1}H NMR (400 MHz, DMSO-d_6) δ = 7.80 (d, J = 2.7 Hz, 1H), 7.66 (br d, J = 8.4 Hz, 2H), 7.24 (br dd, J = 8.6, 2.0 Hz, 1H), 7.00 (d, J = 8.6 Hz, 1H), 0.01 (d, J = 8.4 Hz, 2H), 3.46 (t, J = 4.5 Hz, 4H), 3.06 (br t,

J = 4.5 Hz, 4H), 1.42 (s, 9H) ppm. Pyrazole C–H is not a clear peak, likely very broad between peaks 7.66 and 7.24 ppm due to annular tautomerism.
^{13}C NMR (101 MHz, DMSO-d_6) δ = 153.9, 153.4, 147.4, 131.0, 128.8, 126.4, 122.9, 122.1, 121.4, 118.1, 116.3, 79.0, 48.9, 48.4, 28.1 ppm. Quaternary carbon and pyrazole C–H peaks missing.
HRMS (ESI$^+$): *m/z* calcd. for $C_{25}H_{29}ClN_5O_4^+$ ([*M*+H]$^+$) 498.1903, measured 498.1890.

N-(4-(4-Carbamimidoylpiperazin-1-yl)phenyl)-5-(5-chloro-2-hydroxyphenyl)-1*H*-pyrazole-3-carboxamide (**6G**):

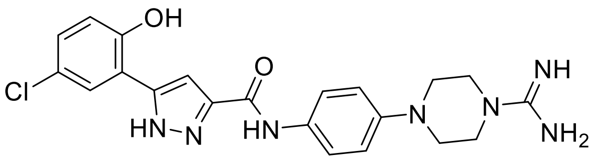

Compound **6G** was prepared following GP-3 with adaptations. Amine **6A** (102 mg, 0.20 mmol) was reacted with DIPEA (216 mg, 1.7 mmol) and guanidinylation agent **65** (45 mg, 0.31 mmol). The reaction mixture stirred for 20 h and the crude (156 mg) was triturated with MeOH (2 mL). Compound **6G** was obtained as grey solid in form of a TFA salt (38 mg, 0.069 mmol, 34%).
^{1}H NMR (400 MHz, DMSO-d_6, 120 °C): δ = 9.68 (br s, 1H), 7.73 (br s, 1H), 7.66 (d, J = 8.2 Hz, 2H), 7.42 (br s, 2H), 7.38 (s, 1H), 7.21 (d, J = 8.6 Hz, 1H), 7.04 (d, J = 8.6 Hz, 1H), 6.97 (d, J = 8.2 Hz, 2H), 3.64 (br s, 4H), 3.26 (br s, 4H) ppm.
^{13}C NMR (101 MHz, DMSO-d_6, 120 °C): δ = 161.5, 158.7, 156.3, 153.0, 146.2, 130.6, 128.0, 125.9, 122.6, 121.1, 117.7, 115.5, 108.9, 104.4, 47.4, 44.4 ppm.
HRMS (ESI$^+$): *m/z* calcd. for $C_{21}H_{23}ClN_7O_2^+$ ([*M*+H]$^+$) 440.1596, measured 440.1585.

N-(3-(Aminomethyl)phenyl)-5-(5-chloro-2-hydroxyphenyl)-1H-pyrazole-3-carboxamide (**7A**):

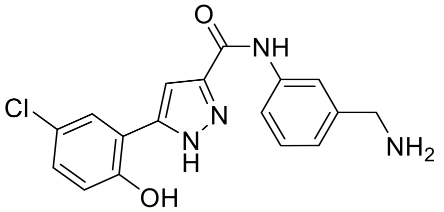

Compound **7A** was prepared following GP-2, *N*-Boc amine **7B** (161 mg, 0.36 mmol) was reacted with TFA (5.8 mmol) and the reaction mixture stirred for 1 h. Compound **7A** was obtained as off-white powder in form of a TFA salt in quantitative yield (165 mg, 0.36 mmol).
^{1}H NMR (400 MHz, DMSO-d_6) δ = 10.65 (br s, 1H), 10.23 (br s, 1H), 8.24 (br s, 2H), 8.01 (br s, 1H), 7.81 (d, J = 2.3 Hz, 1H), 7.74 (br d, J = 8.0 Hz, 1H), 7.42 (br t, J = 8.0 Hz, 1H), 7.25 (dd, J = 8.6, 2.3 Hz, 1H), 7.21 (br d, J = 8.0 Hz, 1H), 7.03 (d, J = 8.6 Hz, 1H), 4.04 (br d, J = 5.1 Hz, 2H) ppm.
^{13}C NMR (101 MHz, DMSO-d_6) δ = 158.4, 158.1, 157.8, 153.5, 139.2, 134.6, 129.2, 129.0, 126.7, 124.1, 123.1, 120.9, 120.7, 118.3, 106.1, 42.7 ppm. Quaternary carbon peaks missing.
HRMS (ESI$^-$): *m/z* calcd. for $C_{17}H_{14}ClN_4O_2^-$ ([*M*-H]$^-$) 341.08108, measured 341.0798.

tert-Butyl (3-(5-(5-chloro-2-hydroxyphenyl)-1*H*-pyrazole-3-carboxamido)benzyl)carbamate (**7B**):

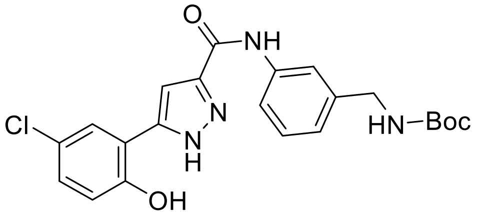

Compound **7B** was prepared following GP-1, chromene amide **44** (400 mg, 0.93 mmol) was reacted with hydrazine hydrate (374 mg, 7.46 mmol). The solvent was removed under reduced pressure to obtain compound **7B** as white solid in quantitative yield (440 mg, 0.93 mmol).
^{1}H NMR (400 MHz, DMSO-d_6) δ = 10.13 (br s, 1H), 7.81 (br s, 1H), 7.64-7.75 (m, 2H), 7.48 (br s, 1H), 7.34-7.41 (m, 1H), 7.21-7.32 (m, 2H), 6.89-7.09 (m, 2H), 4.13 (br s, 2H), 1.40 (br s, 9H) ppm.
^{13}C NMR (101 MHz, DMSO-d_6) δ = 155.6, 153.2, 140.6, 138.4, 128.6, 128.3, 126.3, 122.8, 122.1, 118.7, 118.6, 117.9, 105.6, 77.6, 43.3, 28.1 ppm. Quaternary carbon peaks missing.
HRMS (ESI$^-$): *m/z* calcd. for $C_{22}H_{22}ClN_4O_4^-$ ([*M*-H]$^-$) 441.1335, measured 441.1327.

5-(5-Chloro-2-hydroxyphenyl)-*N*-(3-(((4,5-dihydro-1H-imidazol-2-yl)amino)methyl)phenyl)-1*H*-pyrazole-3-carboxamide (**7C**):

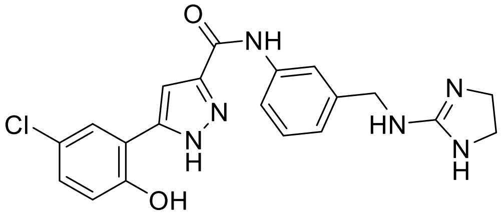

Compound **7C** was prepared following GP-2, *N*-Boc guanidine **7D** (163 mg, 0.32 mmol) was reacted with TFA (5.1 mmol) and the reaction mixture stirred overnight. Compound **7C** was obtained as off-white powder in form of a TFA salt in quantitative yield (169 mg, 0.32 mmol).

^{1}H NMR (400 MHz, DMSO-*d*$_6$) δ = 10.64 (br s, 1H), 10.10 (br s, 1H), 8.74 (br s, 1H), 7.89 (br s, 1H), 7.80 (s, 1H), 7.69 (d, *J* = 7.6 Hz, 1H), 7.35 (t, *J* = 7.6 Hz, 1H), 7.26 (d, *J* = 7.6 Hz, 2H), 7.03 (br s, 1H), 7.01 (s, 1H), 4.39 (br d, *J* = 5.5 Hz, 2H), 3.63 (s, 4H) ppm.

^{13}C NMR (101 MHz, DMSO-*d*$_6$) δ = 160.5, 159.6, 158.5, 158.2, 153.3, 139.0, 137.6, 128.9, 126.5, 122.9, 122.3, 119.5, 118.9, 118.7, 118.1, 115.7, 105.9, 45.5, 42.6 ppm.

HRMS (ESI+): *m*/*z* calcd. for $C_{20}H_{20}ClN_6O_2^+$ ([*M*+H]$^+$) 411.1331, measured 411.1308.

tert-Butyl 2-((3-(5-(5-chloro-2-hydroxyphenyl)-1*H*-pyrazole-3-carboxamido)benzyl)amino)-4,5-dihydro-1*H*-imidazole-1-carboxylate (**7D**):

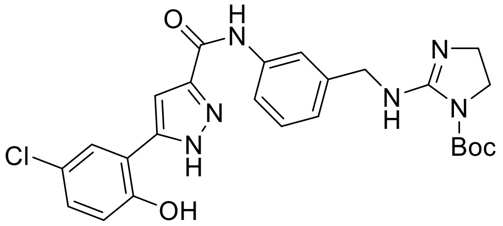

Compound **7D** was prepared following GP-3, amine **3A** (200 mg, 0.44 mmol) was reacted with DIPEA (80 mg, 0.66 mmol) and guanidinylation agent **64** (284 mg, 1.31 mmol). The reaction mixture stirred for 24 h, and compound **7D** was obtained as off-white solid (182 mg, 0.36 mmol, 82%). Product contains 5% Boc-deprotected compound **7C**.

^{1}H NMR (400 MHz, DMSO-*d*$_6$) δ = 10.12 (br s, 1H), 7.79 (br d, *J* = 1.6 Hz, 1H), 7.69-7.76 (m, 2H), 7.43 (br s, 1H), 7.30 (br t, *J* = 7.8 Hz, 1H), 7.23 (br s, 1H), 7.05 (br d, *J* = 8.6 Hz, 1H), 6.99 (br d, *J* = 8.6 Hz, 1H), 4.36 (s, 2H), 3.69 (t, *J* = 8.0 Hz, 2H), 3.48 (t, *J* = 8.0 Hz, 2H), 1.46 (s, 9H) ppm.

^{13}C NMR (101 MHz, DMSO-*d*$_6$) δ = 158.9, 153.1, 152.1, 151.5, 139.8, 138.3, 128.2, 128.1, 125.9, 122.3, 122.2, 118.7, 118.4, 118.0, 117.7, 105.1, 81.1, 47.7, 46.0, 45.3, 27.4 ppm.

HRMS (ESI+): *m*/*z* calcd. for $C_{25}H_{28}ClN_6O_4^+$ ([*M*+H]$^+$) 511.1855, measured 511.1838.

5-(5-Chloro-2-hydroxyphenyl)-*N*-(3-(guanidinomethyl)phenyl)-1*H*-pyrazole-3-carboxamide (**7G**):

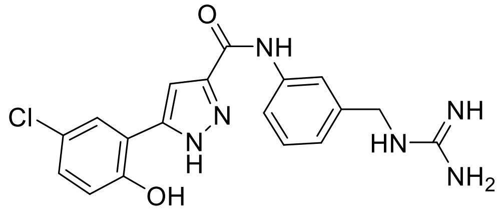

Compound **7G** was prepared following GP-3, amine **7A** (108 mg, 0.24 mmol) was reacted with DIPEA (183 mg, 1.4 mmol) and guanidinylation agent **65** (52 mg, 0.36 mmol). The reaction mixture stirred for 5 h, and **7G** was obtained as white solid in form of a TFA salt (74 mg, 0.23 mmol, 63%).

^{1}H NMR (400 MHz, DMSO-*d*$_6$) δ = 9.92 (br s, 1H), 7.92 (br s, 1H), 7.70 (br d, *J* = 7.0 Hz, 1H), 7.49 (br s, 1H), 7.31 (t, *J* = 7.0 Hz, 1H), 7.15 (br s, 1H), 6.96 (br s, 2H), 6.71 (br d, *J* = 7.0 Hz, 1H), 4.37 (br s, 2H) ppm.

^{13}C NMR (101 MHz, DMSO-*d*$_6$) δ = 162.1, 158.0, 156.9, 149.3, 146.0, 139.9, 137.5, 128.8, 125.9, 124.1, 121.2, 120.2, 119.5, 118.5, 118.4, 118.0, 100.0, 44.3 ppm.

HRMS (ESI$^+$): *m/z* calcd. for $C_{18}H_{18}ClN_6O_2^+$ ([*M*+H]$^+$) 385.1174, measured 385.1166.

N-(4-(Aminomethyl)phenyl)-5-(5-chloro-2-hydroxyphenyl)-1*H*-pyrazole-3-carboxamide (**8A**):

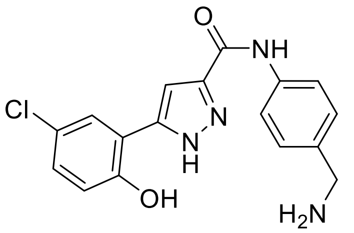

Compound **8A** was prepared following GP-2, *N*-Boc amine **8B** (289 mg, 0.65 mmol) was reacted with TFA (10.4 mmol) and the reaction mixture stirred overnight. Compound **8A** was obtained as off-white solid in form of a TFA salt in quantitative yield (299 mg, 0.65 mmol).

^{1}H NMR (400 MHz, DMSO-*d*$_6$) δ = 10.24 (br s, 1H), 7.85 (br d, *J* = 8.2 Hz, 2H), 7.81 (br d, *J* = 2.7 Hz, 1H), 7.42 (br d, *J* = 8.2 Hz, 2H), 7.34 (br s, 1H), 7.25 (br dd, *J* = 8.6, 2.7 Hz, 1H), 7.02 (d, *J* = 8.6 Hz, 1H), 4.00 (s, 2H) ppm.

^{13}C NMR (101 MHz, DMSO-*d*$_6$, 120 °C): δ = 158.7, 153.0, 138.4, 138.3, 128.6, 128.4, 128.1, 126.1, 122.7, 119.9, 117.9, 117.8, 104.7, 41.8 ppm.

HRMS (ESI$^-$): *m*/*z* calcd. for $C_{17}H_{14}ClN_4O_2^-$ ([*M*-H]$^-$) 341.0811, measured 341.0799.

tert-Butyl(4-(5-(5-chloro-2-hydroxyphenyl)-1*H*-pyrazole-3-carboxamido)benzyl)carbamate (**8B**):

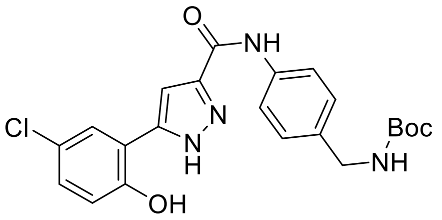

Compound **8B** was prepared following GP-1, chromene amide **45** (280 mg, 0.65 mmol) was reacted with hydrazine hydrate (261 mg, 5.2 mmol). The solvent was removed under reduced pressure obtaining **8B** as white solid in quantitative yield (290 mg, 0.65 mmol).

^{1}H NMR (400 MHz, DMSO-d_6) δ = 10.08 (br s, 1H), 7.80 (d, J = 2.7 Hz, 1H), 7.73 (br d, J = 8.2 Hz, 2H), 7.35 (br s, 1H), 7.24 (dd, J = 8.8, 2.7 Hz, 1H), 7.21 (d, J = 8.2 Hz, 2H), 7.01 (d, J = 8.8 Hz, 1H), 4.09 (d, J = 5.9 Hz, 2H), 1.40 (s, 9H) ppm.

^{13}C NMR (101 MHz, DMSO-d_6) δ = 155.8, 153.3, 137.2, 135.4, 128.7, 127.2, 126.4, 122.9, 120.2, 118.1, 105.7, 77.7, 43.0, 28.3 ppm. Quaternary carbon peaks missing.

HRMS (ESI$^-$): *m/z* calcd. for $C_{22}H_{22}ClN_4O_4^-$ ([*M*-H]$^-$) 441.1335, measured 441.13266.

5-(5-Chloro-2-hydroxyphenyl)-*N*-(4-(((4,5-dihydro-1*H*-imidazol-2-yl)amino)methyl)phenyl)-1*H*-pyrazole-3-carboxamide(**8C**):

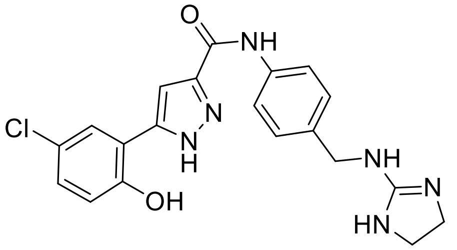

Compound **8C** was prepared following GP-2, *N*-Boc guanidine **8D** (21 mg, 0.042 mmol) was reacted with TFA (1.4 mmol) and the reaction mixture stirred for 24 h. Compound **8C** was obtained as light brown powder in form of a TFA salt (21 mg, 0.040 mmol, 96%).

^{1}H NMR (400 MHz, DMSO-d_6) δ = 13.50 (s, 2H), 10.64 (br s, 1H), 10.15 (br s, 1H), 8.80 (br s, 1H), 8.46 (br s, 1H), 8.45 (br s, 1H), 7.83 (br s, 2H), 7.18-7.38 (m, 3H), 7.03 (br s, 1H), 4.35 (s, 2H), 3.62 (s, 2H), 3.11 (br s, 2H) ppm.

^{13}C NMR (101 MHz, DMSO-d_6) δ = 159.9, 158.6, 158.3, 153.7, 138.6, 132.6, 129.2, 128.1, 126.9, 123.3, 120.8, 118.5, 106.3, 54.0, 46.1, 45.5, 43.0 ppm.

HRMS (ESI$^+$): *m/z* calcd. for $C_{20}H_{20}ClN_6O_2^+$ ([*M*+H]$^+$) 411.1331, measured 411.1317.

tert-Butyl 2-((4-(5-(5-chloro-2-hydroxyphenyl)-1*H*-pyrazole-3-carboxamido)benzyl)amino)-4,5-dihydro-1*H*-imidazole-1-carboxylate (**8D**):

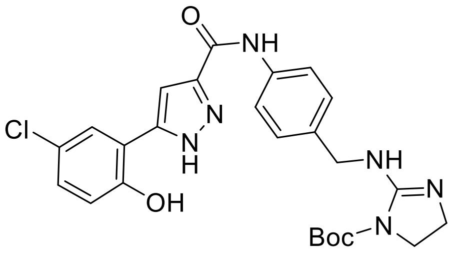

Compound **8D** was prepared following GP-4 with adaptations. Amine **8A** (74 mg, 0.17 mmol) was reacted with DIPEA (129 mg, 1.0 mmol) and guanidinylation agent **64** (107 mg, 0.49 mmol), the reaction mixture stirred for 150 h. It was quenched with water (10 mL) and extracted with EtOAc (3 x 10 mL). The combined organic layers were washed with brine, dried over anhydrous $MgSO_4$, filtered, and concentrated *in vacuo*, obtaining of a viscous brown oil (129 mg). The crude was purified by flash column chromatography using 10% MeOH in DCM as eluent to obtain **8D** as a white solid (41 mg, 0.080 mmol, 47%).

^{1}H NMR (400 MHz, DMSO-d_6) δ = 10.25 (br s, 1H), 10.20 (br s, 1H), 8.79 (br s, 1H), 7.95 (br s, 1H), 7.76-7.84 (m, 3H), 7.43 (br s, 1H), 7.28-7.35 (m, 2H), 7.24 (dd, J = 8.8, 2.3 Hz, 1H), 7.05 (dd, J = 8.8, 2.9 Hz, 1H), 3.80 (t, J = 8.4 Hz, 2H), 3.61 (s, 2H), 3.54 (t, J = 8.4 Hz, 2H), 1.48 (s, 9H) ppm.

^{13}C NMR (101 MHz, DMSO-d_6) δ = 162.3, 159.4, 153.4, 151.5, 138.2, 137.8, 133.3, 132.1, 128.7, 127.8, 127.7, 126.4, 122.8, 120.3, 120.3, 118.1, 105.8, 82.8, 45.4, 45.1, 42.5, 27.7 ppm.

HRMS (ESI$^+$): *m/z* calcd. for $C_{25}H_{28}ClN_6O_4^+$ ([*M*+H]$^+$) 511.1855, measured 511.1829.

5-(5-Chloro-2-hydroxyphenyl)-*N*-(4-(guanidinomethyl)phenyl)-1*H*-pyrazole-3-carboxamide (**8G**):

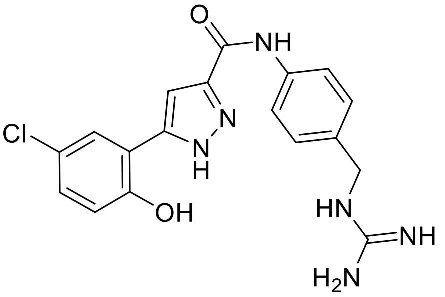

Compound **8G** was prepared following GP-3, amine **8A** (65 mg, 0.15 mmol) was reacted with DIPEA (124 mg, 0.96 mmol) and guanidinylation agent **65** (37 mg, 0.25 mmol). The reaction mixture stirred for 3.5 h and **8G** was obtained as off-white solid in form of a TFA salt (53 mg, 0.11 mmol, 74%).

^{1}H NMR (500 MHz, DMSO-d_6) δ = 10.21 (br s, 1H), 8.24 (br s, 1H), 7.78 (br d, *J* = 8.2 Hz, 2H), 7.75 (br s, 1H), 7.39 (br s, 1H), 7.26 (br d, *J* = 8.1 Hz, 2H), 7.18 (br d, *J* = 8.1 Hz, 1H), 7.00 (d, *J* = 8.1 Hz, 1H), 4.30 (br d, *J* = 4.7 Hz, 2H) ppm.

^{13}C NMR (101 MHz, DMSO-d_6) δ = 158.5, 156.9, 153.4, 138.1, 132.2, 128.8, 127.6, 126.4, 122.9, 120.4, 118.6, 118.1, 115.6, 112.7, 105.8, 43.7 ppm.

HRMS (ESI$^+$): *m/z* calcd. for $C_{18}H_{18}ClN_6O_2^+$ ([*M*+H]$^+$) 385.1174, measured 385.1176.

N-(3-(Aminomethyl)-5-(trifluoromethyl)phenyl)-5-(5-chloro-2-hydroxyphenyl)-1*H*-pyrazole-3-carboxamide (**9A**):

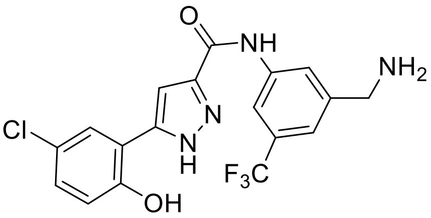

Compound **9A** was prepared following GP-2, *N*-Boc amine **9B** (138 mg, 0.27 mmol) was reacted with TFA (6.7 mmol) and the reaction mixture stirred for 24 h. Compound **9A** was obtained as off-white solid in form of a TFA salt in quantitative yield (141 mg, 0.27 mmol).

^{1}H NMR (400 MHz, DMSO-d_6) δ = 10.68 (br s, 1H), 10.53 (br s, 1H), 8.24-8.40 (m, 3H), 8.20 (br s, 1H), 7.81 (d, *J* = 2.3 Hz, 1H), 7.60 (s, 1H), 7.30 (br s, 1H), 7.26 (br dd, *J* = 7.4, 2.3 Hz, 2H), 7.04 (d, *J* = 7.4 Hz, 1H), 4.12 (br s, 2H) ppm.

^{13}C NMR (101 MHz, DMSO-d_6) δ = 158.2, 153.7, 140.3, 136.4, 130.2, 129.9, 129.5, 127.0, 125.7, 123.0, 120.6, 118.5, 117.0, 106.6, 42.5 ppm. Quaternary carbon peaks missing.

^{19}F NMR (376 MHz, DMSO-d_6) δ = -61.38, -73.61 ppm.

HRMS (ESI$^+$): *m/z* calcd. for $C_{18}H_{15}ClF_3N_4O_2^+$ ([*M*+H]$^+$) 411.0830, measured 411.0811.

tert-Butyl (3-(5-(5-chloro-2-hydroxyphenyl)-1*H*-pyrazole-3-carboxamido)-5-(trifluoromethyl)benzyl)carbamate (**9B**):

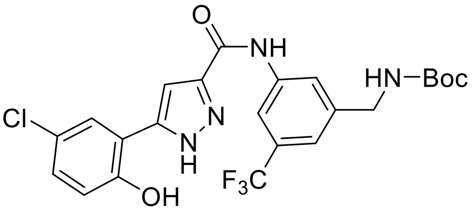

Compound **9B** was prepared following GP-1, chromene amide **46** (137 mg, 0.28 mmol) was reacted with hydrazine hydrate (111 mg, 2.2 mmol). The mixture stirred for 2.5 h and the solvent was removed under reduced pressure to obtain **9B** as yellow solid in quantitative yield (143 mg, 0.28 mmol).

^{1}H NMR (500 MHz, DMSO-d_6) δ = 10.49 (br s, 1H), 8.15 (s, 1H), 8.02 (br s, 1H), 7.81 (d, *J* = 2.4 Hz, 1H), 7.51 (br t, *J* = 6.0 Hz, 1H), 7.30 (s, 1H), 7.25 (br dd, *J* = 8.7, 2.4 Hz, 1H), 7.01 (d, *J* = 8.7 Hz, 1H), 4.20 (d, *J* = 6.0 Hz, 2H), 1.41 (s, 9H) ppm.

^{13}C NMR (126 MHz, DMSO-d_6) δ = 156.3, 153.8, 142.9, 140.1, 129.9, 129.6, 129.3, 127.9, 127.0, 125.7, 123.6, 123.4, 122.6, 118.5, 115.3, 106.6, 78.5, 43.7, 28.7 ppm.

^{19}F NMR (470 MHz, DMSO-d_6) δ = -61.28 ppm.

HRMS (ESI$^-$): *m*/z calcd. for $C_{23}H_{21}ClF_3N_4O_4^-$ ([*M*-H]$^-$) 509.1209, measured 509.1194.

5-(5-Chloro-2-hydroxyphenyl)-*N*-(3-(((4,5-dihydro-1*H*-imidazol-2-yl)amino)methyl)-5-(trifluoromethyl)phenyl)-1*H*-pyrazole-3-carboxamide (**9C**):

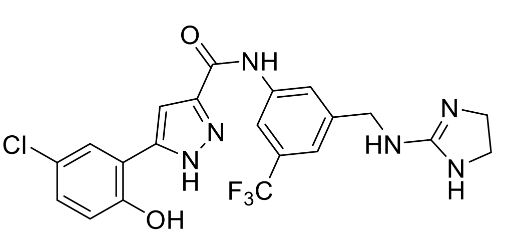

Compound **9C** was prepared following GP-2, *N*-Boc guanidine **9D** (50 mg, 0.086 mmol) was reacted with TFA (1.3 mmol) and the reaction mixture stirred overnight. Compound **9C** was obtained as light grey solid in form of a TFA salt in quantitative yield (51 mg, 0.086 mmol).

^{1}H NMR (400 MHz, acetone-d_6) δ = 10.40 (br s, 1H), 10.00 (br s, 1H), 8.39 (s, 1H), 8.15 (br s, 1H), 7.75 (s, 1H), 7.59 (br s, 1H), 7.45 (s, 1H), 7.21 (d, *J* = 8.0 Hz, 1H), 7.06 (br d, *J* = 8.0 Hz, 1H), 4.65 (br s, 2H), 4.04 (br s, 2H), 3.78 (br s, 4H) ppm.

^{13}C NMR (101 MHz, acetone-d_6) δ = 171.9, 160.9, 154.3, 140.3, 139.7, 131.3, 129.4, 126.8, 125.9, 124.3, 123.2, 122.3, 119.2, 118.6, 118.0, 115.9, 104.3, 45.9, 45.5, 43.1 ppm.
^{19}F NMR (376 MHz, acetone-d_6) δ = -63.12, -75.77 ppm.
HRMS (ESI+): m/z calcd. for $C_{21}H_{19}ClF_3N_6O_2^+$ ([M+H]$^+$) 479.1205, measured 479.1192.

tert-Butyl 2-((3-(5-(5-chloro-2-hydroxyphenyl)-1*H*-pyrazole-3-carboxamido)-5-(trifluoromethyl)benzyl)amino)-4,5-dihydro-1*H*-imidazole-1-carboxylate (**9D**):

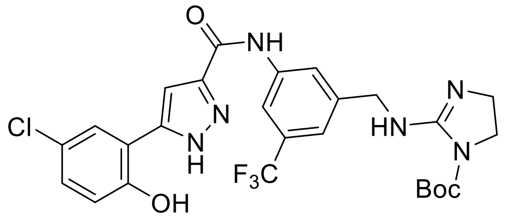

Compound **9D** was prepared following GP-3, amine **9A** (200 mg, 0.44 mmol) was reacted with DIPEA (80 mg, 0.66 mmol) and guanidinylation agent **64** (26 mg, 0.20 mmol). The reaction mixture stirred for 48 h, and compound **9D** was obtained as off-white powder (54 mg, 0.93 mmol, 70%). Product contains 5% Boc-deprotected compound **9C.**

^1H NMR (500 MHz, DMSO-d_6) δ = 10.50 (br s, 1H), 8.19 (s, 1H), 8.04 (s, 1H), 7.81 (d, *J* = 2.3 Hz, 1H), 7.45 (br s, 1H), 7.40 (s, 1H), 7.25 (dd, *J* = 8.7, 2.3 Hz, 1H), 7.02 (d, *J* = 8.7 Hz, 1H), 4.45 (s, 2H), 3.73-3.78 (m, 2H), 3.69-3.73 (m, 2H), 1.48 (s, 9H) ppm.
^{13}C NMR (126 MHz, DMSO-d_6) δ = 158.2, 153.9, 153.1, 152.3, 142.7, 140.0, 129.8, 129.5, 129.3, 126.9, 125.8, 123.6, 123.0, 119.2, 118.6, 115.4, 106.4, 82.1, 47.8, 47.0, 45.7, 28.3 ppm.
^{19}F NMR (470 MHz, DMSO-d_6) δ = -61.14 ppm.
HRMS (ESI+): m/z calcd. for $C_{26}H_{27}ClF_3N_6O_4^+$ ([M+H]$^+$) 579.1729, measured 579.1713.

5-(5-Chloro-2-hydroxyphenyl)-*N*-(3-(guanidinomethyl)-5-(trifluoromethyl)phenyl)-1*H*-pyrazole-3-carboxamide (**9G**):

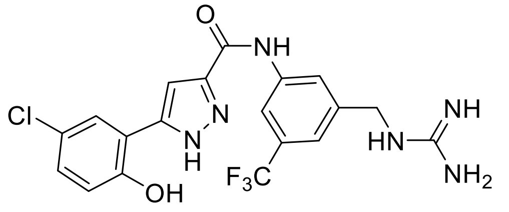

Compound **9G** was prepared following GP-3 with adaptations, amine **9A** (40 mg, 0.076 mmol) was reacted with DIPEA (59 mg, 0.46 mmol) and guanidinylation agent **65** (17 mg, 0.11 mmol). The reaction mixture stirred for 30 h obtaining an off-white solid (54 mg). Part of the crude (15 mg, 28%) was purified by preparative HPLC to afford **9G** as white powder (5 mg, 0.09 mmol, 43%).

^1H NMR (500 MHz, DMSO-d_6) δ = 10.61 (s, 1H), 8.90 (br s, 1H), 8.41 (s, 1H), 8.21 (s, 1H), 8.14 (s, 1H), 7.85 (br s, 2H), 7.78 (d, *J* = 2.6 Hz, 1H), 7.39 (s, 1H), 7.36 (s, 1H), 7.21 (dd, *J* = 8.8, 2.6 Hz, 1H), 7.01 (d, *J* = 8.8 Hz, 1H), 4.46 (d, *J* = 4.1 Hz, 2H) ppm.
^{13}C NMR (126 MHz, DMSO-d_6) δ = 167.0, 160.8, 157.9, 154.5, 140.5, 140.4, 130.0, 129.8, 129.0, 126.7, 125.7, 122.9, 122.5, 118.8, 118.6, 115.8, 106.0, 43.9 ppm.
^{19}F NMR (470 MHz, DMSO-d_6) δ = -61.24 ppm.
HRMS (ESI+): m/z calcd. for $C_{19}H_{17}ClF_3N_6O_2^+$ ([M+H]$^+$) 453.1048, measured 453.1028.

N-(4-(Aminomethyl)-3-(trifluoromethyl)phenyl)-5-(5-chloro-2-hydroxyphenyl)-1*H*-pyrazole-3-carboxamide (**10A**):

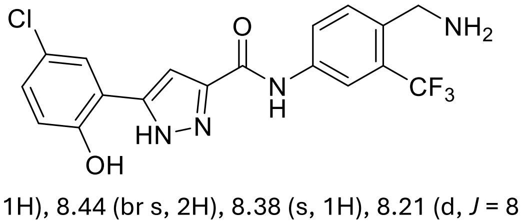

Compound **10A** was prepared following GP-2, *N*-Boc amine **10B** (1910 mg, 3.74 mmol) was reacted with TFA (94.0 mmol) and the reaction mixture stirred for 3 h. Compound **10A** was obtained as light orange solid in form of a TFA salt in quantitative yield (1999 mg, 3.74 mmol).

^1H NMR (400 MHz, DMSO-d_6) δ = 10.69 (br s, 1H), 10.63 (br s, 1H), 8.44 (br s, 2H), 8.38 (s, 1H), 8.21 (d, *J* = 8.2 Hz, 1H), 7.82 (br s, 1H), 7.69 (d, *J* = 8.2 Hz, 1H), 7.41 (br s, 1H), 7.25 (d, *J* = 8.6 Hz, 1H), 7.04 (d, *J* = 8.6 Hz, 1H), 4.16 (s, 2H) ppm.
^{13}C NMR (101 MHz, DMSO-d_6) δ = 160.2, 158.7, 153.4, 139.6, 131.5, 128.9, 127.7, 126.6, 126.2, 125.3, 123.6, 122.9, 122.6, 118.2, 117.4, 115.4, 106.3, 38.5 ppm
^{19}F NMR (376 MHz, DMSO-d_6) δ = -58.24, -73.93 ppm.
HRMS (ESI$^-$): *m*/*z* calcd. for $C_{18}H_{13}ClF_3N_4O_2^-$ ([*M*-H]$^-$) 409.0685, measured 409.0674.

tert-Butyl (4-(5-(5-chloro-2-hydroxyphenyl)-1*H*-pyrazole-3-carboxamido)-2-(trifluoromethyl)benzyl)carbamate (**10B**):

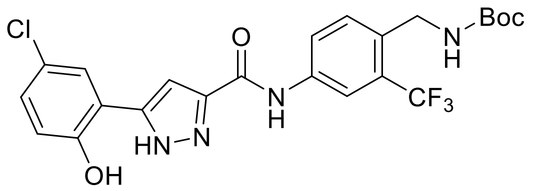

Compound **10B** was prepared following GP-1, chromene amide **47** (2000 mg, 4.02 mmol) was reacted with hydrazine hydrate (1621 mg, 32.4 mmol). The mixture stirred for 3 h and the solvent was removed under reduced pressure obtaining **10B** as white solid (1972 mg, 3.86 mmol, 96%).

^{1}H NMR (400 MHz, DMSO-d_6) δ = 10.46 (s, 1H), 8.27 (s, 1H), 8.09 (d, *J* = 8.2 Hz, 1H), 7.81 (br d, *J* = 2.2 Hz, 1H), 7.48 (br s, 1H), 7.46 (br s, 1H), 7.43 (br s, 1H), 7.24 (dd, *J* = 8.6, 2.2 Hz, 1H), 7.02 (d, *J* = 8.6 Hz, 1H), 4.30 (d, *J* = 3.9 Hz, 2H), 1.41 (s, 9H) ppm.

^{13}C NMR (101 MHz, DMSO-d_6) δ = 160.3, 156.2, 153.8, 144.5, 142.7, 138.2, 133.2, 129.2, 126.9, 126.7, 126.4, 126.1, 124.1, 123.4, 123.3, 120.6, 118.5, 117.7, 106.4, 78.6, 28.6 ppm.

^{19}F NMR (376 MHz, DMSO-d_6) δ = -59.19 ppm.

HRMS (ESI-): *m/z* calcd. for $C_{23}H_{21}ClF_3N_4O_4^-$ ([*M*-H]$^-$) 509.1209, measured 509.1189.

5-(5-Chloro-2-hydroxyphenyl)-*N*-(4-(((4,5-dihydro-1*H*-imidazol-2-yl)amino)methyl)-3-(trifluoromethyl)phenyl)-1*H*-pyrazole-3-carboxamide (**10C**):

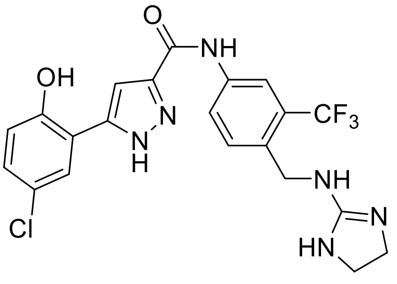

Compound **10C** was prepared following GP-2, *N*-Boc guanidine **10D** (122 mg, 0.21 mmol) was reacted with TFA (3.4 mmol) and the reaction mixture stirred for 48 h. Compound **10C** was obtained as brown solid in form of a TFA salt in quantitative yield (125 mg, 0.21 mmol).

^{1}H NMR (400 MHz, DMSO-d_6) δ = 10.59 (s, 1H), 8.78 (t, *J* = 5.8 Hz, 1H), 8.36 (d, *J* = 1.8 Hz, 1H), 8.17 (dd, *J* = 8.4, 1.8 Hz, 1H), 7.82 (d, *J* = 2.3 Hz, 1H), 7.53 (d, *J* = 8.4 Hz, 2H), 7.43 (br s, 1H), 7.26 (dd, *J* = 8.6, 2.3 Hz, 1H), 7.04 (d, *J* = 8.6 Hz, 1H), 4.53 (br d, *J* = 5.8 Hz, 2H), 3.66 (s, 4H) ppm.

^{13}C NMR (101 MHz, DMSO-d_6) δ = 159.6, 158.6, 158.3, 153.3, 138.9, 130.1, 129.0, 128.9, 127.0, 126.5, 125.5, 123.7, 122.9, 122.7, 118.1, 117.7, 106.2, 42.6, 42.5 ppm.

^{19}F NMR (376 MHz, DMSO-d_6) δ = -58.85, -74.55 ppm.

HRMS (ESI$^+$): *m/z* calcd. for $C_{21}H_{19}ClF_3N_6O_2^+$ ([*M*+H]$^+$) 479.1205, measured 479.1186.

tert-Butyl 2-((4-(5-(5-chloro-2-hydroxyphenyl)-1*H*-pyrazole-3-carboxamido)-2-(trifluoromethyl)benzyl)amino)-4,5-dihydro-1*H*-imidazole-1-carboxylate (**10D**):

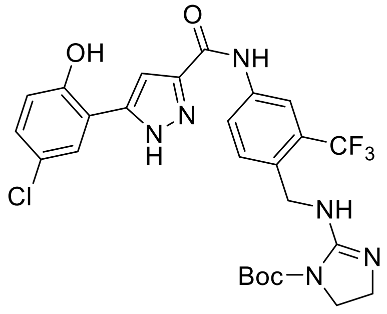

Compound **10D** was prepared following GP-3 with adaptations. Amine **10A** (314 mg, 0.62 mmol) was reacted with DIPEA (471 mg, 3.6 mmol) and guanidinylation agent **64** (392 mg, 1.81 mmol). The reaction mixture stirred for 6 days. The obtained precipitate was filtered and purified by column chromatography using 10% MeOH in DCM as eluent to obtain **10D** as a white powder (186 mg, 0.32 mmol, 52%).

^{1}H NMR (400 MHz, DMSO-d_6) δ = 10.50 (br s, 1H), 8.28 (br s, 1H), 8.07 (br d, *J* = 8.2 Hz, 1H), 7.81 (br d, *J* = 2.4 Hz, 1H), 7.55 (br d, *J* = 8.2 Hz, 1H), 7.42 (br s, 1H), 7.25 (br dd, *J* = 8.6, 2.3 Hz, 1H), 7.02 (br d, *J* = 8.6 Hz, 1H), 4.54 (br s, 1H), 3.72 (br t, *J* = 8.2 Hz, 2H), 3.47 (t, *J* = 8.2 Hz, 2H), 1.47 (s, 9H) ppm.

^{13}C NMR (101 MHz, DMSO-d_6) δ = 153.4, 152.6, 151.9, 141.7, 141.6, 138.0, 132.2, 129.7, 128.8, 126.5, 126.2, 125.7, 123.7, 123.0, 122.9, 118.1, 117.4, 106.1, 81.8, 47.7, 46.5, 42.2, 27.8 ppm.

^{19}F NMR (376 MHz, DMSO-d_6) δ = -59.05 ppm.

HRMS (ESI$^+$): *m/z* calcd. for $C_{26}H_{27}ClF_3N_6O_4^+$ ([*M*+H]$^+$) 579.1729, measured 579.1710.

5-(5-Chloro-2-hydroxyphenyl)-*N*-(4-(guanidinomethyl)-3-(trifluoromethyl)phenyl)-1*H*-pyrazole-3-carboxamide (**10G**):

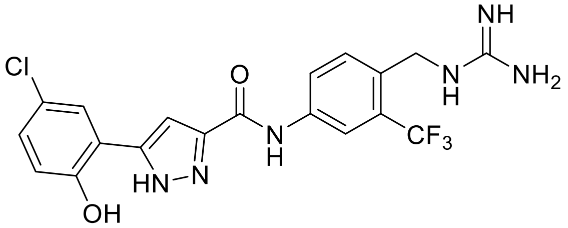

Compound **10G** was prepared following GP-3, amine **10A** (260 mg, 0.51 mmol) was reacted with DIPEA (402 mg, 3.1 mmol) and guanidinylation agent **65** (114 mg, 0.78 mmol). The reaction mixture stirred for 3.5 h and **10G** was obtained as light yellow solid in form of a TFA salt (190 mg, 0.37 mmol, 75%).

^{1}H NMR (400 MHz, DMSO-*d*$_6$) δ = 10.62 (br s, 1H), 8.37 (s, 1H), 8.17 (d, *J* = 8.2 Hz, 1H), 8.02 (br s, 1H), 7.82 (s, 1H), 7.53 (br d, *J* = 8.2 Hz, 1H), 7.44 (br s, 2H), 7.25 (br d, *J* = 8.2 Hz, 1H), 7.06 (br d, *J* = 8.2 Hz, 1H), 4.51 (br s, 2H) ppm.

^{13}C NMR (101 MHz, DMSO-*d*$_6$) δ = 158.6, 157.1, 153.4, 138.9, 130.2, 129.2, 128.9, 128.2, 127.1, 126.5, 125.5, 123.7, 122.9, 118.6, 118.1, 117.7, 115.6, 106.2, 41.3 ppm.

^{19}F NMR (376 MHz, DMSO-*d*$_6$) δ = -58.65, -73.55 ppm.

HRMS (ESI$^+$): *m/z* calcd. for $C_{19}H_{17}ClF_3N_6O_2^+$ ([*M*+H]$^+$) 453.1048, measured 453.1028.

5-(5-Chloro-2-hydroxyphenyl)-*N*-(3-(piperazin-1-yl)-5-(trifluoromethyl)phenyl)-1*H*-pyrazole-3-carboxamide (**11A**):

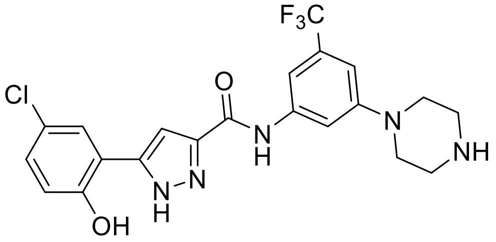

Compound **11A** was prepared following GP-2 with adaptations, *N*-Boc amine **11B** (500 mg, 0.88 mmol) was reacted with TFA (27 mmol) and the reaction mixture stirred overnight. A precipitate appeared which was filtered and washed with ice-cold EtOH (2 mL) to obtain **11A** as white powder in form of a TFA salt (393 mg, 0.70 mmol, 79%).

^{1}H NMR (400 MHz, DMSO-*d*$_6$, 120 °C): δ = 10.01 (br s, 1H), 7.73-7.77 (m, 2H), 7.72 (br s, 1H), 7.39 (s, 1H), 7.22 (br d, *J* = 8.6 Hz, 1H), 7.04 (br d, *J* = 8.6 Hz, 1H), 6.99 (br s, 1H), 3.46-3.55 (m, 4H), 3.26-3.35 (m, 4H) ppm.

^{13}C NMR (101 MHz, DMSO-*d*$_6$, 120 °C): δ = 159.0, 152.9, 150.2, 142.9, 139.9, 130.2, 129.9, 128.1, 126.1, 125.0, 122.7, 117.8, 117.7, 110.1, 107.5, 106.8, 104.9, 44.8, 42.2 ppm.

^{19}F NMR (376 MHz, DMSO-*d*$_6$) δ = -61.27, -73.55 ppm.

HRMS (ESI$^+$): *m/z* calcd. for $C_{21}H_{20}ClF_3N_5O_2^+$ ([*M*+H]$^+$) 466.1252, measured 466.1229.

tert-Butyl 4-(3-(5-(5-chloro-2-hydroxyphenyl)-1*H*-pyrazole-3-carboxamido)-5-(trifluoromethyl)phenyl)piperazine-1-carboxylate (**11B**):

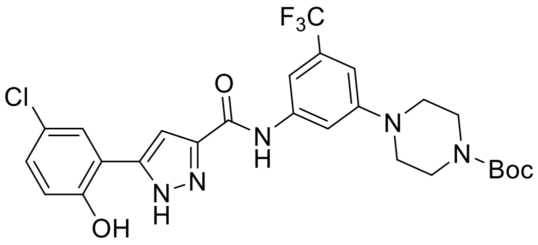

Compound **11B** was prepared following GP-1, chromene amide **48** (611 mg, 1.1 mmol) was reacted with hydrazine hydrate (438 mg, 8.7 mmol). The mixture stirred for 1 h and the solvent was removed under reduced pressure to obtain **11B** as white solid in quantitative yield (614 mg, 1.1 mmol).

^{1}H NMR (400 MHz, DMSO-*d*$_6$) δ = 10.27 (br s, 10H), 7.80 (d, *J* = 2.5 Hz, 1H), 7.77 (s, 1H), 7.68 (s, 1H), 7.39 (br s, 1H), 7.24 (dd, *J* = 8.8, 2.5 Hz, 1H), 7.01 (d, *J* = 8.8 Hz, 1H), 6.96 (s, 1H), 3.48 (t, *J* = 5.4 Hz, 4H), 3.20 (br t, *J* = 5.4 Hz, 4H), 1.42 (s, 9H) ppm.

^{13}C NMR (101 MHz, DMSO-*d*$_6$) δ = 154.6, 153.9, 152.0, 140.8, 131.4, 131.1, 130.8, 129.2, 127.1, 126.2, 123.8, 123.4, 118.9, 118.8, 110.8, 107.7, 107.4, 105.9, 79.6, 48.3, 43.6, 28.6 ppm.

^{19}F NMR (376 MHz, DMSO-*d*$_6$) δ = -61.29 ppm.

HRMS (ESI$^-$): *m/z* calcd. for $^-$ ([*M*-H]$^-$) 564.1631, measured 564.1617.

5-(5-Chloro-2-hydroxyphenyl)-*N*-(3-(4-(4,5-dihydro-1*H*-imidazol-2-yl)piperazin-1-yl)-5-(trifluoromethyl)phenyl)-1*H*-pyrazole-3-carboxamide (**11C**):

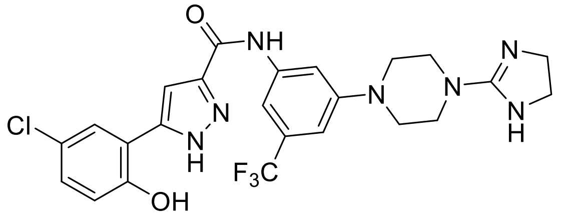

Compound **11C** was prepared following GP-3 with adaptations. Amine **11A** (91 mg, 0.16 mmol) was reacted with DIPEA (152 mg, 1.2 mmol) and guanidinylation agent **63** (122 mg, 0.50 mmol), the reaction mixture stirred for 20 h. After water (20 mL) addition, the obtained precipitate was filtered off and purified by flash column chromatography using NH_4OH:MeOH:DCM (1:4:15) as eluent system to obtain **11C** as white solid (20 mg, 0.037, 23%).

^{1}H NMR (600 MHz, DMSO-d_6, 50 °C): δ = 10.07 (br s, 1H), 7.85 (s, 1H), 7.69 (s, 1H), 7.56 (d, J = 2.5 Hz, 1H), 7.07 (s, 1H), 7.01 (dd, J = 8.6, 2.5 Hz, 1H), 6.93 (s, 1H), 6.81 (d, J = 8.6 Hz, 1H), 3.63 (s, 4H), 3.57 (t, J = 5.0 Hz, 4H), 3.36 (t, J = 5.0 Hz, 4H) ppm.

^{13}C NMR (151 MHz, DMSO-d_6, 50 °C): δ = 161.6, 159.6, 156.4, 150.8, 147.5, 145.2, 140.9, 130.1, 126.4, 125.2, 124.5, 120.5, 119.6, 118.0, 109.3, 106.4, 106.1, 101.4, 46.7, 45.3, 43.5 ppm

^{19}F NMR (376 MHz, DMSO-d_6) δ = -61.2 (s, 1F) ppm.

HRMS (ESI+): m/z calcd. for $C_{24}H_{24}ClF_3N_7O_2^+$ ([M+H]$^+$) 534.1627, measured 534.1601

N-(3-(4-Carbamimidoylpiperazin-1-yl)-5-(trifluoromethyl)phenyl)-5-(5-chloro-2-hydroxyphenyl)-1*H*-pyrazole-3-carboxamide (**11G**):

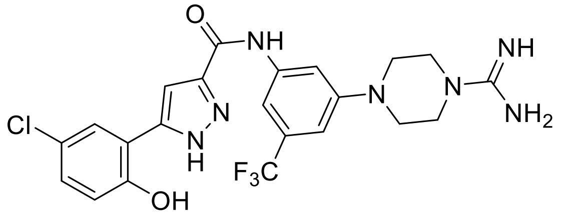

Compound **11G** was prepared following GP-3, amine **11A** (94 mg, 0.17 mmol) was reacted with DIPEA (152 mg, 1.17 mmol) and guanidinylation agent **65** (36 mg, 0.25 mmol). The reaction mixture stirred for 48 h to obtain **11G** as light grey solid (38 mg, 0.08 mmol, 46%).

^{1}H NMR (400 MHz, DMSO-d_6) δ = 10.64 (br s, 1H), 7.82 (br s, 2H), 7.74 (br s, 1H), 7.65 (br s, 3H), 7.44 (br s, 1H), 7.24 (br d, J = 6.6 Hz, 2H), 7.08 (br d, J = 6.6 Hz, 1H), 7.01 (br s, 1H), 3.62 (s, 4H), 3.35 (s, 4H) ppm.

^{13}C NMR (151 MHz, DMSO-d_6) δ = 160.8, 156.3, 153.4, 150.8, 146.7, 140.5, 139.7, 130.1, 128.9, 126.5, 125.2, 122.8, 118.1, 117.2, 109.7, 106.9, 106.9, 106.2, 46.8, 44.5 ppm.

^{19}F NMR (376 MHz, DMSO-d_6) δ = -61.23 ppm.

HRMS (ESI$^+$): *m/z* calcd. for $C_{22}H_{22}ClF_3N_7O_2^+$ ([*M*+H]$^+$) 508.1470, measured 508.1443.

5-(5-Chloro-2-hydroxyphenyl)-*N*-(4-(piperazin-1-yl)-3-(trifluoromethyl)phenyl)-1*H*-pyrazole-3-carboxamide (**12A**):

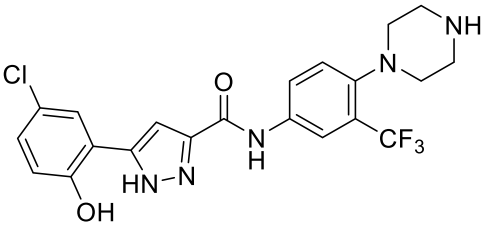

Compound **12A** was prepared following GP-2 with adaptations, *N*-Boc amine **12B** (140 mg, 0.25 mmol) was reacted with TFA (6.2 mmol) and the reaction mixture stirred for 20 h. A precipitate appeared which was filtered and washed with DCM (1 mL) to obtain **12A** as light grey solid in form of a TFA salt (113 mg, 0.20 mmol, 81%).

^{1}H NMR (400 MHz, DMSO-d_6) δ = 10.60 (br s, 1H), 10.49 (s, 1H), 8.83 (br s, 2H), 8.25 (s, 1H), 8.15 (dd, J = 8.6, 2.3 Hz, 1H), 7.81 (d, J = 2.3 Hz, 1H), 7.56 (d, J = 8.6 Hz, 1H), 7.37 (br s, 1H), 7.25 (dd, J = 8.6, 2.3 Hz, 1H), 7.02 (d, J = 8.6 Hz, 1H), 3.21 (br s, 4H), 3.04 (br s, 4H) ppm.

^{13}C NMR (101 MHz, DMSO-d_6) δ = 158.8, 158.4, 153.4, 145.8, 136.7, 128.9, 127.9, 126.5, 126.1, 125.9, 125.1, 124.9, 122.9, 122.4, 118.5, 118.1, 106.1, 50.0, 43.6 ppm.

^{19}F NMR (376 MHz, DMSO-d_6) δ = -59.31, -73.81 ppm.

HRMS (ESI$^+$): *m/z* calcd. for $C_{21}H_{20}ClF_3N_5O_2^+$ ([*M*+H]$^+$) 466.1252, measured 466.1227.

tert-Butyl 4-(4-(5-(5-chloro-2-hydroxyphenyl)-1*H*-pyrazole-3-carboxamido)-2-(trifluoromethyl)phenyl)piperazine-1-carboxylate (**12B**):

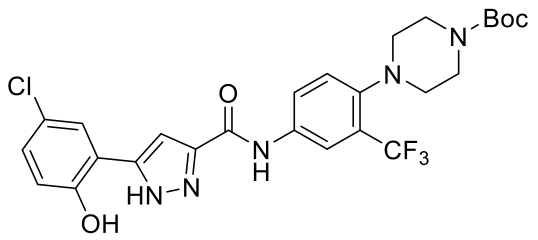

Compound **12B** was prepared following GP-1 with adaptations, chromene amide **49** (401 mg, 0.83 mmol) was reacted with hydrazine hydrate (338 mg, 6.8 mmol) and the mixture stirred for 30 min. A precipitate appeared, which was filtered and washed with ice-cold EtOH (1 mL) to obtain **13B** as beige solid (405 mg, 0.81 mmol, 98%).

^{1}H NMR (400 MHz, DMSO-*d$_6$*) δ = 10.40 (br s, 1H), 8.24 (d, *J* = 2.3 Hz, 1H), 8.08 (dd, *J* = 8.6, 2.3 Hz, 1H), 7.76 (d, *J* = 2.3 Hz, 1H), 7.57 (d, *J* = 8.6 Hz, 1H), 7.34 (s, 1H), 7.20 (dd, *J* = 8.6, 2.3 Hz, 1H), 6.97 (d, *J* = 8.6 Hz, 1H), 3.43 (br s, 4H), 2.79 (br t, *J* = 4.7 Hz, 4H), 1.43 (s, 9H) ppm.

^{13}C NMR (101 MHz, DMSO-*d$_6$*) δ = 159.6, 153.9, 153.3, 147.1, 136.2, 128.8, 126.5, 126.1, 125.8, 125.5, 125.3, 124.8, 122.9, 122.5, 118.3, 118.3, 118.1, 106.0, 79.0, 53.0, 44.2, 28.1 ppm.

^{19}F NMR (376 MHz, DMSO-*d$_6$*) δ = -59.08 ppm.

HRMS (ESI$^+$): *m/z* calcd. for $C_{26}H_{28}ClF_3N_5O_4^+$ ([*M*+H]$^+$) 566.1776, measured 566.1755.

N-(4-(4-Carbamimidoylpiperazin-1-yl)-3-(trifluoromethyl)phenyl)-5-(5-chloro-2-hydroxyphenyl)-1*H*-pyrazole-3-carboxamide (**12G**):

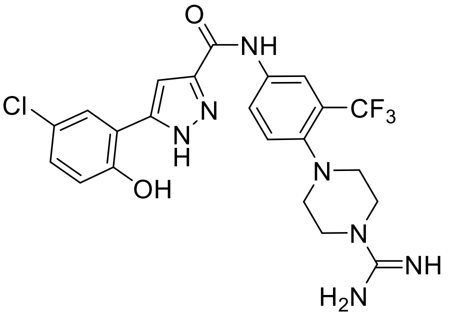

Compound **12G** was prepared following GP-3, amine **12A** (260 mg, 0.51 mmol) was reacted with DIPEA (403 mg, 3.12 mmol) and guanidinylation agent **65** (114 mg, 0.78 mmol). The reaction mixture stirred for 20 h and **4G** was obtained as off-white solid in form of a TFA salt (190 mg, 0.37 mmol, 75%).

^{1}H NMR (400 MHz, DMSO-*d$_6$*): δ = 10.51 (br s, 1H), 8.26 (br s, 1H), 8.12 (br s, 1H), 7.81 (br s, 1H), 7.61 (br s, 4H), 7.44 (br s, 1H), 7.25 (br s, 1H), 7.05 (br s, 1H), 3.54 (br s, 4H), 2.91 (br s, 4H) ppm.

^{13}C NMR (151 MHz, DMSO-*d$_6$*) δ = 158.4, 158.2, 156.4, 153.3, 146.2, 136.5, 128.8, 126.5, 126.0, 125.5, 124.8, 124.7, 122.9, 122.9, 121.1, 118.3, 118.1, 106.1, 52.3, 45.7 ppm.

^{19}F NMR (376 MHz, DMSO-*d$_6$*) δ = -59.13, -73.65 ppm.

HRMS (ESI$^+$): *m/z* calcd. for $C_{22}H_{22}ClF_3N_7O_2^+$ ([*M*+H]$^+$) 508.1470, measured 508.1441.

5-(5-Chloro-2-hydroxyphenyl)-*N*-(3-(piperazin-1-yl)-4-(trifluoromethyl)phenyl)-1*H*-pyrazole-3-carboxamide (**13A**):

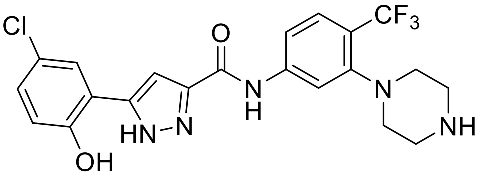

Compound **13A** was prepared following GP-2, *N*-Boc amine **13B** (150 mg, 0.27 mmol) was reacted with TFA (8.0 mmol) and the reaction mixture stirred for 3 h. Compound **13A** was obtained as white solid in form of a TFA salt in quantitative yield (150 mg, 0.27 mmol).

^{1}H NMR (400 MHz, DMSO-*d$_6$*) δ = 10.69 (br s, 1H), 10.55 (s, 1H), 8.97 (s, 1H), 8.08 (br s, 1H), 7.90 (d, *J* = 8.8 Hz, 1H), 7.82 (d, *J* = 2.4 Hz, 1H), 7.67 (d, *J* = 8.8 Hz, 1H), 7.41 (br s, 1H), 7.26 (dd, *J* = 8.8, 2.4 Hz, 1H), 7.04 (d, *J* = 8.8 Hz, 1H), 3.23 (br s, 4H), 3.07 (br s, 4H) ppm.

^{13}C NMR (101 MHz, DMSO-*d$_6$*) δ = 158.2, 153.3, 151.3, 143.6, 128.9, 128.1, 127.8, 126.5, 125.4, 122.9, 122.7, 120.1, 119.8, 118.1, 116.6, 115.2, 106.2, 50.0, 43.6 ppm.

^{19}F NMR (376 MHz, DMSO-*d$_6$*) δ = -58.28, -74.01 ppm.

HRMS (ESI$^+$): *m/z* calcd. for $C_{21}H_{20}ClF_3N_5O_2^+$ ([*M*+H]$^+$) 466.1252, measured 466.1227.

tert-Butyl 4-(5-(5-(5-chloro-2-hydroxyphenyl)-1*H*-pyrazole-3-carboxamido)-2-(trifluoromethyl)phenyl)piperazine-1-carboxylate (**13B**):

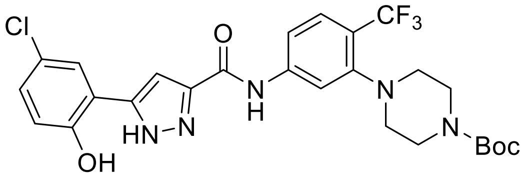

Compound **13B** was prepared following GP-1, chromene amide **50** (170 mg, 0.31 mmol) was reacted with hydrazine hydrate (141 mg, 2.8 mmol). The mixture stirred for 2 h and the solvent was removed under reduced pressure to obtain **13B** as white solid (169 mg, 0.30 mmol, 96%).

^{1}H NMR (400 MHz, DMSO-*d*$_6$) *δ* = 10.42 (br s, 1H), 7.99 (s, 1H), 7.90 (br d, *J* = 8.6 Hz, 1H), 7.81 (d, *J* = 2.5 Hz, 1H), 7.65 (d, *J* = 8.6 Hz, 1H), 7.41 (br s, 1H), 7.25 (dd, *J* = 8.8, 2.5 Hz, 1H), 7.02 (d, *J* = 8.8 Hz, 1H), 3.46 (br s, 4H), 2.81 (t, *J* = 4.5 Hz, 4H), 1.43 (s, 9H) ppm.
^{13}C NMR (101 MHz, DMSO-*d*$_6$) *δ* = 153.9, 153.3, 152.4, 143.4, 128.9, 127.6, 126.5, 125.5, 122.9, 122.8, 120.1, 119.8, 118.1, 116.1, 115.4, 106.1, 79.0, 53.1, 28.1 ppm. Quaternary carbon peaks missing.
^{19}F NMR (376 MHz, DMSO-*d*$_6$) *δ* = -58.03 ppm.
HRMS (ESI$^+$): *m/z* calcd. for $C_{26}H_{28}ClF_3N_5O_4^+$ ([*M*+H]$^+$) 566.1776, measured 566.1751.

N-(3-(4-Carbamimidoylpiperazin-1-yl)-4-(trifluoromethyl)phenyl)-5-(5-chloro-2-hydroxyphenyl)-1*H*-pyrazole-3-carboxamide (**13G**):

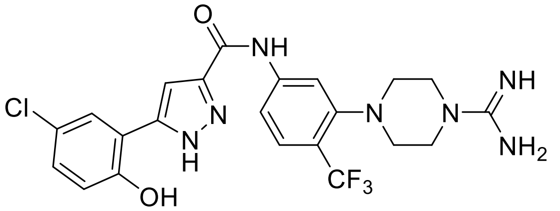

Compound **13G** was prepared following GP-3, amine **13A** (129 mg, 0.23 mmol) was reacted with DIPEA (190 mg, 1.47 mmol) and guanidinylation agent **65** (52 mg, 0.36 mmol). The reaction mixture stirred for 6 h and **13G** was obtained as white solid in form of a TFA salt (103 mg, 0.20 mmol, 88%).

^{1}H NMR (400 MHz, DMSO-*d*$_6$) *δ* = 8.11 (s, 1H), 7.89 (d, *J* = 9.0 Hz, 1H), 7.61 (d, *J* = 9.0 Hz, 1H), 7.53 (br d, *J* = 2.3 Hz, 1H), 7.02 (s, 1H), 6.98 (dd, *J* = 8.6, 2.3 Hz, 1H), 6.76 (d, *J* = 8.6 Hz, 1H), 3.56 (br s, 4H), 2.93 (br s, 4H) ppm.
^{13}C NMR (101 MHz, DMSO-*d*$_6$): δ = 162.8, 157.2, 156.7, 152.1, 149.6, 146.0, 144.7, 128.0, 126.5, 124.7, 123.4, 121.0, 120.6, 119.3, 118.5, 116.0, 115.1, 101.3, 52.8, 46.2 ppm.
^{19}F NMR (376 MHz, DMSO-*d*$_6$): δ = -57.82 ppm.
HRMS (ESI$^+$): *m/z* calcd. for $C_{22}H_{22}ClF_3N_7O_2^+$ ([*M*+H]$^+$) 508.1470, measured 508.1451.

(5-(5-Chloro-2-hydroxyphenyl)-1*H*-pyrazol-3-yl)(piperazin-1-yl)methanone (**14A**):

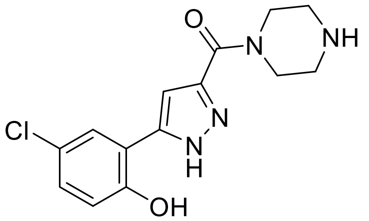

Compound **14A** was prepared following GP-2. *N*-Boc amine **14B** (280 mg, 0.69 mmol) was reacted with TFA (15 mmol) and the reaction mixture stirred overnight. Compound **4A** was obtained as grey powder in form of a TFA salt (270 mg, 0.67 mol, 98%).

^{1}H NMR (400 MHz, DMSO-*d*$_6$) *δ* = 13.41 (br s, 1H), 10.71 (br s, 1H), 9.04 (br s, 2H), 7.75 (br s, 1H), 7.24 (d, *J* = 7.4 Hz, 1H), 7.14 (s, 1H), 7.00 (d, *J* = 7.4 Hz, 1H), 4.27 (br s, 2H), 3.84 (br s, 2H), 3.25-3.73 (m, 4H) ppm.
^{13}C NMR (101 MHz, DMSO-*d*$_6$) *δ* = 161.9, 153.3, 146.3, 139.0, 128.9, 126.6, 122.9, 118.6, 118.1, 107.9, 43.4, 42.8 ppm.
^{19}F NMR (376 MHz, DMSO-*d*$_6$) *δ* = -73.70 ppm.
HRMS (ESI$^+$): *m/z* calcd. for $C_{14}H_{16}ClN_4O_2^+$ ([*M*+H]$^+$) 307.0956, measured 307.0938.

tert-Butyl 4-(5-(5-chloro-2-hydroxyphenyl)-1*H*-pyrazole-3-carbonyl)piperazine-1-carboxylate (**14B**):

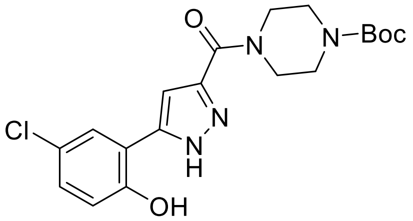

Compound **14B** was prepared following GP-1 with adaptations, chromene amide **51** (405 mg, 1.03 mmol) was reacted with hydrazine hydrate (422 mg, 8.4 mmol) and the mixture stirred for 2 h. A precipitate appeared, which was filtered and washed with ice-cold water (2 mL) to obtain **14B** as white solid (350 mg, 0.86 mmol, 83%). The product was used without further purification.

^{1}H NMR (400 MHz, DMSO-*d*$_6$) *δ* = 7.79 (s, 1H), 7.22 (d, *J* = 7.6 Hz, 1H), 7.15 (s, 1H), 6.98 (d, *J* = 7.6 Hz, 1H), 3.90 (br s, 1H), 3.62 (br s, 4H), 3.33 (br s, 4H), 1.42 (br s, 9H) ppm.

4-(5-(5-Chloro-2-hydroxyphenyl)-1*H*-pyrazole-3-carbonyl)piperazine-1-carboximidamide (**14G**):

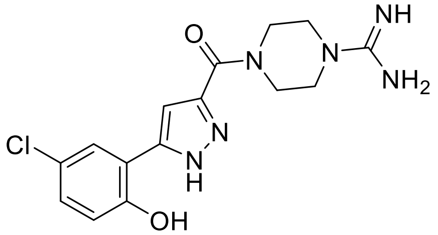

Compound **14G** was prepared following GP-3 with adaptations, amine **14A** (89 mg, 0.22 mmol) was reacted with DIPEA (175 mg, 1.35 mmol) and guanidinylation agent **65** (48 mg, 0.33 mmol). The reaction mixture stirred for 2 h obtaining an off-white solid (35 mg). Part of the crude (15 mg, 43%) was purified by preparative HPLC to afford **14G** as white powder (7 mg, 0.02 mmol, 18%).

^{1}H NMR (500 MHz, DMSO-*d*$_6$) δ = 8.43 (s, 1H), 7.95 (br s, 3H), 7.76 (br s, 1H), 7.21 (br d, *J* = 7.8 Hz, 1H), 7.15 (s, 1H), 6.99 (br d, *J* = 7.8 Hz, 1H), 4.11 (br s, 2H), 3.73 (br s, 2H), 3.53 (br s, 4H) ppm.

^{13}C NMR (126 MHz, DMSO-*d*$_6$) δ = 167.2, 162.2, 157.1, 154.3, 141.8, 129.1, 126.8, 123.0, 118.6, 118.2, 107.3, 45.8, 45.6, 44.9, 41.6 ppm.

Intermediates 16–62

tert-Butyl (3-amino-5-(trifluoromethyl)benzyl)carbamate (**16**):

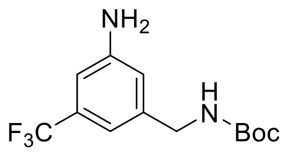

To a solution of compound **54** (137 mg, 0.74 mmol, 1 eq.) in DCM (2 mL), NEt_3 (149 mg, 1.48 mmol, 2 eq.) was added and, at 0 °C Boc_2O (171 mg, 0.78 mmol, 1.06 eq.) in DCM (2 mL) was added dropwise. The mixture was allowed to slowly warm to r.t. and stirred overnight. Excess DCM was removed *in vacuo* and the crude was purified by column chromatography using 30% EtOAc in PE to obtain compound **16** (59 mg, 0.20 mmol, 28%) as a orange solid.

^{1}H NMR (400 MHz, $CDCl_3$) δ = 6.89 (s, 1H), 6.79 (br s, 1H), 6.75 (br s, 1H), 4.27 (*J* = 4.7 Hz, 2H), 3.87 (s, 2H), 1.47 (s, 9H) ppm.

^{13}C NMR (101 MHz, $CDCl_3$) δ = 155.9, 147.1, 141.2, 131.8, 124.0, 116.7, 113.7, 110.3, 79.7, 44.2 ,28.3 ppm.

^{19}F NMR (376 MHz, $CDCl_3$) δ = -62.96 ppm.

tert-Butyl (4-amino-2-(trifluoromethyl)benzyl)carbamate (**17**):

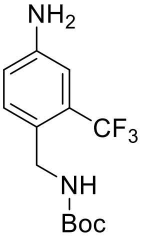

4-(aminomethyl)-3-(trifluoromethyl)aniline **55** (4126 mg, 21.7 mmol, 1 eq.) was dissolved in DCM (10 mL) and NEt_3 (4444 mg, 43.9 mmol, 2 eq.) was added. The reaction mixture was cooled to 0 °C and a cold solution of Boc_2O (4765 mg, 21.8 mmol, 1 eq) in DCM (10mL) was added dropwise. After 6 h, the mixture was quenched with saturated bicarbonate aq. (50 mL) and extracted with DCM (3 x 50 mL). The combined organic layers were washed with brine, dried over anhydrous $MgSO_4$, filtered, and concentrated *in vacuo*, obtaining an orange oil. The crude was purified by column chromatography using a gradient of 2–10% EtOAc in DCM as eluent to obtain compound **17** as orange solid (3543 mg, 12.2 mmol, 56%).

^{1}H NMR (400 MHz, $CDCl_3$) δ = 7.33 (br d, *J* = 8.2 Hz, 1H), 6.91 (d, *J* = 2.0 Hz, 1H), 6.78 (dd, *J* = 2.0, 8.2 Hz, 1H), 4.83 (br s, 1H), 4.35 (d, *J* = 5.9 Hz, 2H), 3.84 (s, 2H), 1.45 (s, 9H) ppm.

^{13}C NMR (101 MHz, $CDCl_3$) δ = 155.7, 145.7, 132.0, 128.4, 126.4, 124.3, 117.9, 112.1, 79.4, 40.8, 28.4 ppm.

^{19}F NMR (376 MHz, $CDCl_3$) δ = -59.76 ppm.

tert-Butyl 4-(4-amino-2-(trifluoromethyl)phenyl)piperazine-1-carboxylate (**18**):

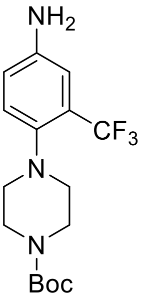

Compound **58** (136.4 mg, 0.36 mmol, 1 eq) was dissolved in EtOH (10 mL) and sodium dithionite (261.5 mg, 1.5 mmol, 4.1 eq) was added. The reaction mixture was heated to 90 °C. After 6 h the mixture was quenched with water (15 mL) and extracted with EtOAc (3 x 30 mL). The combined organic layers were washed with brine, dried over anhydrous $MgSO_4$, filtered, and concentrated to dryness *in vacuo*. The resulting crude was purified by column chromatography using 30% EtOAc in PE to obtain compound **18** as a light brown powder (75.3 mg, 0.22 mmol, 60%).

^{1}H NMR (400 MHz, $CDCl_3$) δ = 7.14 (d, *J* = 8.6 Hz, 1H), 6.91 (d, *J* = 2.5 Hz, 1H), 6.79 (dd, *J* = 2.5, 8.6 Hz, 1H), 3.76 (s, 2H), 3.52 (br s, 4H), 2.77 (br t, *J* = 4.5 Hz, 4H), 1.49 (s, 9H) ppm.

^{13}C NMR (101 MHz, $CDCl_3$) δ = 154.7, 143.6, 142.7, 128.4, 125.1, 123.6, 118.3, 112.7, 79.4, 53.3, 44.4, 28.2 ppm.

^{19}F NMR (376 MHz, $CDCl_3$) δ = -60.88 ppm.

tert-Butyl 4-(3-amino-5-(trifluoromethyl)phenyl)piperazine-1-carboxylate (**19**):

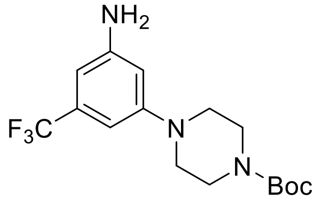

Compound **59** (2000 mg, 5.33 mmol, 1 eq.) was suspended in EtOH (20 mL) and heated to 80 °C. The solution was allowed to cool for 5 min and sodium dithionite (3794 mg, 21.8 mmol, 4.1 eq) was added under vigorous stirring. The reaction mixture was heated to 90 °C, the yellow suspension changed into a white colour and after 6 h the reaction was complete. The reaction mixture was filtered over celite while warm and water was added (50 mL). The mixture was extracted with EtOAc (3 x 50 mL) and the combined organic layers were washed with brine, dried over anhydrous $MgSO_4$, filtered, and concentrated to dryness *in vacuo*. The resulting crude was purified by column chromatography using 2.5% MeOH in DCM to obtain compound **19** as a pale yellow powder (1181 mg, 3.42 mmol, 64%).
^{1}H NMR (400 MHz, $CDCl_3$) δ = 6.55 (s, 1H), 6.44 (s, 1H), 6.34 (s, 1H), 3.79 (s, 2H), 3.60 (s, 4H), 3.11 (s, 4H), 1.49 (s, 9H) ppm.
^{13}C NMR (101 MHz, $CDCl_3$) δ = = 154.6, 152.5, 147.7, 132.3, 125.0, 105.1, 103.4, 80.0, 48.9, 41.9, 28.4 ppm.
^{19}F NMR (376 MHz, $CDCl_3$) δ = -63.15 ppm.

tert-Butyl 4-(5-amino-2-(trifluoromethyl)phenyl)piperazine-1-carboxylate (**20**):

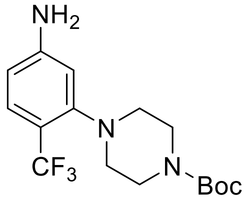

Compound **62** (3332 mg, 8.9 mmol, 1 eq.) was dissolved in EtOH (30 mL) and heated to 50 °C. Sodium dithionite (6284 mg, 36.1 mmol, 4.1 eq.) was added to the stirring solution. The reaction mixture stirred at reflux for 6 h and was filtered over celite while warm and water was added (50 mL). The mixture was extracted with EtOAc (3 x 50 mL) and the combined organic layers were washed with brine, dried over anhydrous $MgSO_4$, filtered, and concentrated to dryness *in vacuo*. The resulting crude was purified by column chromatography using 15% EtOAc in DCM to obtain compound **20** as a pale yellow powder (506 mg, 1.46 mmol, 17%).
^{1}H NMR (400 MHz, $CDCl_3$) δ = 7.37 (d, J = 8.2 Hz, 1H), 6.51 (s, 1H), 6.45 (d, J = 8.6 Hz, 1H), 3.99 (s, 2H), 3.57 (br t, J = 4.5 Hz, 4H), 2.82 (br t, J = 4.5 Hz, 4H), 1.48 (s, 9H) ppm.
^{13}C NMR (101 MHz, $CDCl_3$) δ = 154.9, 153.6, 150.4, 128.6, 124.5, 116.5, 110.5, 109.3, 79.6, 53.2, 44.6, 28.4 ppm.
^{19}F NMR (376 MHz, $CDCl_3$) δ = -58.78 ppm.

6-Chloro-4-oxo-*N*-[3-(trifluoromethyl)phenyl]chromene-2-carboxamide (**34**):

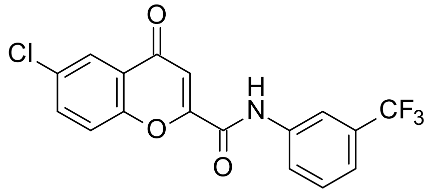

Compound **34** was prepared following GP-4, carboxylic acid **15** (537 mg, 2.33 mmol) was reacted with DIPEA (353 mg, 2.66 mmol), HATU (1037 mg, 2.66 mmol) and 3-(trifluoromethyl)aniline **21** (362 mg, 2.22 mmol). The reaction mixture stirred for 22 h and the resulting crude was washed with EtOH (2 mL), filtered, and evaporated to dryness to obtain compound **34** as white powder (419 mg, 1.14 mmol, 51%).
^{1}H NMR (400 MHz, DMSO-d_6): δ = 11.00 (s, 1H), 8.19 (s, 1H), 8.06 (d, J = 8.6 Hz, 1H), 7.94 (d, J = 8.6 Hz, 1H), 7.85 (d, J = 8.6 Hz, 1H), 7.64 (t, J = 8.0, 1H), 7.53 (d, J = 8.0 Hz, 1H), 7.00 (s, 1 H) ppm.
^{13}C NMR (101 MHz, DMSO-d_6): δ = 176.6, 158.3, 155.8, 154.1, 138.8, 135.5, 131.1, 130.6, 130.1, 129.8, 125.2, 125.0, 124.4, 121.9, 121.7, 117.6, 111.7 ppm.
^{19}F NMR (470 MHz, DMSO-d_6) δ = -61.31 ppm.

6-Chloro-*N*-(3-fluorophenyl)-4-oxo-4*H*-chromene-2-carboxamide (**35**):

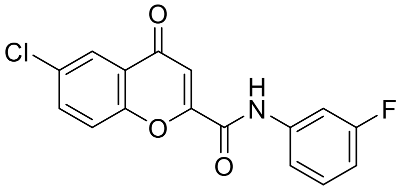

Compound **35** was prepared following GP-4, carboxylic acid **15** (263 mg, 1.17 mmol) was reacted with DIPEA (182 mg, 1.41 mmol), HATU (534 mg, 1.41 mmol) and 3-fluoroaniline **22** (124 mg, 1.11 mmol). The reaction mixture stirred for 4 h at rt, obtaining compound **35** as yellow powder (335 mg, 1.05 mmol, 95%). The product was used without further purification.

^{1}H NMR (400 MHz, DMSO-d_6) δ = 10.85 (br s, 1H), 7.98 (d, J = 2.2 Hz, 1H), 7.96 (dd, J = 8.8, 2.2 Hz, 1H), 7.86 (br d, J = 8.8 Hz, 1H), 7.71 (br d, J = 11.7 Hz, 1H), 7.59 (br d, J = 8.2 Hz, 1H), 7.45 (dd, J = 8.2, 6.6 Hz, 1H), 7.03 (br t, J = 8.2 Hz, 1H), 6.99 (s, 1H) ppm.

6-Chloro-*N*-(3-nitrophenyl)-4-oxo-4*H*-chromene-2-carboxamide (**36**):

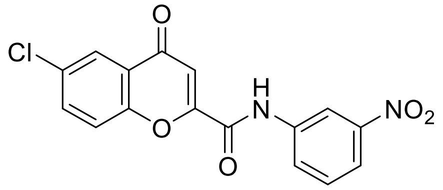

Compound **36** was prepared following GP-4 with adaptations, carboxylic acid **15** (263 mg, 1.17 mmol) was reacted with DIPEA (182 mg, 1.41 mmol), HATU (534 mg, 1.41 mmol) and 3-nitroaniline **23** (154 mg, 1.11 mmol) overnight at r.t. and at 80 °C for 20 h. The crude mixture was purified by flash column chromatography using 20% EtOAc in PE as eluent to obtain compound **36** as yellow powder (160 mg, 0.46 mmol, 34%).

^{1}H NMR (400 MHz, DMSO-d_6) δ = 11.07 (br s, 1H), 8.71 (s, 1H), 8.20 (d, J = 7.4 Hz, 1H), 8.02 (d, J = 7.8 Hz, 1H), 7.97 - 7.89 (m, 2H), 7.87 - 7.77 (m, 1H), 7.68 (t, J = 8.2 Hz, 1H), 6.99 (s, 1H) ppm.

^{13}C NMR (101 MHz, DMSO-d_6) δ = 176.6, 158.4, 155.6, 154.1, 148.3, 139.1, 135.5, 131.1, 130.8, 127.2, 125.2, 124.4, 121.8, 119.8, 115.6, 111.8 ppm.

6-Chloro-4-oxo-*N*-(p-tolyl)-4*H*-chromene-2-carboxamide (**37**):

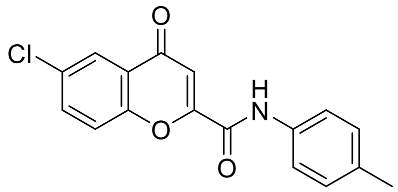

Compound **37** was prepared following GP-4, carboxylic acid **15** (200 mg, 0.89 mmol) was reacted with DIPEA (132 mg, 1.02 mmol), HATU (387 mg, 1.02 mmol) and *p*-toluidine **24** (91 mg, 0.85 mmol). The reaction mixture stirred for 2 h, obtaining compound **37** as off-white solid (122 mg, 0.39 mmol, 46%).

^{1}H NMR (400 MHz, DMSO-d_6) δ = 10.68 (br s, 1H), 8.01 (d, J = 2.3 Hz, 1H), 7.98 (br dd, J = 2.3, 9.0 Hz, 1H), 7.89 (br d, J = 9.3 Hz, 1H), 7.67 (br d, J = 8.2 Hz, 2H), 7.22 (br d, J = 8.2 Hz, 2H), 6.99 (s, 1H), 2.30 (s, 3H) ppm.

^{13}C NMR (101 MHz, DMSO-d_6) δ = 176.8, 157.7, 156.4, 154.2, 135.4, 135.4, 134.7, 131.0, 129.7, 125.3, 124.4, 122.0, 121.5, 111.4, 21.0 ppm.

6-Chloro-2-(morpholine-4-carbonyl)-4*H*-chromen-4-one (**38**):

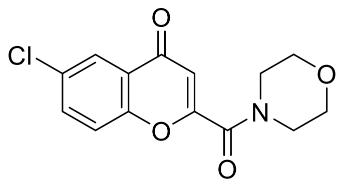

Compound **38** was prepared following GP-4, carboxylic acid **15** (308 mg, 0.89 mmol) was reacted with DIPEA (212 mg, 1.6 mmol), HATU (610 mg, 1.6 mmol) and morpholine **25** (109 mg, 1.25 mmol). The reaction mixture stirred for 24 h and was quenched with water (20 mL) and extracted with EtOAc (3 x 20 mL). The combined organic layers were washed with brine, dried over anhydrous $MgSO_4$, filtered, and concentrated *in vacuo*, obtaining a yellow oil. The crude was purified by column chromatography using 10% MeOH in DCM to obtain compound **38** as yellow powder (110 mg, 0.37 mmol, 28%).

^{1}H NMR (400 MHz, DMSO-d_6) δ = 7.98 (d, J = 2.5 Hz, 1H), 7.90 (dd, J = 2.5, 9.0 Hz, 1H), 7.77 (d, J = 9.0 Hz, 1H), 6.63 (s, 1H), 3.54-3.71 (m, 8H) ppm.

^{13}C NMR (101 MHz, DMSO-d_6) δ = 176.03, 160.40, 158.23, 154.51, 134.98, 130.84, 125.29, 124.29, 121.60, 111.19, 66.59, 66.12, 47.33, 42.54 ppm.

6-Chloro-*N*-(3-(morpholinomethyl)phenyl)-4-oxo-4*H*-chromene-2-carboxamide (**39**):

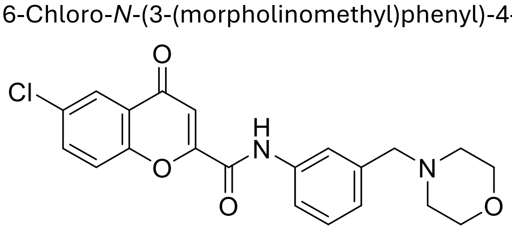

Compound **39** was prepared following GP-4, carboxylic acid **15** (200 mg, 0.89 mmol) was reacted with 3-(morpholinomethyl)aniline **26** (163 mg, 0.85 mmol) and the reaction mixture stirred for 4 h. White precipitate was filtered, obtaining compound **39** (277 mg, 0.69 mmol, 76%). The product was used without further purification.

^{1}H NMR (400 MHz, DMSO-d_6) δ = 11.13 (br s, 1H), 8.02 (br s, 1H), 8.03 - 7.92 (m, 2H), 7.85 (br d, J = 7.4 Hz, 1H), 7.56 (br s), 7.43 (br t, J = 7.4 Hz, 1H), 7.00 (s, 1H), 4.30 (br s, 2H), 3.84 (br s, 4H), 3.07 (br s, 4H) ppm.

tert-Butyl 4-(3-(6-chloro-4-oxo-4*H*-chromene-2-carboxamido)benzyl)piperazine-1-carboxylate (**40**):

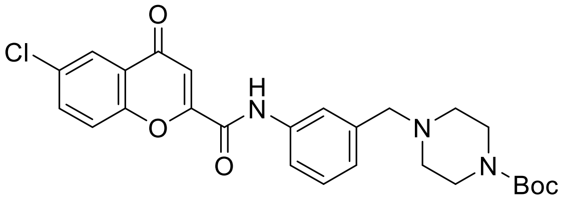

Compound **40** was prepared following GP-4, carboxylic acid **15** (250 mg, 1.11 mmol) was reacted with DIPEA (164 mg, 1.27 mmol), HATU (484 mg, 1.27 mmol) and *tert*-butyl 4-(3-aminobenzyl)piperazine-1-carboxylate **27** (309 mg, 1.06 mmol). The reaction mixture stirred for 2 h and the resulting crude was washed with DCM (2 mL), filtered, and evaporated to dryness to obtain compound **40** as off-white powder (277 mg, 0.56 mmol, 50%).

^{1}H NMR (400 MHz, DMSO-d_6) δ = 10.90 (br s, 1H), 8.05 (br s, 1H), 8.03 - 7.94 (m, 2H), 7.87 (br d, *J* = 8.6 Hz, 1H), 7.78 (br d, *J* = 7.8 Hz, 1H), 7.54 (br t, *J* = 7.2 Hz, 1H), 7.34 (br d, *J* = 6.6 Hz, 1H), 7.01 (br s, 1H), 4.37 (br s, 2H), 3.06 (br s, 4H), 2.49 (br s, 4H), 1.41 (s, 9H) ppm.

^{13}C NMR (101 MHz, DMSO-d_6) δ = 176.7, 158.1, 156.1, 154.2, 153.7, 138.3, 135.5, 131.1, 130.0, 128.3, 125.3, 124.4, 124.1, 124.1, 122.8, 121.9, 111.6, 80.4, 59.5, 51.0, 40.6, 28.4 ppm.

tert-Butyl 4-(4-(6-chloro-4-oxo-4*H*-chromene-2-carboxamido)benzyl)piperazine-1-carboxylate (**41**):

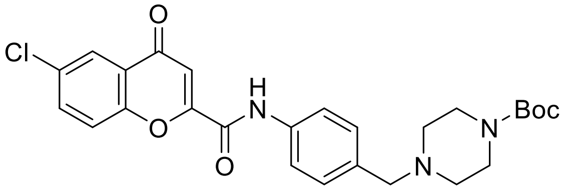

Compound **41** was prepared following GP-4, carboxylic acid **15** (250 mg, 1.11 mmol) was reacted with DIPEA (164 mg, 1.27 mmol), HATU (484 mg, 1.27 mmol) and *tert*-butyl 4-(4-aminobenzyl)piperazine-1-carboxylate **28** (309 mg, 1.06 mmol). The reaction mixture stirred for 2 h and the crude was purified by flash column chromatography using 5% MeOH in DCM as eluent to obtain compound **41** as off-white powder (172 mg, 0.35 mmol, 31%).

^{1}H NMR (400 MHz, DMSO-d_6) δ = 10.72 (br s, 1H), 8.00 (br d, *J* = 2.3 Hz, 1H), 7.97 (br dd, *J* = 2.3, 8.6 Hz, 1H), 7.88 (br d, *J* = 7.0 Hz, 1H), 7.73 (br d, *J* = 8.6 Hz, 2H), 7.33 (br d, *J* = 8.2 Hz, 2H), 6.99 (br s, 1H), 3.46 (br s, 2H), 3.30 - 3.24 (m, 4H), 2.30 (br t, *J* = 5.4 Hz, 4H), 1.37 (s, 9H) ppm.

^{13}C NMR (101 MHz, DMSO-d_6) δ = 176.7, 157.8, 156.4, 154.3, 154.2, 136.8, 135.4, 135.0, 131.0, 129.8, 125.3, 124.4, 122.0, 121.3, 111.4, 79.2, 61.9, 52.8, 43.4, 28.5 ppm.

tert-Butyl 4-(3-(6-chloro-4-oxo-4*H*-chromene-2-carboxamido)phenyl)piperazine-1-carboxylate (**42**):

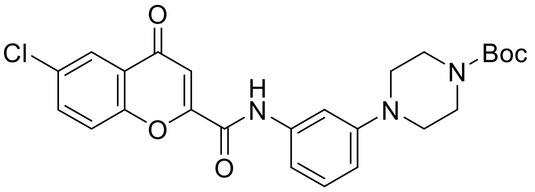

Compound **42** was prepared following GP-4, carboxylic acid **15** (310 mg, 1.38 mmol) was reacted with DIPEA (204 mg, 1.58 mmol), HATU (600 mg, 1.58 mmol) and *tert*-butyl 4-(3-aminophenyl)piperazine-1-carboxylate **29** (365 mg, 1.32 mmol). The reaction mixture stirred for 1 h and the resulting crude was impregnated on silica and purified by flash column chromatography using 5% MeOH in DCM as eluent to obtain compound **42** as off-white powder (230 mg, 0.48 mmol, 34%).

^{1}H NMR (400 MHz, DMSO-d_6) δ = 10.59 (br s, 1H), 8.03 - 7.95 (m, 2H), 7.89 (br d, *J* = 9.2 Hz, 1H), 7.40 (br s, 1H), 7.31 - 7.22 (m, 2H), 6.98 (br s, 1H), 6.80 (br d, *J* = 6.2 Hz, 1H), 3.48 (br s, 4H), 3.12 (br s, 4H), 1.42 (s, 9H) ppm.

^{13}C NMR (101 MHz, DMSO-d_6): δ = 176.7, 157.7, 156.4, 154.4, 154.2, 151.7, 138.7, 135.4, 131.0, 129.7, 125.3, 124.4, 122.0, 113.3, 112.6, 111.4, 109.0, 79.5, 48.7, 28.5 ppm.

tert-Butyl 4-(4-(6-chloro-4-oxo-4*H*-chromene-2-carboxamido)phenyl)piperazine-1-carboxylate (**43**):

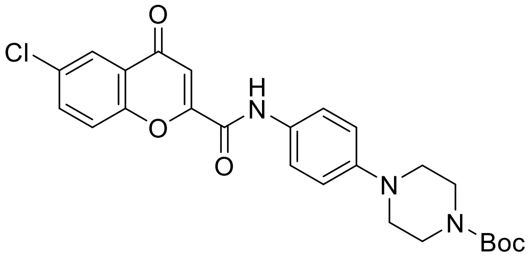

Compound **43** was prepared following GP-4, carboxylic acid **15** (250 mg, 1.11 mmol) was reacted with DIPEA (164 mg, 1.27 mmol), HATU (484 mg, 1.27 mmol) and *tert*-butyl 4-(4-aminophenyl)piperazine-1-carboxylate **30** (294 mg, 1.06 mmol). The reaction mixture stirred for 1 h the resulting crude was washed with EtOAc (2 mL) to obtain compound **43** as yellow powder (220mg, 0.45 mmol, 41%).

^{1}H NMR (400 MHz, DMSO-d_6) δ = 10.59 (br s, 1H), 8.00 (br d, J = 2.5 Hz, 1H), 7.96 (dd, J = 2.5, 9.0 Hz, 1H), 7.87 (d, J = 9.0 Hz, 1H), 7.65 (d, J = 9.0 Hz, 2H), 6.99 (d, J = 9.0 Hz, 2H), 6.96 (s, 1H), 3.46 (br t, J = 5.3 Hz, 4H), 3.10 (br t, J = 5.1 Hz, 4H), 1.42 (s, 9H) ppm.
^{13}C NMR (101 MHz, DMSO-d_6) δ = 176.7, 157.2, 156.6, 154.3, 148.6, 135.4, 131.0, 129.9, 125.3, 124.4, 123.4, 122.5, 121.8, 116.7, 111.2, 79.4, 49.3, 48.8, 28.5 ppm.

tert-Butyl (3-(6-chloro-4-oxo-4*H*-chromene-2-carboxamido)benzyl)carbamate (**44**):

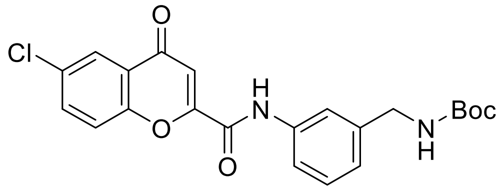

Compound **44** was prepared following GP-4, carboxylic acid **15** (250 mg, 1.11 mmol) was reacted with DIPEA (164 mg, 1.27 mmol), HATU (484 mg, 1.27 mmol) and *tert*-butyl (3-aminobenzyl)carbamate **27** (236 mg, 1.06 mmol). The reaction mixture stirred for 2 h, obtaining compound **44** as off-white powder (232 mg, 0.54 mmol, 49%).
^{1}H NMR (400 MHz, DMSO-d_6) δ = 10.76 (br s, 1H), 8.03 - 7.95 (m, 2H), 7.89 (br d, J = 9.4 Hz, 1H), 7.73 - 7.63 (m, 2H), 7.44 (br s, 1H), 7.36 (t, J = 7.8 Hz, 1H), 7.08 (br d, J = 7.4 Hz, 1H), 6.99 (br s, 1H), 4.15 (br d, J = 5.5 Hz, 2H), 1.40 (s, 9H) ppm.
^{13}C NMR (101 MHz, DMSO-d_6) δ = 176.8, 157.9, 156.4, 156.3, 154.2, 141.5, 137.9, 135.4, 131.0, 129.2, 125.3, 124.4, 124.1, 122.0, 120.1, 119.9, 111.4, 78.3, 43.8, 28.7 ppm.

tert-Butyl (4-(6-chloro-4-oxo-4*H*-chromene-2-carboxamido)benzyl)carbamate (**45**):

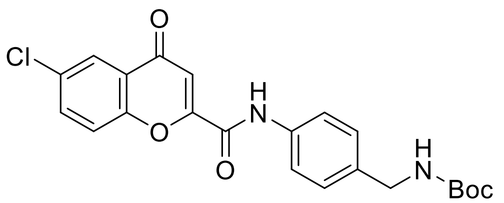

Compound **45** was prepared following GP-4, carboxylic acid **15** (250 mg, 1.11 mmol) was reacted with DIPEA (164 mg, 1.27 mmol), HATU (484 mg, 1.27 mmol) and *tert*-butyl (4-aminobenzyl)carbamate **28** (236 mg, 1.06 mmol). The reaction mixture stirred for 1 h, the crude was washed with acetone (2 mL) to obtain compound **45** as off-white powder (188 mg, 0.44 mmol, 39%).
^{1}H NMR (400 MHz, DMSO-d_6) δ = 10.73 (br s, 1H), 8.03 - 7.94 (m, 2H), 7.88 (br d, J = 8.9 Hz, 1H), 7.72 (br d, J = 7.0 Hz, 2H), 7.39 (br s, 1H), 7.28 (br d, J = 8.5 Hz, 2H), 6.99 (br s, 1H), 4.12 (br d, J = 3.6 Hz, 2H), 1.40 (s, 9H) ppm.
^{13}C NMR (101 MHz, DMSO-d_6) δ = 176.7, 157.8, 156.3, 156.2, 154.2, 137.4, 136.4, 135.4, 131.0, 127.8, 125.3, 124.4, 122.0, 121.5, 111.4, 78.3, 43.5, 28.7 ppm.

tert-Butyl (3-(6-chloro-4-oxo-4*H*-chromene-2-carboxamido)-5-(trifluoromethyl)benzyl)carbamate (**46**):

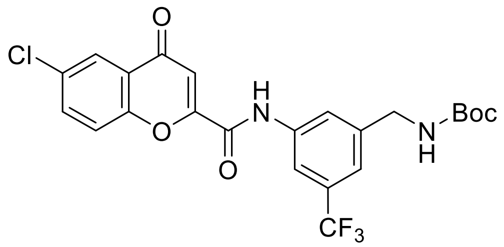

Compound **46** was prepared following GP-4, carboxylic acid **15** (105 mg, 0.47 mmol) was reacted with DIPEA (74 mg, 0.57 mmol), HATU (216 mg, 0.57 mmol) and aniline **16** (138 mg, 0.48 mmol). The reaction mixture stirred for 48 h, obtaining compound **46** as yellow powder (220 mg, 0.44 mmol, 95%).
^{1}H NMR (400 MHz, DMSO-d_6) δ = 11.01 (br s, 1H), 8.11 (s, 1H), 8.00 (s, 1H), 7.97 (s, 1H), 7.87 (d, J = 8.6 Hz, 1H), 7.55 (br s, 1H), 7.41 (s, 1H), 7.02 (s, 1H), 4.24 (d, J = 5.1 Hz, 2H), 1.41 (s, 9H) ppm.
^{19}F NMR (376 MHz, DMSO-d_6) δ = -61.41 ppm.

tert-Butyl *N*-[[4-[(6-chloro-4-oxo-chromene-2-carbonyl)amino]-2-(trifluoromethyl)phenyl]methyl]carbamate (**47**):

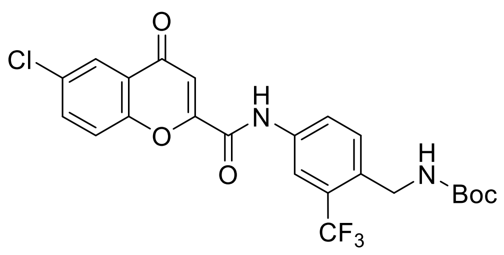

Compound **47** was prepared following GP-4 with adaptations, carboxylic acid **15** (2559 mg, 11.4 mmol) was reacted with DIPEA (1693 mg, 13.1 mmol), HATU (4968 mg, 13.1 mmol) and aniline **17** (3171 mg, 10.9 mmol). The reaction mixture stirred for 4 h, quenched with water (100 mL) and extracted with EtOAc (3 x 100 mL). The combined organic layers were washed with brine, dried over anhydrous $MgSO_4$, filtered, and concentrated under

reduced pressure. The crude was triturated with MeOH (10 mL) to obtain compound **47** as yellow powder (2050 mg, 4.1 mmol, 38%).

^{1}H NMR (400 MHz, DMSO-d_6) δ = 10.98 (s, 1H), 8.18 (s, 1H), 8.09 (d, J = 8.2 Hz, 1H), 8.00 (s, 1H), 7.87 (d, J = 9.0 Hz, 1H), 7.54 (d, J = 8.6 Hz, 1H), 7.51 (s, 1H), 7.02 (s, 1H), 4.31 (d, J = 5.1 Hz, 2H), 1.42 (s, 9H) ppm.

^{13}C NMR (101 MHz, DMSO-d_6) δ = 176.2, 157.7, 155.8, 155.4, 153.6, 136.5, 135.0, 134.4, 130.6, 128.9, 126.4, 124.8, 124.4, 123.9, 122.8, 121.4, 118.0, 111.2, 78.2, 40.1, 28.2 ppm.

^{19}F NMR (376 MHz, DMSO-d_6) δ = -59.32 ppm.

tert-Butyl 4-[3-[(6-chloro-4-oxo-chromene-2-carbonyl)amino]-5-(trifluoromethyl)phenyl]piperazine-1-carboxylate (**48**):

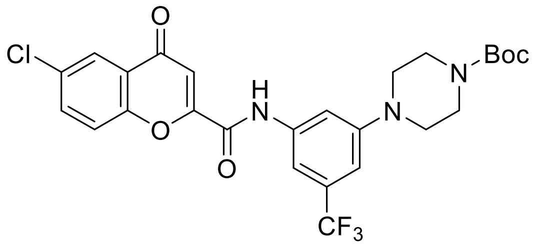

Compound **48** was prepared following GP-4, carboxylic acid **15** (530 mg, 2.36 mmol) was reacted with DIPEA (363 mg, 2.8 mmol), HATU (1074 mg, 2.8 mmol) and aniline **19** (138 mg, 0.48 mmol). The reaction mixture stirred for 24 h, and the crude was washed with ice-cold EtOH (5 mL) to obtain compound **48** as pale yellow powder (646.6 mg, 1.17 mmol, 52%).

^{1}H NMR (400 MHz, DMSO-d_6) δ = 8.01 (s, 1H), 7.98 (d, J = 2.7 Hz, 1H), 7.88 (d, J = 9.0 Hz, 1H), 7.65 (s, 1H), 7.64 (s, 1H), 7.06 (s, 1H), 7.01 (s, 1H), 3.49 (br s, 4H), 3.23 (br s, 4H), 1.43 (s, 9H) ppm.

^{13}C NMR (101 MHz, DMSO-d_6) δ = 176.2, 157.7, 155.49, 153.9, 153.6, 151.5, 139.1, 135.0, 130.6, 130.4, 130.1, 129.8, 130.1, 128.2, 125.5, 124.0, 121.4, 121.5, 111.1, 110.6, 108.1, 107.4, 79.1, 47.6, 39.9, 28.1 ppm.

^{19}F NMR (376 MHz, DMSO-d_6) δ = -61.38 ppm.

tert-Butyl 4-[4-[(6-chloro-4-oxo-chromene-2-carbonyl)amino]-2-(trifluoromethyl)phenyl]piperazine-1-carboxylate (**49**):

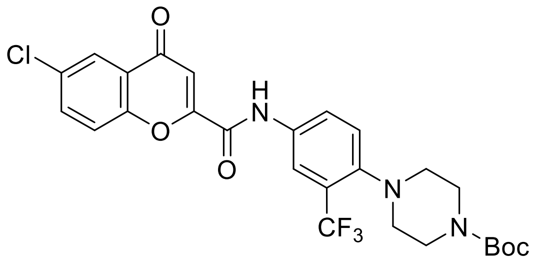

Compound **49** was prepared following GP-4, carboxylic acid **15** (248 mg, 1.1 mmol) was reacted with DIPEA (176 mg, 1.34 mmol), HATU (509 mg, 1.34 mmol) and aniline **18** (138 mg, 0.48 mmol). The reaction mixture stirred for 24 h, and the crude was washed with diethyl ether (3 mL) to obtain compound **49** as white powder (311 mg, 0.56 mmol, 51%).

^{1}H NMR (400 MHz, DMSO-d_6) δ = 10.93 (s, 1H), 8.15 (s, 1H), 8.08 (d, J = 9.0 Hz, 1H), 8.00 (s, 1H), 7.97 (br s, 1H), 7.87 (d, J = 8.6 Hz, 1H), 7.63 (d, J = 8.2 Hz, 1H), 7.01 (s, 1H), 3.43 (br s, 4H), 2.81 (br s, 4H), 1.43 (s, 9H) ppm.

^{13}C NMR (101 MHz, DMSO-d_6) δ = 153.9, 153.3, 147.1, 136.2, 128.8, 126.5, 126.1, 125.8, 125.5, 125.3, 124.8, 122.9, 123.9, 118.3, 118.1, 106.0, 79.0, 53.0, 44.1, 28.1 ppm.

^{19}F NMR (376 MHz, DMSO-d_6) δ = -58.99 ppm.

tert-Butyl 4-[5-[(6-chloro-4-oxo-chromene-2-carbonyl)amino]-2-(trifluoromethyl)phenyl]piperazine-1-carboxylate (**50**):

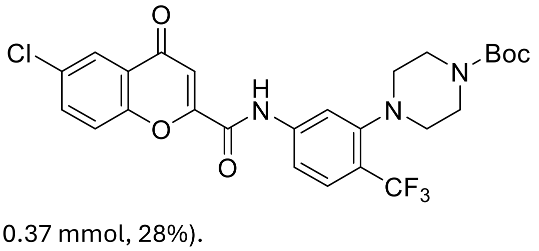

Compound **50** was prepared following GP-4, carboxylic acid **15** (295 mg, 1.3 mmol) was reacted with DIPEA (210 mg, 1.6 mmol), HATU (592 mg, 1.6 mmol) and aniline **20** (421 mg, 1.22 mmol). The reaction mixture stirred for 24 h, and the crude was purified by column chromatography using 5% MeOH in DCM to obtain compound **50** as pale yellow powder (204 mg, 0.37 mmol, 28%).

^{1}H NMR (400 MHz, DMSO-d_6) δ = 10.89 (s, 1H), 7.97 (s, 1H), 7.95 (d, J = 1.0 Hz, 1H), 7.91 (s, 1H), 7.85 (d, J = 9.8 Hz, 1H), 7.81 (br d, J = 8.6 Hz, 1H), 7.69 (d, J = 8.6 Hz, 1H), 7.00 (s, 1H), 3.46 (br s, 4H), 2.82 (br t, J = 3.9 Hz, 4H), 1.43 (s, 9H) ppm.

^{13}C NMR (101 MHz, DMSO-d_6) δ = 176.1, 157.8, 155.2, 153.8, 153.6, 152.4, 142.0, 135.0, 130.6, 127.9, 124.8, 123.9, 123.9,121.4, 121.2, 116.9, 116.0, 111.3, 79.0, 53.1, 43.5, 28.0 ppm.

^{19}F NMR (376 MHz, DMSO-d_6) δ = 58.35 ppm.

tert-Butyl 4-(6-chloro-4-oxo-chromene-2-carbonyl)piperazine-1-carboxylate (**51**):

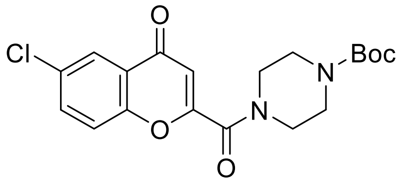

Compound **51** was prepared following GP-4 with adaptations, carboxylic acid **15** (996.0 mg, 4.43 mmol) was reacted with DIPEA (700 mg, 5.4 mmol), HATU (2037 mg, 5.4 mmol) and *tert*-butyl piperazine-1-carboxylate **33** (797 mg, 4.3 mmol). The reaction mixture stirred for 72 h, quenched with water (25 mL) and extracted with EtOAc (3 x 25 mL). The combined organic layers were washed with brine, dried over anhydrous $MgSO_4$, filtered, and the solvent was removed under reduced pressure. The resulting crude was triturated with ice-cold MeOH (5 mL) to obtain compound **51** as light grey powder (463 mg, 1.18 mmol, 27%).

^{1}H NMR (400 MHz, DMSO-d_6) δ = 7.96 (br s, 1H), 7.88 (br s, 1H), 7.76 (br d, J = 2.3 Hz, 1H), 6.61 (br s, 1H), 3.53 (s, 4H), 3.29 (br s, 4H), 1.39 (br s, 9H) ppm.

^{13}C NMR (101 MHz, DMSO-d_6) δ = 175.6, 160.1, 157.9, 154.1, 153.7, 134.6, 130.4, 124.8, 123.8, 121.2, 110.7, 79.3, 46.2, 41.6, 28.0 ppm.

3-Amino-5-(trifluoromethyl)benzonitrile (**53**):

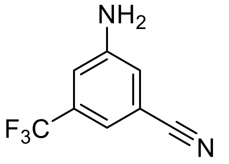

3-Nitro-5-(trifluoromethyl)benzonitrile **52** (2471 mg, 11.4 mmol, 1 eq.) and ammonium chloride (2446.0 mg, 46.4 mmol, 4 eq.) were dissolved and stirred in EtOH (25 ml) and water (12.5 mL). After heating to 90 °C for 30 minutes, 70 mesh >99% iron powder (2593.2 mg, 46.4 mmol, 4.1 eq) was added. TLC (20% EtOAc/PE) overnight showed full conversion of the starting material. The mixture was quenched with saturated bicarbonate solution (30 mL) and extracted with EtOAc (30 mL). The combined organic layers were washed with brine, dried over anhydrous $MgSO_4$, filtered, and concentrated *in vacuo*. The crude was purified by column chromatography 25% EtOAc in petroleum ether (PE) to obtain compound **53** as a yellow oil (1362 mg, 7.3 mmol, 64%).

^{1}H NMR (400 MHz, $CDCl_3$) δ = 7.24 (s, 1H), 7.08 (s, 1H), 7.05 (s, 1H), 4.15 (s, 2H) ppm.

^{13}C NMR (101 MHz, $CDCl_3$) δ = 147.5, 133.1, 122.9, 120.1, 117.9, 115.1, 113.9 ppm.

^{19}F NMR (376 MHz, $CDCl_3$) δ = -63.52 ppm.

3-(Aminomethyl)-5-(trifluoromethyl)aniline (**54**):

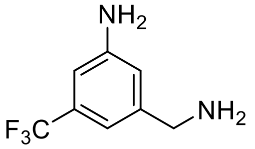

A solution of nitrile **53** (1340 mg, 7.20 mmol, 1 eq.) in THF (5 mL) was added dropwise to a solution of $LiAlH_4$ (15 mL, 36 mmol, 2.4 M) in THF (20 mL). After the addition was completed, the reaction mixture was heated to reflux and after 4 h quenched with slow addition of NaOH aq. (50 mL, 1M). A solution of Rochelle salt (500 mg) in water (10 mL) was added, and the mixture extracted with EtOAc (3 x 30 mL). The combined organic layers were washed with brine, dried over anhydrous $MgSO_4$, and filtered. The solvent was removed under reduced pressure to obtain compound **54** in quantitative yield (1577 mg, 8.29 mmol). The product was used without further purification.

^{1}H NMR (400 MHz, $CDCl_3$) δ = 6.94 (s, 1H), 6.84 (br s, 2H), 6.77 (s, 1H), 4.38 (s, 2H), 3.83 (s, 2H) ppm.

^{13}C NMR (101 MHz, $CDCl_3$) δ = 147.0, 145.1, 131.7, 124.1, 116.5, 113.6, 109.9, 46.0 ppm.

^{19}F NMR (376 MHz, $CDCl_3$) δ = -62.83 ppm.

tert-Butyl 4-(4-nitro-2-(trifluoromethyl)phenyl)piperazine-1-carboxylate (**58**):

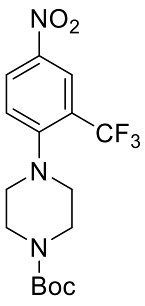

To a solution of 1-fluoro-4-nitro-2-(trifluoromethyl)benzene **56** (500 mg, 2.39 mmol, 1 eq.) in DMSO (5 mL), *tert*-butyl piperazine-1-carboxylate **33** (668 mg, 3.59 mmol, 1.5 eq.) and potassium carbonate (690 mg, 5.0 mmol, 2.1 eq) were added and the reaction mixture stirred for 18 h at 100 °C. Once completed, the reaction was quenched with water (10 mL) and extracted with EtOAc (3 x 30 mL). The combined organic layers were washed with brine, dried over anhydrous $MgSO_4$, filtered, and concentrated to dryness *in vacuo* to obtain a sticky solid (883 mg). Part of the crude (364 mg, 41%) was purified by column chromatography (25% EtOAc in PE) to obtain compound **10** as a yellow solid (187 mg, 0.50 mmol, 50%).

^{1}H NMR (400 MHz, $CDCl_3$) δ = 8.52 (d, J = 2.7 Hz, 1H), 8.35 (dd, J = 2.7, 9.0 Hz, 1H), 7.30 (d, J = 9.0 Hz, 1H), 3.60 (t, J = 5.1 Hz, 4H), 3.06 (t, J = 4.7 Hz, 4H), 1.49 (s, 9H) ppm.

^{13}C NMR (101 MHz, $CDCl_3$) δ = 157.0, 154.6, 142.7, 127.9, 125.2, 124.4, 122.8, 123.0, 80.1, 52.7, 44.1, 28.4 ppm.

^{19}F NMR (376 MHz, $CDCl_3$) δ = -60.23 ppm.

tert-Butyl 4-[3-nitro-5-(trifluoromethyl)phenyl]piperazine-1-carboxylate (**59**):

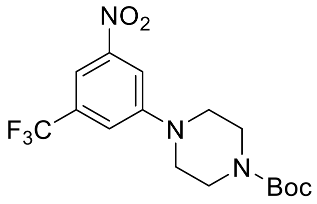

To a solution of 1-fluoro-3-nitro-5-(trifluoromethyl)benzene **57** (1506 mg, 7.2 mmol, 1 eq.) in DMSO (10 mL), *tert*-butyl piperazine-1-carboxylate **33** (1473 mg, 7.91 mmol, 1.1 eq.) and potassium carbonate (1995 mg, 14.4 mmol, 2 eq.) were added and the mixture was heated to 100 °C. After 20 h the reaction was complete and was quenched with water (50 mL). The mixture was extracted with EtOAc (3 x 50 mL) and the combined organic layers were washed with brine, dried over anhydrous $MgSO_4$, filtered, and concentrated to dryness *in vacuo*. The resulting crude was purified by column chromatography (10% EtOAc in PE) to obtain compound **59** (2001 mg, 5.33 mmol, 74%) as an orange powder.

^1H NMR (400 MHz, $CDCl_3$) δ = 7.90 (s, 1H), 7.86 (t, *J* = 2.1 Hz, 1H), 7.36 (s, 1H), 3.64 (br t, *J* = 3.1 Hz, 4H), 3.34 (br t, *J* = 5.5 Hz, 4H), 1.50 (s, 9H) ppm.

^{13}C NMR (101 MHz, $CDCl_3$) δ = 154.5, 151.9, 149.4, 143.2, 132.8, 123.0, 116.9, 112.3, 110.3, 80.4, 47.9, 41.9, 28.4 ppm.

^{19}F NMR (376 MHz, $CDCl_3$) δ = -63.11 ppm.

1-(5-Nitro-2-(trifluoromethyl)phenyl)piperazine (**61**):

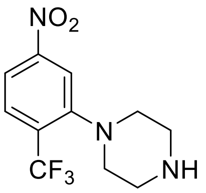

Piperazine (6211 mg, 72 mmol, 6 eq.) and K_2CO_3 (3340 mg, 24 mmol, 2 eq.) were suspended in DMSO (20 mL). To the stirring solution was added 2-fluoro-4-nitro-1-(trifluoromethyl)benzene **60** (2516.3 mg, 12 mmol, 1 eq.) and it was heated to 100 °C. After 24 h the reaction was complete, quenched with water (50 mL) and extracted with EtOAc (3 x 50 mL). The combined organic layers were washed with brine and saturated NH_4Cl, dried over anhydrous $MgSO_4$, filtered, and concentrated *in vacuo* to obtain compound **61** as an orange oil (3088 mg, 11.2 mmol, 93%).

^1H NMR (400 MHz, $CDCl_3$) δ = 8.14 (s, 1H), 8.02 (d, *J* = 8.6 Hz, 1H), 7.81 (d, *J* = 8.2 Hz, 1H), 3.03 (br s, 4H), 2.97 (br s, 4H) ppm.

^{13}C NMR (101 MHz, $CDCl_3$) δ = 154.1, 150.7, 132.1, 128.8, 123.0, 118.8, 118.6, 54.5, 46.0 ppm.

^{19}F NMR (376 MHz, $CDCl_3$) δ = -61.14 ppm.

tert-Butyl 4-[5-nitro-2-(trifluoromethyl)phenyl]piperazine-1-carboxylate (**62**):

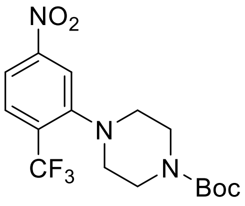

To a solution of di-*tert*-butyl dicarbonate (5409 mg, 24.8 mmol, 2.2 eq) in DCM (5 mL), compound **61** (3000 mg, 10.9 mmol, 1 eq.) as solution in DCM (10 mL) and DMAP (141 mg, 1.15 mmol, 0.1 eq.) were added and the mixture was . stirred for 72 h. The resulting crude was purified by column chromatography (15% EtOAc in PE) to obtain compound **62** as a yellow oil (3462 mg, 9.22 mmol, 85%).

^1H NMR (400 MHz, $CDCl_3$) δ = 8.12 (br s, 1H), 8.06 (br d, *J* = 8.6 Hz, 1H), 7.83 (dd, *J* = 3.1, 8.6 Hz, 1H), 3.59 (br d, *J* = 3.9 Hz, 4H), 2.94 (br d, *J* = 3.5 Hz, 4H), 1.48 (s, *J* = 3.5 Hz, 9H) ppm.

^{13}C NMR (101 MHz, $CDCl_3$) δ = 154.6, 153.6, 150.7, 132.5, 128.8, 122.9, 119.5, 118.8, 80.0, 53.3, 43.9, 28.4 ppm.

^{19}F NMR (376 MHz, $CDCl_3$) δ = -60.84 ppm.

tert-Butyl 2-(methylthio)-4,5-dihydro-1*H*-imidazole-1-carboxylate (**64**):

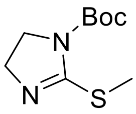

To a solution of 2-(methylthio)-4,5-dihydro-1*H*-imidazole **63** (2256 mg, 9.24 mmol, 1 eq.) in DCM (10mL) and triethylamine (2.6 mL, 18.5 mmol, 2 eq.), was added a solution of di-*tert*-butyl di-carbonate (2078 mg, 9.52 mmol, 1 eq.) in DCM (10mL). After 24 h the solvent was removed under reduced pressure, and the resulting crude was purified by flash column chromatography using 50% EtOAc in PE as eluent to obtain compound **63** as colourless crystals (1747 mg, 8.07 mmol, 87 %).

^1H NMR (400 MHz, $CDCl_3$) δ = 3.80-3.88 (m, 4H), 2.39 (s, 3H), 1.51 (s, 9H) ppm.

^{13}C NMR (101 MHz, $CDCl_3$) δ = 159.6, 150.8, 82.5, 53.4, 47.6, 28.2, 15.0 ppm.

P. falciparum culturing and *in vitro* drug sensitivity assay on asexual blood stage parasites

The *Plasmodium falciparum* NF54 wild-type parasites were cultured in RPMI 1640 medium that was supplemented with 25 mM HEPES, 0.36 mM hypoxanthine, 24 mM $NaHCO_3$ (pH = 7.3), 0.5% Albumax II and 100 µg/mL neomycin. The parasites were maintained at 37 °C with the mixed gas containing 3% O_2, 4% CO_2, and 93% N_2 and kept in an incubator under atmospheric pressure. The compounds were tested for *in vitro* drug sensitivity using the [^{3}H]-hypoxanthine incorporation assay as described.[7] Briefly, compounds were dissolved in DMSO at 10 mM and was diluted in hypoxanthine-free culture medium. The compounds were then titrated in duplicates over a 64-fold range in a 6-step twofold dilution in a 96-well plate. The parasites were then added (100µL) to each well and mixed with the compound to obtain a final parasitemia of 0.3% and haematocrit of 1.25%. The plates were then incubated for 48 h and 0.25 µCi of [^{3}H]-hypoxanthine was added per well and was incubated for an addition 24 h. The parasites were ten harvested on a glass-fiber filter using a Microbeta FilterMate cell harvester (Perkin Elmer, Waltham, US) and radioactivity was counted using a MicroBeta2 liquid scintillation counter (Perkin Elmer, Waltham, US). The results were recorded and expressed as percentage of untreated controls. The fifty percent inhibitory concentration (IC_{50}) was estimated by linear interpolation as described.[8]

Determination of *in vitro* antibacterial activity

Minimal inhibitory concentration (MIC) values were determined in 96-well plates (Sarstedt, Nümbrecht, Germany) against, *Escherichia coli ΔtolC, E. coli* K12, *Acinetobacter baumannii*, *Pseudomonas aeruginosa* PA14, *Staphylococcus aureus ssp. Aureus*, and *Streptococcus pneumoniae* (DSM20566). As bacteria start OD_{600} 0.03 was used in a total volume of 200 µL in lysogeny broth (LB) medium containing the compounds dissolved in DMSO (DMSO concentration in the experiment: 1%). Final compound concentrations (in duplicates) were prepared by serial dilution ranging from 0.02–100 µg/mL depending on their antibacterial activity and solubility in growth medium. The ODs were measured using a CLARIOstar platereader (BMG labtech, Ortenberg, Germany) after inoculation and after incubation for 18 h at 37 °C with 50 rpm (200 rpm for *P. aeruginosa* PA14). Given MIC values are means of at least two independent determinations and defined as the lowest concentration of compound that reduced the OD_{600} by ≥ 95%.

Determination of *in vitro* anti-tubercular activity and solubility in 7H9 medium

Anti-tubercular tests were performed as previously described.[9] In brief, 7H9 complete medium (BD Difco; Becton Dickinson, Maryland,USA) supplemented with 10% OADC (BD), 0.2% glycerol, and 0.05% Tween80 as previously described,[10] was used to culture *Mycobacterium tuberculosis* (*Mtb*) strain H37Rv (ATCC 25618) carrying a mCherry-expressing plasmid (pCherry10).[11] Cultures were harvested at mid-log phase and frozen in aliquots at –80 °C. Prior to testing aliquots were thawed followed by centrifugation and the pellet was resuspended in 7H9 medium with 10% OADC (without glycerol and Tween80). This was further thoroughly resuspended by passing it through a syringe with a 26-gauge needle to avoid clumping of the bacteria. 2×105 CFU (colony forming units) were then cultured in a total volume of 100 µl culture medium (triplicates) to test the non-precipitating compounds for the anti-tubercular activity at the concentrations indicated. For these assays, 96-well flat clear bottom black polystyrene microplates (Corning® CellBIND®, Merck, New York, USA) were used. Each plate had Rifampicin (at 1 µg/ml and 0.1 µg/ml) (National Reference Center, Borstel) as a reference compound. Plates were sealed with an air-permeable membrane (Porvair Sciences, Wrexham, UK) in a 37 °C incubator with mild agitation (TiMix5, Edmund Bühler, Germany). The activity of compounds was determined after 7 days by measuring the bacterial growth as relative light units (RLU) from the fluorescence intensity obtained at an excitation wavelength of 575 nm and an emission wavelength of 635 nm in a microplate reader (Synergy 2, BioTek Instruments, Vermont, USA). Two independent experiments (each in triplicates) were performed, and all values were normalized to untreated control sample (100%) in each experiment.

Cytotoxicity assay

To obtain information regarding the toxicity of our compounds, their impact on the viability of human cells was investigated. HepG2 cells (2x104 cells per well) were seeded in 96-well, flat-bottomed culture plates in 100 µL culture medium (DMEM containing 10% fetal calve serum, 1% penicillin-streptomycin). Twenty-four hours after seeding the cells, medium was removed and replaced by medium containing test compounds in a final DMSO concentration of 1%. Compounds were tested in duplicates at a single concentration or, for CC_{50} determination, at 8 concentrations that were prepared via 2-fold serial dilutions in 1% DMSO/medium. Epirubicin and doxorubicin were used as positive controls in serial dilutions starting

from 10 µM, and rifampicin was used as a negative control (at 100 µM). The living cell mass was determined 48 h after treatment with compounds by adding 0.1 volumes of 3-(4,5-dimethylthiazol-2-yl)-2,5-diphenyltetrazolium bromide (MTT) solution (5 mg/mL sterile PBS) (Sigma, St. Louis, MO) to the wells. After incubating the cells for 30 min at 37 °C (atmosphere containing 5% CO_2), medium was removed and MTT crystals were dissolved in 75 µL of a solution containing 10% SDS and 0.5% acetic acid in DMSO. The optical density (OD) of the samples was determined photometrically at 570 nm in a PHERAstar Omega plate reader (BMG labtech, Ortenberg, Germany). To obtain percent viability for each sample, their ODs were related to those of DMSO controls. At least two independent measurements were performed for each compound. The calculation of CC_{50} was performed using the nonlinear regression function of GraphPad Prism 10 (GraphPad Software, San Diego, CA, USA).

Measurement of *in vitro* cytotoxicity by XTT assay

An 2,3-bis-(2-methoxy-4-nitro-5-sulfophenyl)-2*H*-tetrazolium-5-carboxanilide (XTT) assay was used to determine the cytotoxicity of the selected compounds (Table S2). Human Monocyte-derived Macrophages (hMdM) were differentiated from peripheral blood mononuclear cells (PBMC) of healthy volunteers and cultured as previously described.[12] 5×104 cells/well in RPMI medium containing 10% (*v/v*) heat-inactivated Fetal Bovine Serum and 2 mmol/L l-glutamine (Biochrom, Berlin, Germany) were seeded in presence of 2-fold dilution concentrations (64 µM to 1 µM) of the compound for 24 h with a final volume of 200 µL/well. For this assay, 96-well clear flat bottom plates (Nunclon™ Delta Surface, ThermoScientific, Denmark) were used. Triton™-X 100 (Sigma-Aldrich, Missouri, USA; 1% in RPMI medium) was used as a positive control. Cells were incubated with 200 µl of Triton-X 100 and incubated for 10 min at 37 °C. XTT dye from the kit (SERVA Electrophoresis GmbH; 50 µL) was added to each well and resuspended thoroughly and further incubated for 3–4 hours at 37 °C. Subsequently, absorbance values were measured at 490 nm on a multi-well plate reader (Synergy 2, BioTek Instruments, Vermont, USA). Untreated cells were used as negative control. The Cytotoxic Concentration 50 (CC_{50}) was determined by plotting a curve using GraphPad Prism version 9.4.1.

Supplementary Tables and Figures

Computational evaluation of pKa

All pKa values were computationally determined using ACD/ Percepta.

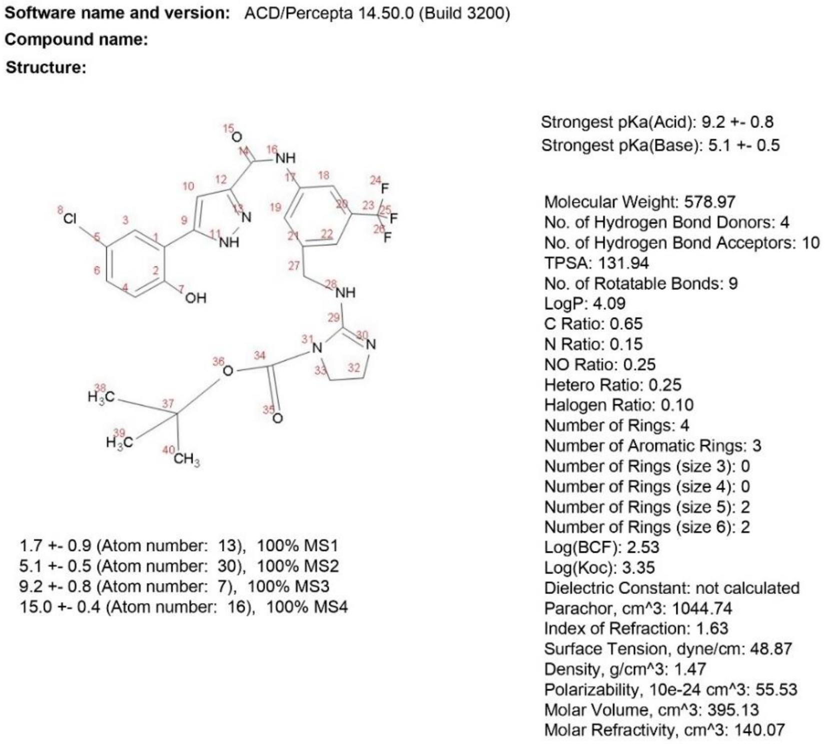

Figure S1. Computational evaluation of pKa of **9D**.

Biological evaluation of compounds

Table S1. Biological activity of pyrazole-amide class in *Escherichia coli* (*EcΔtolC* and *EcK*12), *Acinetobacter baumannii (Ab), Pseudomonas aeruginosa* (PA14) including standard deviation.

Cmp	*EcΔtolC* MIC_{95} (µM)	*EcΔtolC* inh. at 50 µM (%)	EcK12 inh. at 50 µM (%)	*Ab* inh. at 50 µM (%)	PA14 inh. at 50 µM (%)
**1a**	-	<10	<10	<10	<10
**1b**	-	sol. issues	n.d.	n.d.	n.d.
**1c**	-	sol. issues	n.d.	n.d.	n.d.
**1d**	-	<10	n.d.	n.d.	n.d.
**1e**	-	23 ± 3	n.d.	n.d.	n.d.
**2**	-	24 ± 12	n.d.	n.d.	n.d.
**3A**	45 ± 1	-	28 ± 12	21 ± 2	50 ± 4
**3B**	-	sol. issues	<10	n.d.	n.d.
**4A**	40 ± 8	-	34± 5	24.2 ± 7.7	62 ± 3
**4B**	-	sol. issues	17 ± 13	n.d.	n.d.
**5A**	21 ± 2	-	83 ± 8	34.3 ± 0.4	60 ± 3
**5B**	-	19 ± 14	<10	n.d.	n.d.
**5G**	11 ± 2	-	32± 9	<10	31 ± 14
**6A**	22.5 ± 0.0	-	49 ± 6	37 ± 2	63 ± 9
**6B**	-	<10	n.d.	n.d.	n.d.
**6G**	9 ± 3	-	45 ± 5	32 ± 6	55 ± 16
**7A**	47 ± 1	-	27 ± 10	15 ± 3	44 ± 6
**7B**	-	<10	<10	n.d.	n.d.
**7C**	13 ± 3	-	29.4 ± 0.6	33 ± 6	41 ± 19
**7D**	14 ± 4	-	56 ± 2	77 ± 4	54.8 ± 0.8
**7G**	13 ± 3	-	61 ± 6	24 ± 5	56.0 ± 0.0
**8A**	-	44 ± 16	n.d.	n.d.	n.d.
**8B**	-	<10	n.d.	n.d.	n.d.
**8C**	21.5 ± 0.0	-	<10	12 ± 9	10 ± 3
**8D**	-	18.5 ± 0.8	n.d.	n.d.	n.d.
**8G**	47.5 ± 0.0	-	29 ± 11	19.9 ± 0.6	41 ± 6
**9A**	8 ± 3	-	<10	MIC95 = 48.9 ± 0.5	<10
**9B**	-	48 ± 8	n.d.	n.d.	n.d.
**9C**	7 ± 1	-	84 ± 2	47 ± 4	18 ± 21
**9D**	24.0 ± 0.0	-	<10	81.6 ± 0.6	21± 18
**9G**	5 ± 2	-	MIC95 = 46 ± 2	59 ± 8	50 ± 4
**10A**	22.9 ± 0.1	-	61± 17	86 ± 7	<10
**10B**	-	<10	<10	<10	n.d.
**10C**	5.5 ± 0.8	-	77 ± 10	49.6 ± 0.9	29 ± 12
**10D**	18 ± 6	-	17 ± 6	<10	<10
**10G**	3.5 ± 0.8	-	86 ± 2	49 ± 24	55 ± 2
**11A**	7 ± 2	-	72 ± 5	MIC95 = 22 ± 3	<10
**11B**	-	18 ± 4	n.d.	<10	n.d.
**11C**	7 ± 2	-	63 ± 3	53 ± 12	<10
**11G**	4 ± 1	-	MIC95 = 48 ± 5	MIC95 = 17 ± 5	25 ± 10
**12A**	18.9 ± 0.5	-	12 ± 2	29 ± 1	<10
**12B**	-	<10	n.d.	<10	n.d.
**12G**	2.8 ± 0.3	-	51± 6	33 ± 5	<10
**13A**	-	46 ± 5	n.d.	<10	<10
**13B**	-	<10	n.d.	n.d.	n.d.
**13G**	5 ± 2	-	59 ± 8	46 ± 26	17 ± 10
**14A**	-	18 ± 4	n.d.	n.d.	n.d.
**14G**	-	18 ± 9	n.d.	n.d.	n.d.

n.d. = not determined

Table S2. Biological activity of pyrazole-amide class in *Plasmodium falciparum* (*Pf*NF54), *Streptococcus pneumoniae* (*Sp*), *Staphylococcus aureus (Sa),* human liver cells (HepG2) including standard deviation. In addition, for selected compounds; human Monocyte-derived Macrophages (hMdM) toxicity, and solubility in 7h9 media.

Cmp	*Pf*NF54 IC_{50}	*Sp* MIC_{95} (μM)	*Sa* MIC_{95} (μM)	HepG2 CC_{50} (μM)	hMdM CC_{90} (μM)	7h9 media Sol. (μM)
**1a**	0.21 ± 0.02	>50	>50	>50	-	<8
**1b**	0.7 ± 0.2	>50	>50	n.d.	-	<8
**1c**	0.51 ± 0.05	>50	>50	n.d.	-	32
**1d**	2.40 ± 0.03	>50	>50	>50	-	-
**1e**	>5	>50	>50	>50	-	-
**2**	1.1 ± 0.2	>50	>50	49 ± 7%	-	16
**3A**	0.62 ± 0.03	40 ± 7	>50	12 ± 2	-	64
**3B**	1.0 ± 0.3	>50	>50	25 ± 4	-	-
**4A**	0.27 ± 0.03	40 ± 7	>50	13 ± 3	-	64
**4B**	0.2 ± 0.1	>50	>50	7.4 ± 0.7	-	
**5A**	0.13 ± 0.03	26 ± 1	37 ± 10	9 ± 2	-	64
**5B**	0.9 ± 0.3	>50	>50	>50	-	-
**5G**	0.93 ± 0.02	48 ± 1	23.1 ± 0.5	>50	-	-
**6A**	0.14 ± 0.02	45 ± 0	>50	11.8 ± 0.9	-	16
**6B**	1.61 ± 0.01	>50	>50	>50	-	-
**6G**	0.44 ± 0.06	25 ± 2	26 ± 2	>50	-	-
**7A**	0.30 ± 0.08	43 ± 11	>50	28.4 ± 0.2	-	64
**7B**	1.1 ± 0.3	>50	>50	>50	-	-
**7C**	0.39 ± 0.01	48.2 ± 0.8	22.3 ± 0.6	>50	-	-
**7D**	0.36 ± 0.07	31 ± 11	24.0 ± 0.0	19 ± 4	-	-
**7G**	0.21 ± 0.02	49.0 ± 0.0	22 ± 2	>50	-	-
**8A**	1.8 ± 0.5	>50	>50	30 ± 3	-	16
**8B**	1.7 ± 0.3	>50	>50	>50	-	<8
**8C**	0.67 ± 0.02	>50	49 ± 1	>50	-	-
**8D**	>5	>50	>50	>50	-	-
**8G**	0.42 ± 0.02	>50	22 ± 1	>50	-	-
**9A**	0.082 ± 0.001	10.8 ± 2.5	12.1 ± 0.6	7 ± 2	-	-
**9B**	0.2033 ± 0.0003	30 ± 5	>50	5.0 ± 0.8	-	-
**9C**	0.517 ± 0.004	15 ± 4	9 ± 2	>50	-	-
**9D**	0.15 ± 0.02	21 ± 9	12 ± 3	14 ± 2	-	-
**9G**	0.078 ± 0.003	>50	8 ± 3	>50	-	-
**10A**	0.15 ± 0.02	23 ± 3	29 ± 3	13 ± 4	-	-
**10B**	0.19 ± 0.01	>50	>50	>50	-	-
**10C**	0.404 ± 0.002	14 ± 2	8 ± 2	>50	-	-
**10D**	0.14 ± 0.04	>50	11.6 ± 0.2	11 ± 3	-	-
**10G**	0.25 ± 0.09	16 ± 5	5 ± 1	>50	>32	-
**11A**	0.05 ± 0.01	5 ± 1	6 ± 2	9 ± 3	-	-
**11B**	0.13 ± 0.06	>50	>50	4.0 ± 0.4	-	<8
**11C**	0.59 ± 0.08	7 ± 2	8 ± 2	>50	-	-
**11G**	0.5 ± 0.2	28 ± 15	3.2 ± 0.1	>25	>32	-
**12A**	0.06 ± 0.01	8 ± 2	14 ± 2	6 ± 1	-	-
**12B**	0.56 ± 0.06	>50	>50	>50	-	<8
**12G**	0.2 ± 0.1	31 ± 6	2.4 ± 0.3	30 ± 1	32	-
**13A**	0.160 ± 0.008	29 ± 2	>50	8 ± 3	-	-
**13B**	0.3418 ± 0.0006	n.d.	n.d.	2.8 ± 0.7	-	-
**13G**	0.5 ± 0.2	16 ± 6	2.5 ± 0.3	>25	>32	-
**14A**	3.3 ± 0.6	>50	>50	>50	-	-
**14G**	>5	>50	>50	>50	-	-

NMR, HRMS, and LCMS spectra

Compounds **1a–e**

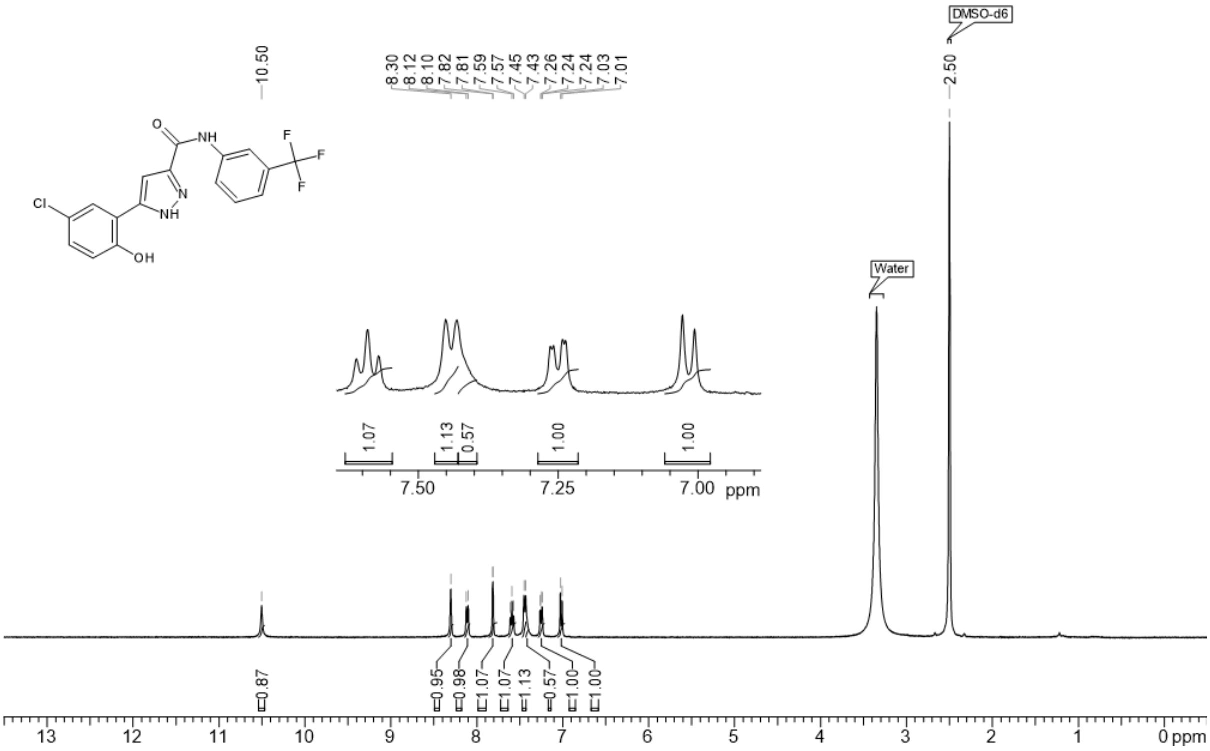

Figure S2. ^{1}H NMR spectrum of **1a**, pyrazole C–H overlap with 7.44 ppm (d).

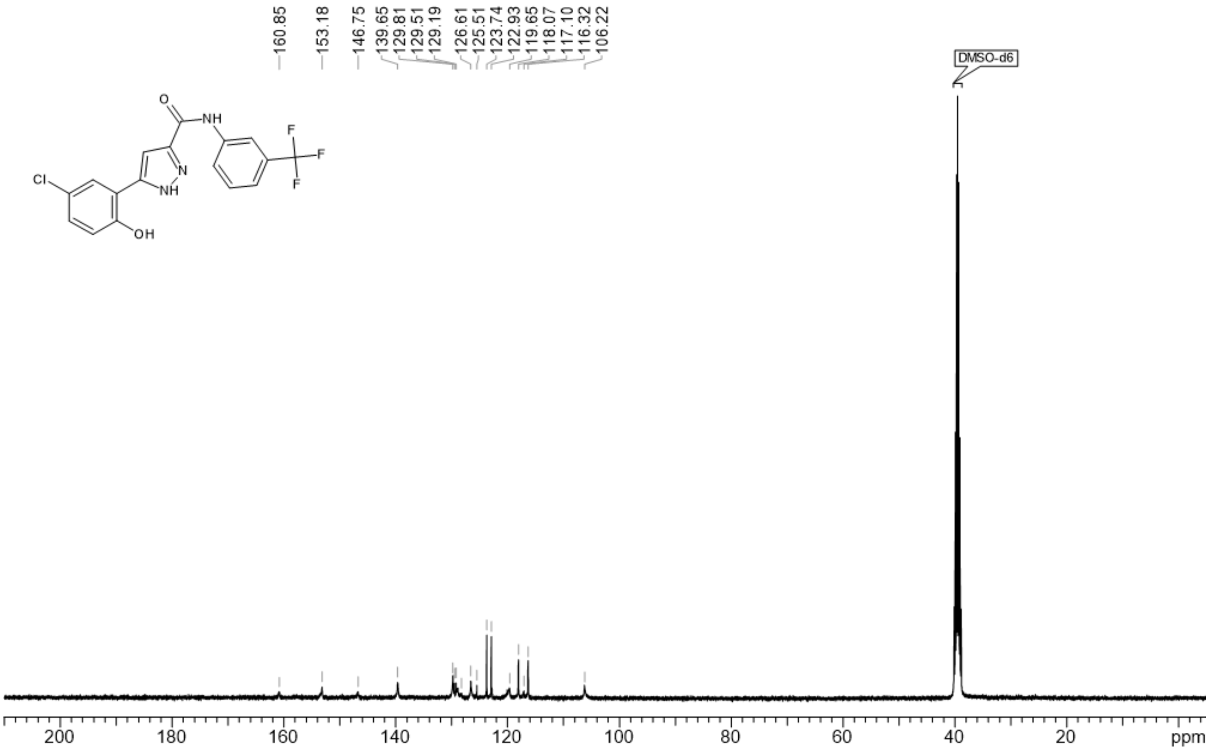

Figure S3. ^{13}C NMR spectrum of **1a**.

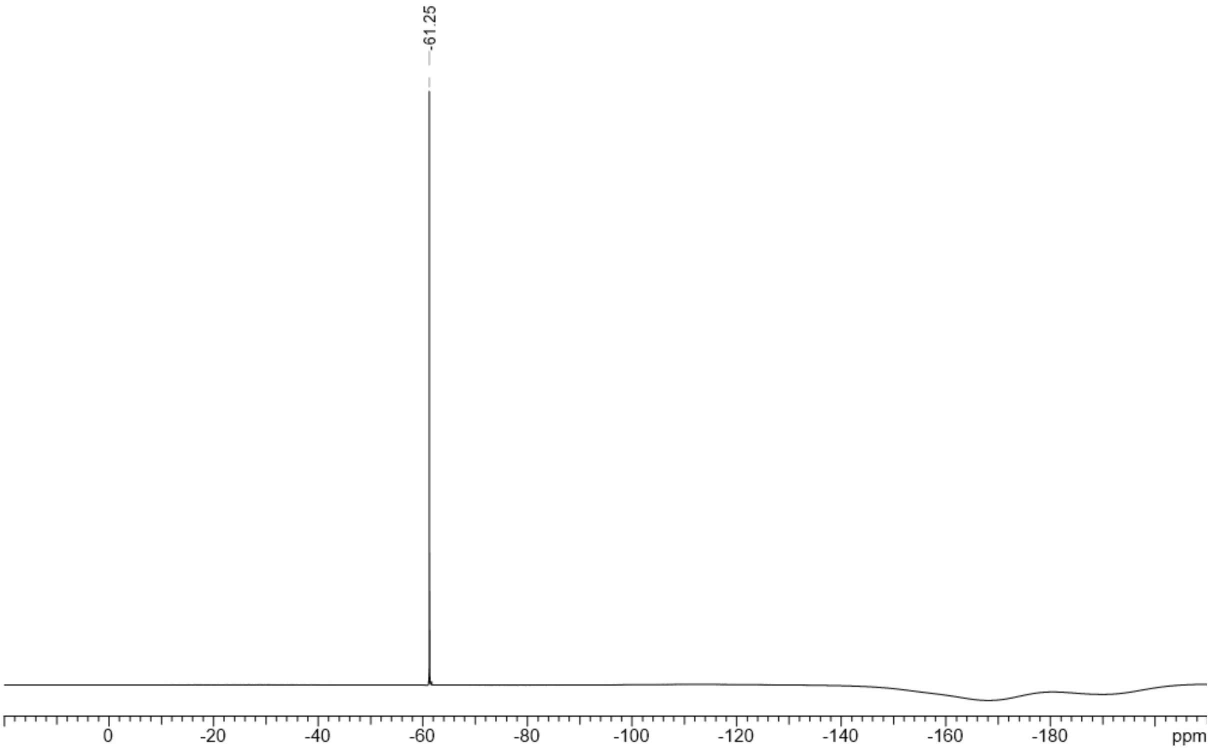

Figure S4. ^{19}F NMR spectrum of **1a**.

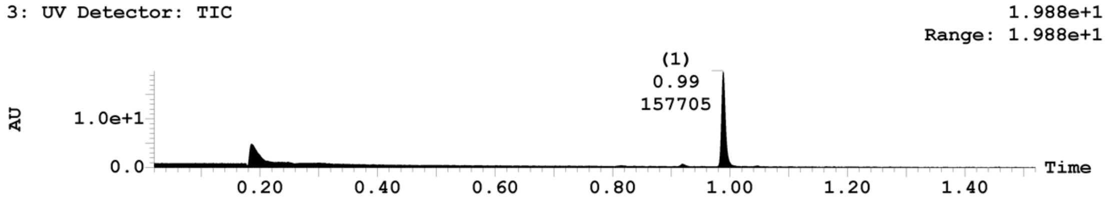

Peak Number	Time	Area %Total
1	0.99	100.00

Figure S5. LCMS purity analysis of **1a**.

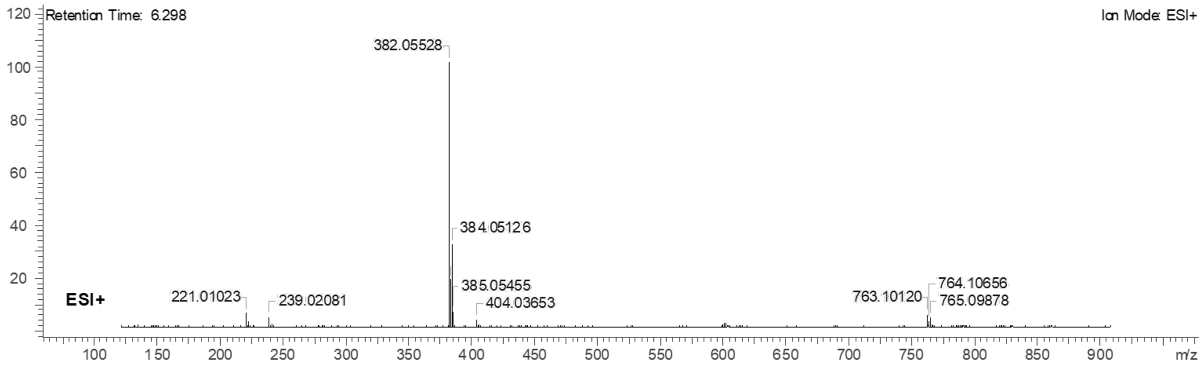

Figure S6. HRMS of **1a**.

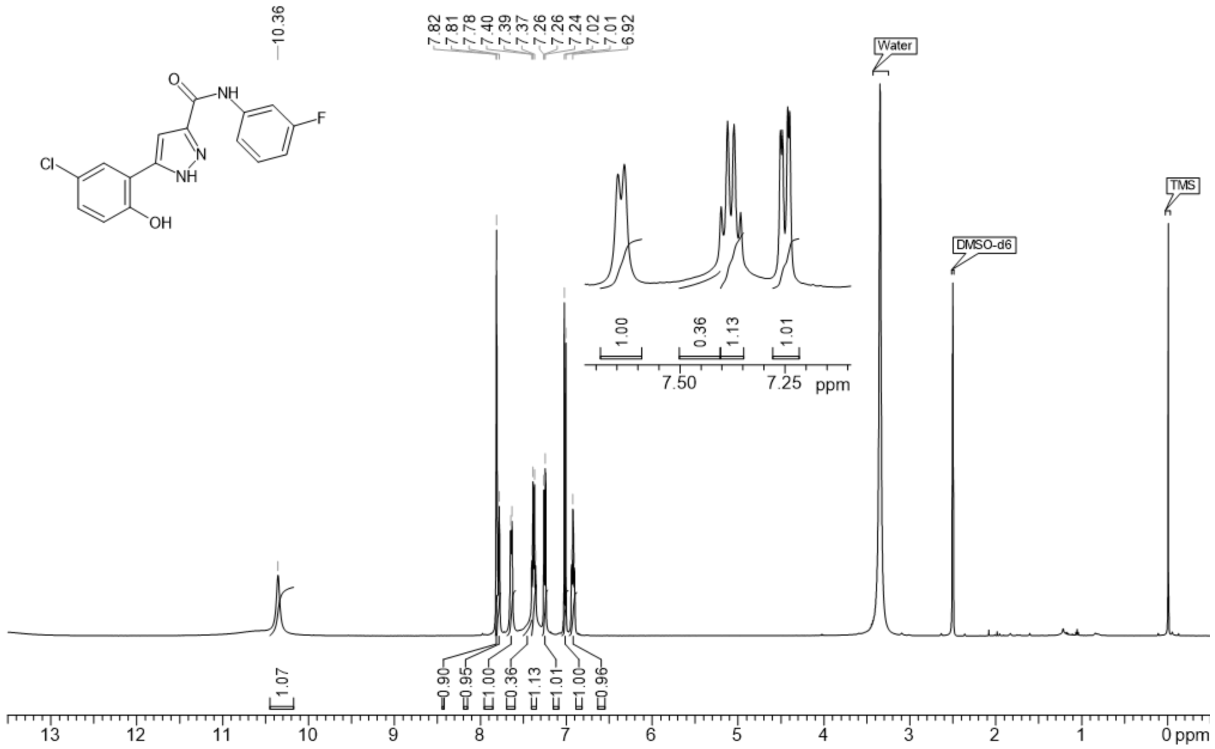

Figure S7. ^{1}H NMR spectrum of **1b**.

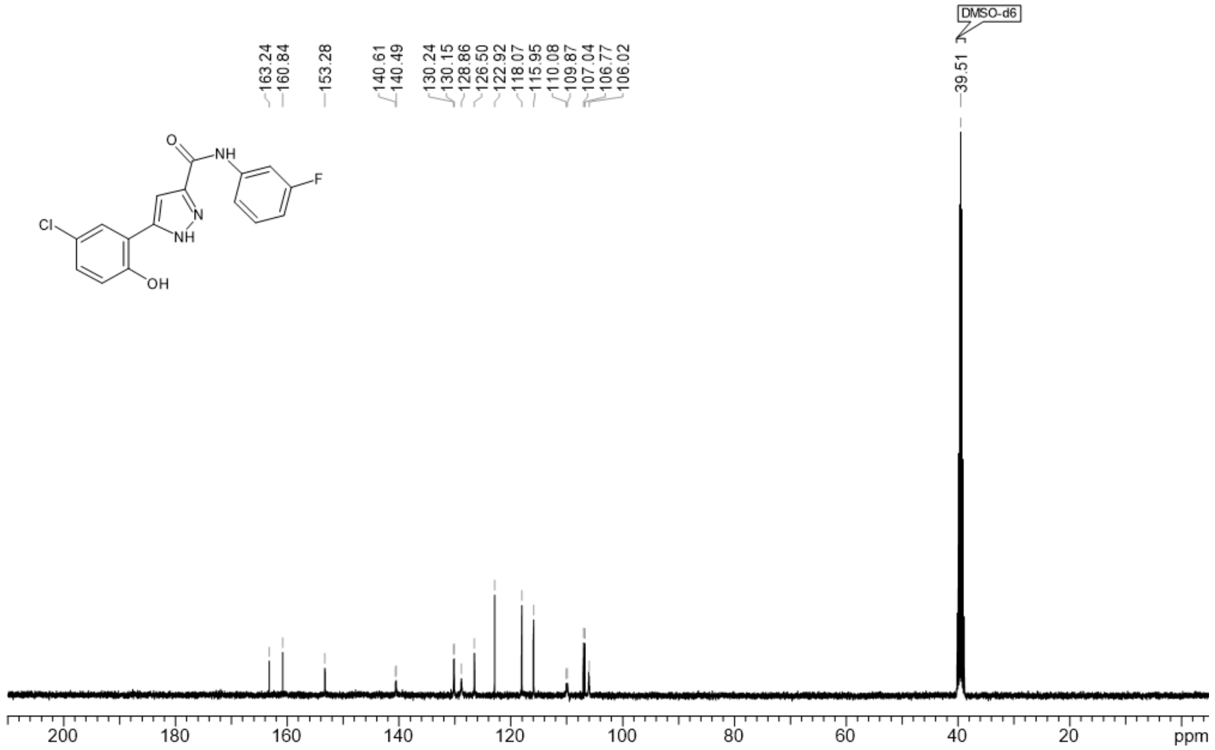

Figure S8. ^{13}C NMR spectrum at of **1b**.

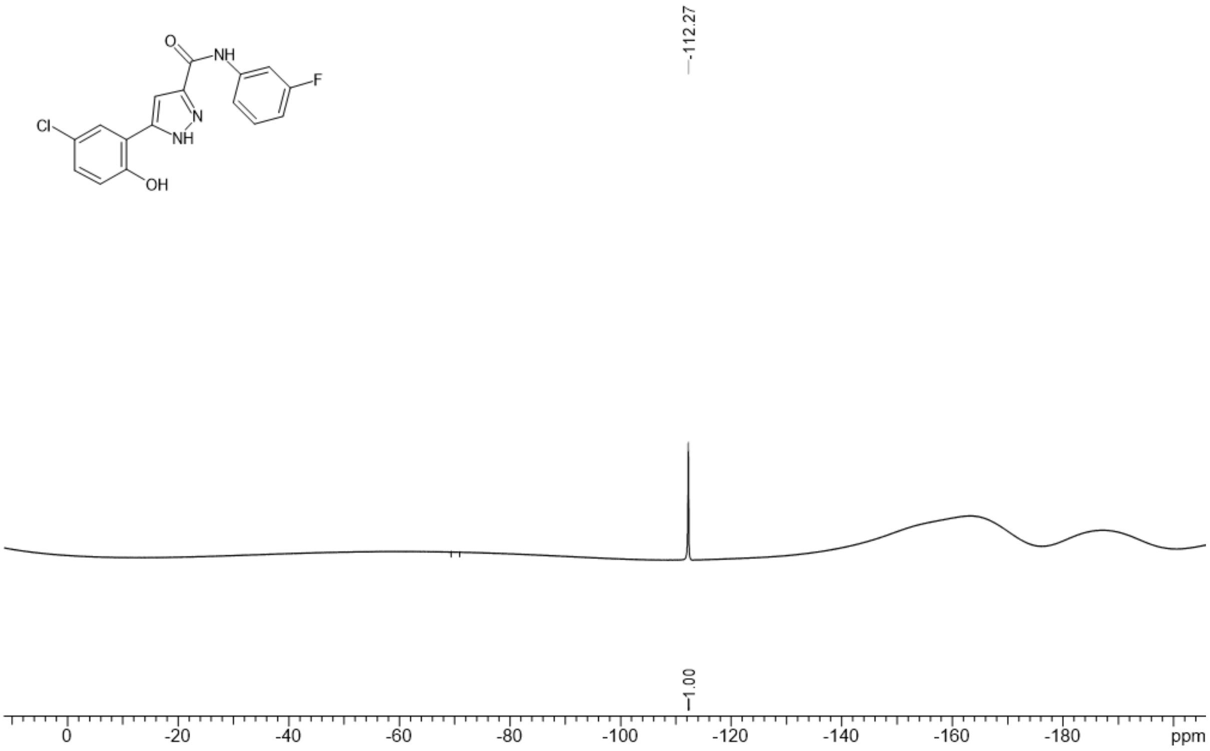

Figure S9. ^{19}F NMR spectrum of **1b**.

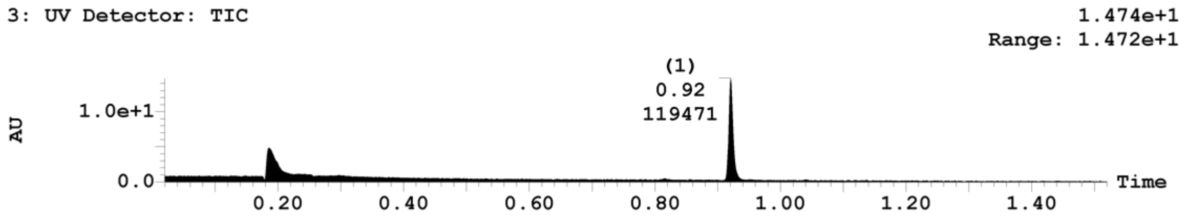

Peak Number	Time	Area %Total
1	0.92	100.00

Figure S10. LCMS purity analysis of **1b**.

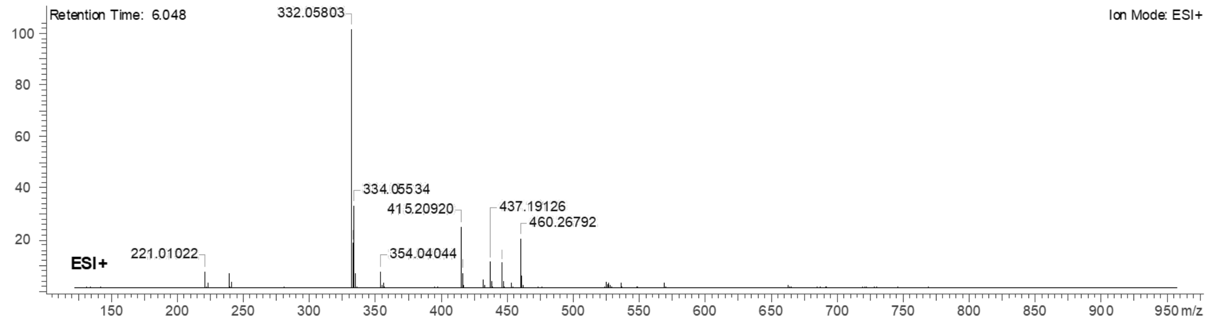

Figure S11. HRMS of **1b**.

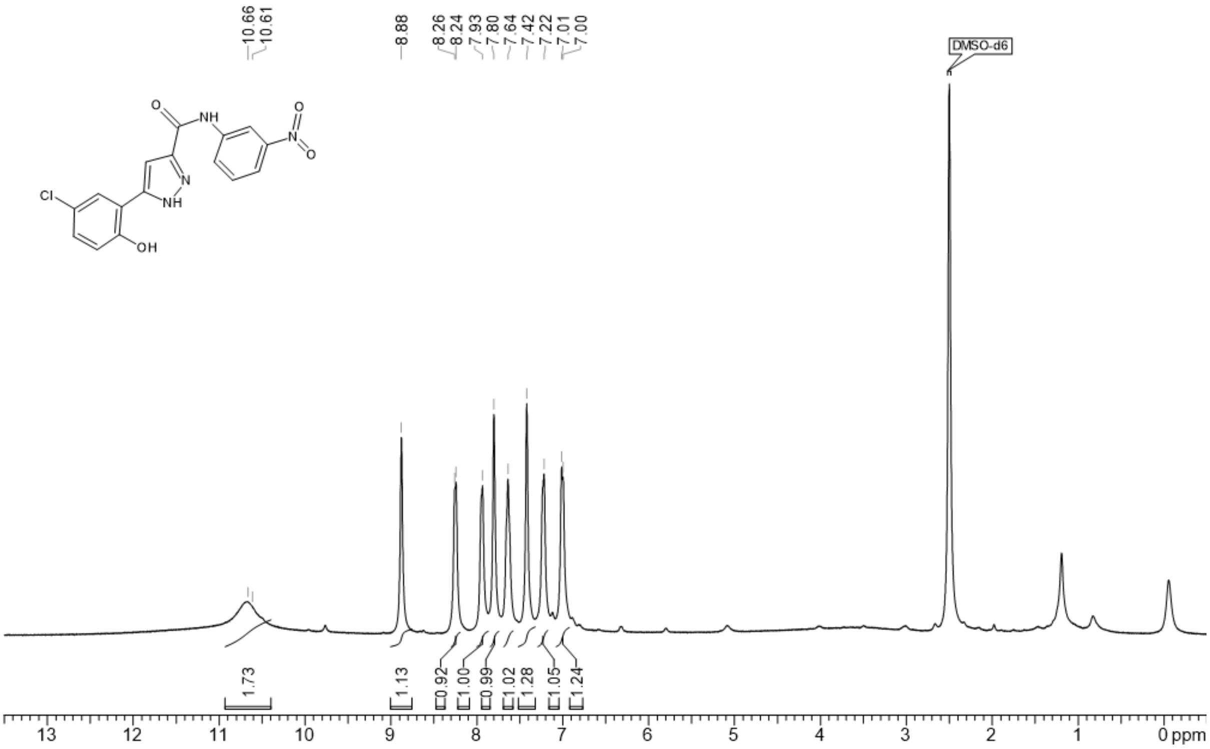

Figure S12. ^{1}H NMR spectrum of **1c**.

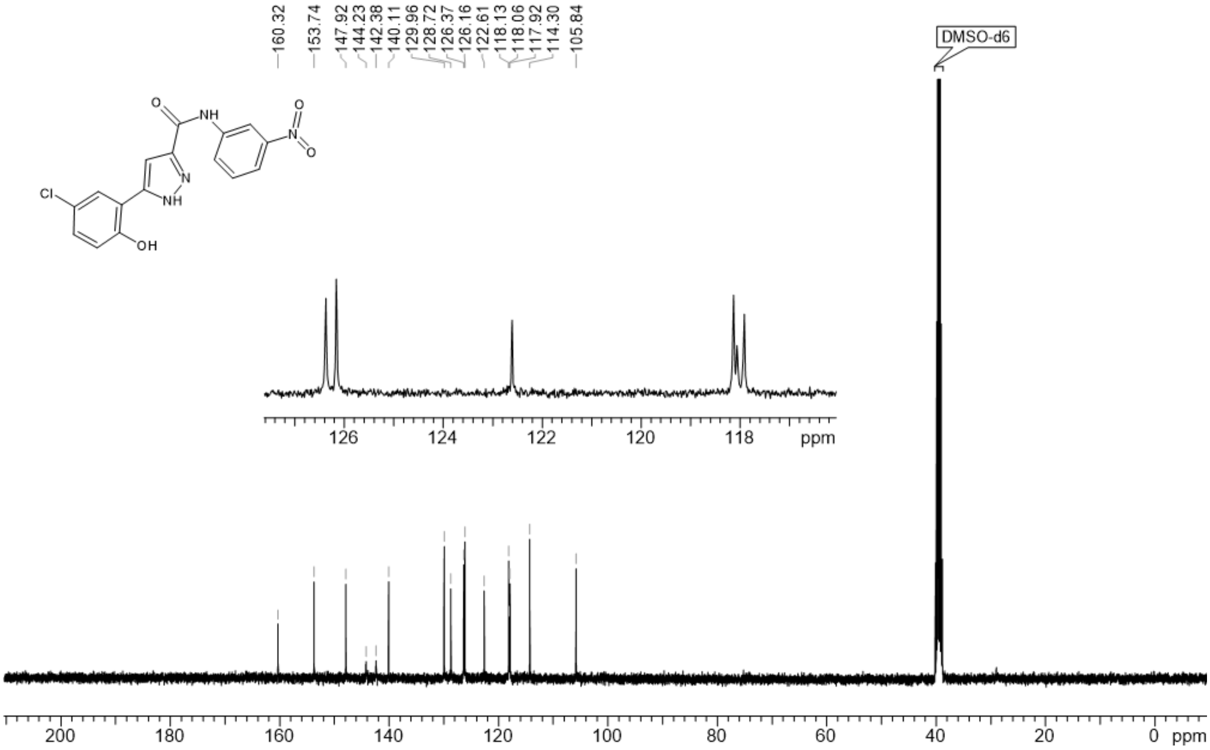

Figure S13. ^{13}C NMR spectrum of **1c**.

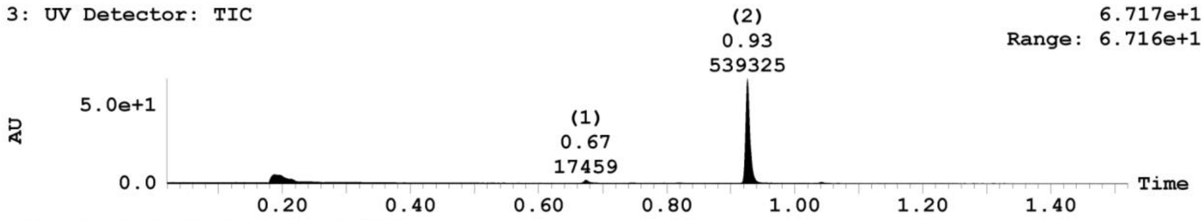

Peak Number	Time	Area %Total
1	0.67	3.14
2	0.93	96.86

Figure S14. LCMS purity analysis of **1c**.

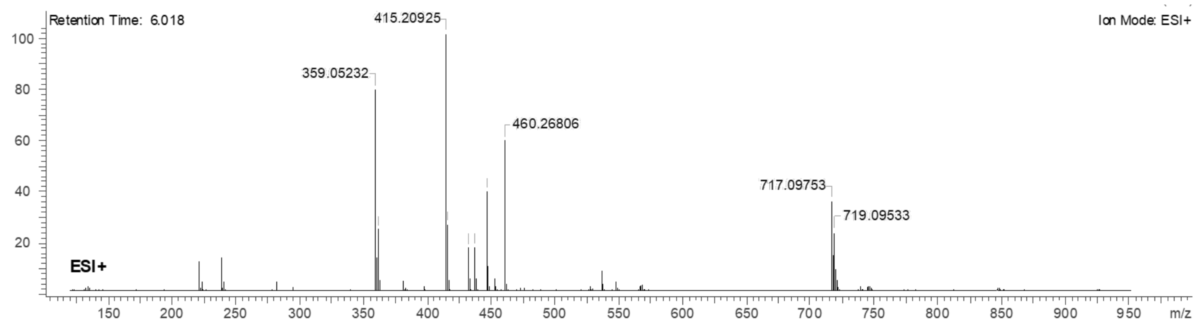

Figure S15. HRMS of **1c**.

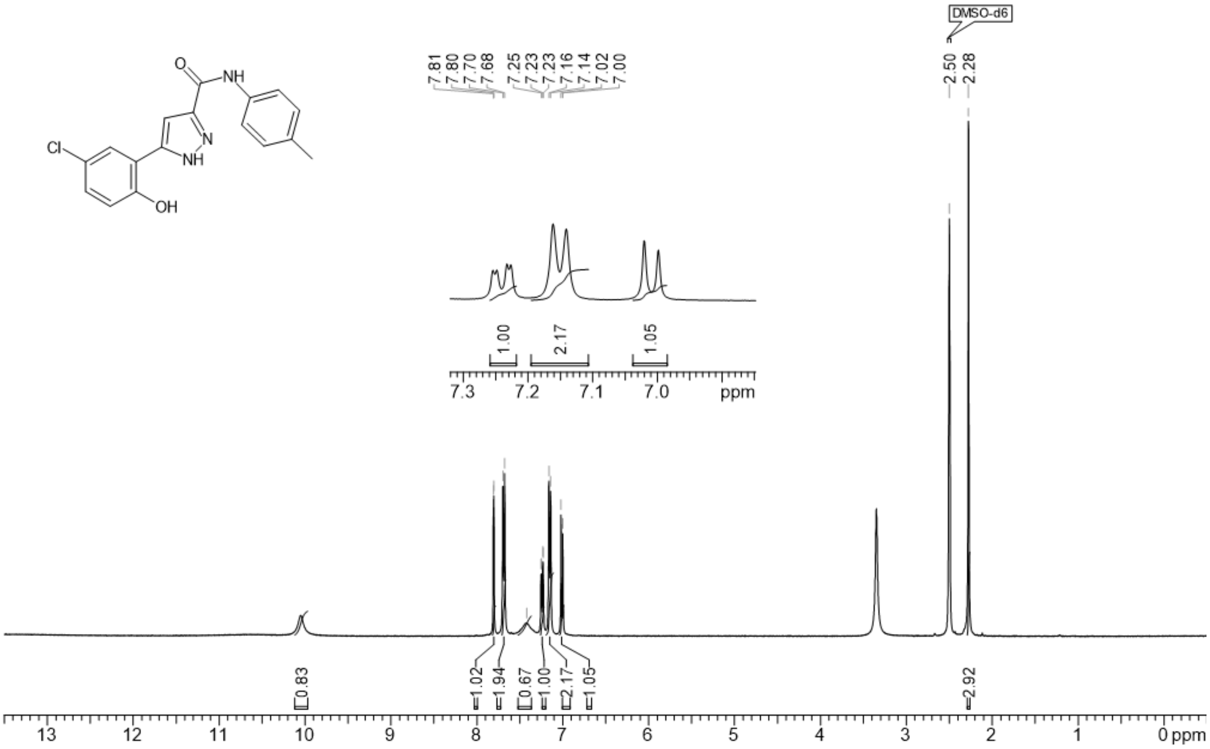

Figure S16. ^{1}H NMR spectrum of **1d**.

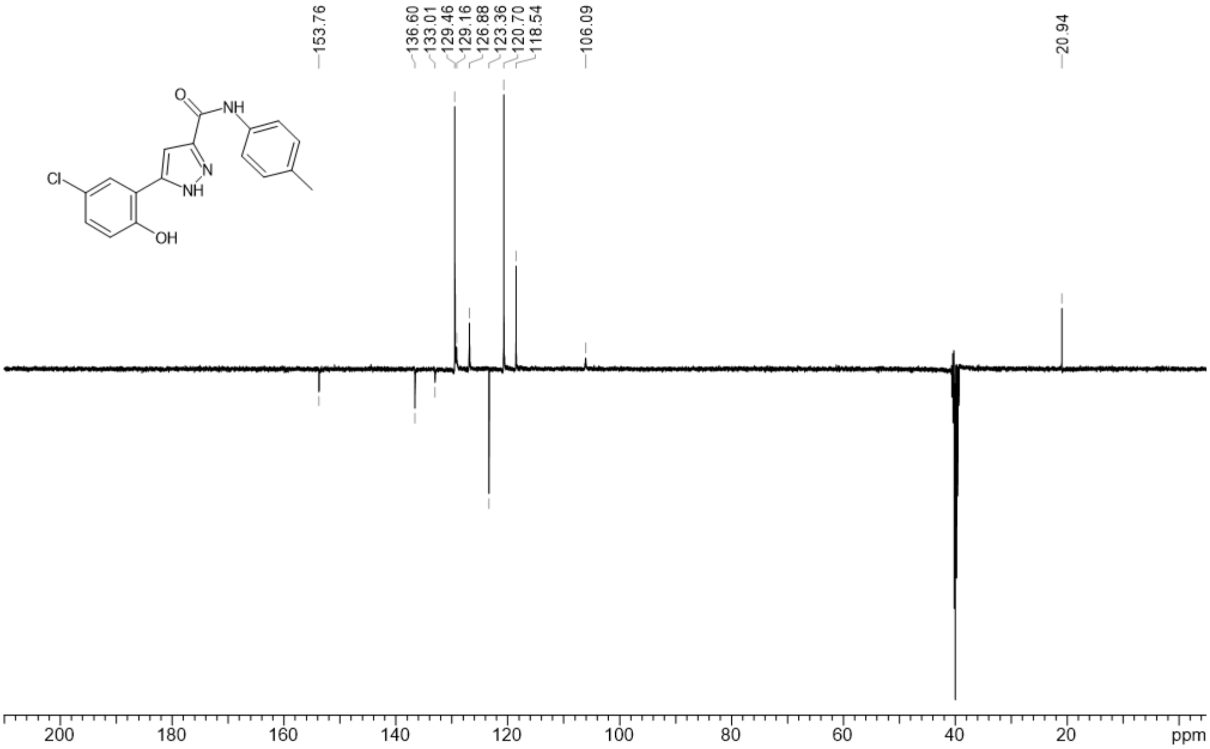

Figure S17. ^{13}C APT NMR spectrum of **1d**.

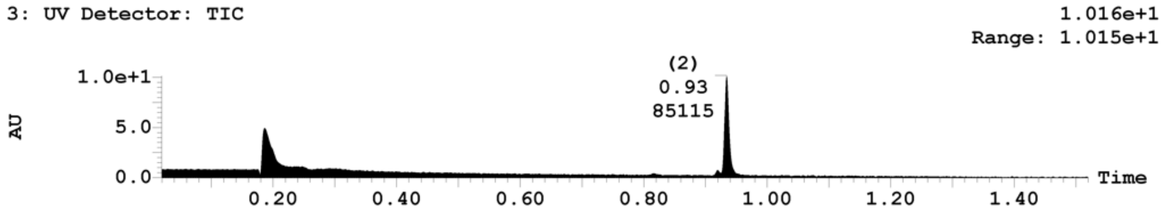

Peak Number	Time	Area %Total
1	0.92	4.49
2	0.93	95.51

Figure S18. LCMS purity analysis of **1d**.

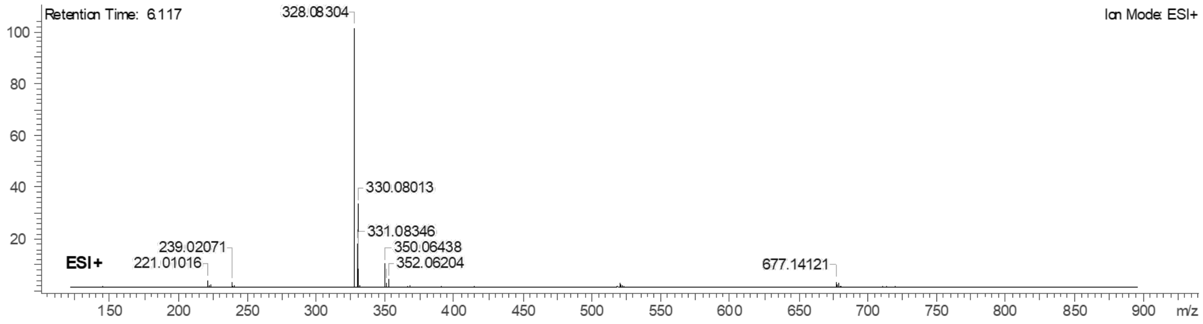

Figure S19. HRMS of **1d**.

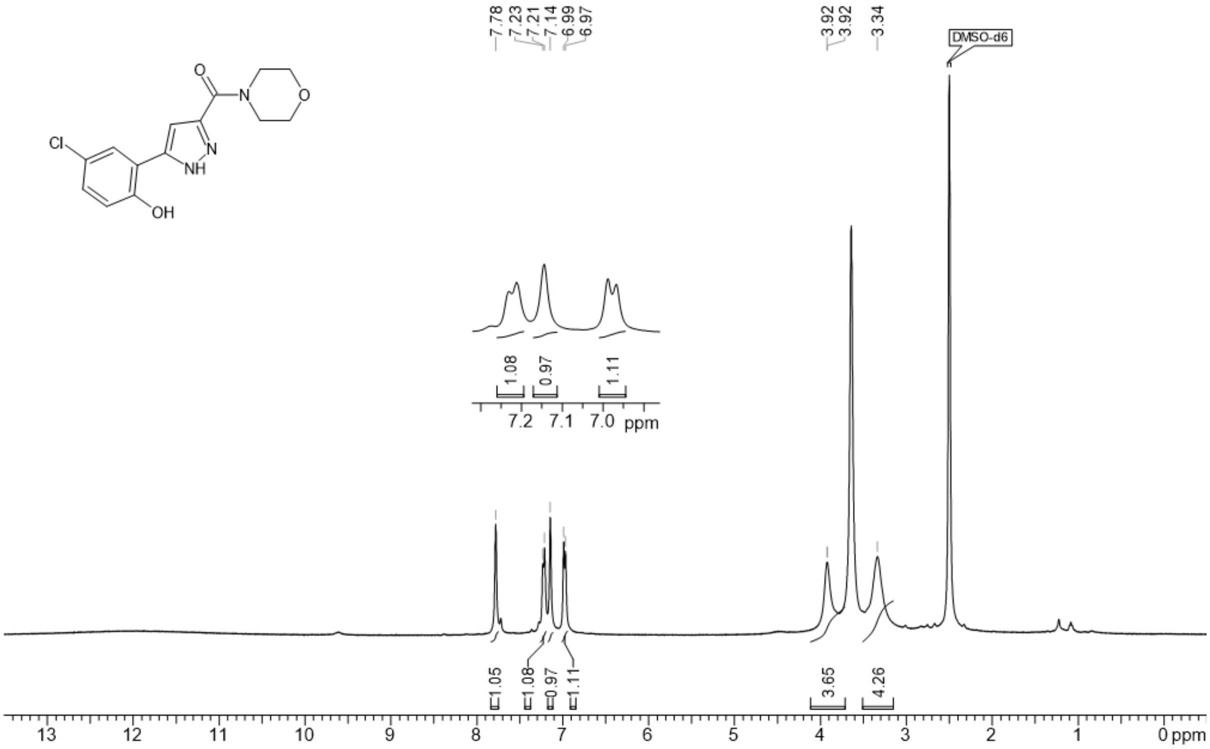

Figure S20. ^{1}H NMR spectrum of **1e**.

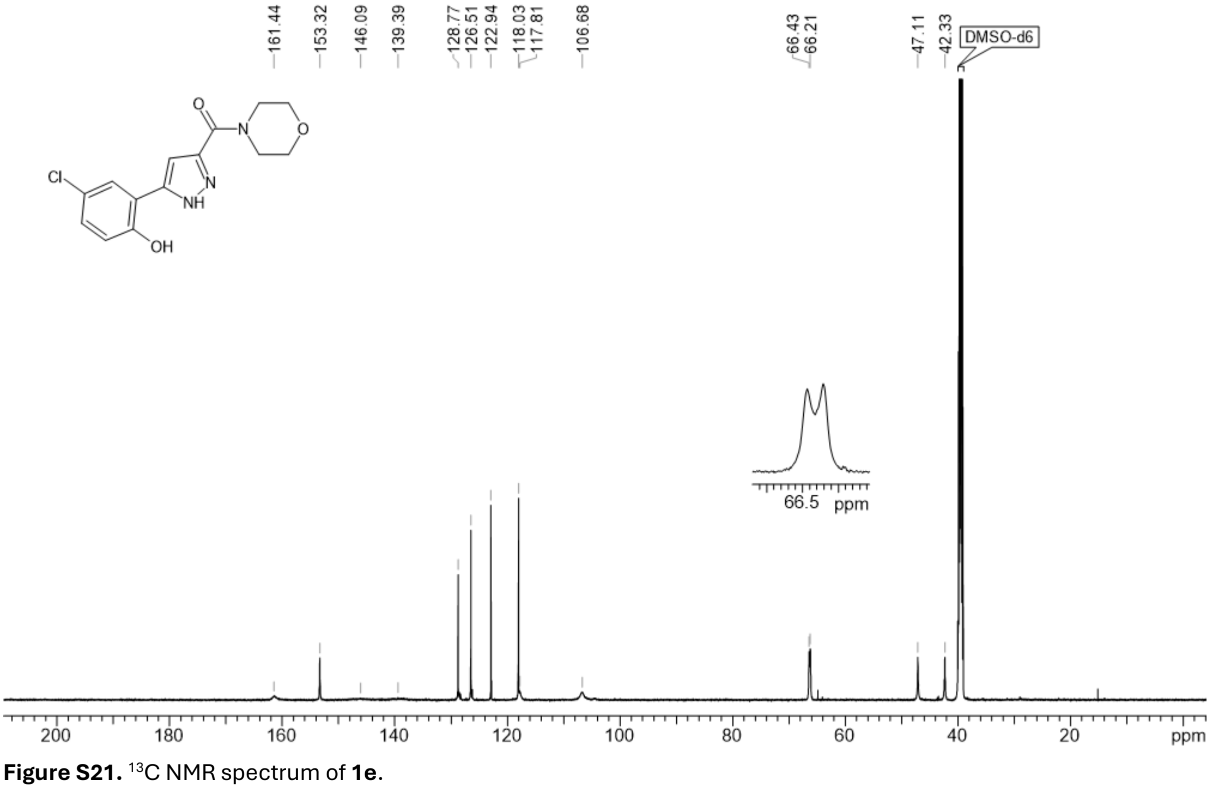

Figure S21. ^{13}C NMR spectrum of **1e**.

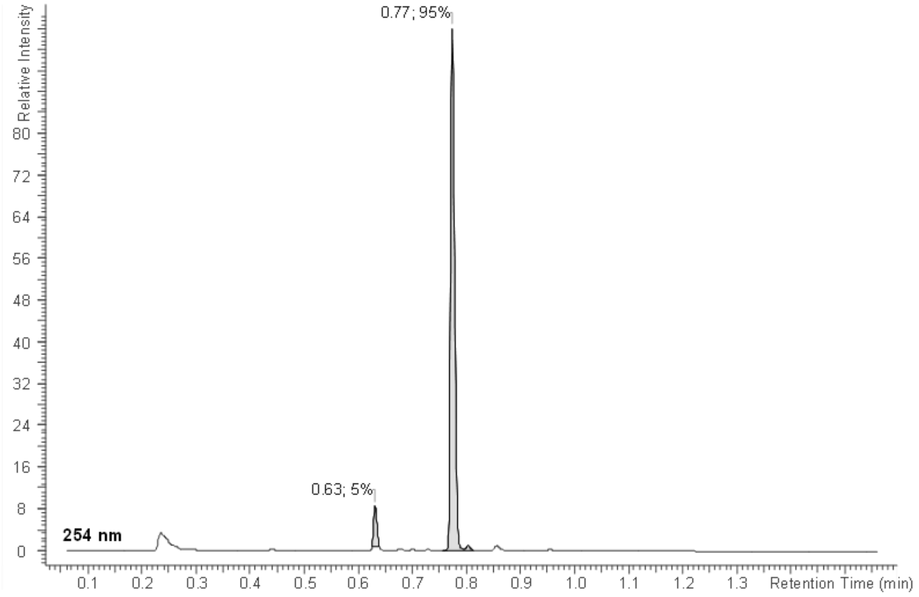

Figure S22. LCMS purity analysis of **1e**.

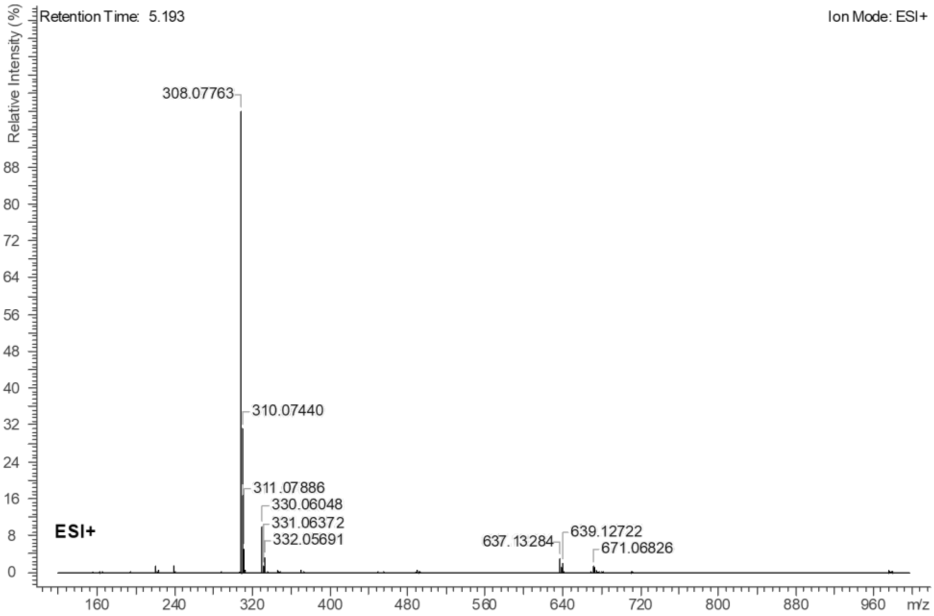

Figure S23. HRMS of **1e**.

Compound **2**

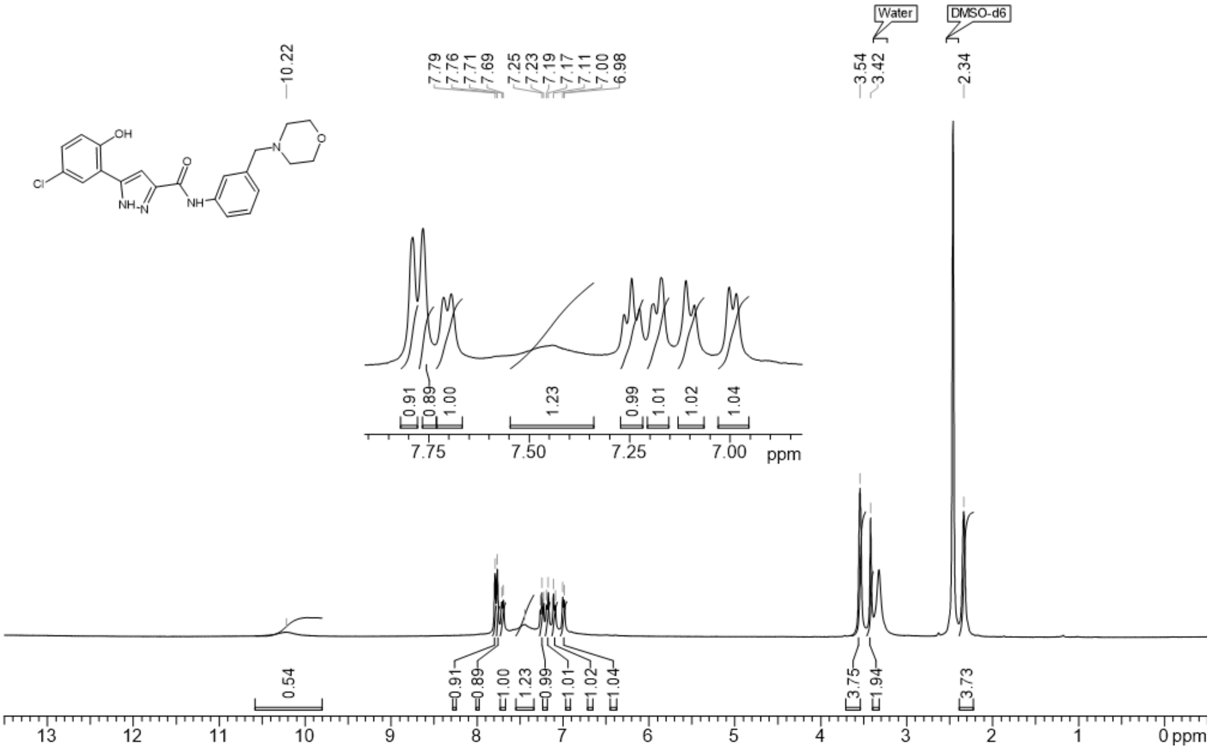

Figure S24. ^{1}H NMR spectrum of **2**.

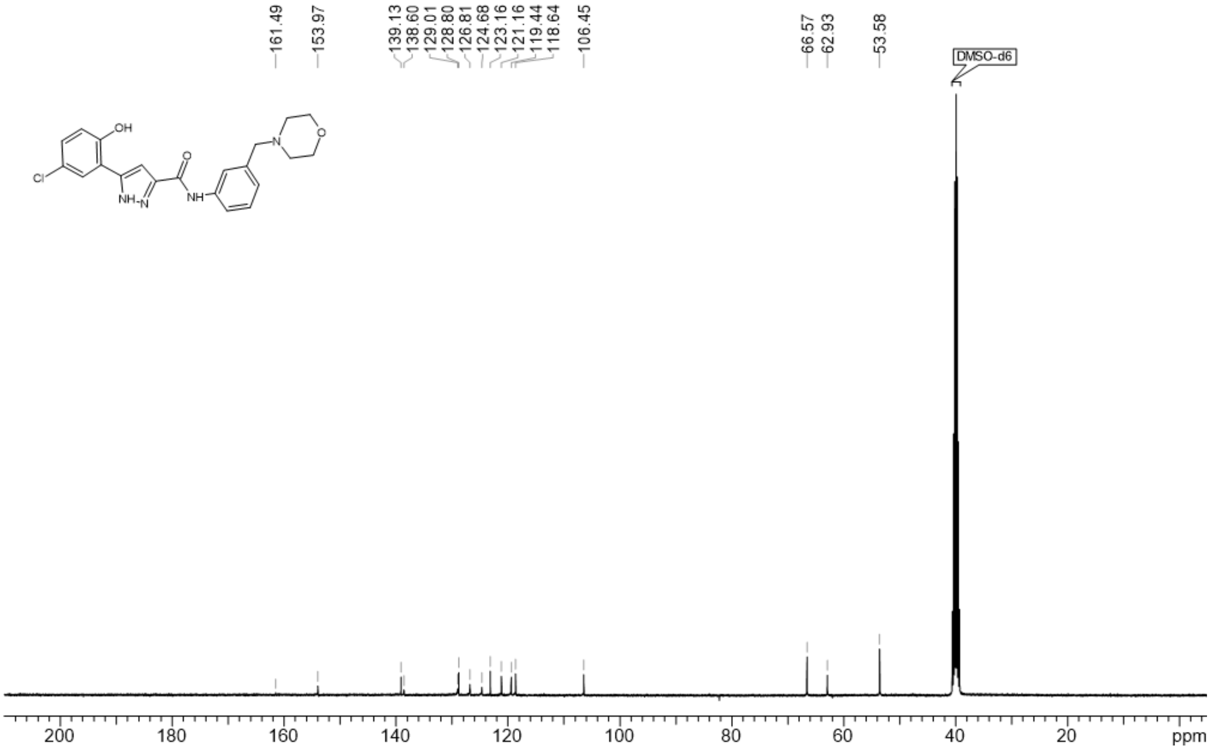

Figure S25. ^{13}C NMR spectrum of **2**.

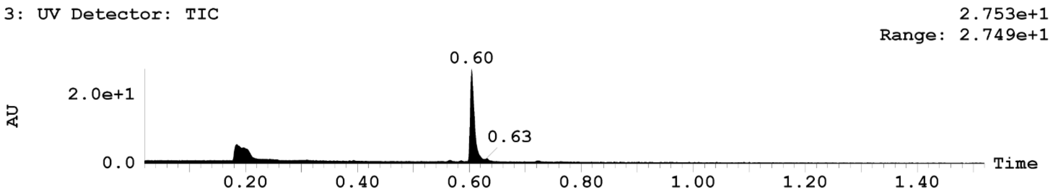

Peak Number	Time	Area %Total
1	0.60	97.37
2	0.63	2.63

Figure S26. LCMS purity analysis of **2**.

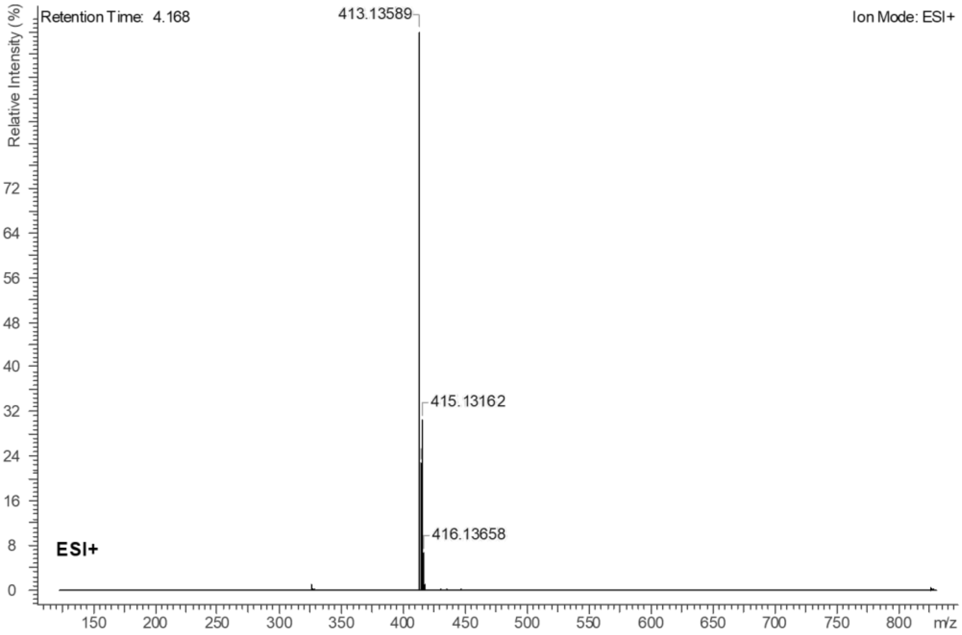

Figure S27. HRMS of **2**.

Compounds **3A–B**

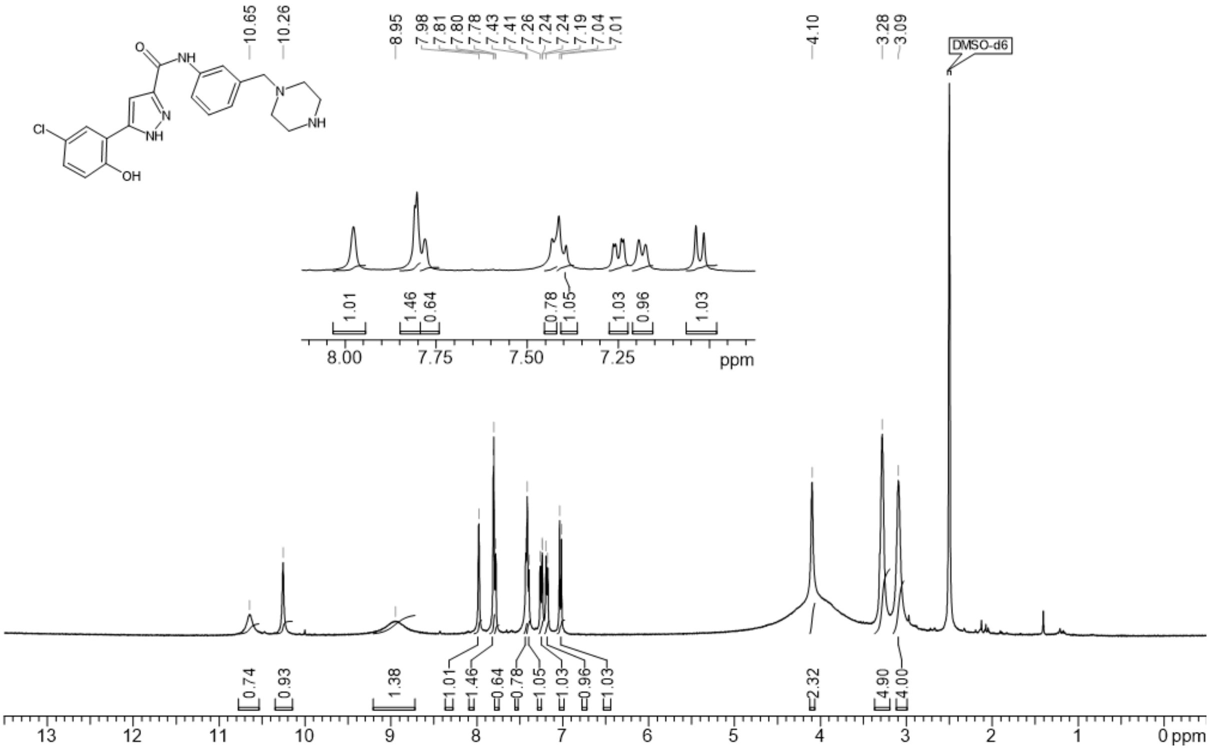

Figure S28. ^{1}H NMR spectrum of **3A**.

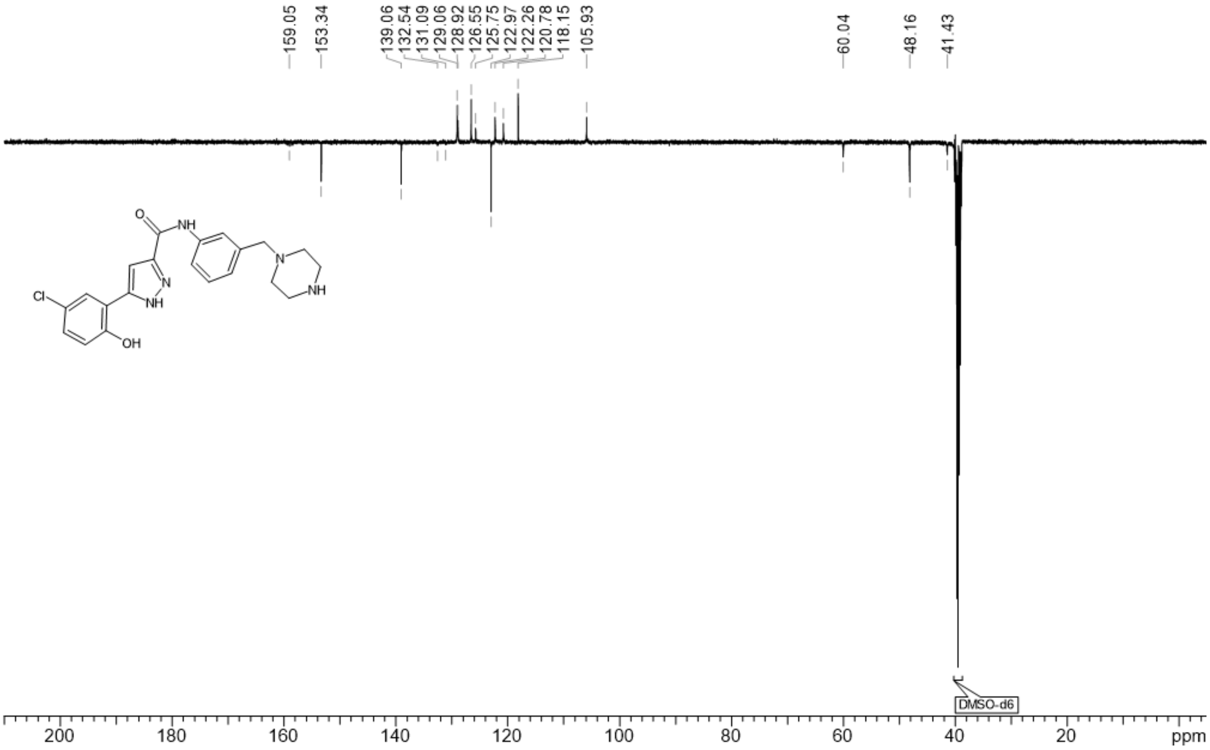

Figure S29. ^{13}C APT NMR spectrum of **3A**.

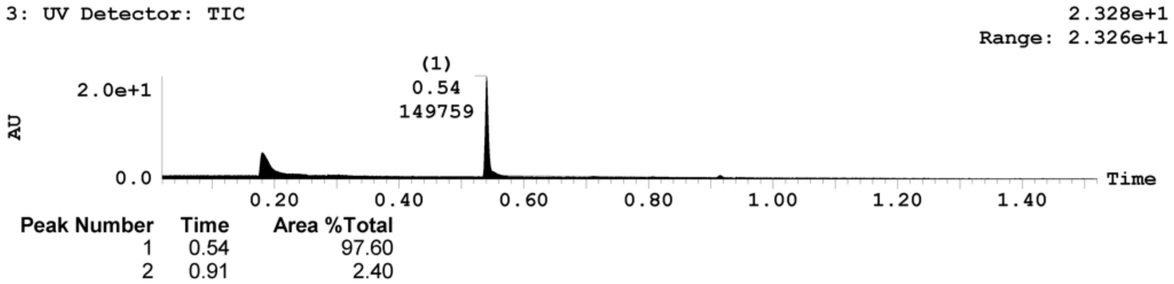

Figure S30. LCMS purity analysis of **3A**.

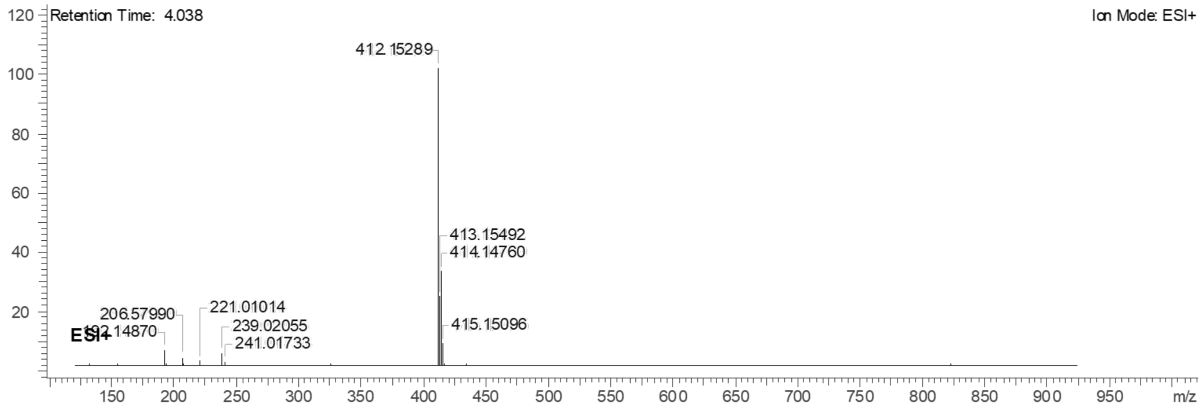

Figure S31. HRMS of **3A**.

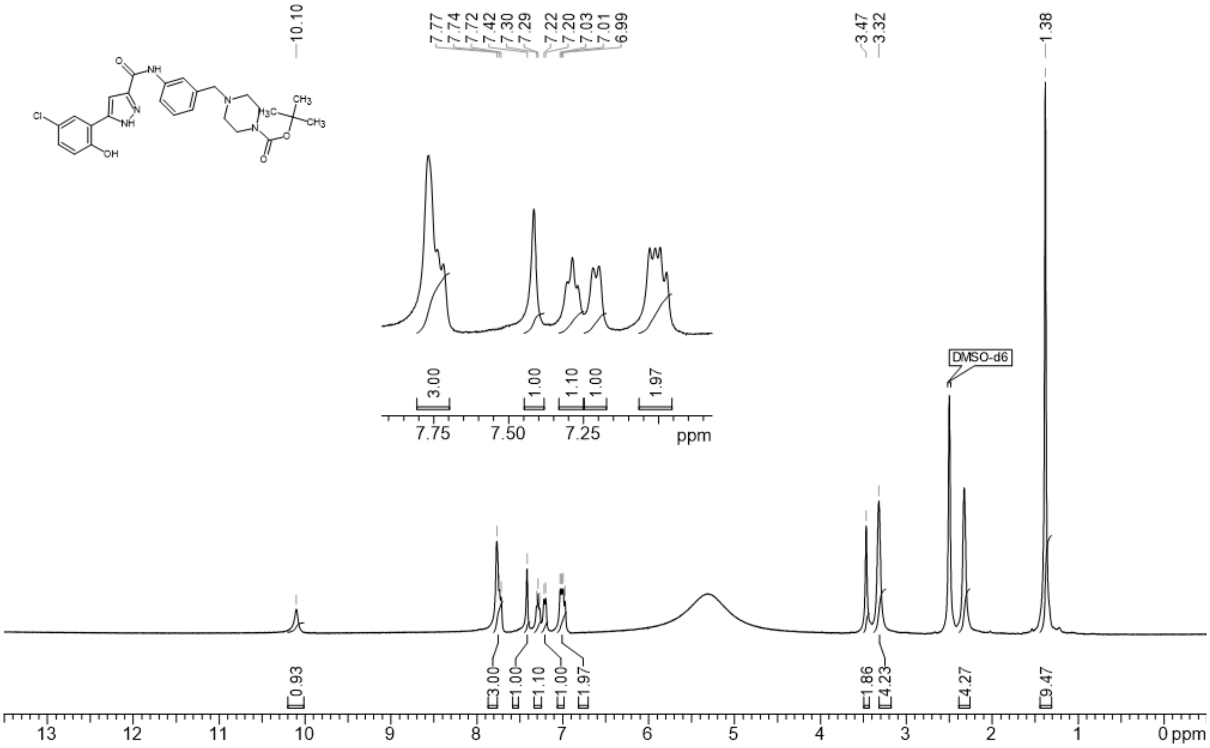

Figure S32. ^{1}H NMR spectrum of **3B**.

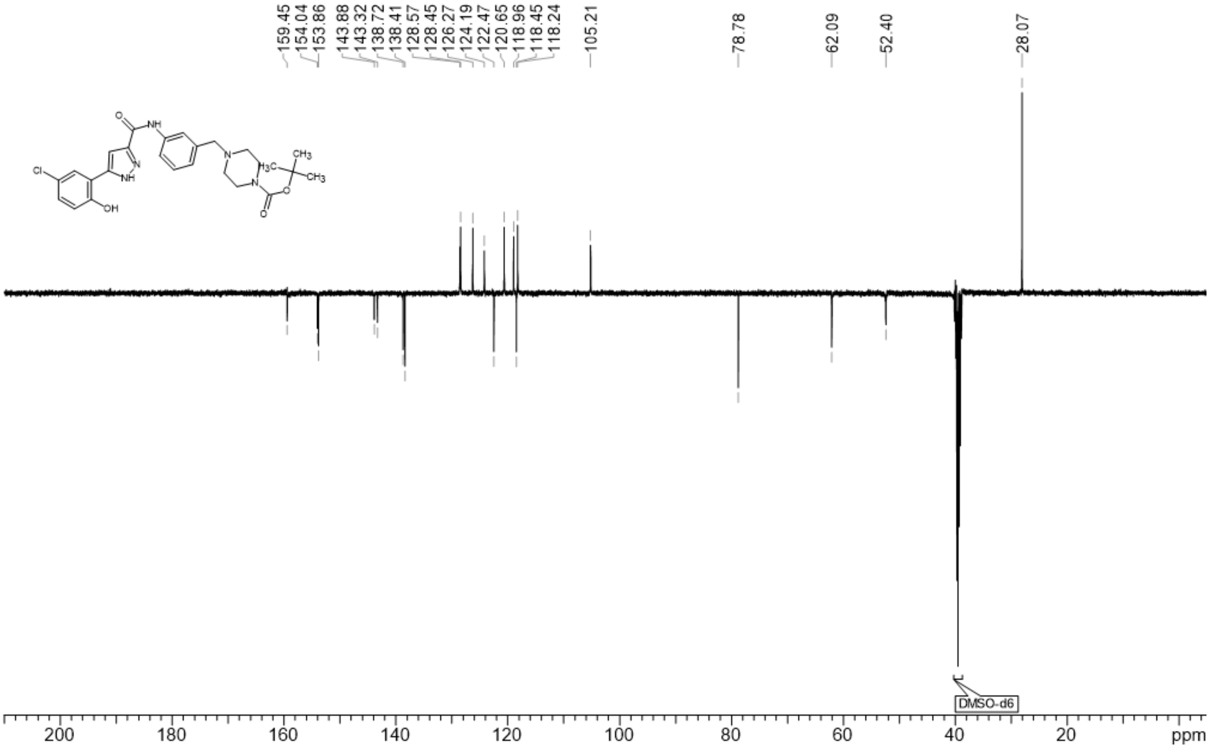

Figure S33. ^{13}C NMR spectrum of **3B**.

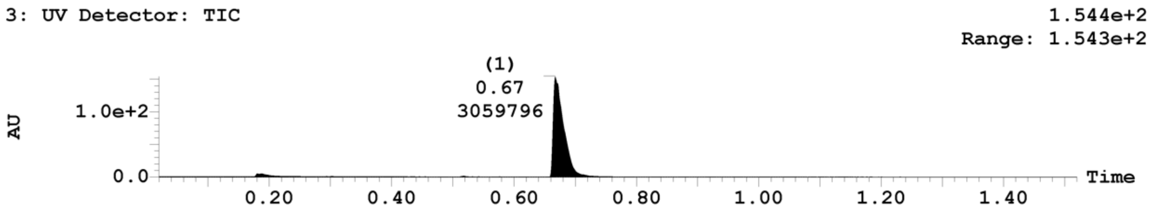

Peak Number	Time	Area %Total
1	0.67	100.00

Figure S34. LCMS purity analysis of **3B**.

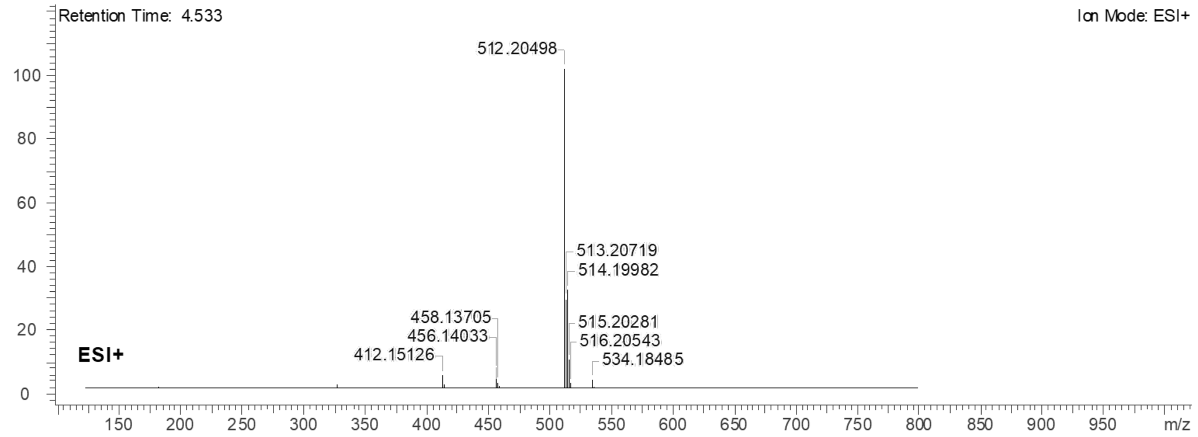

Figure S35. HRMS of **3B**.

Compounds **4A–B**

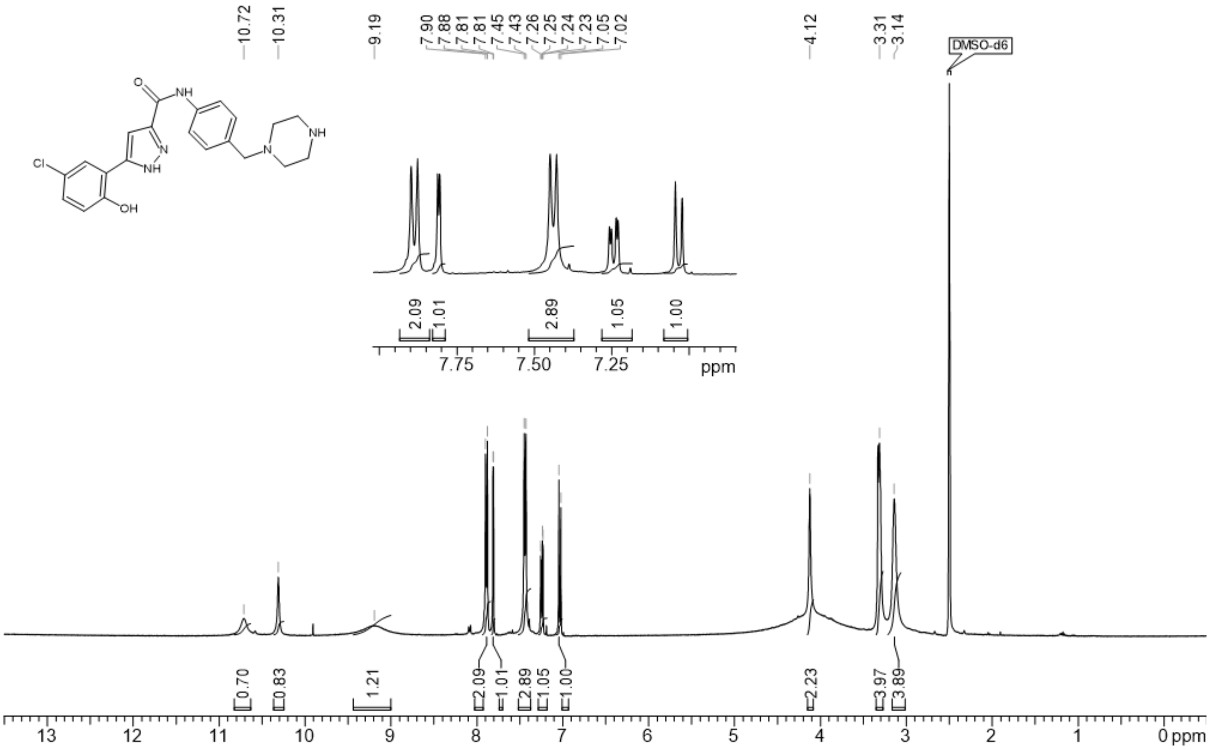

Figure S36. ^{1}H NMR spectrum of **4A,** C–H of pyrazole under 7.44 ppm (br d).

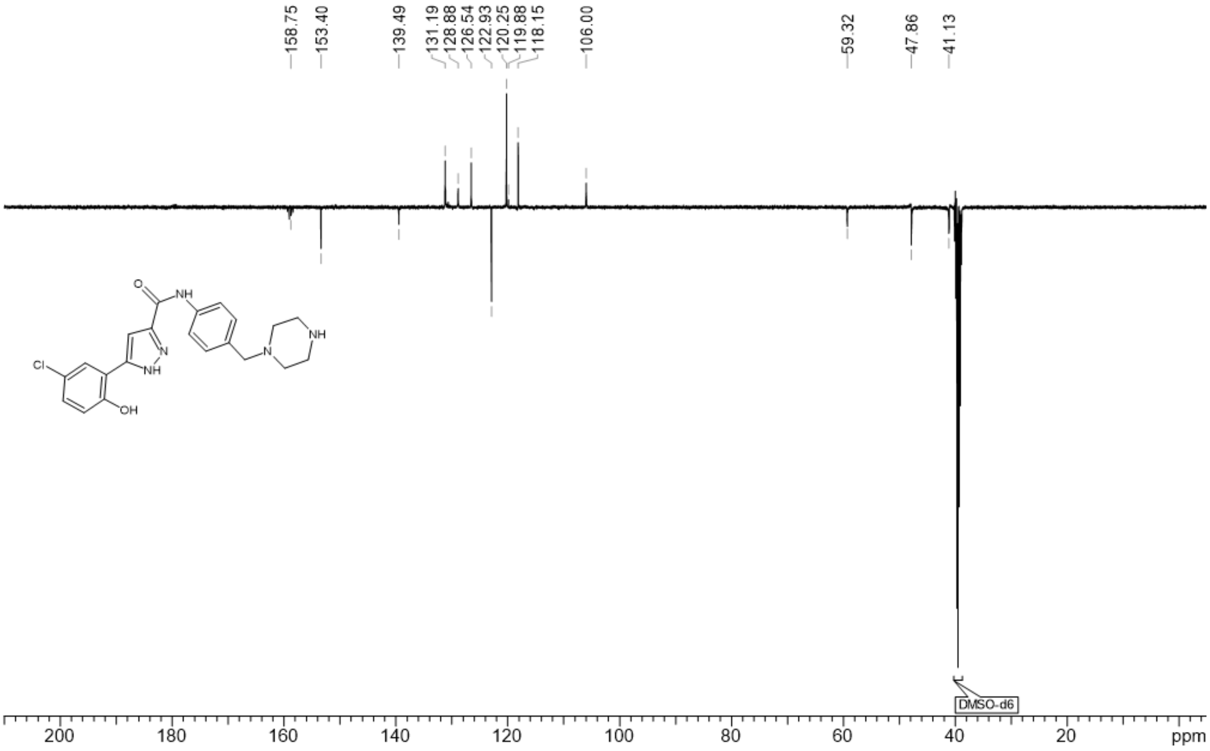

Figure S37. ^{13}C APT NMR spectrum of **4A**.

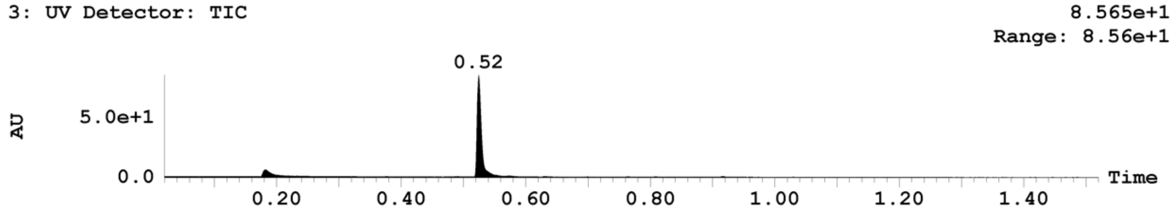

Peak Number	Time	Area %Total
1	0.52	100.00

Figure S38. LCMS purity analysis of **4A**.

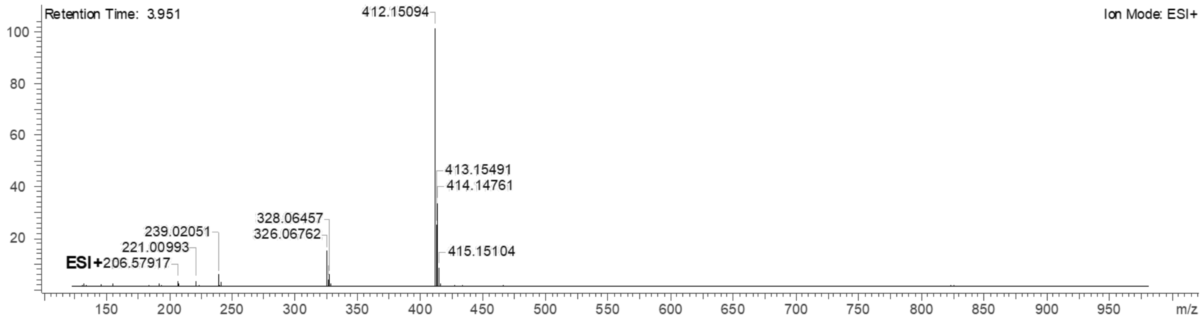

Figure S39. HRMS of **4A**.

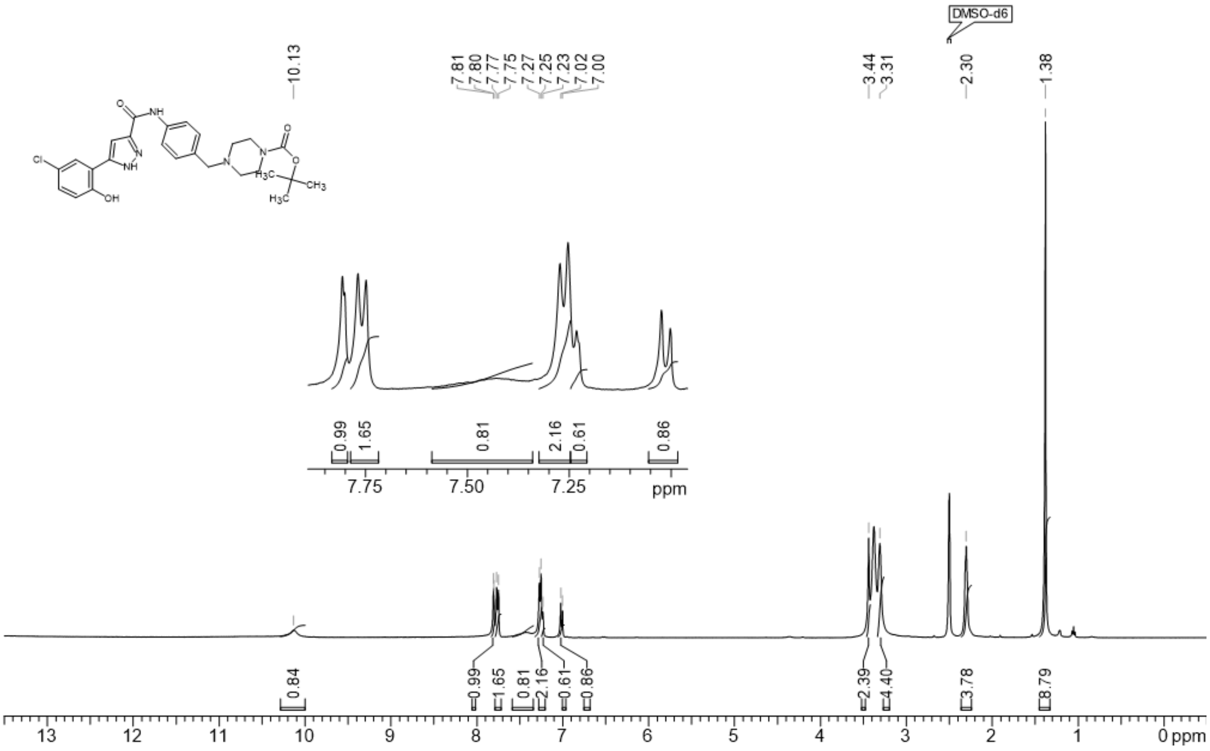

Figure S40. ^{1}H NMR spectrum of **4B**.

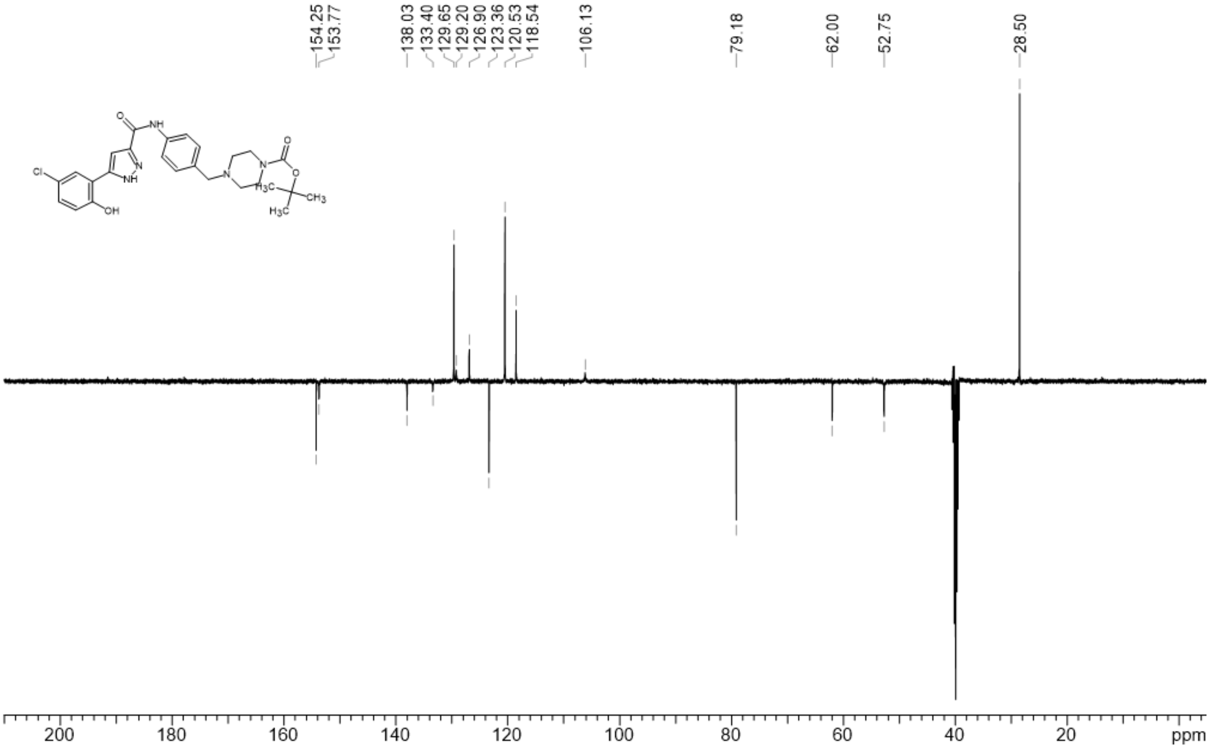

Figure S41. ^{13}C APT NMR spectrum of **4B**.

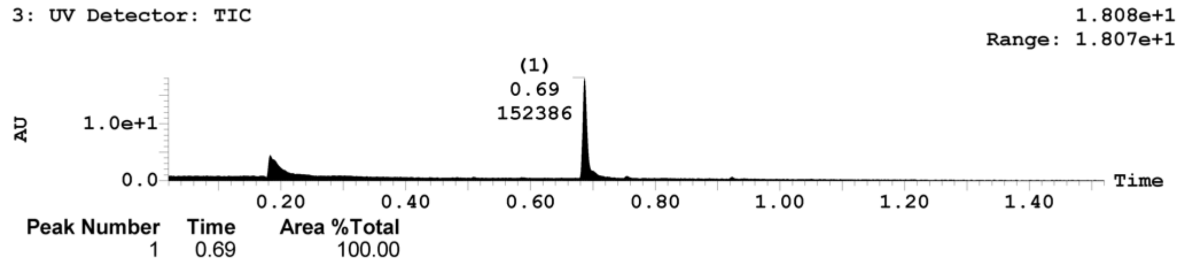

Figure S42. LCMS purity analysis of **4B**.

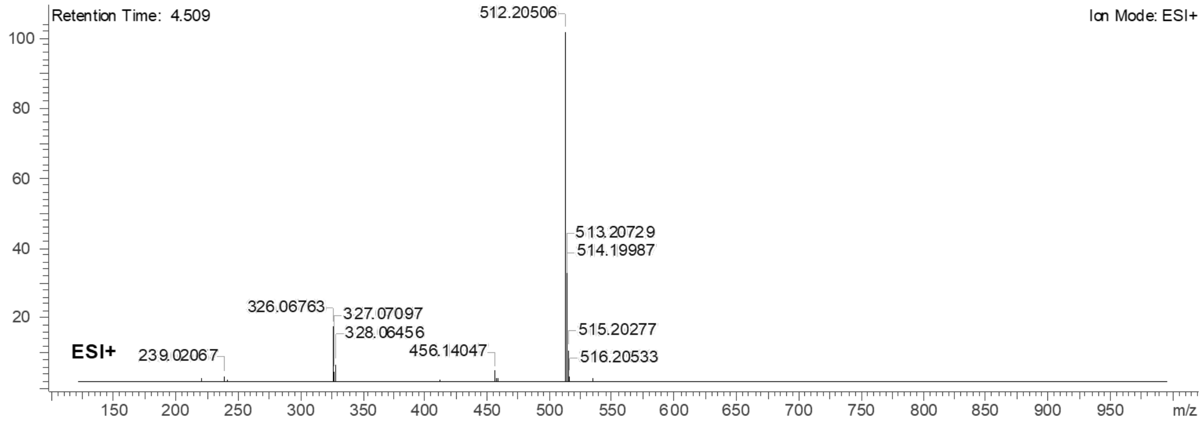

Figure S43. HRMS of **4B**.

Compounds **5A–G**

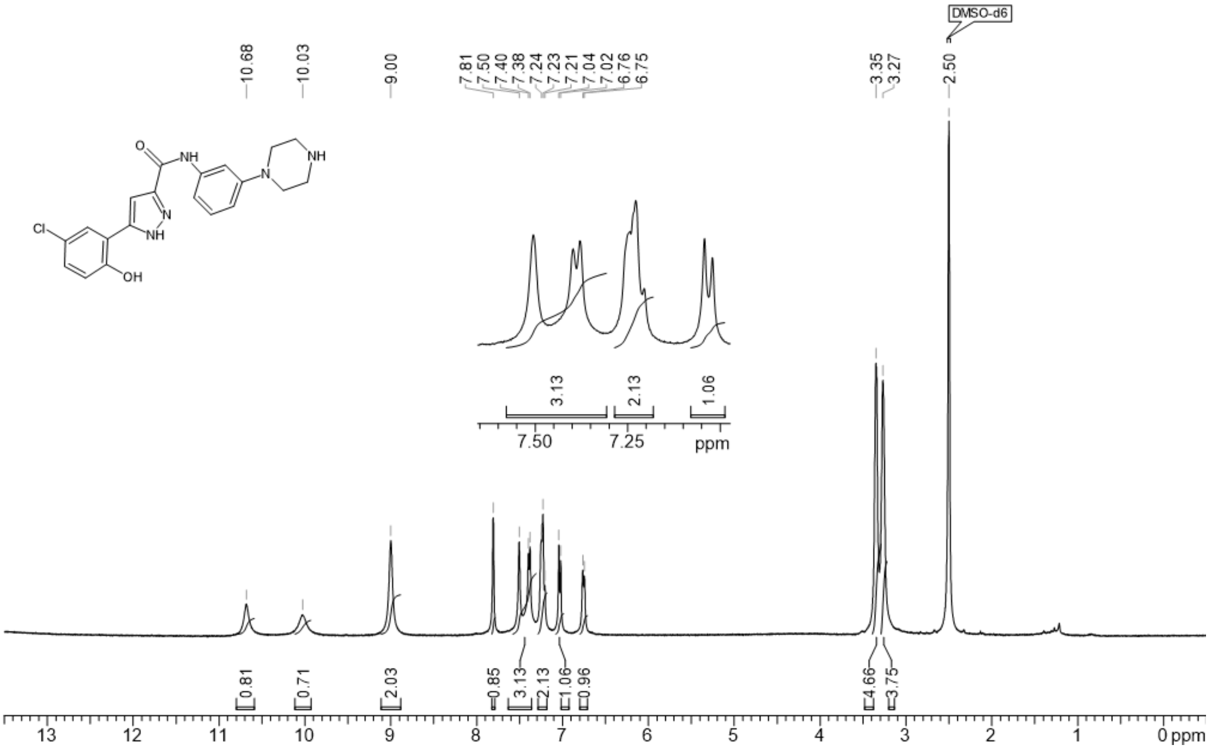

Figure S44. ^{1}H NMR spectrum of **5A**.

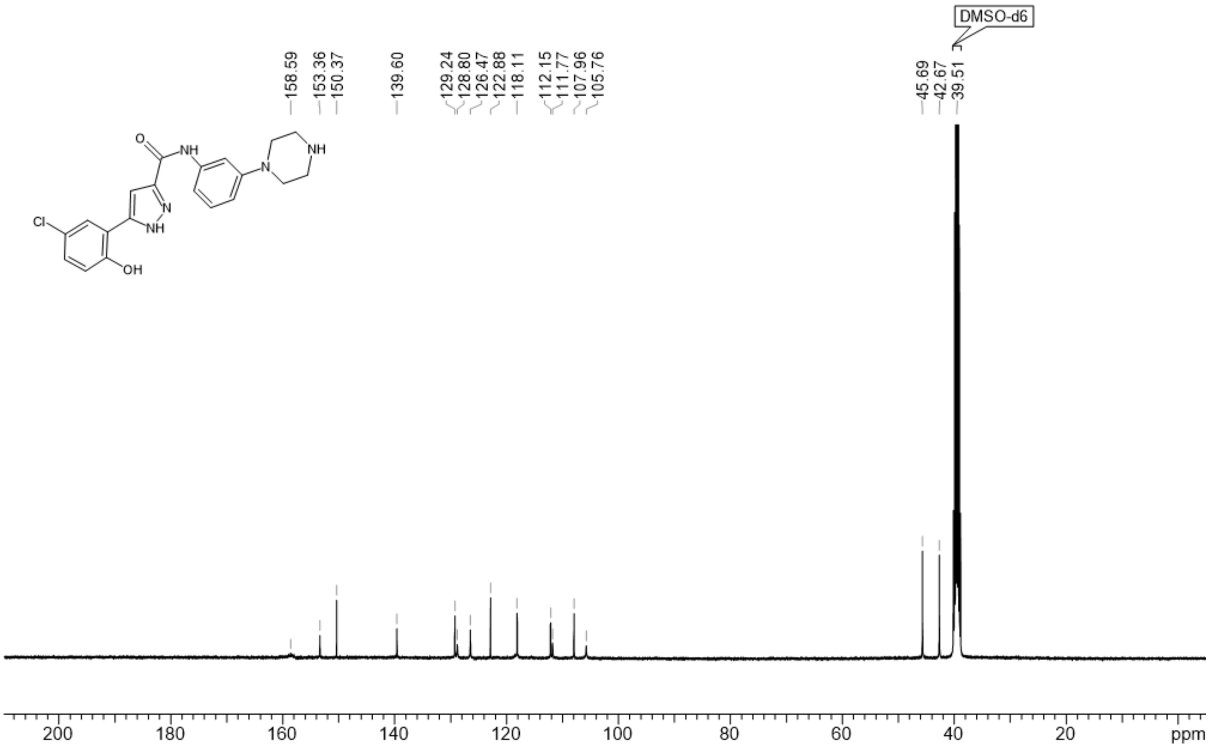

Figure S45. ^{13}C NMR spectrum of **5A**.

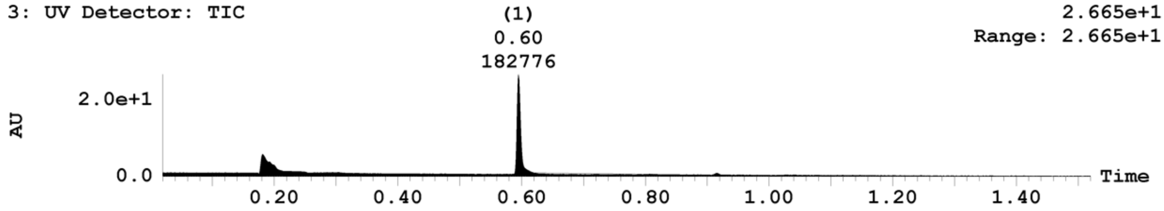

Peak Number	Time	Area %Total
1	0.59	97.88
2	0.92	2.12

Figure S46. LCMS purity analysis of **5A**.

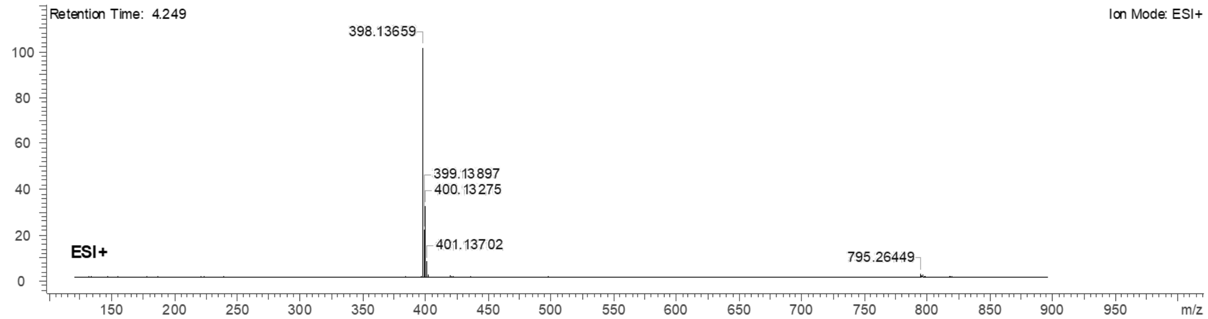

Figure S47. HRMS of **5A**.

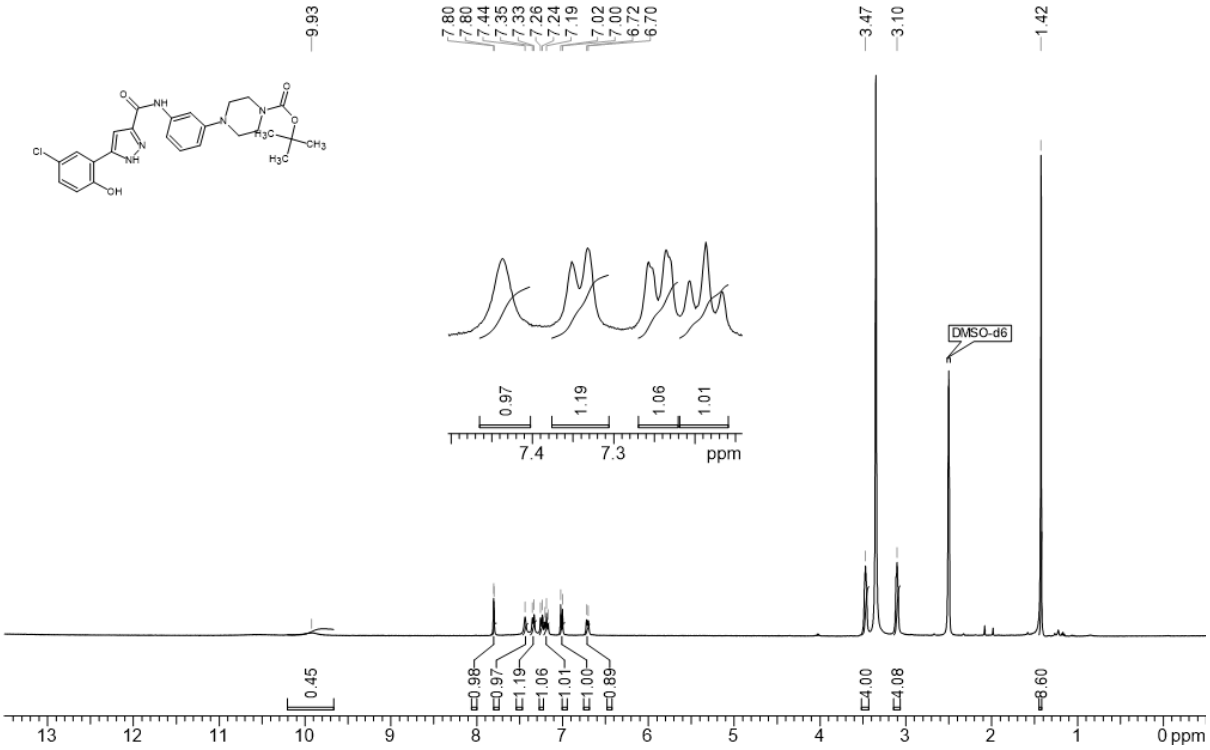

Figure S48. ^{1}H NMR spectrum of **5B**.

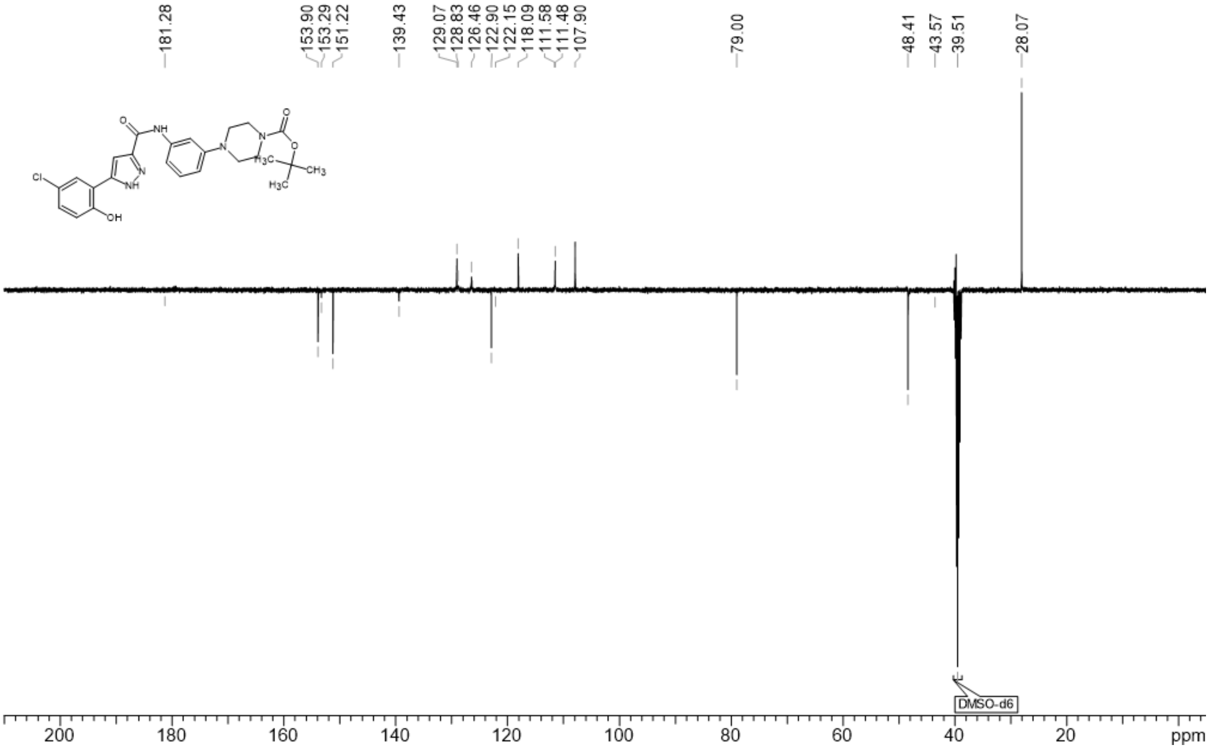

Figure S49. ^{13}C APT NMR spectrum of **5B**.

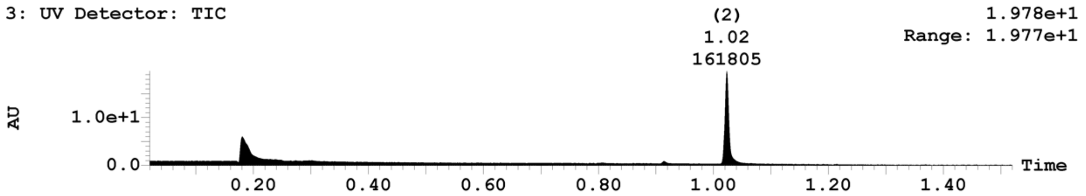

Peak Number	Time	Area %Total
1	0.91	2.60
2	1.02	97.40

Figure S50. LCMS purity analysis of **5B**.

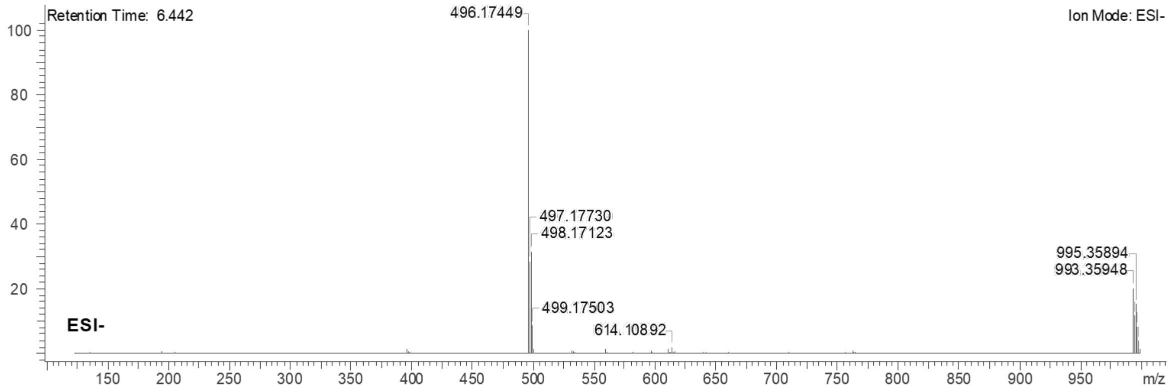

Figure S51. HRMS of **5B.**

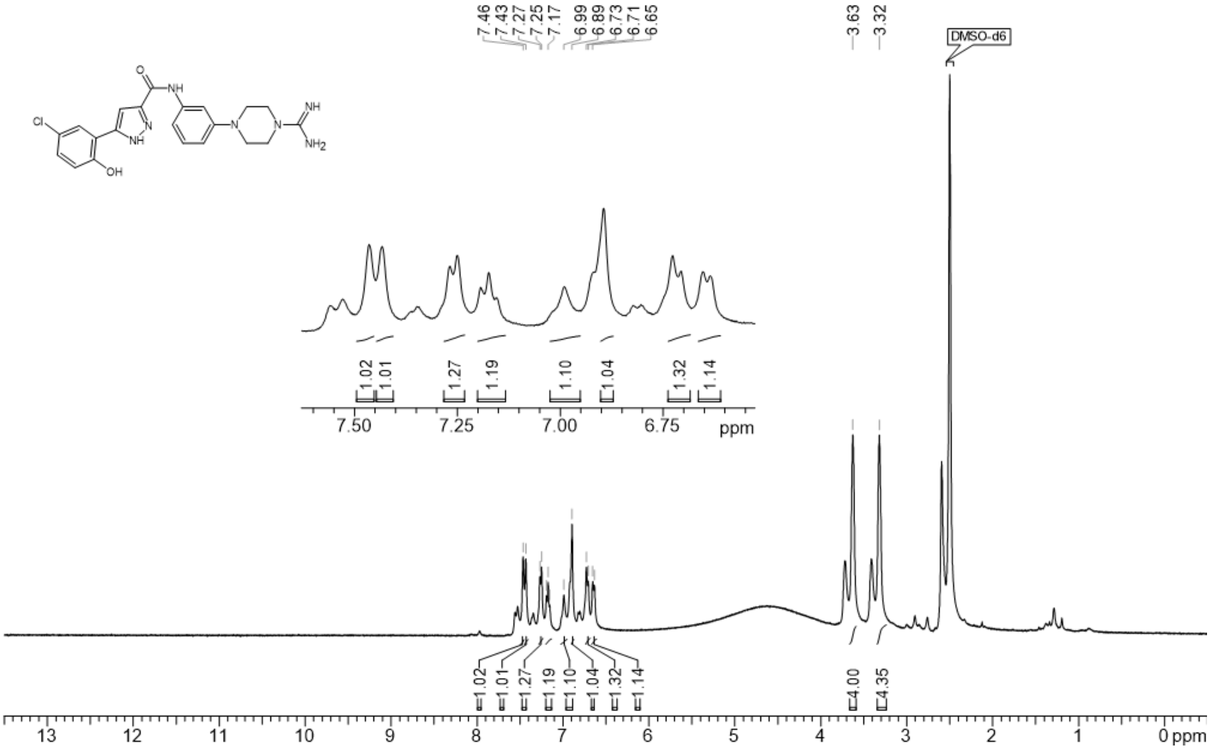

Figure S52. ^{1}H NMR spectrum of **5G** at 120 °C, peaks are splitting in two due to elevated temperature.

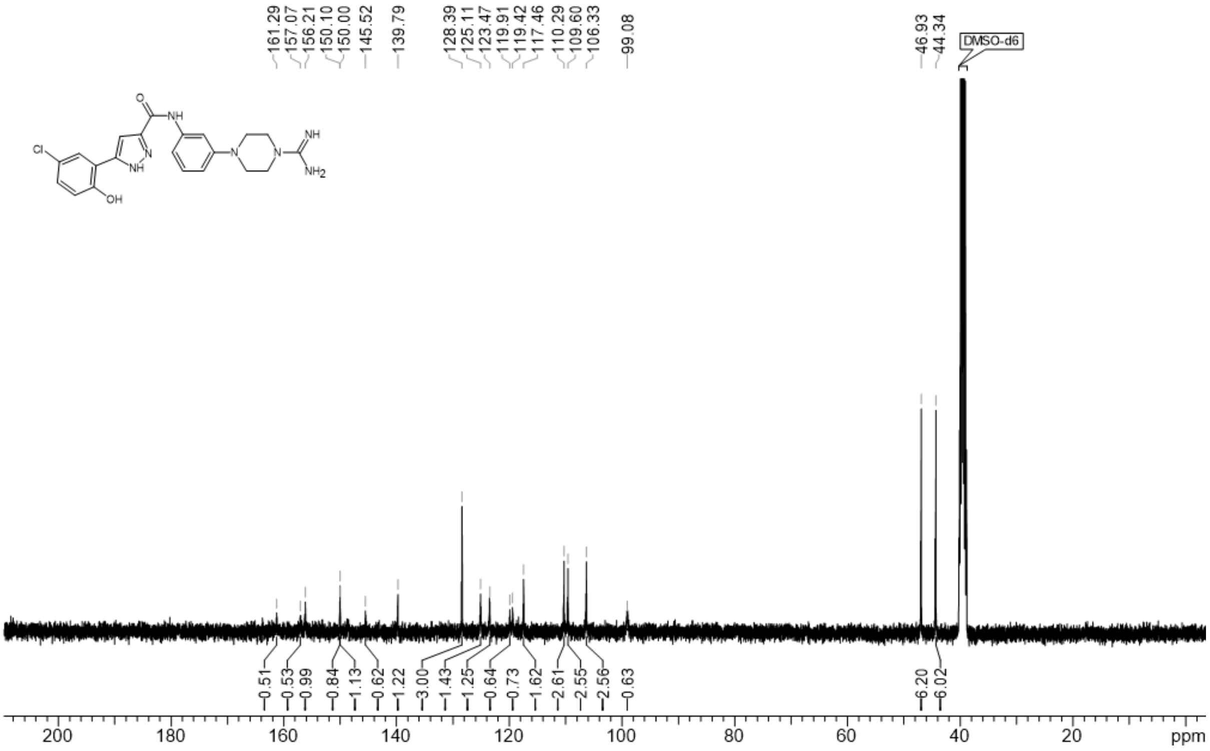

Figure S53. ^{13}C NMR spectrum of **5G** at 120 °C.

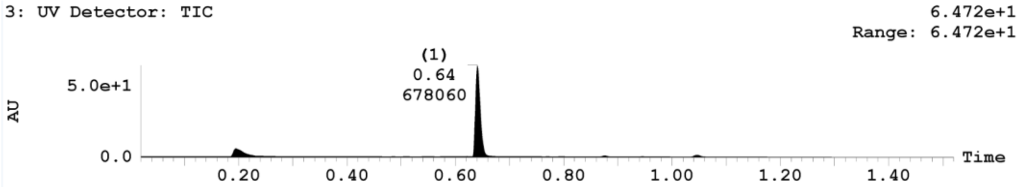

Peak Number	Time	Area %Total
1	0.64	100.00

Figure S54. LCMS purity analysis of **5G.**

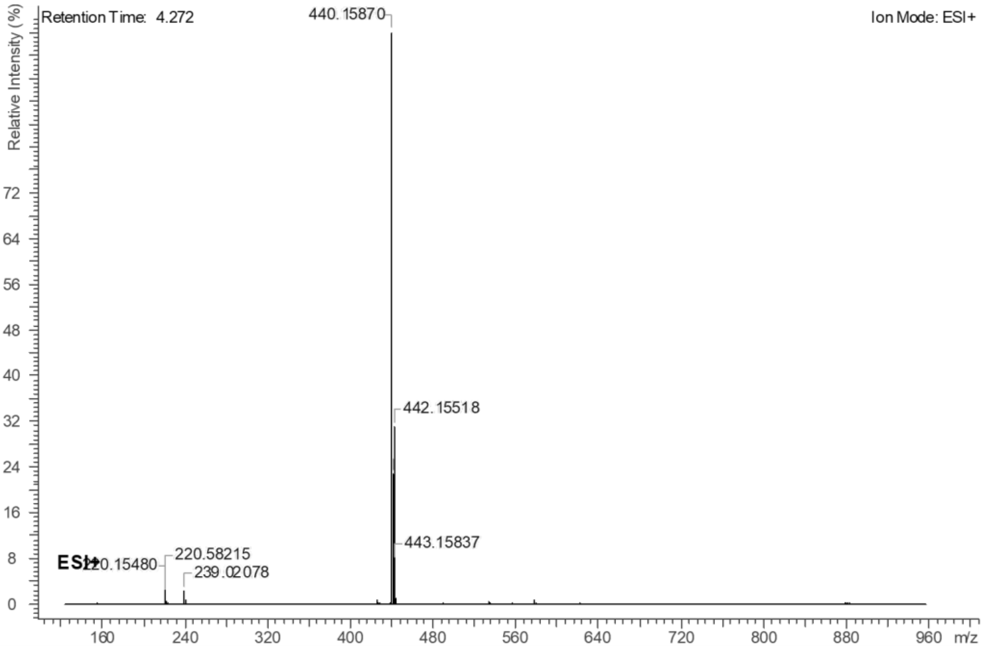

Figure S55. HRMS of **5G.**

Compounds **6A–G**

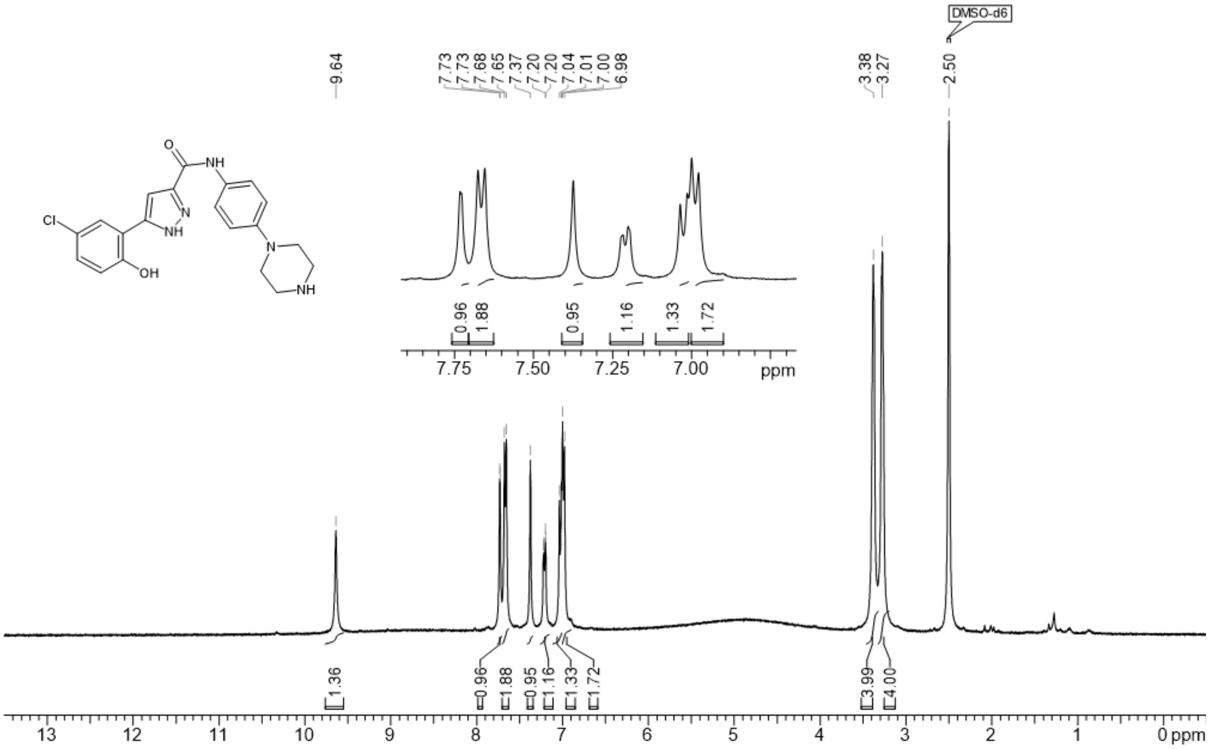

Figure S56. ^{1}H NMR spectrum at 120 °C of **6A.**

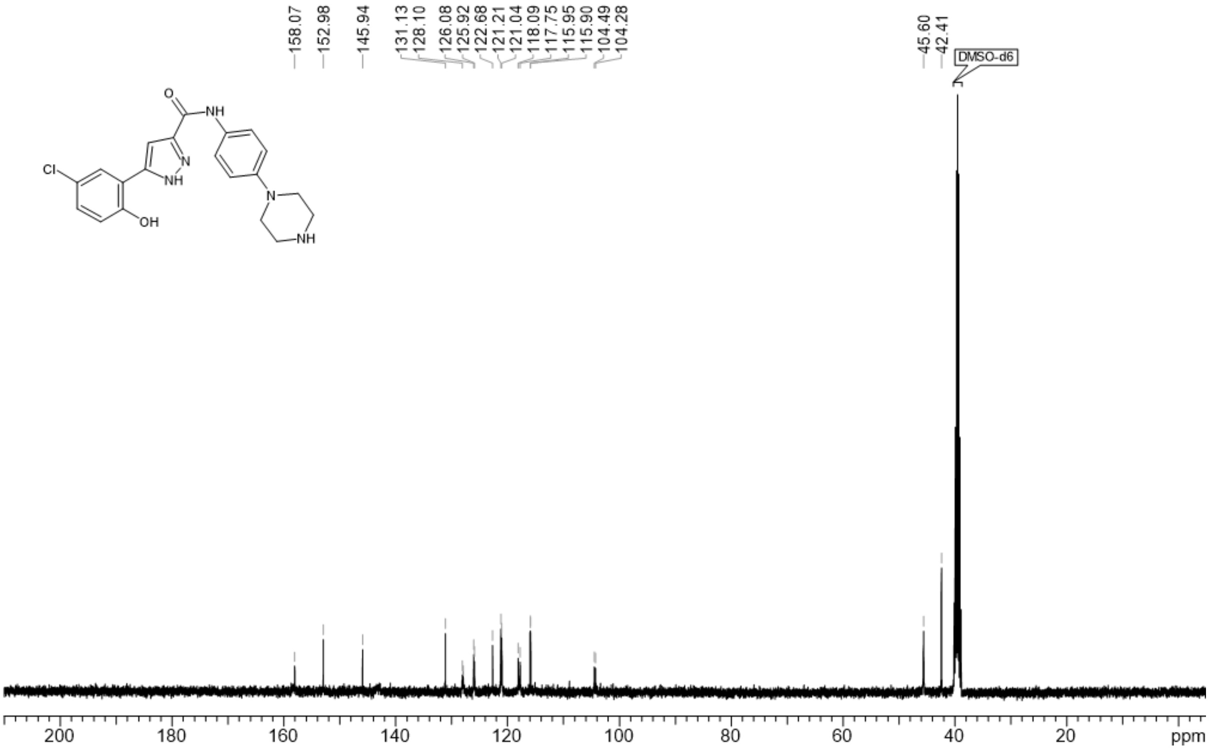

Figure S57. ^{13}C NMR spectrum at 120 °C of **6A.**

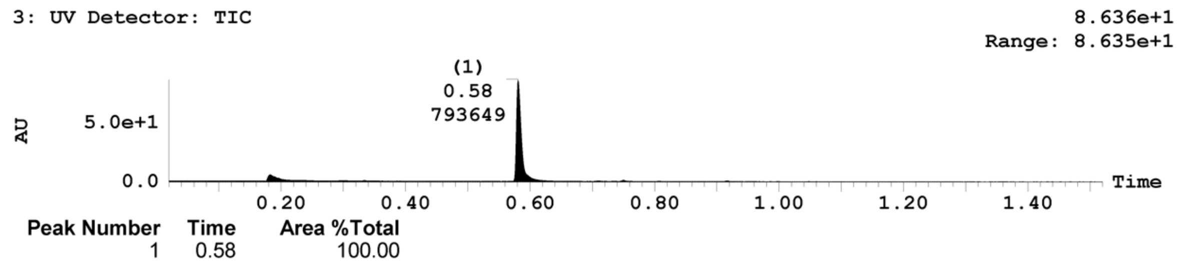

Figure S58. LCMS purity analysis of **6A.**

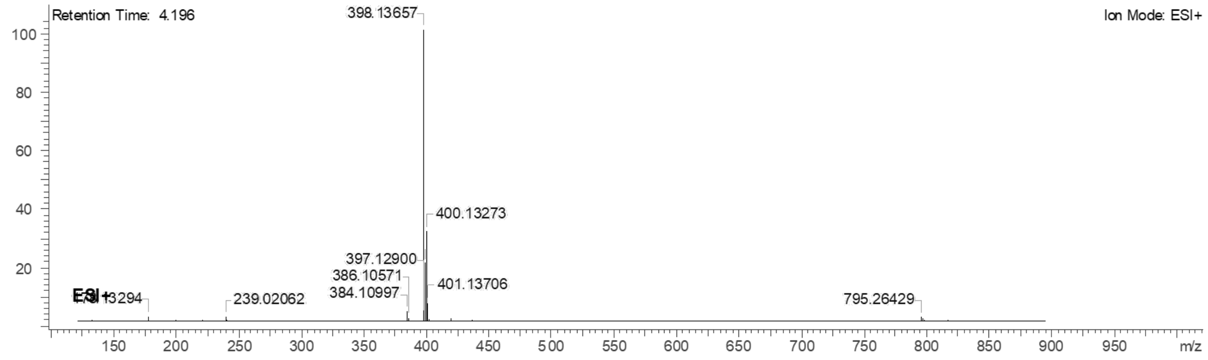

Figure S59. HRMS of **6A.**

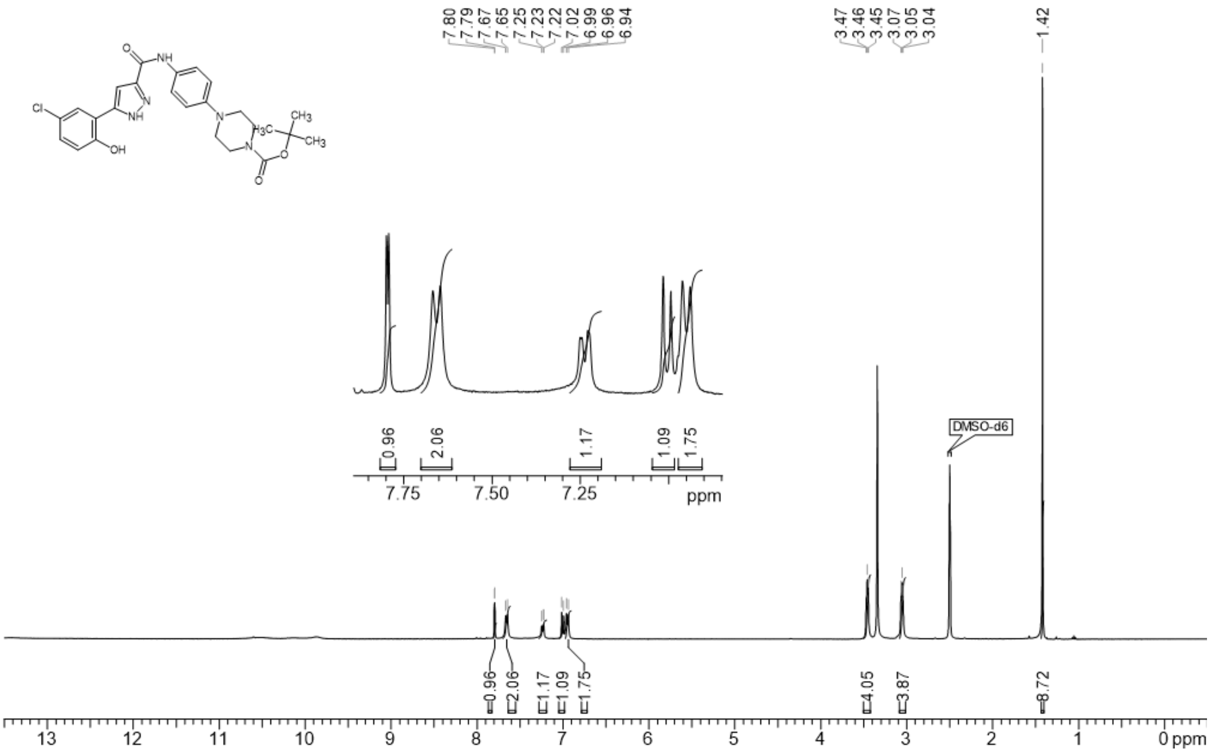

Figure S60. ^{1}H NMR spectrum of **6B.**

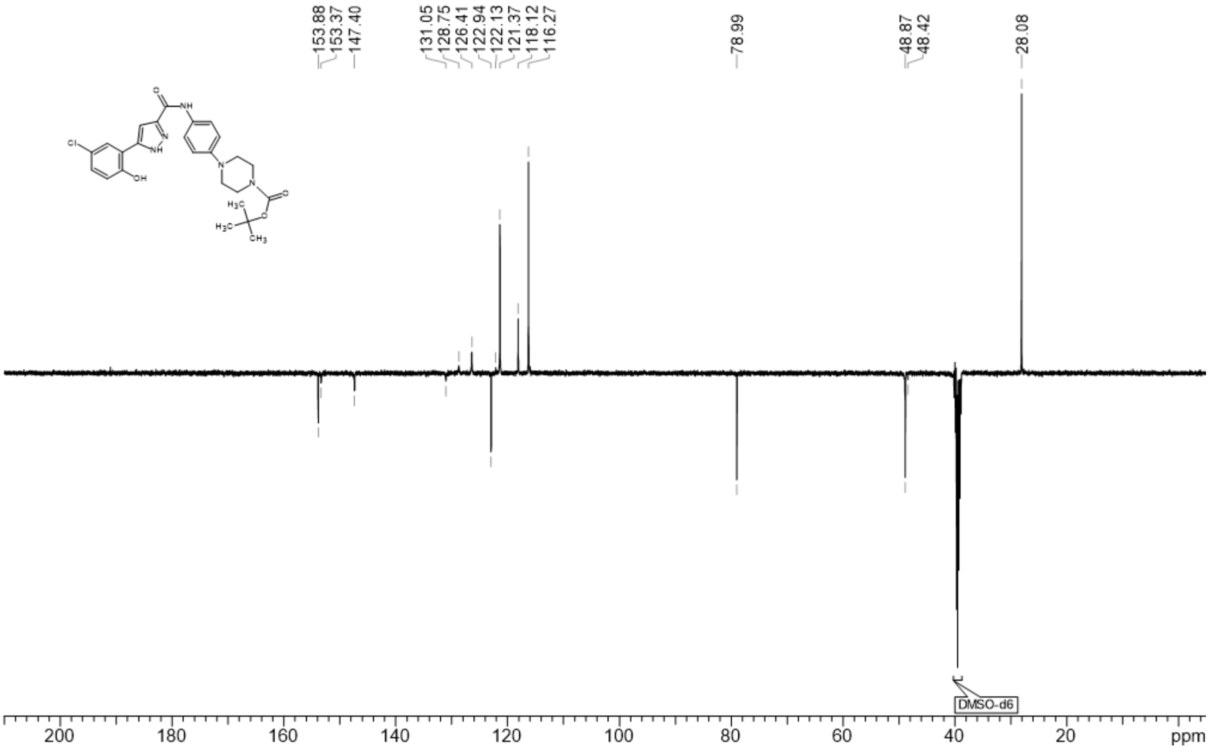

Figure S61. ^{13}C APT NMR spectrum of **6B.**

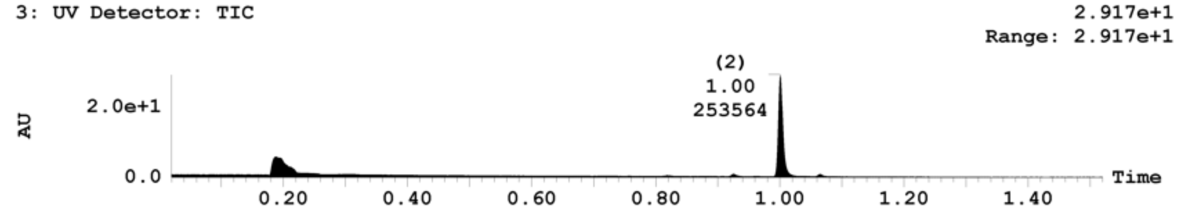

Peak Number	Time	Area %Total
1	0.92	2.29
2	1.00	95.64
3	1.07	2.07

Figure S62. LCMS purity analysis of **6B.**

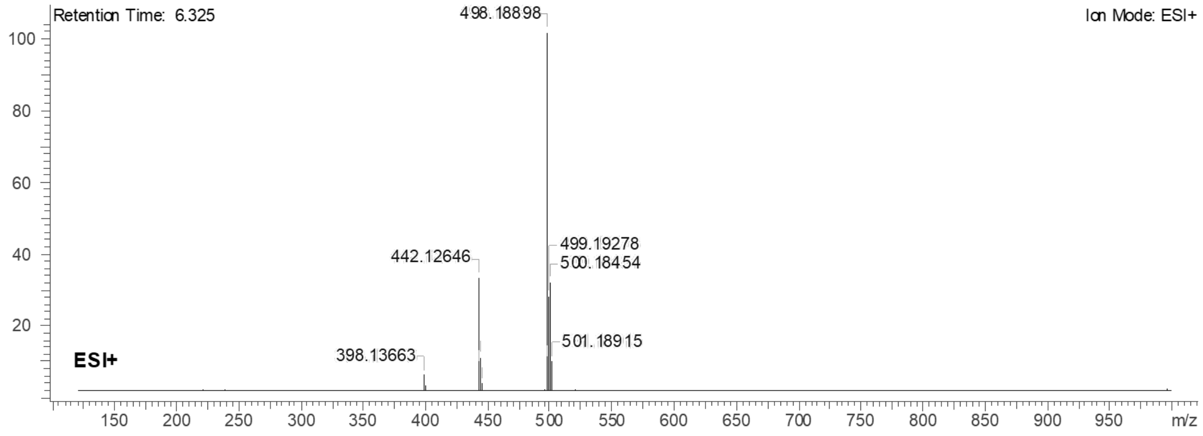

Figure S63. HRMS of **6B.**

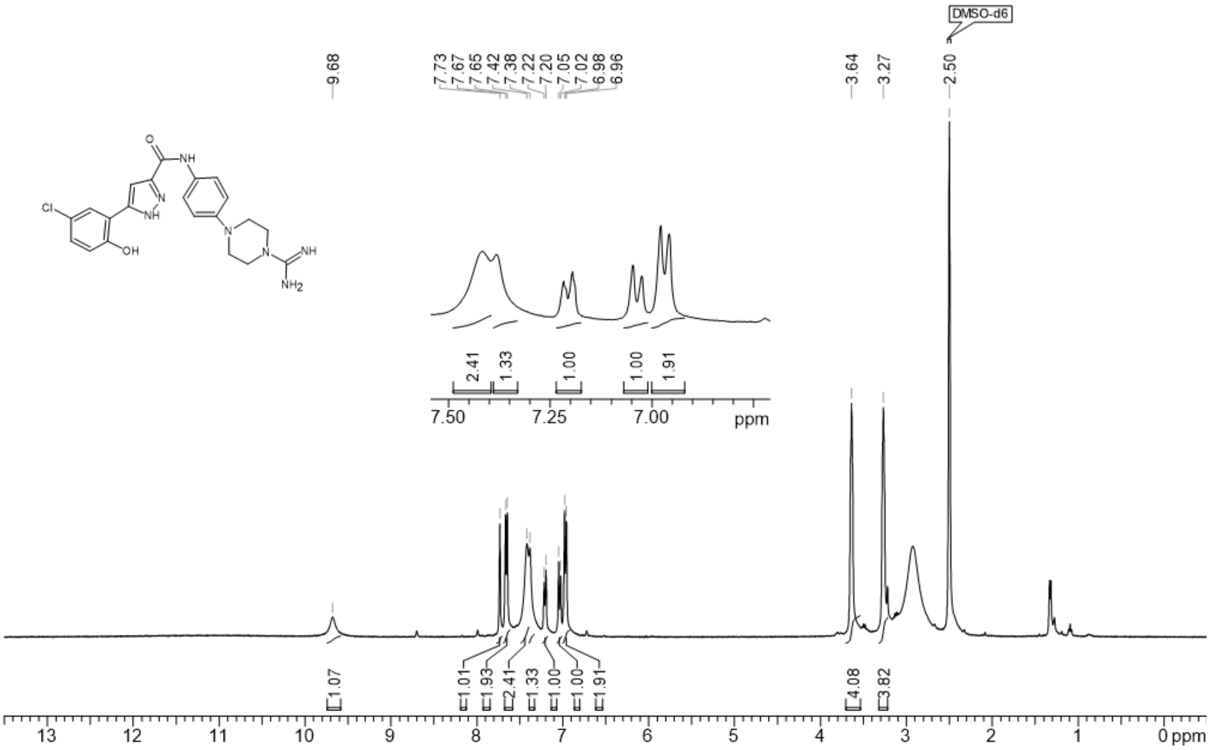

Figure S64. ^{1}H NMR spectrum at 120 °C of **6G.**

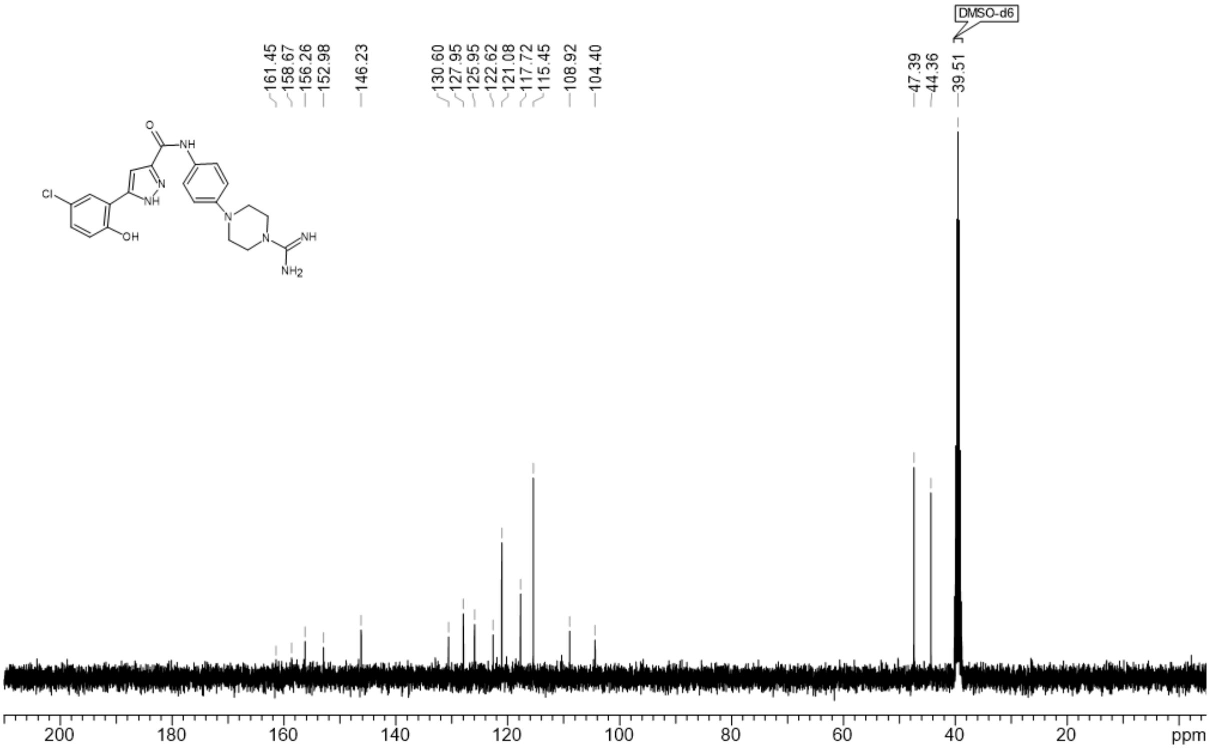

Figure S65. ^{13}C NMR spectrum at 120 °C of **6G.**

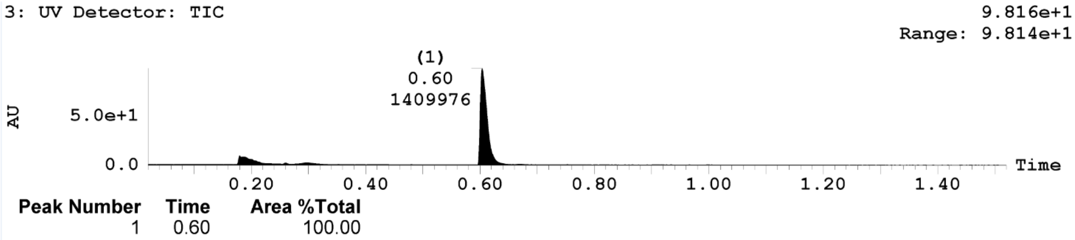

Figure S66. LCMS purity analysis of **6G.**

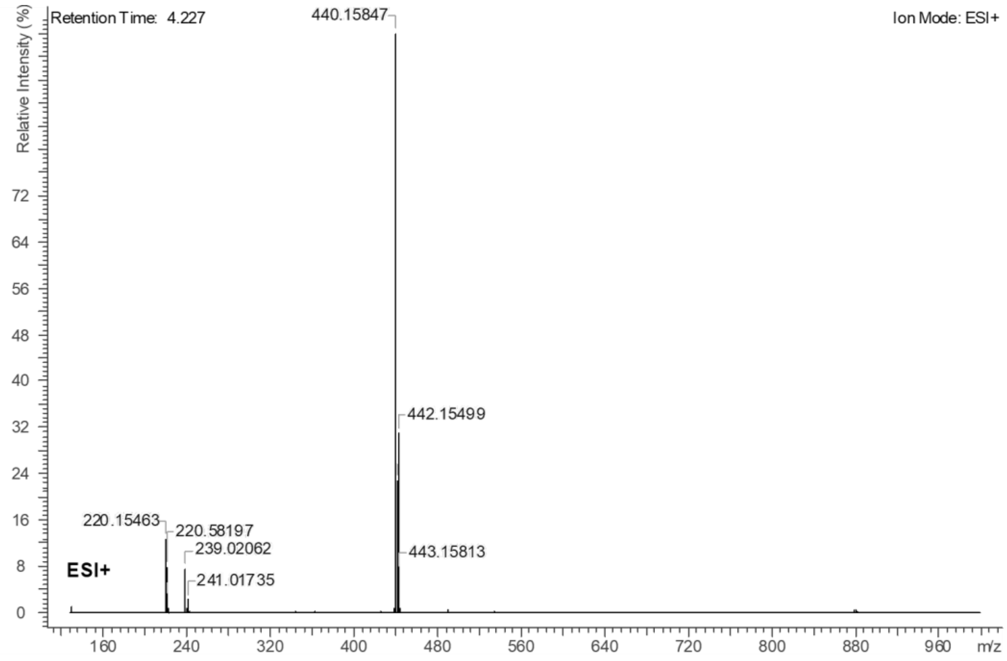

Figure S67. HRMS of **6G.**

Compounds **7A–G**

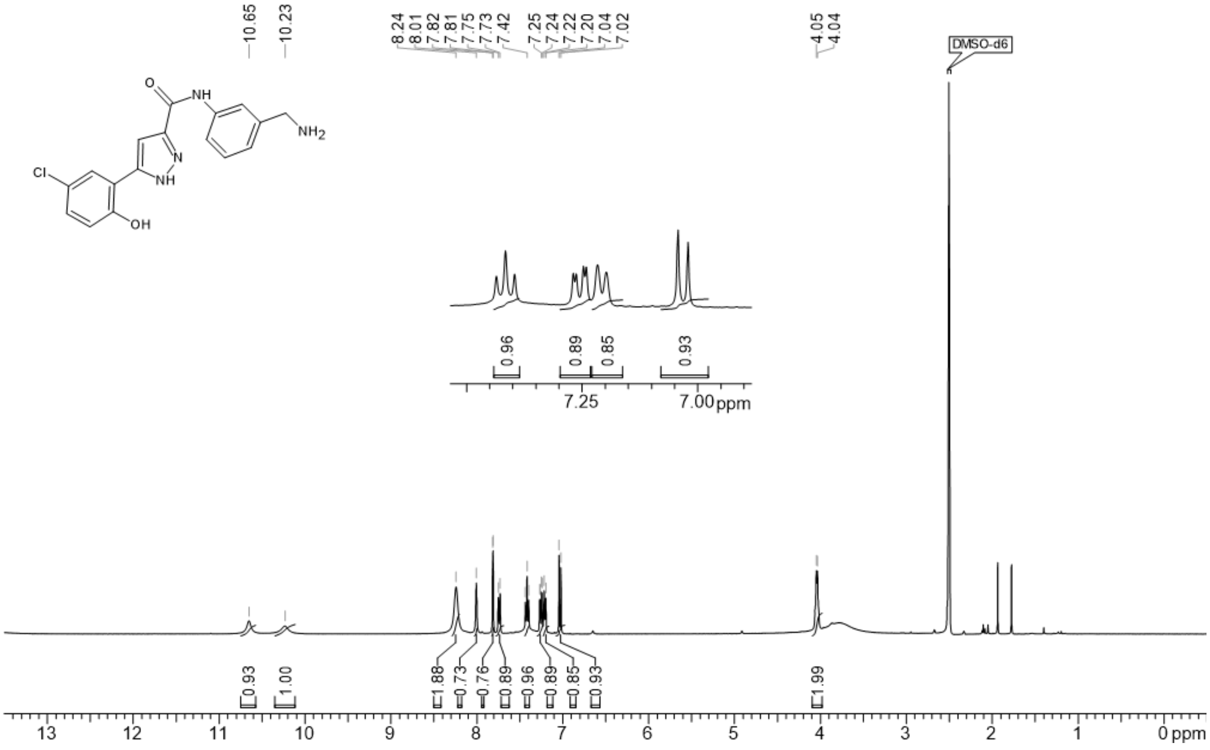

Figure S68. ^{1}H NMR spectrum of **7A.**

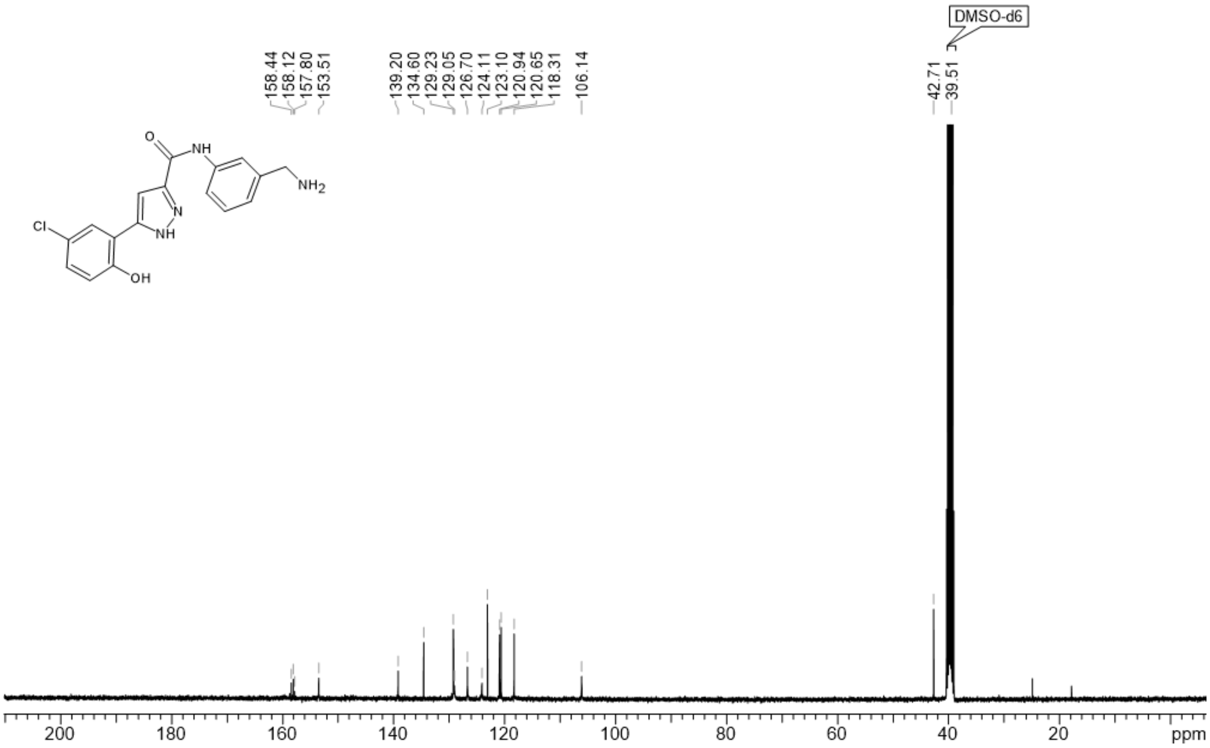

Figure S69. ^{13}C NMR spectrum of **7A.**

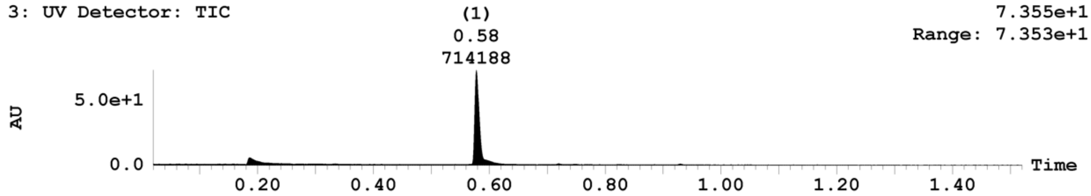

Peak Number	Time	Area %Total
1	0.58	100.00

Figure S70. LCMS purity analysis of **7A.**

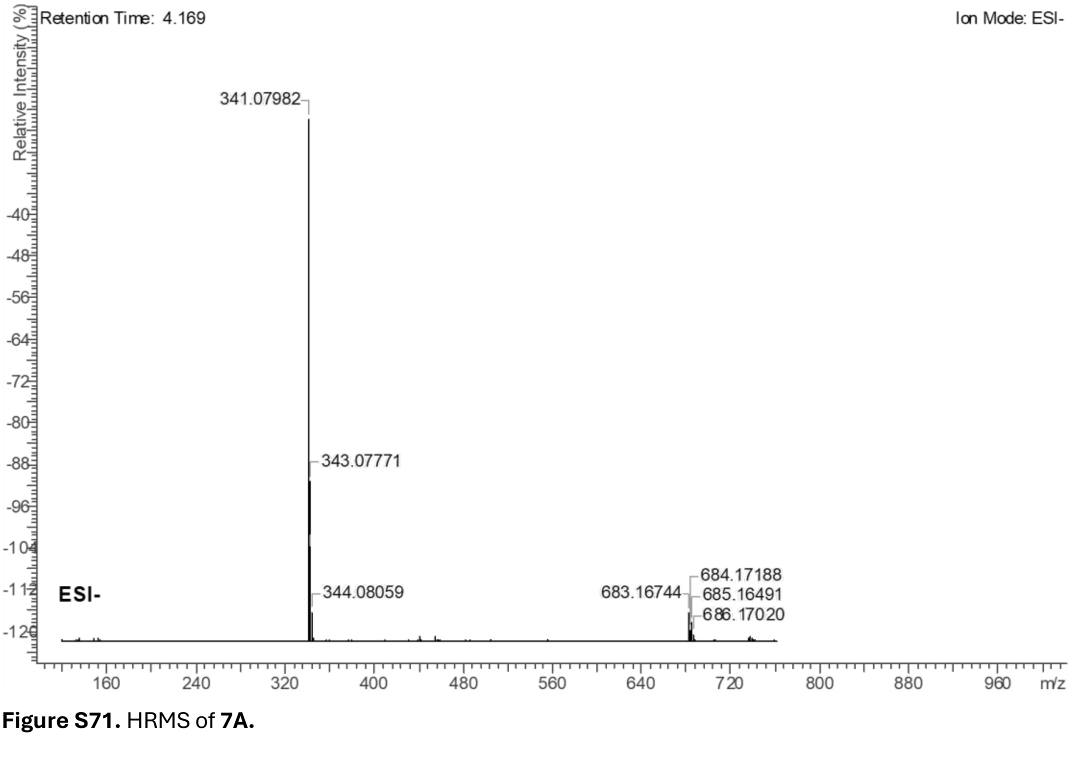

Figure S71. HRMS of **7A.**

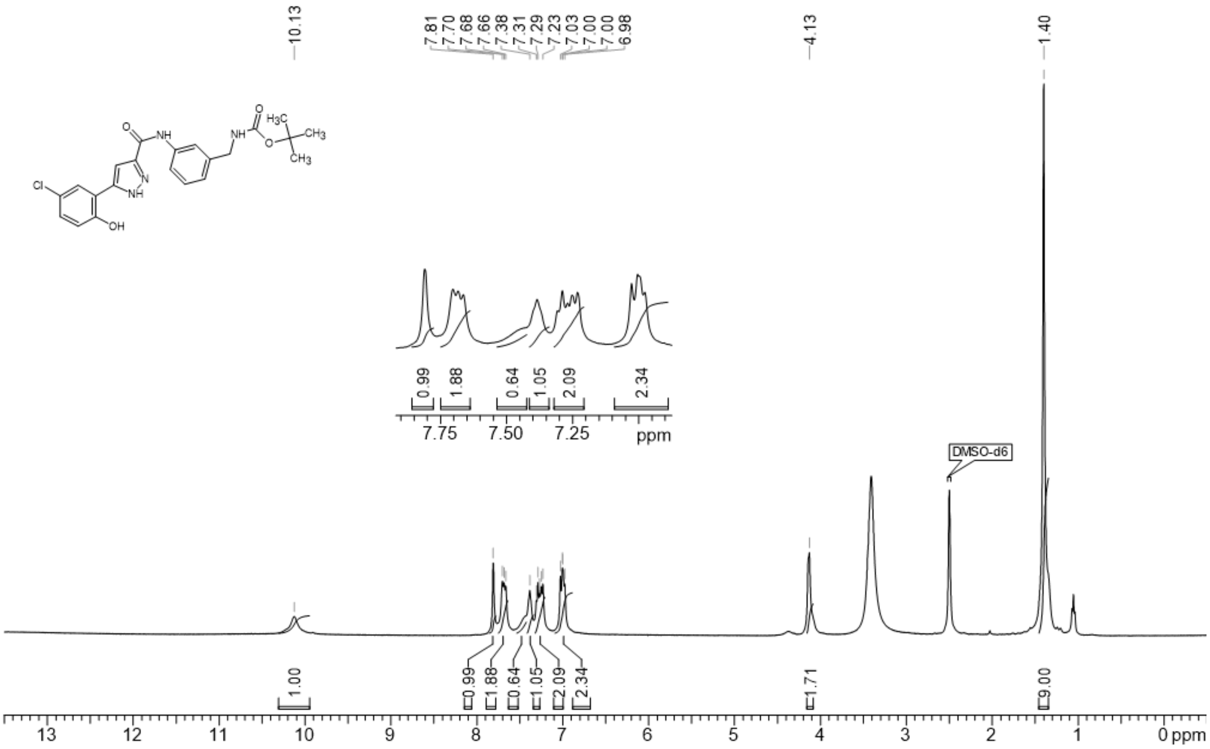

Figure S72. ^{1}H NMR spectrum of **7B.**

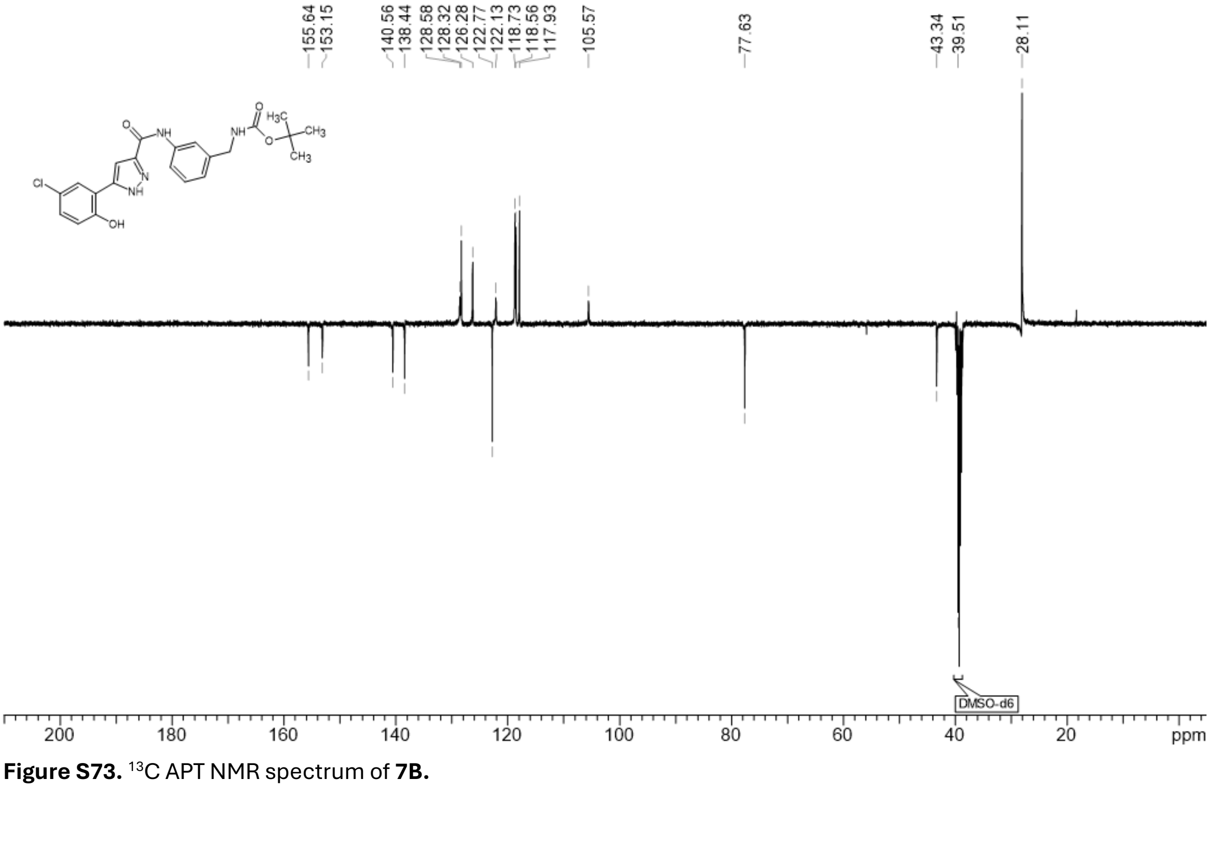

Figure S73. ^{13}C APT NMR spectrum of **7B.**

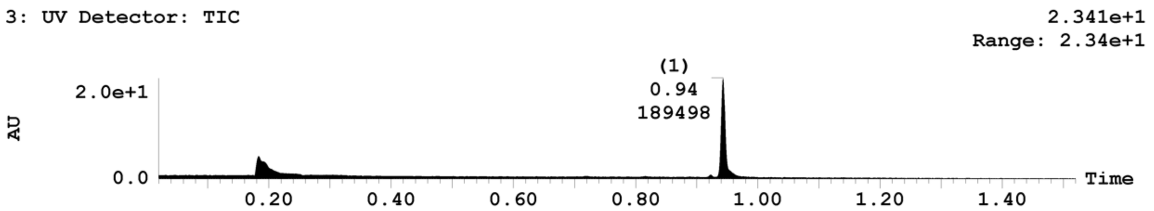

Peak Number	Time	Area %Total
1	0.94	100.00

Figure S74. LCMS purity analysis of **7B.**

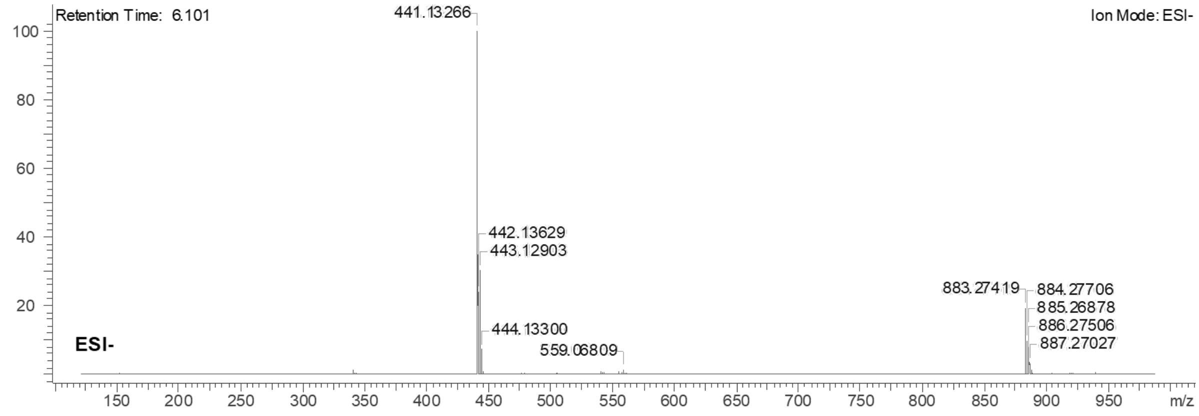

Figure S75. HRMS of **7B.**

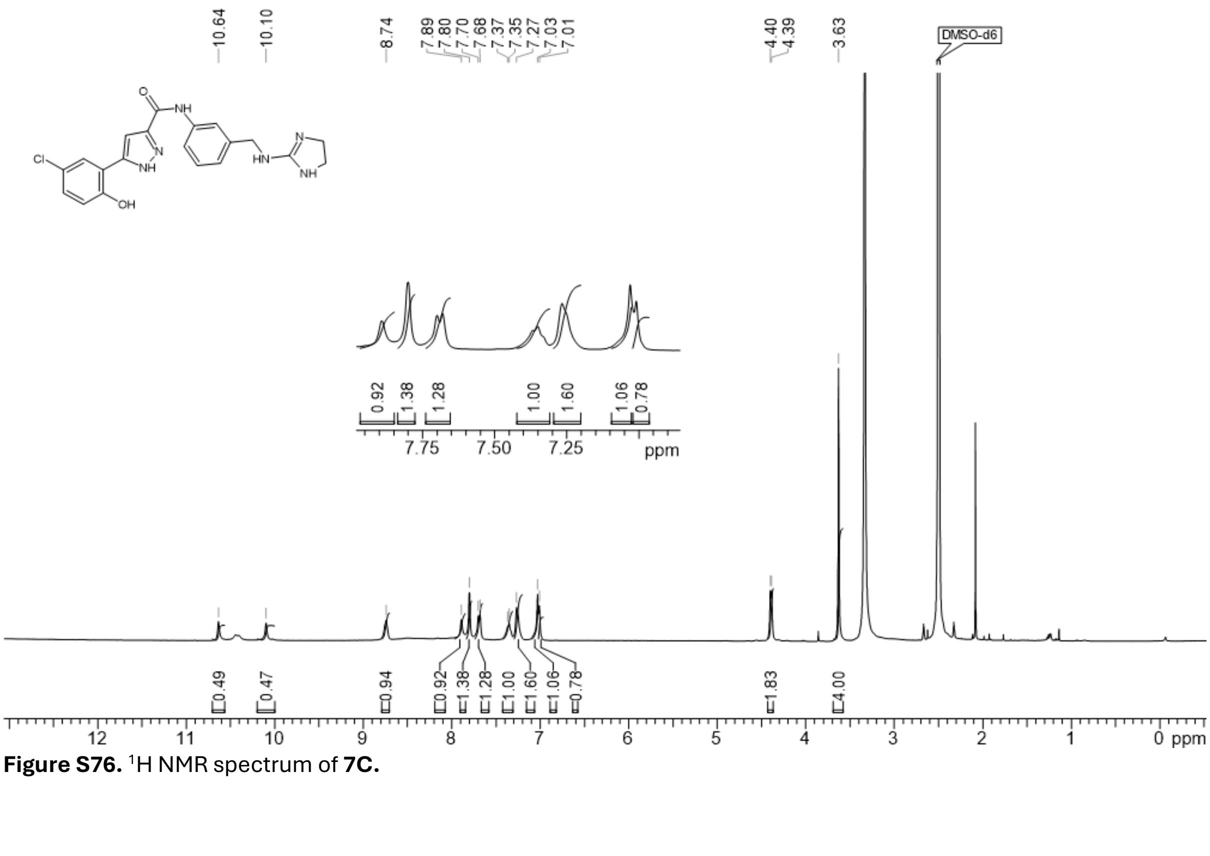

Figure S76. ^{1}H NMR spectrum of **7C.**

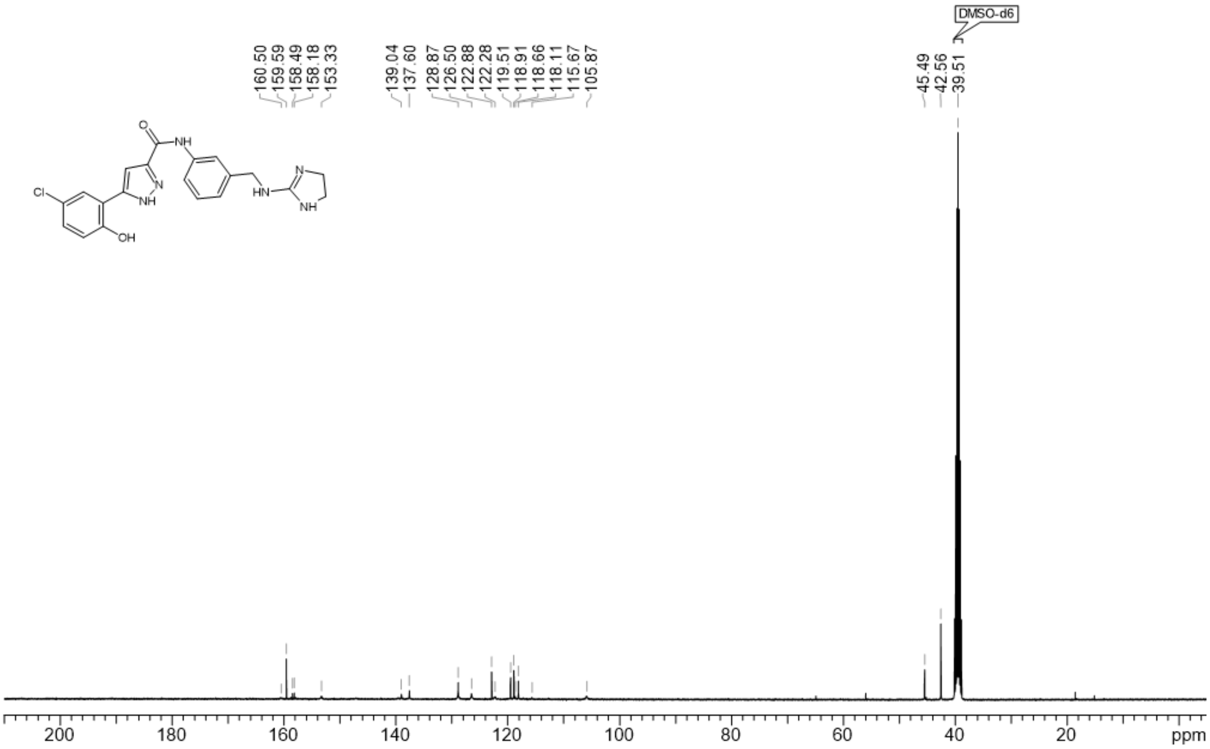

Figure S77. ^{13}C NMR spectrum of **7C.**

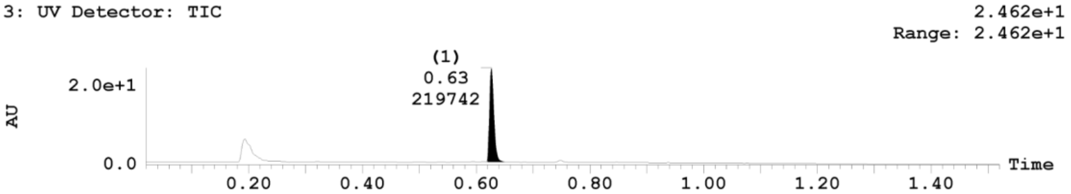

Peak Number	Time	Area %Total
1	0.63	100.00

Figure S78. LCMS purity analysis of **7C.**

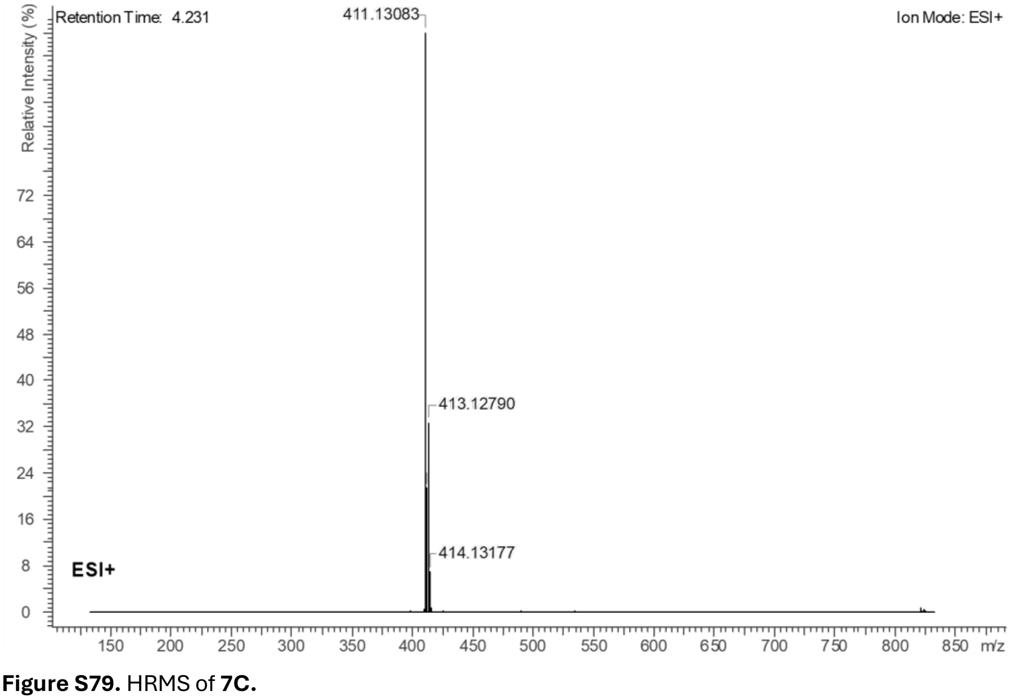

Figure S79. HRMS of **7C.**

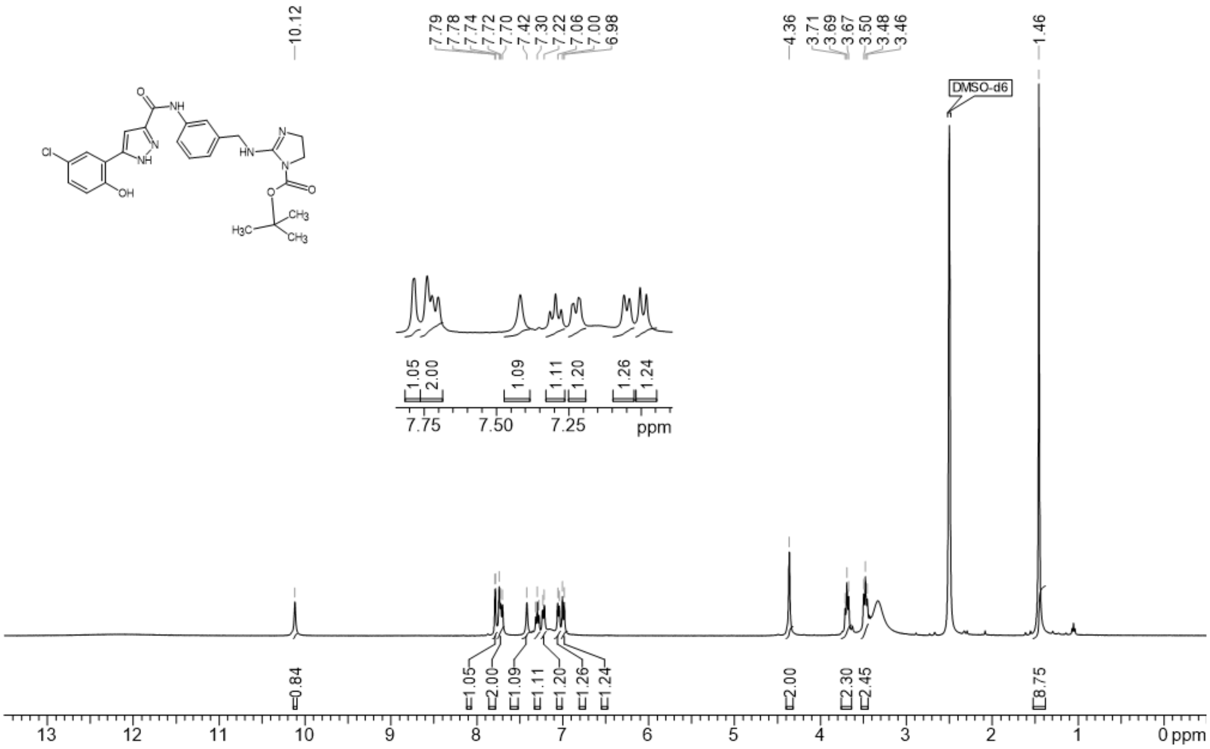

Figure S80. ^{1}H NMR spectrum of **7D.**

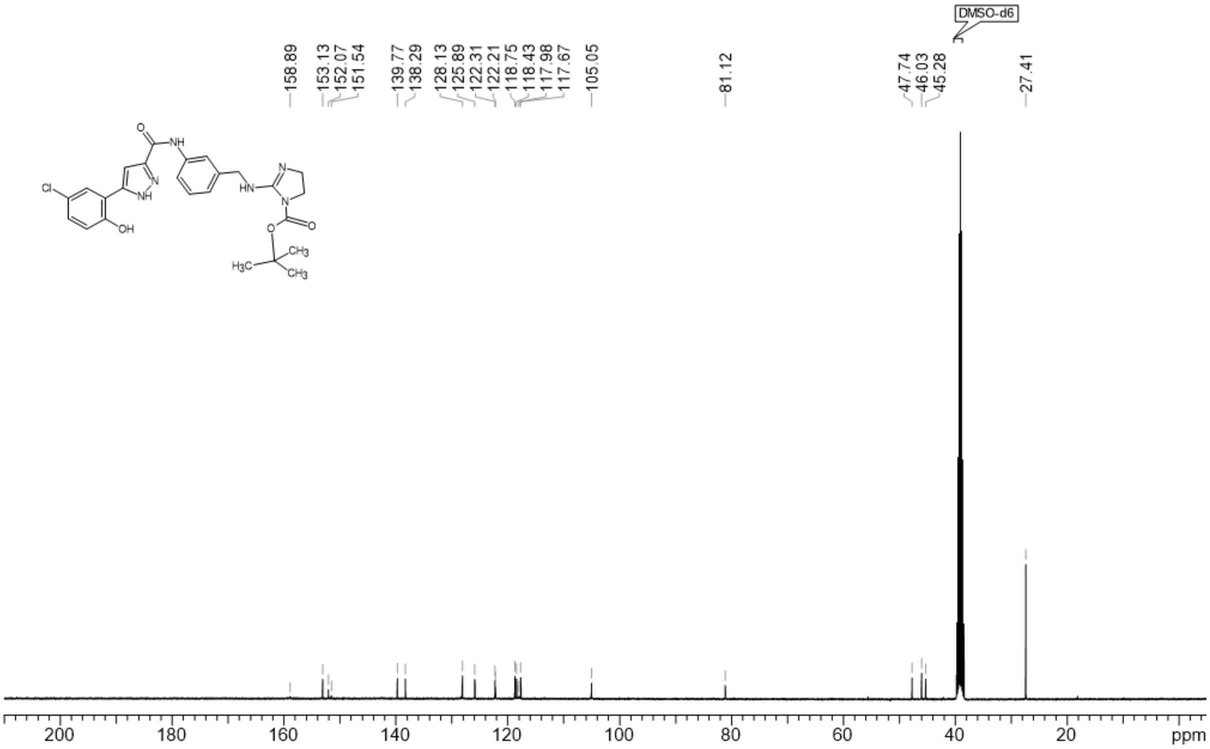

Figure S81. ^{13}C NMR spectrum of **7D.**

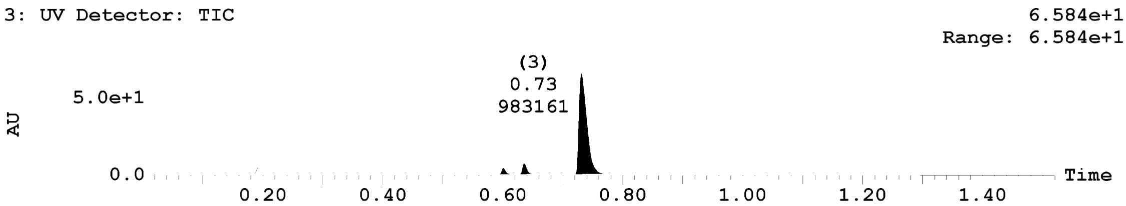

Peak Number	Time	Area %Total
1	0.60	2.56
2	0.64	5.01
3	0.73	92.42

Peak ID	Time	Mass Found	BPM
1	0.60		343.06

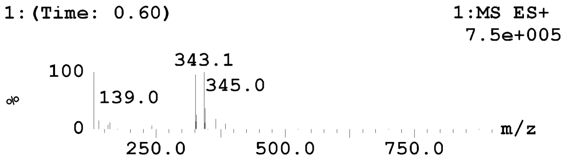

Peak ID	Time	Mass Found	BPM
1	0.60		409.30

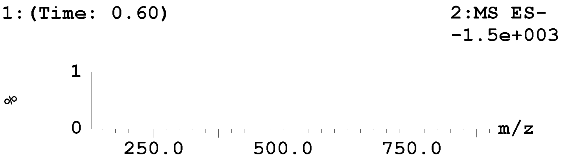

Peak ID	Time	Mass Found	BPM
2	0.64		411.07

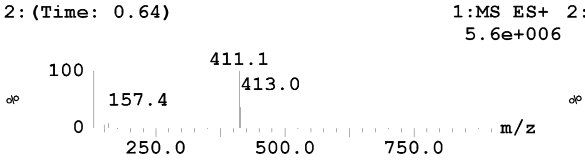

Peak ID	Time	Mass Found	BPM
2	0.64		409.26

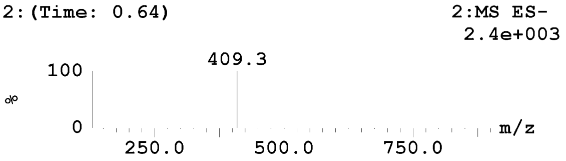

Peak ID	Time	Mass Found	BPM
3	0.73	510.18	511.07

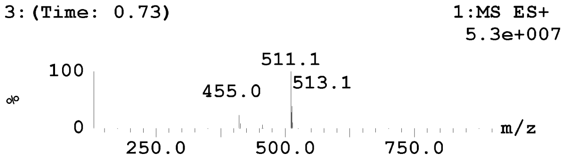

Peak ID	Time	Mass Found	BPM
3	0.73	510.18	509.15

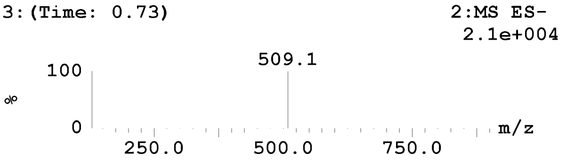

Figure S82. LCMS purity analysis of **7D**, 5% impurity of **7C.**

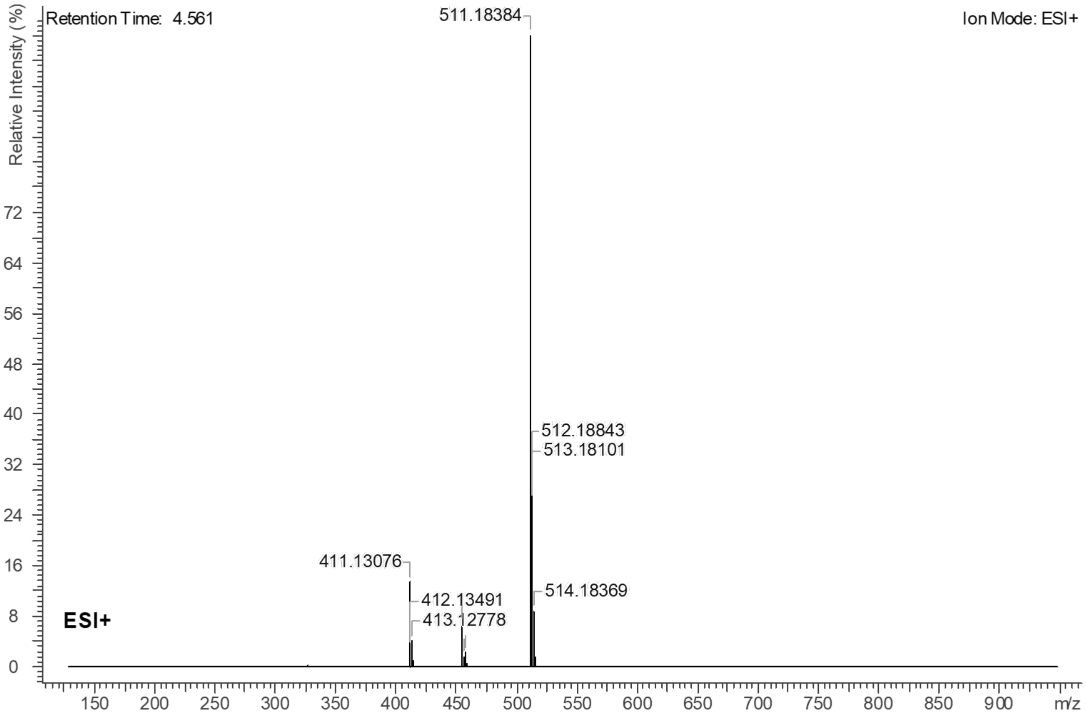

Figure S83. HRMS of **7D.**

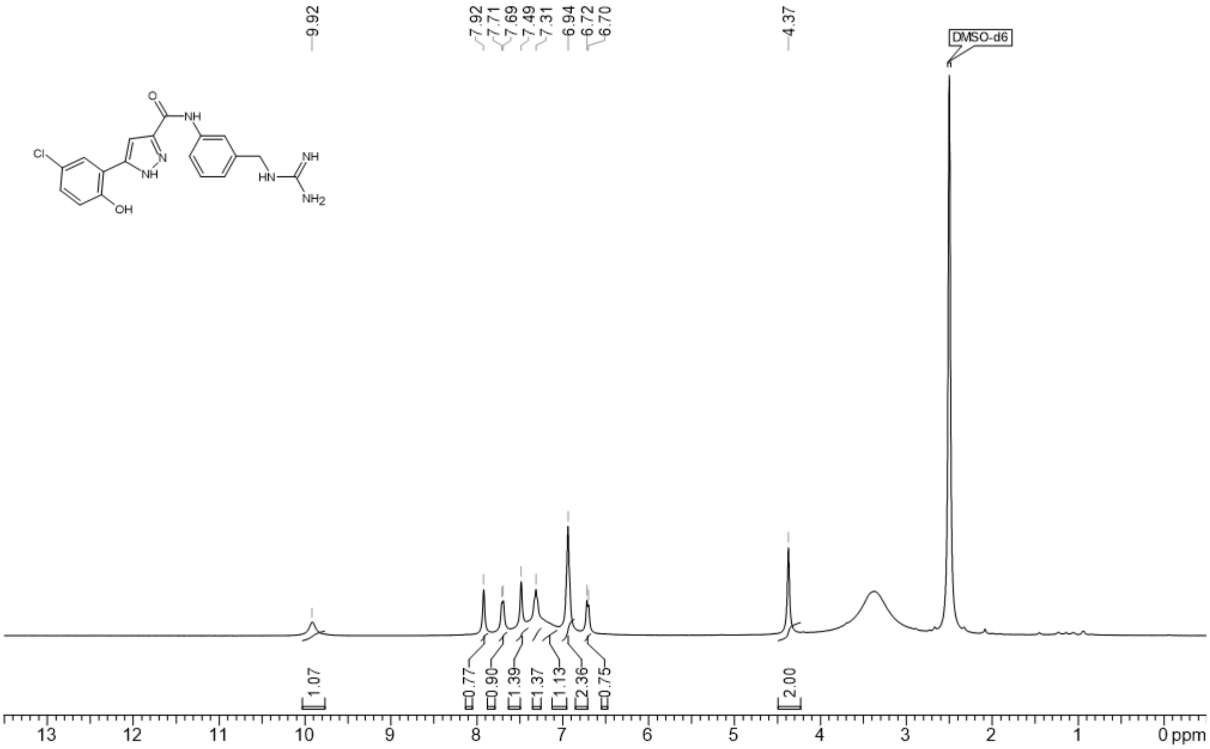

Figure S84. ^{1}H NMR spectrum of **7G.**

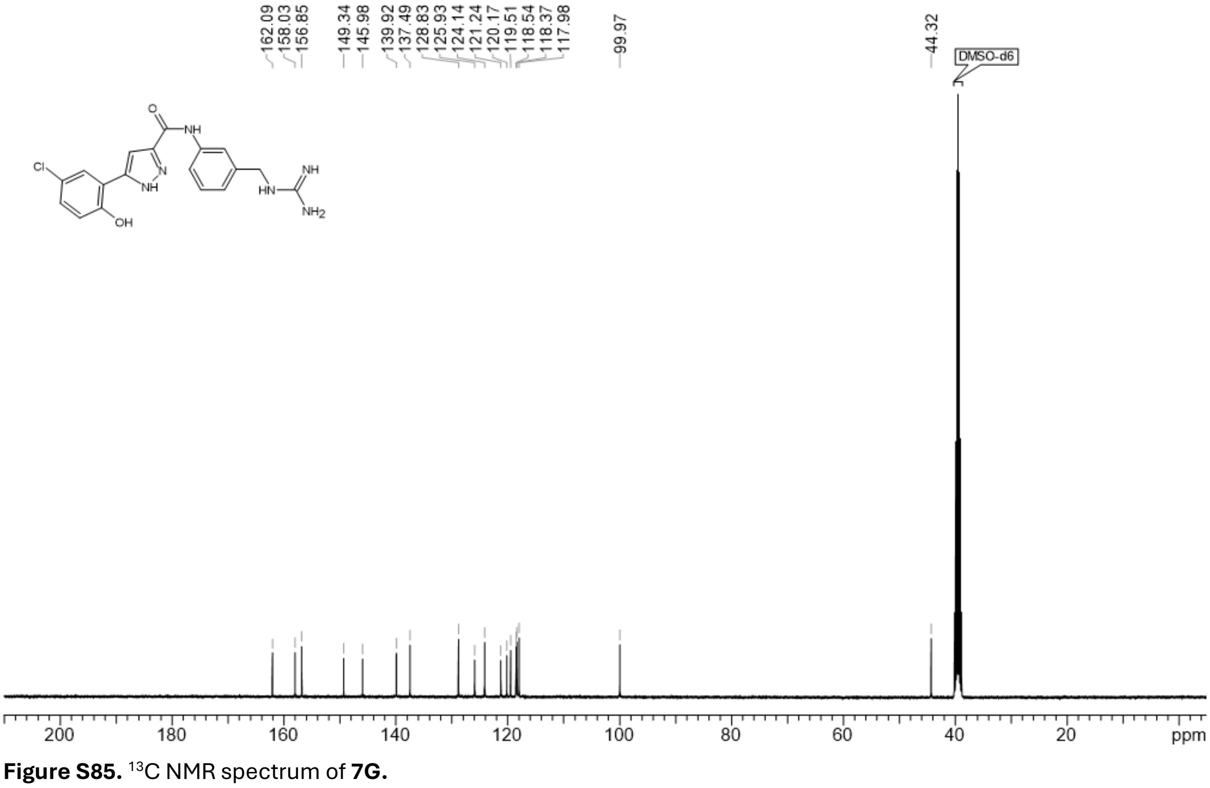

Figure S85. ^{13}C NMR spectrum of **7G.**

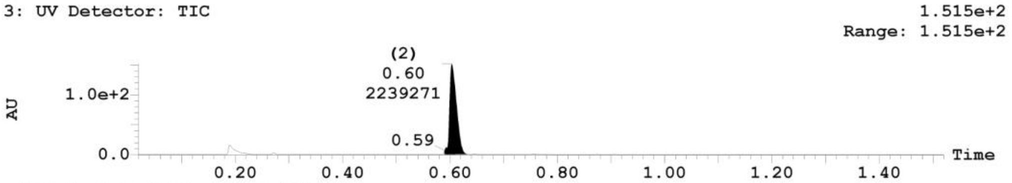

Peak Number	Time	Area %Total
1	0.59	2.13
2	0.60	97.87

Figure S86. LCMS purity analysis of **7G.**

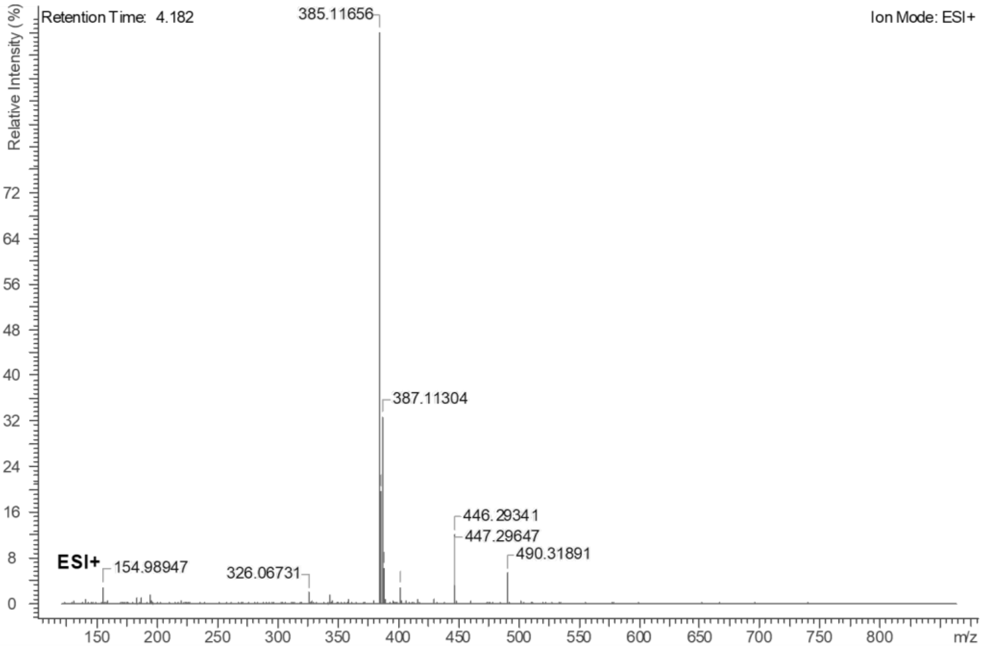

Figure S87. HRMS of **7G.**

Compounds **8A –G**

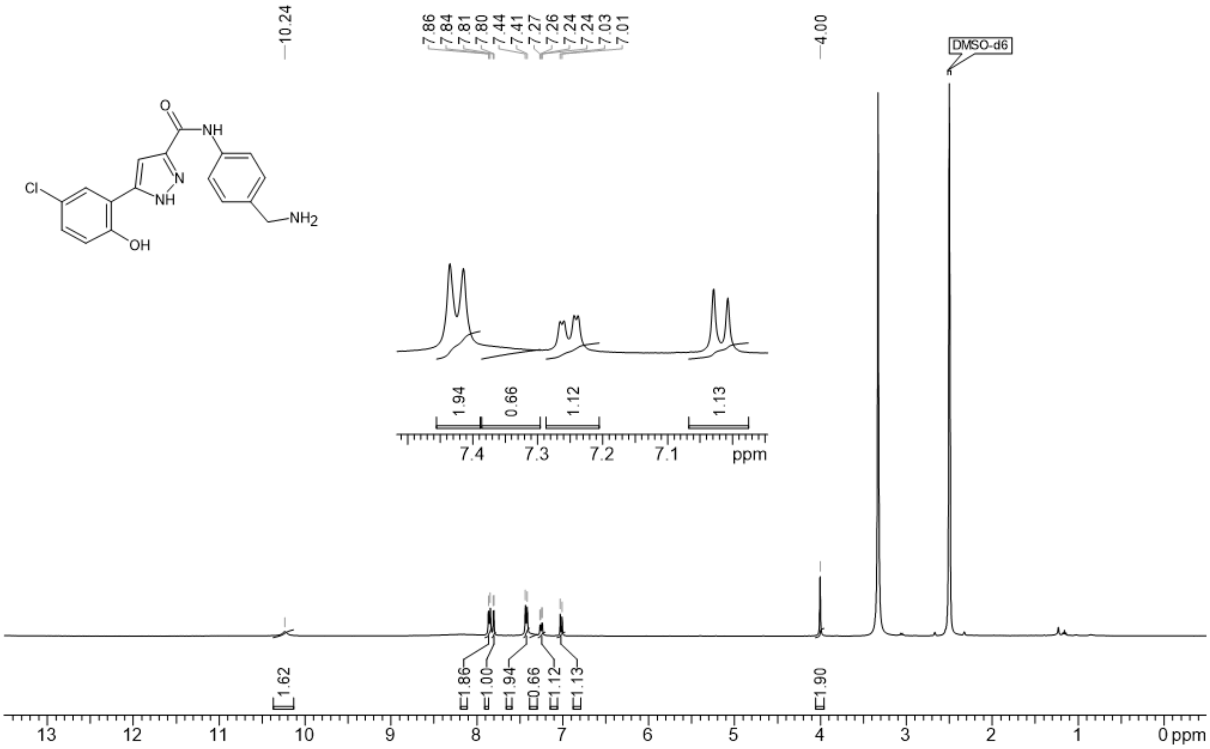

Figure S88. ^{1}H NMR spectrum of **8A** at 25 °C, C–H of pyrazole very broad at 7.36 ppm**.**

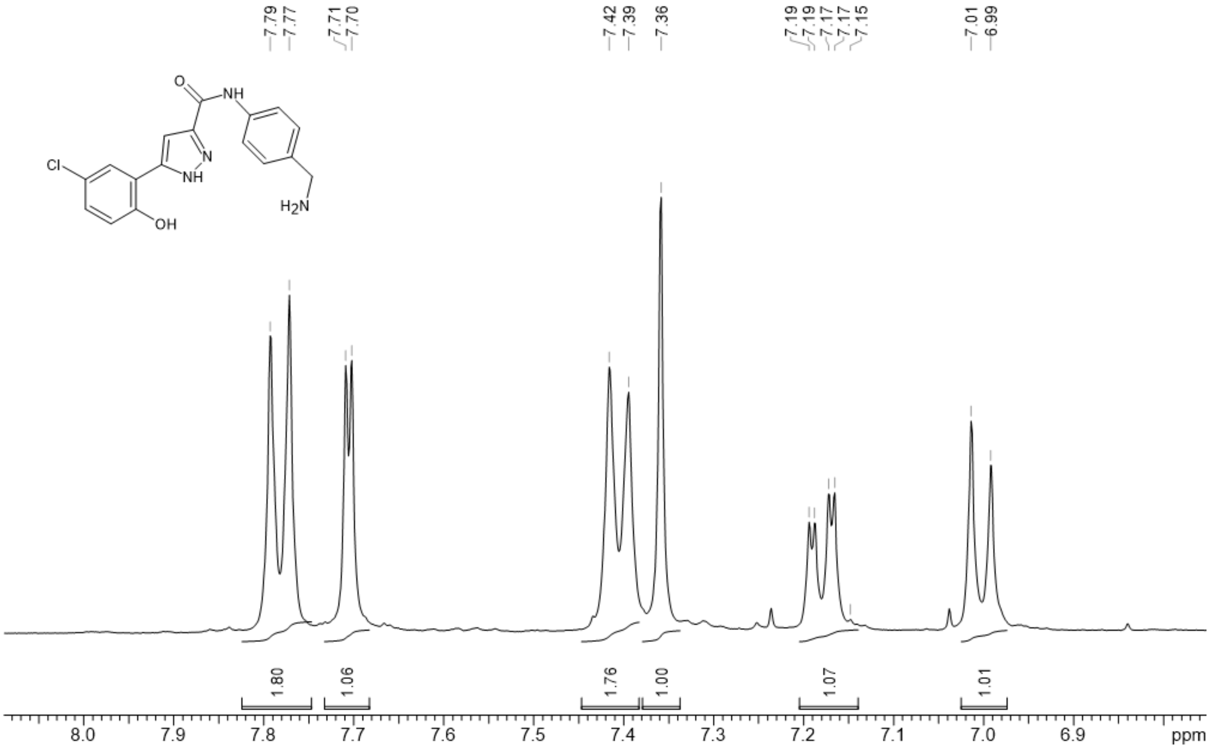

Figure S89. ^{1}H NMR spectrum of **8A** at 120 °C, C–H of pyrazole at 7.36 ppm**.**

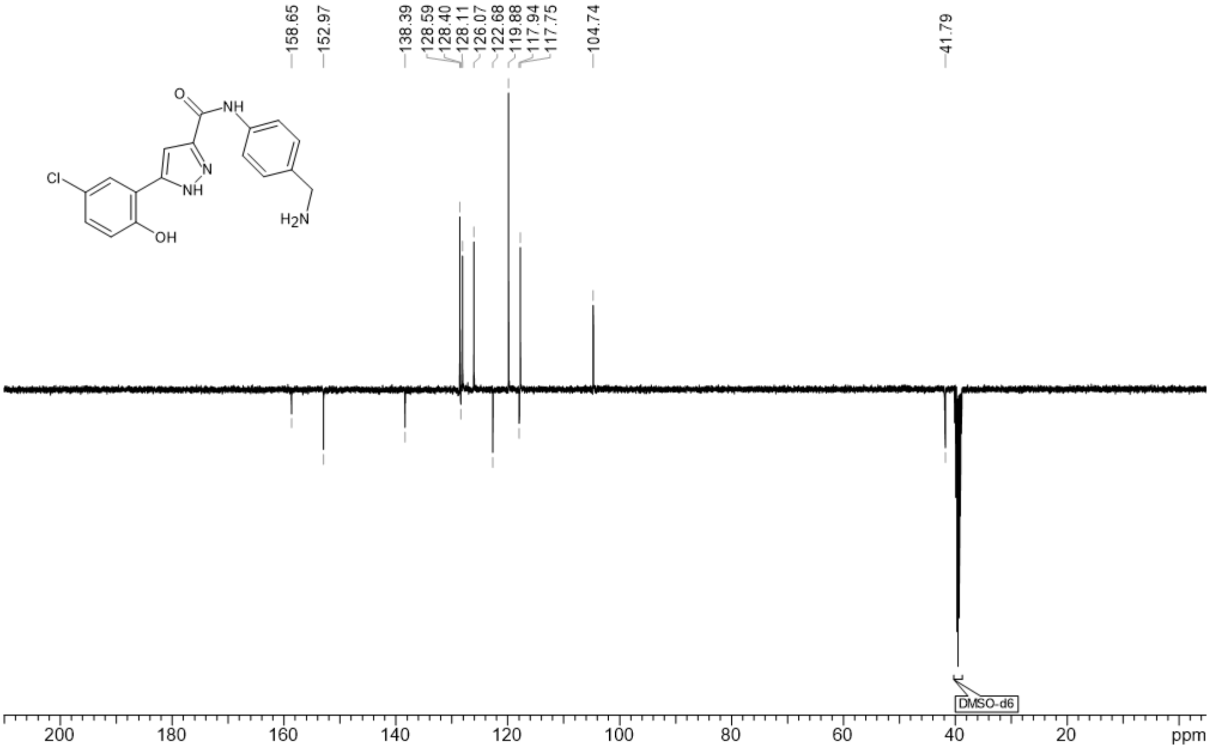

Figure S90. ^{13}C APT NMR spectrum of **8A**.

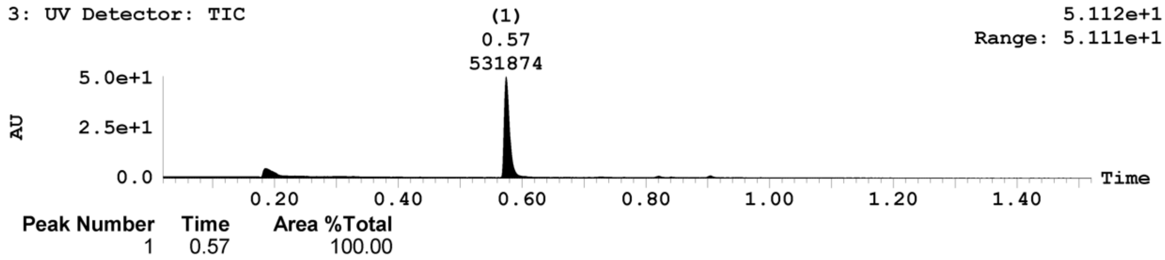

Figure S91. LCMS purity analysis of **8A**.

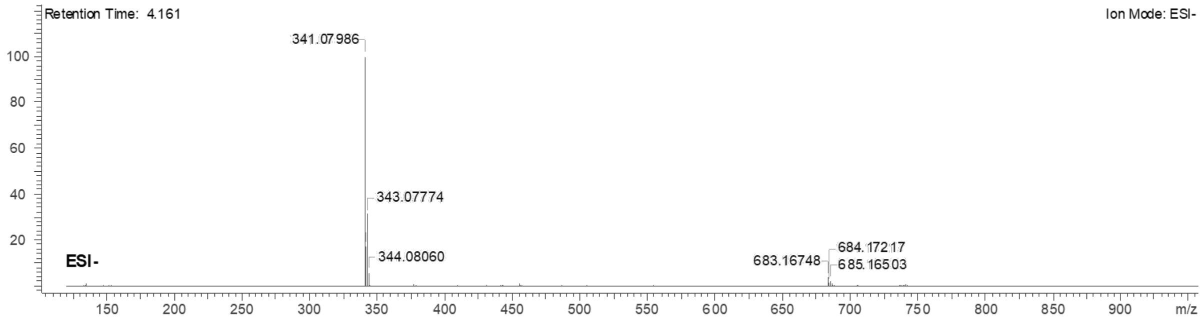

Figure S92. HRMS of **8A.**

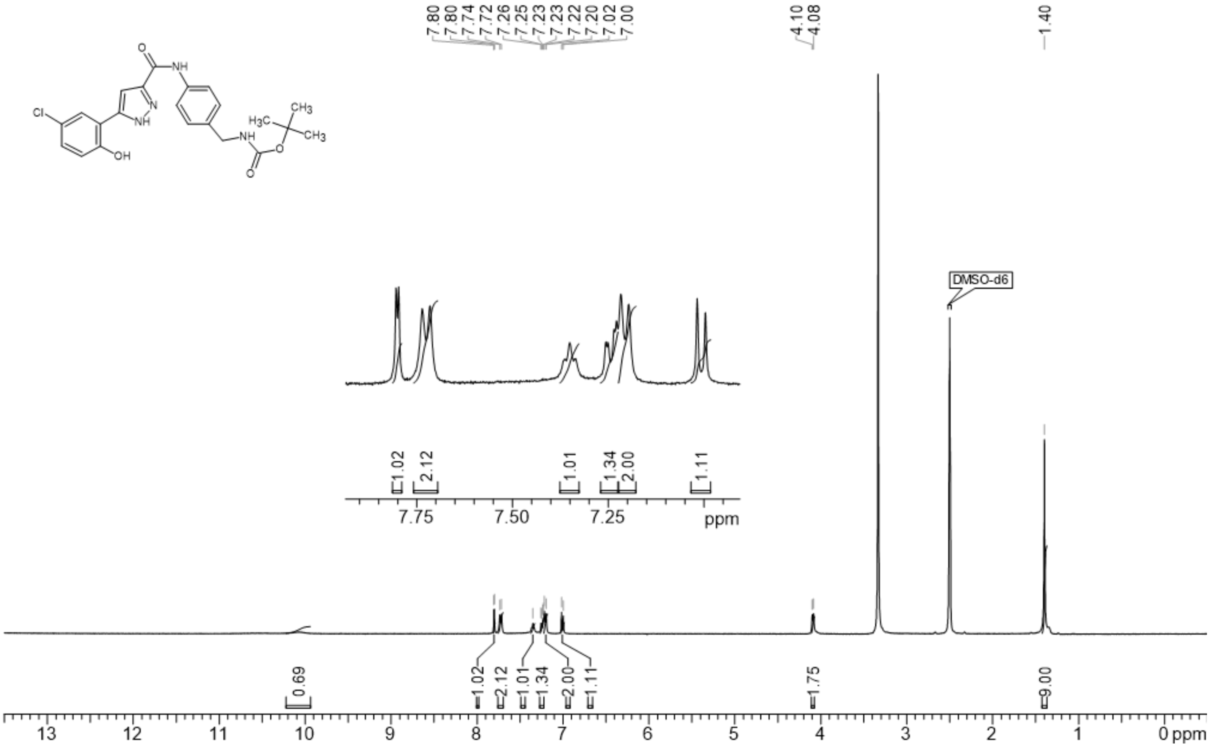

Figure S93. ^{1}H NMR spectrum of **8B.**

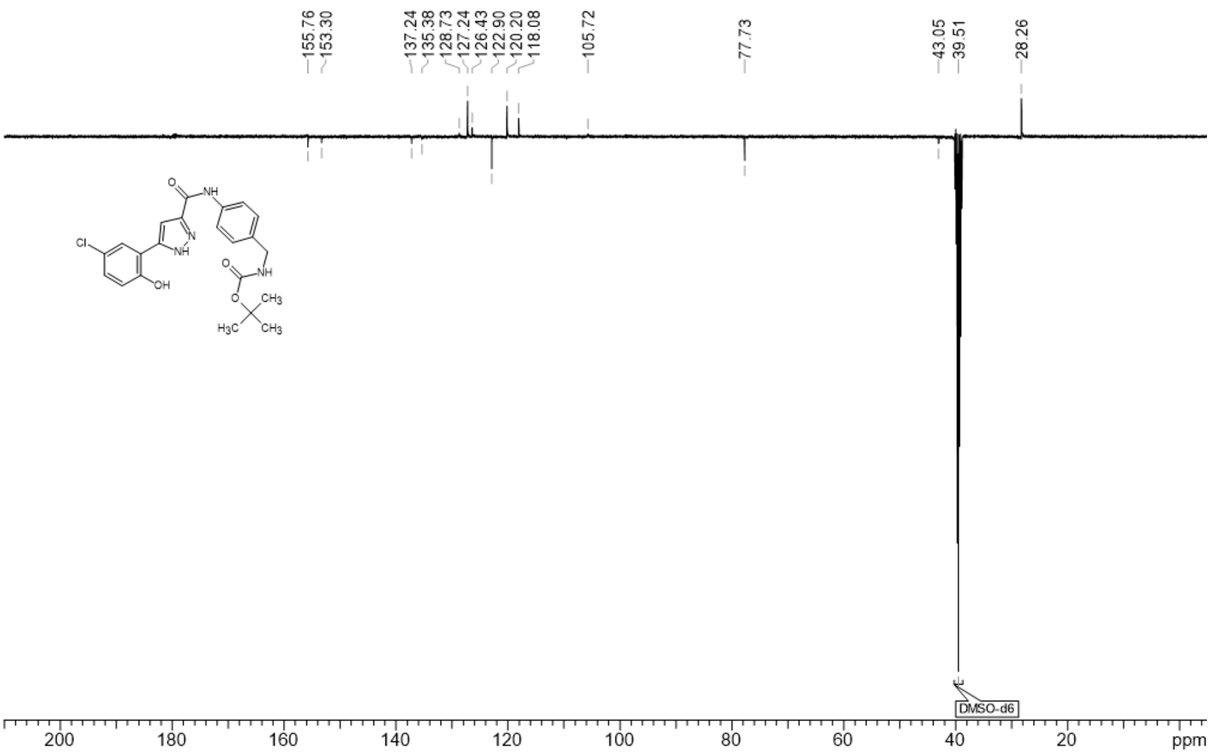

Figure S94. ^{13}C APT NMR spectrum of **8B**.

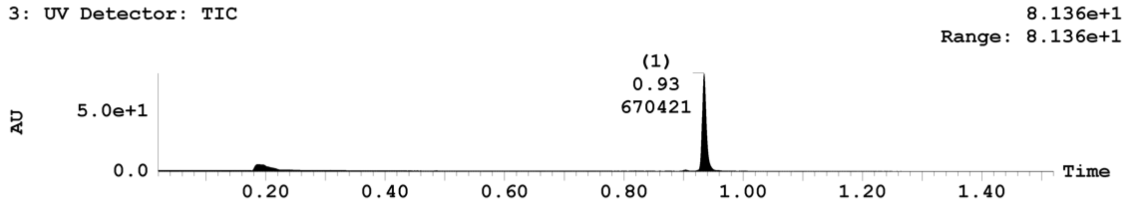

Peak Number	Time	Area %Total
1	0.93	100.00

Figure S95. LCMS purity analysis of **8B.**

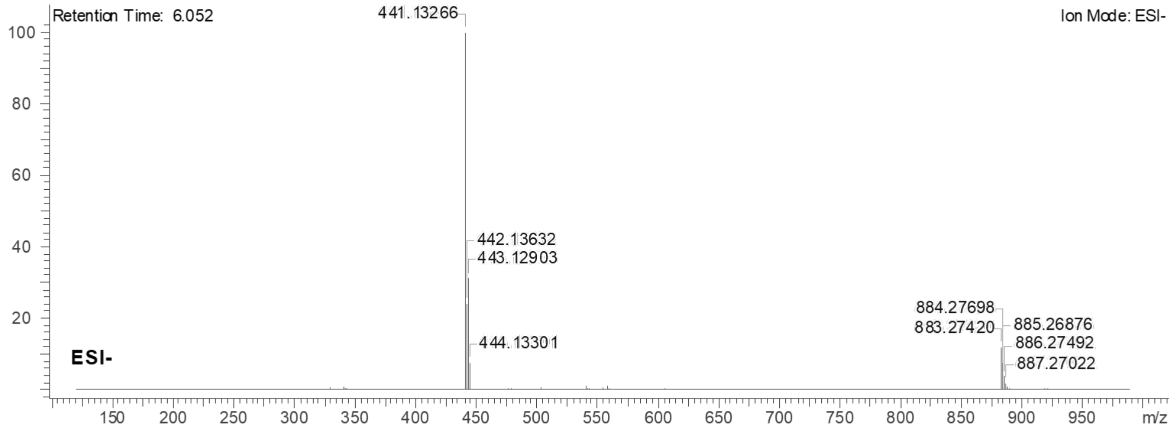

Figure S96. HRMS of **8B.**

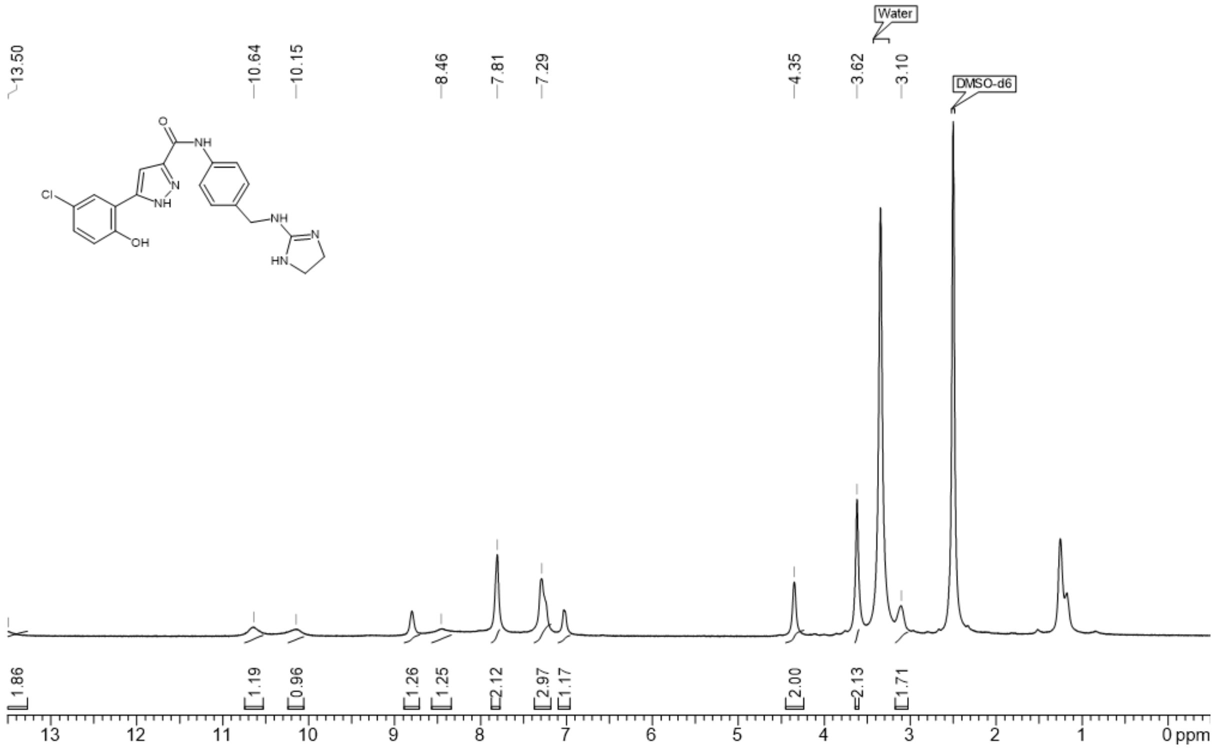

Figure S97. ^{1}H NMR spectrum of **8C.**

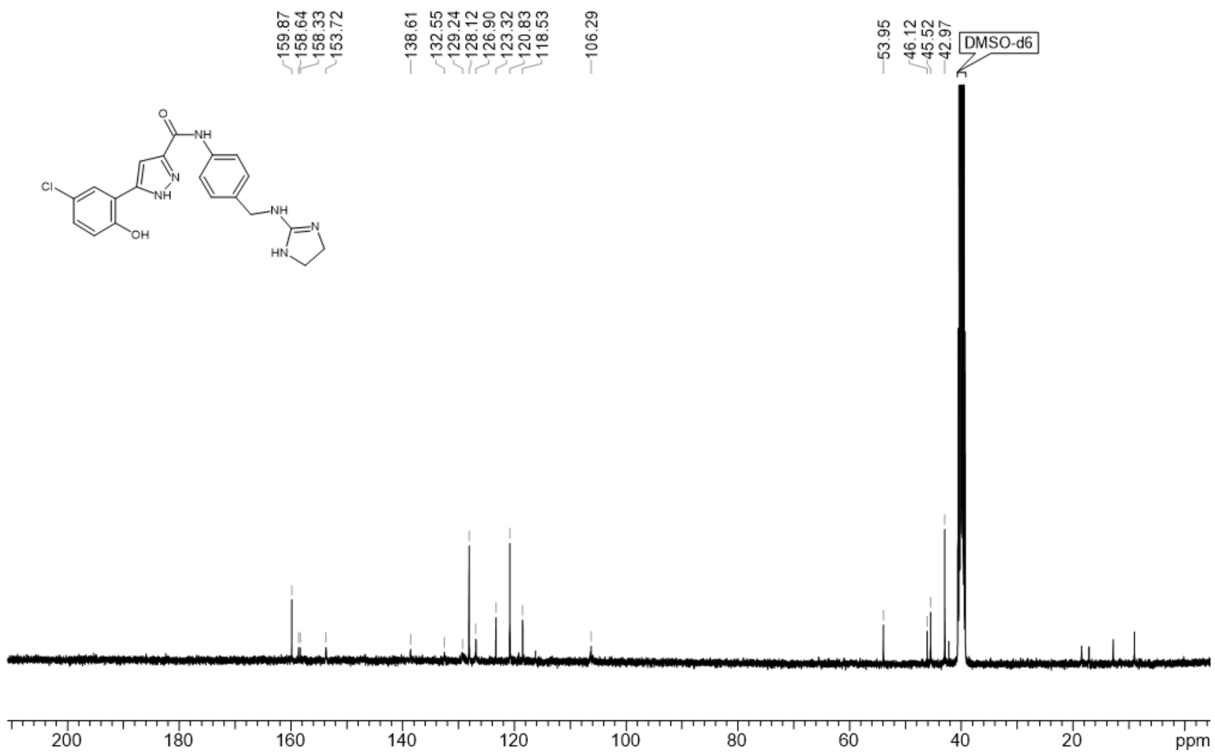

Figure S98. ^{13}C NMR spectrum of **8C.**

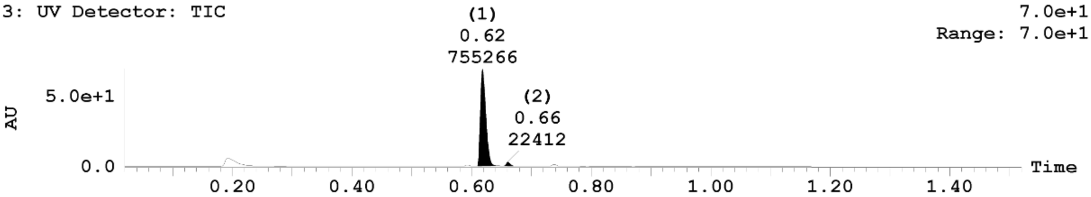

Peak Number	Time	Area %Total
1	0.62	97.12
2	0.66	2.88

Figure S99. LCMS purity analysis of **8C**.

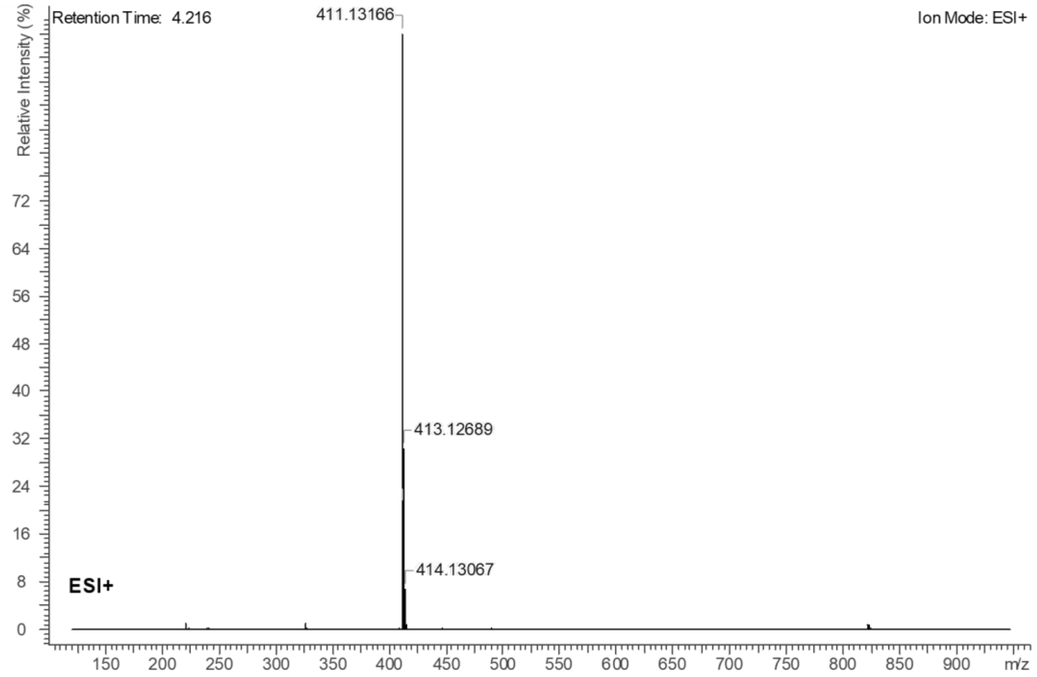

Figure S100. HRMS of **8C**.

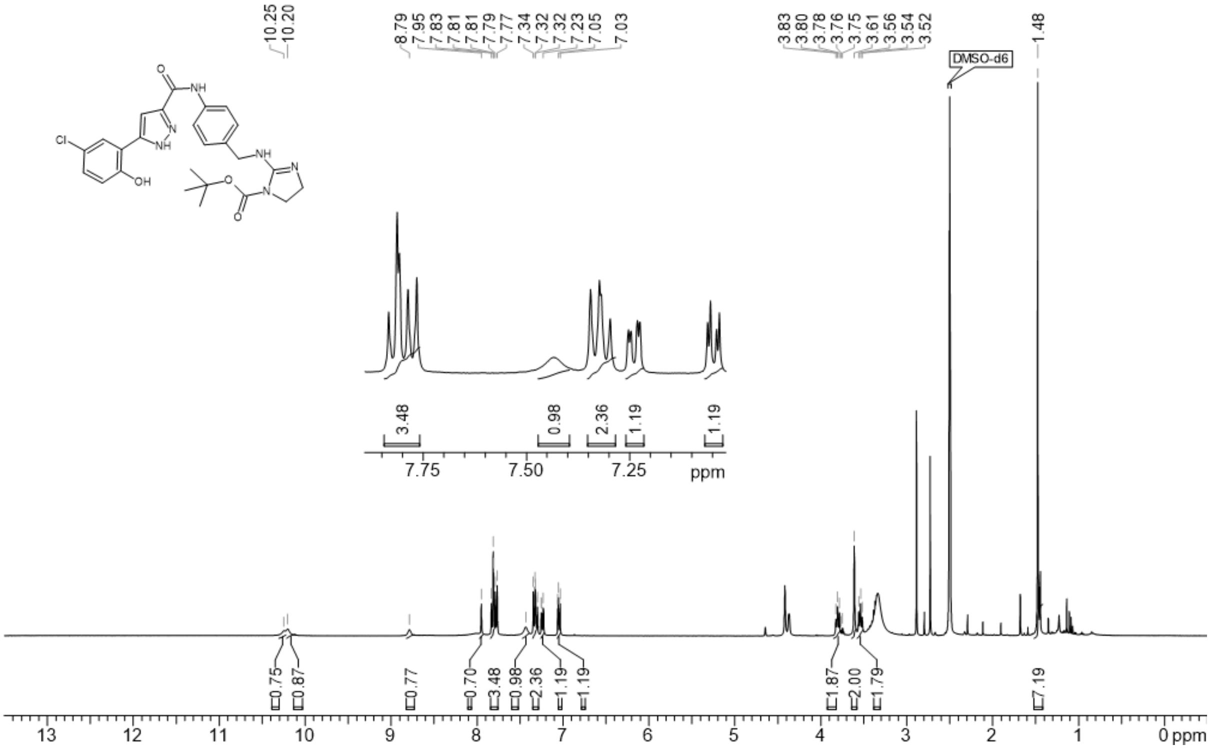

Figure S101. ^{1}H NMR spectrum of **8D**.

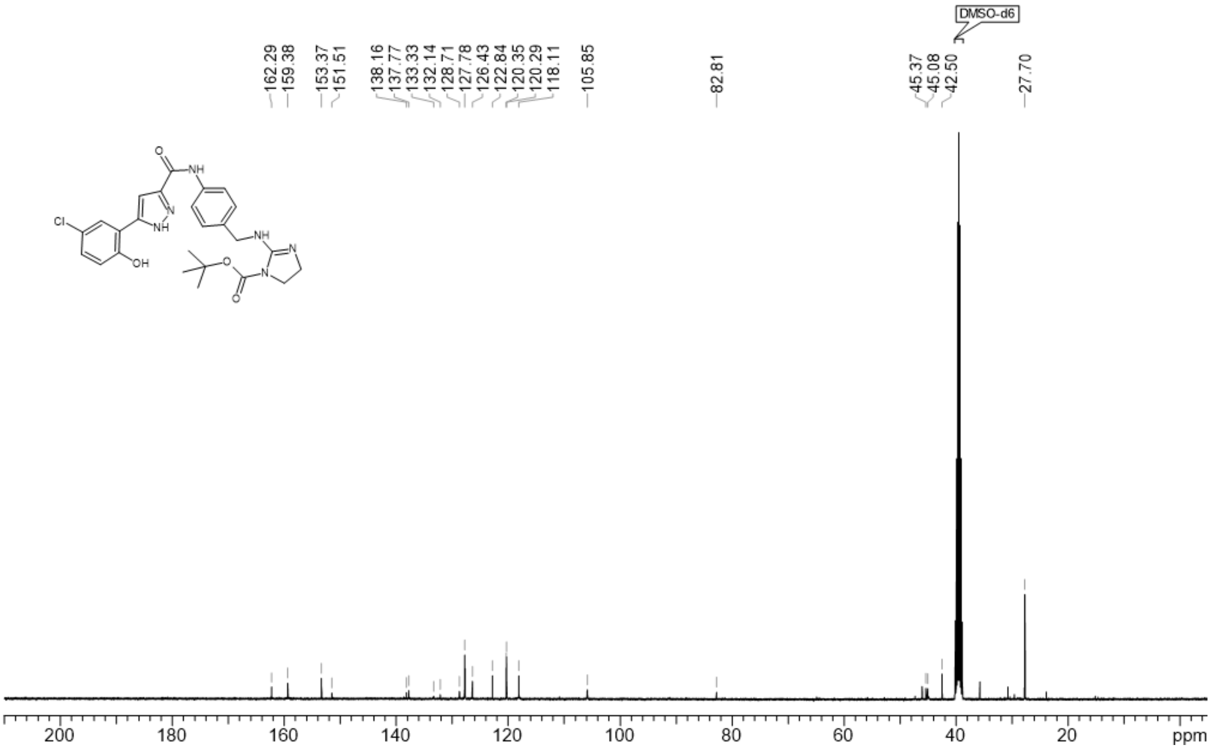

Figure S102. ^{13}C NMR spectrum of **8D.**

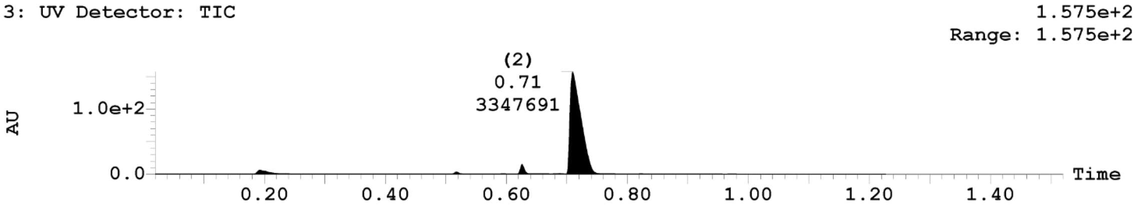

Peak Number	Time	Area %Total
1	0.63	3.35
2	0.71	96.65

Figure S103. LCMS purity analysis of **8D**.

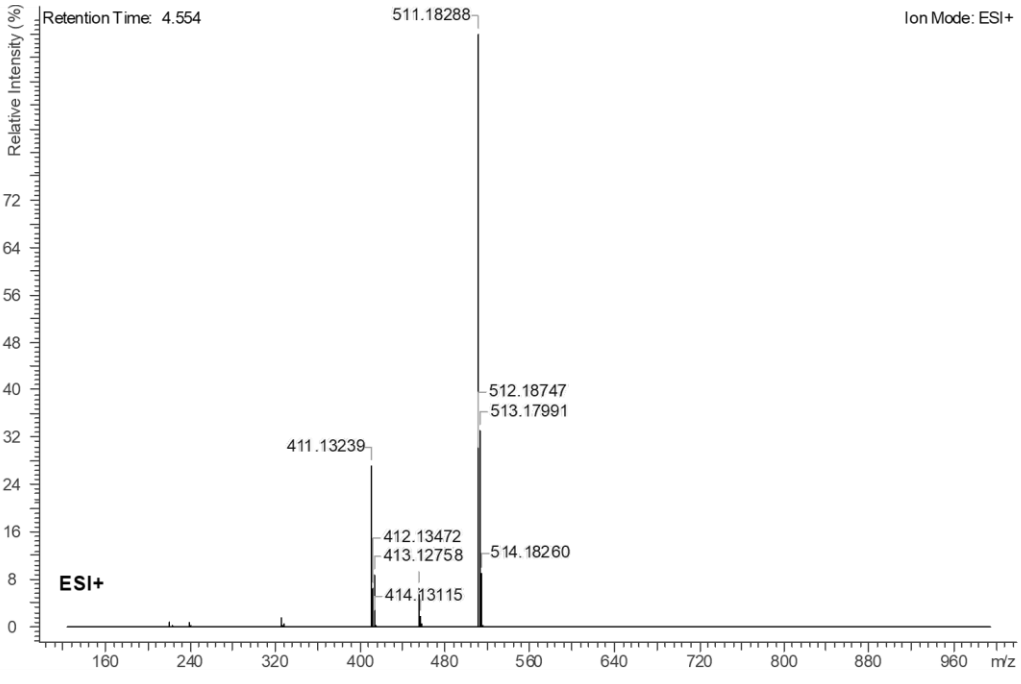

Figure S104. HRMS of **8D**.

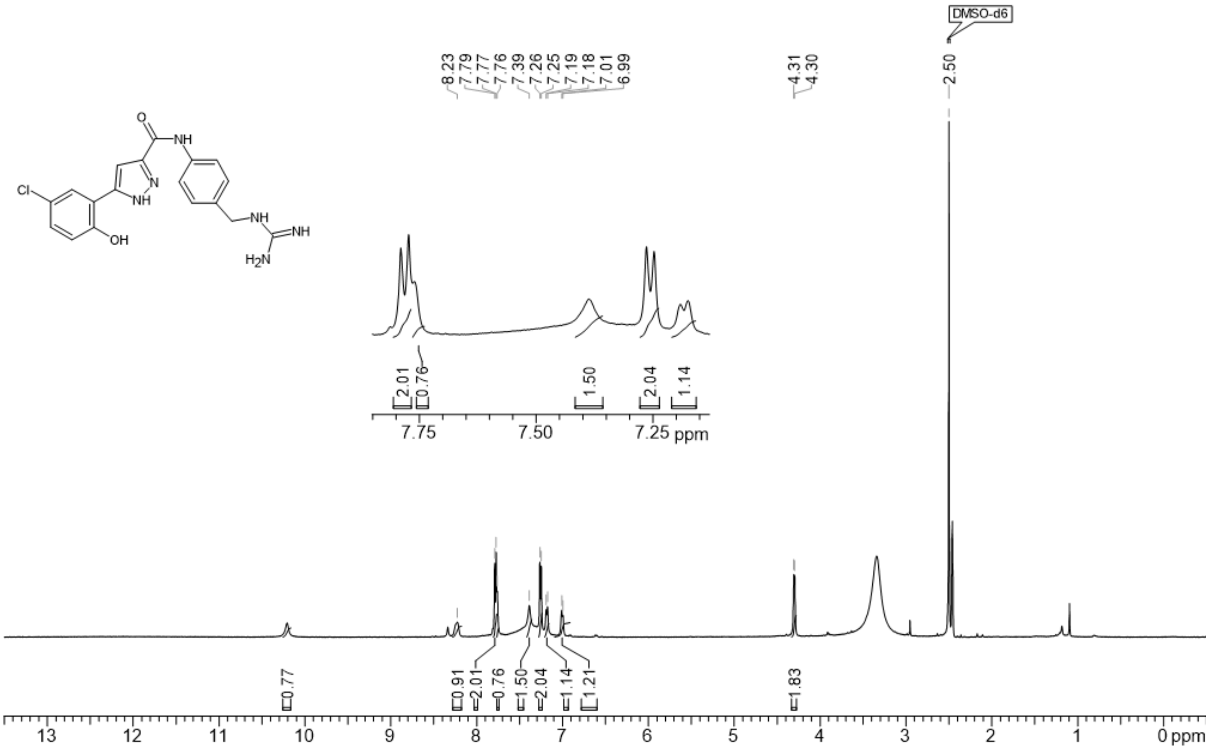

Figure S105. ^{1}H NMR spectrum of **8G.**

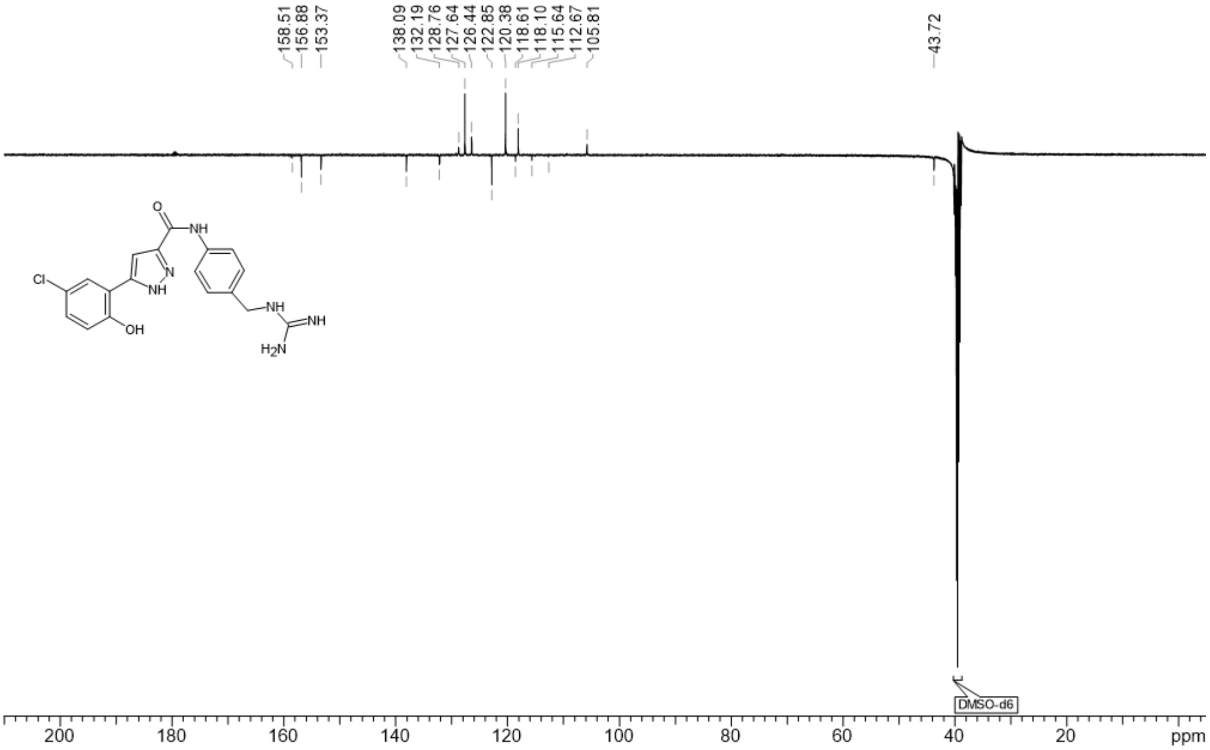

Figure S106. ^{13}C APT NMR spectrum of **8G.**

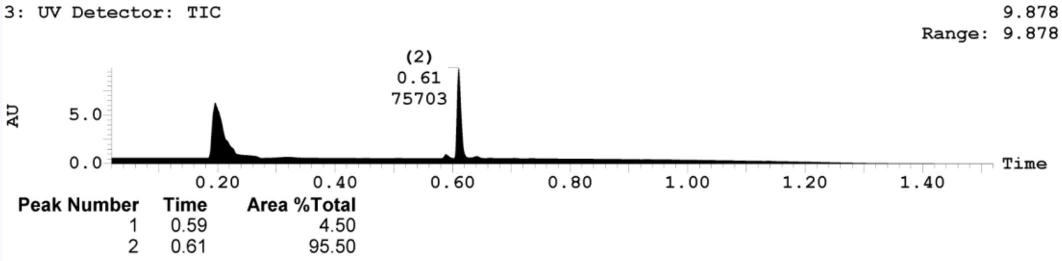

Figure S107. LCMS purity analysis of **8G**.

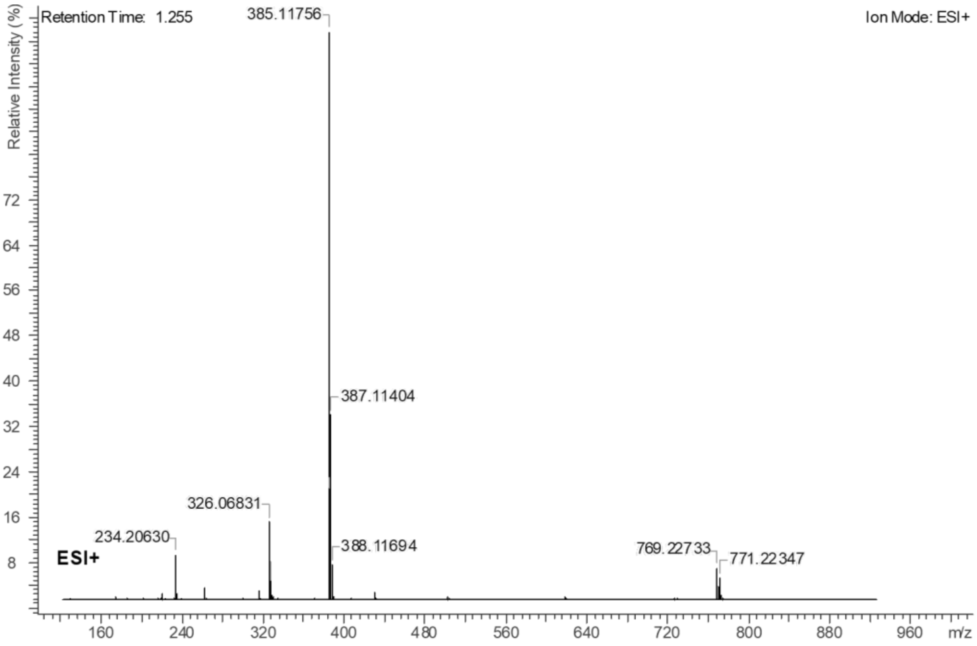

Figure S108. HRMS of **8G.**

Compounds **9A–G**

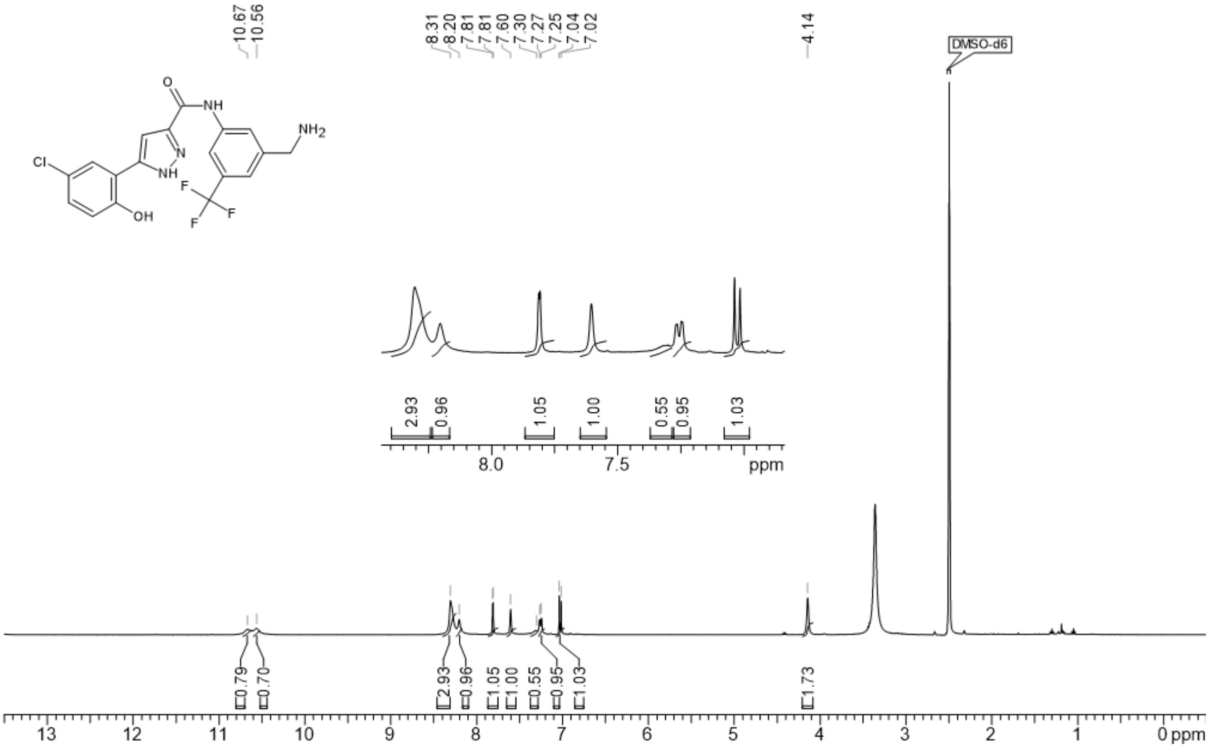

Figure S109. ^{1}H NMR spectrum of **9A.**

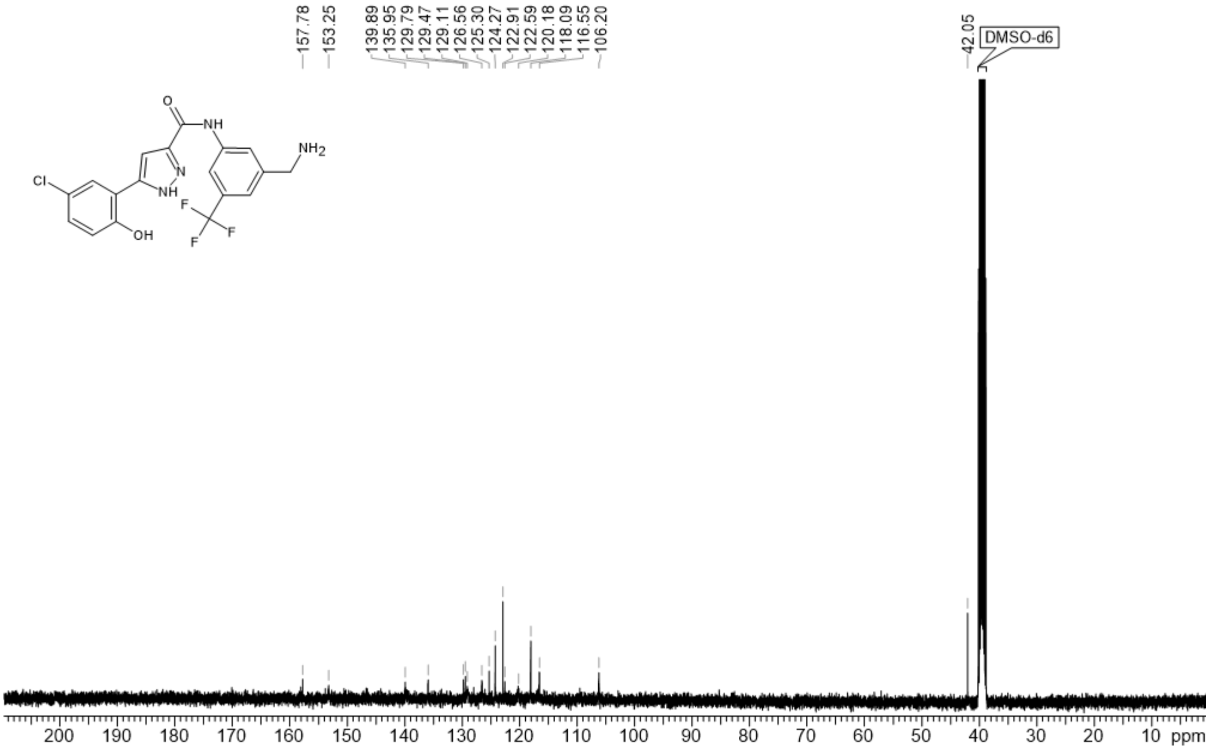

Figure S110. ^{13}C NMR spectrum of **9A.**

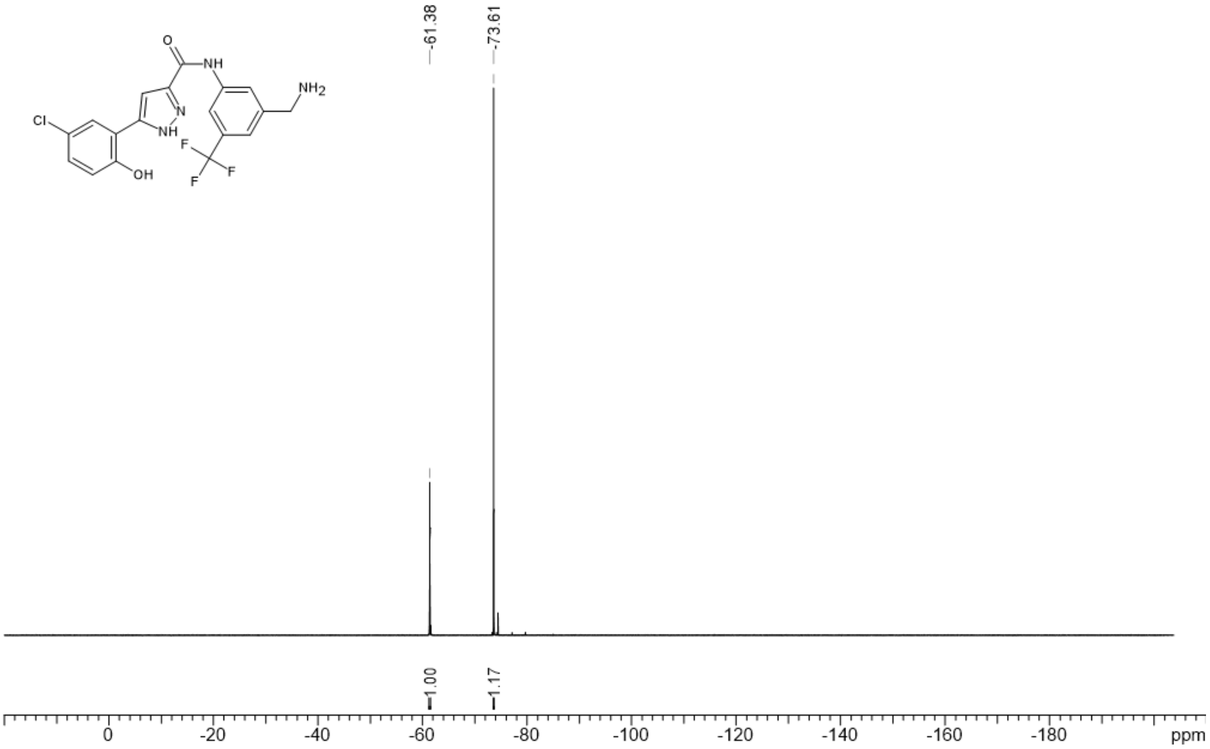

Figure S111. ^{19}F NMR spectrum of **9A.**

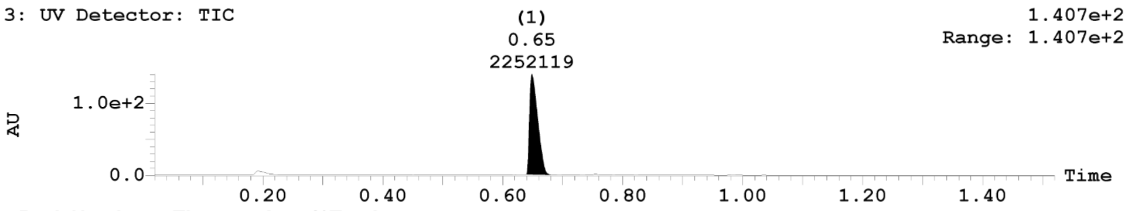

Peak Number	Time	Area %Total
1	0.65	100.00

Figure S112. LCMS purity analysis of **9A.**

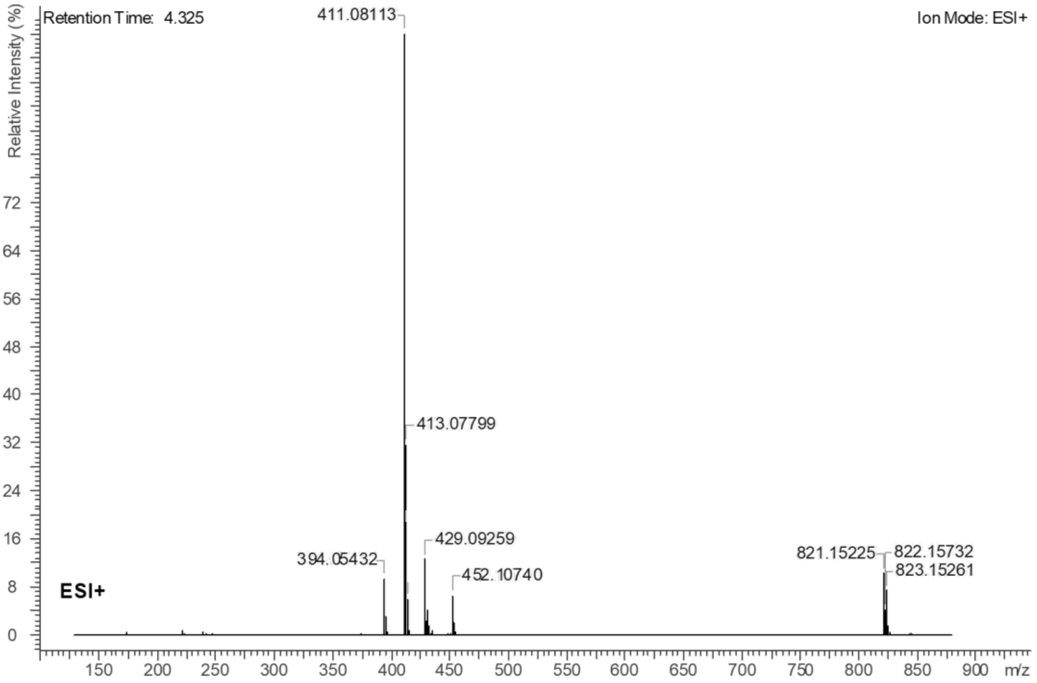

Figure S113. HRMS of **9B**.

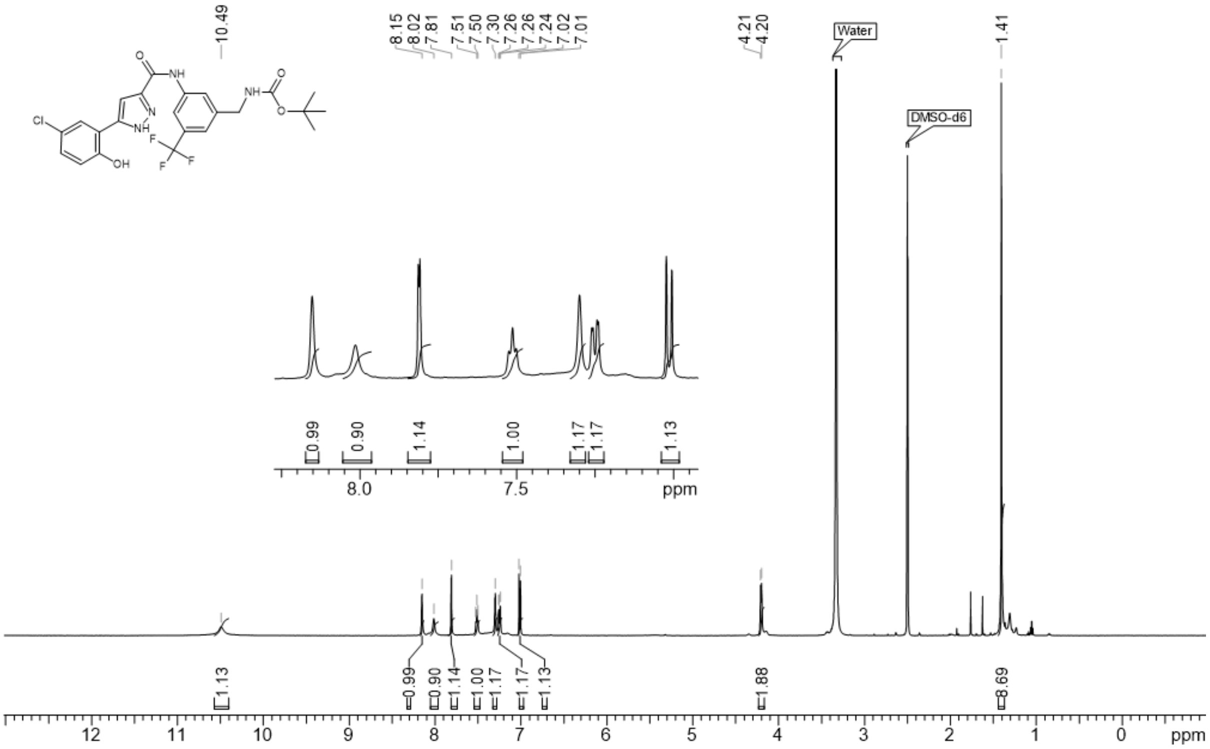

Figure S114. ^{1}H NMR spectrum of **9B.**

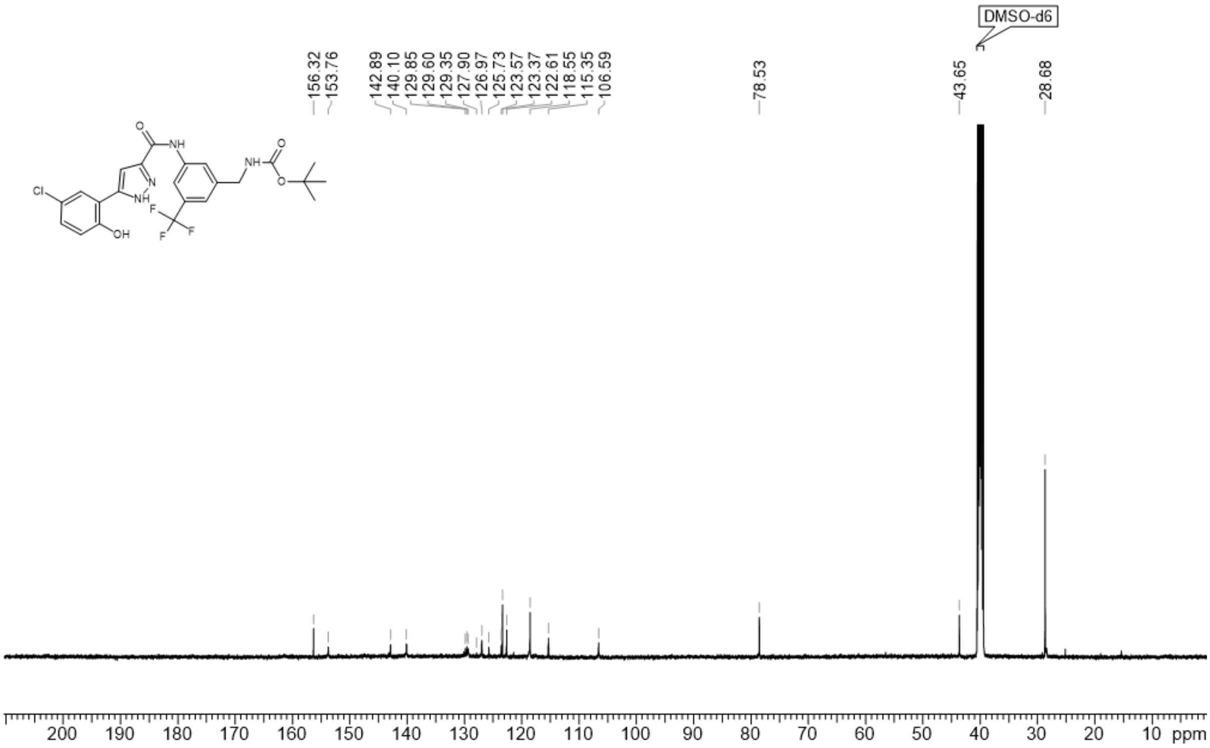

Figure S115. ^{13}C NMR spectrum of **9B.**

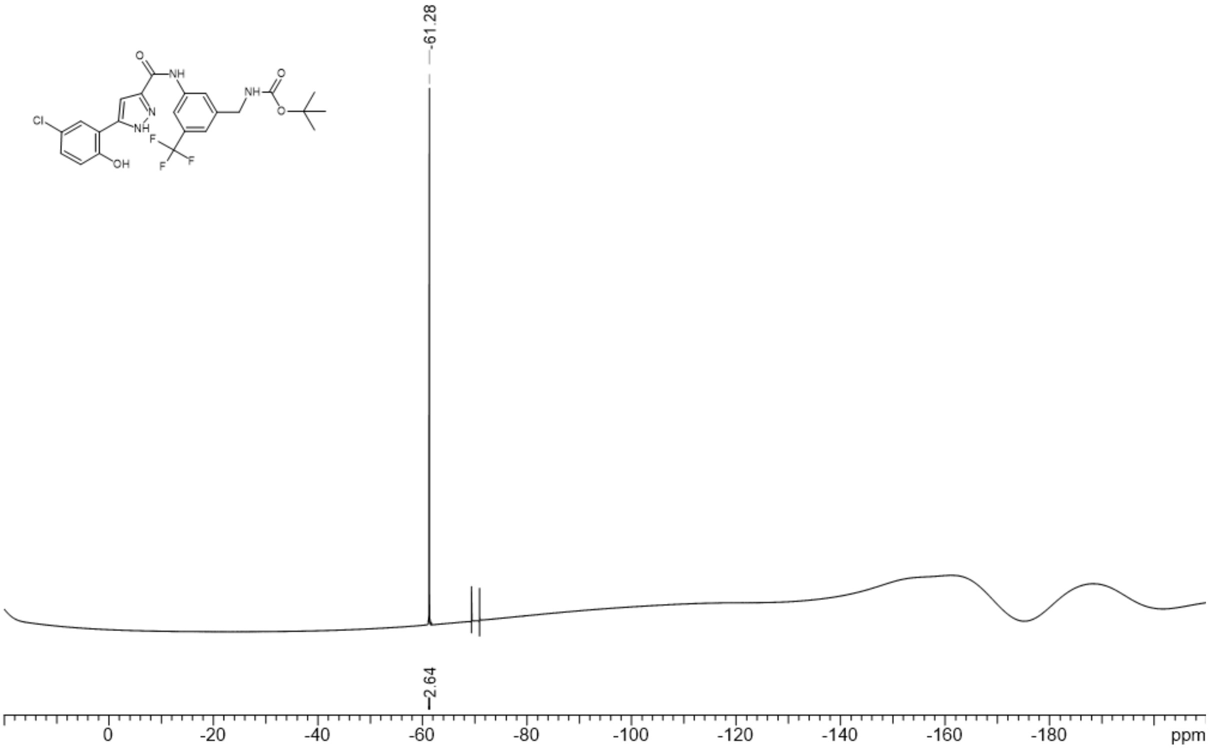

Figure S116. ^{19}F NMR spectrum of **9B.**

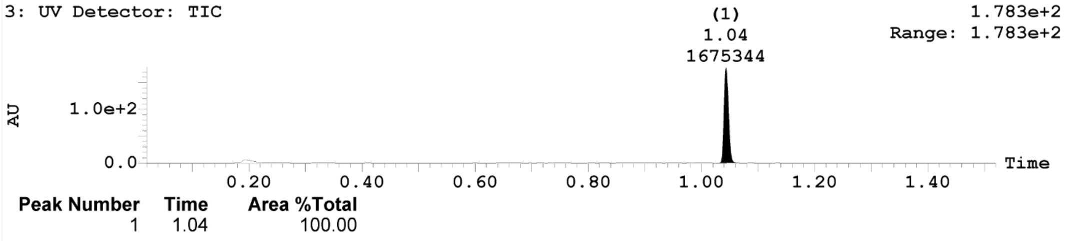

Figure S117. LCMS purity analysis of **9B**.

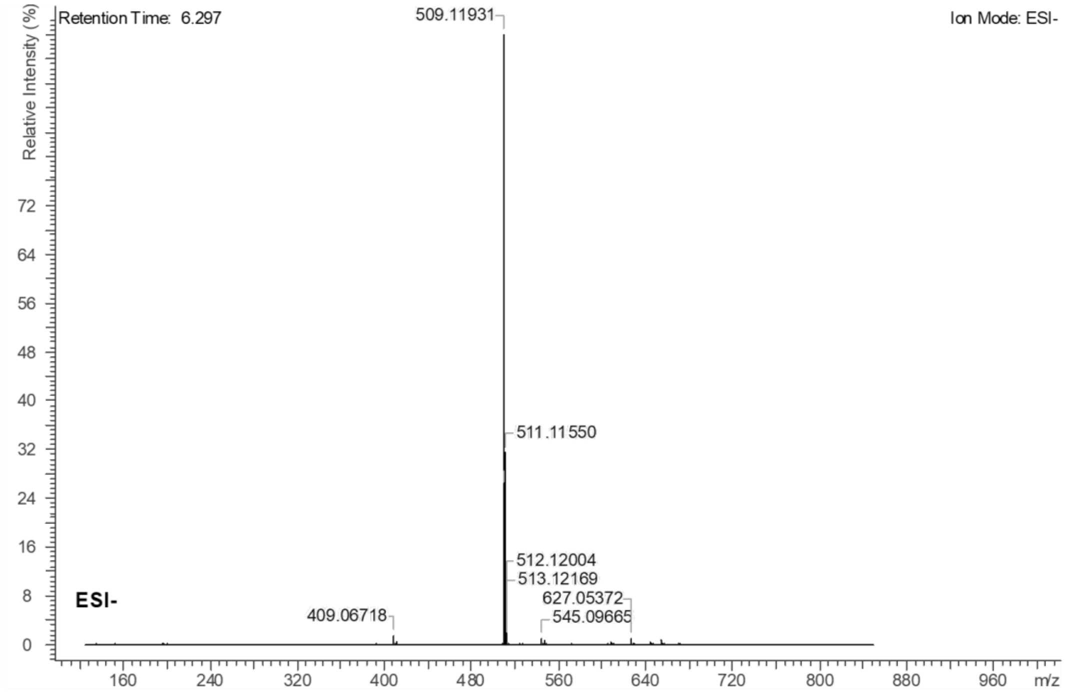

Figure S118. HRMS of **9B**.

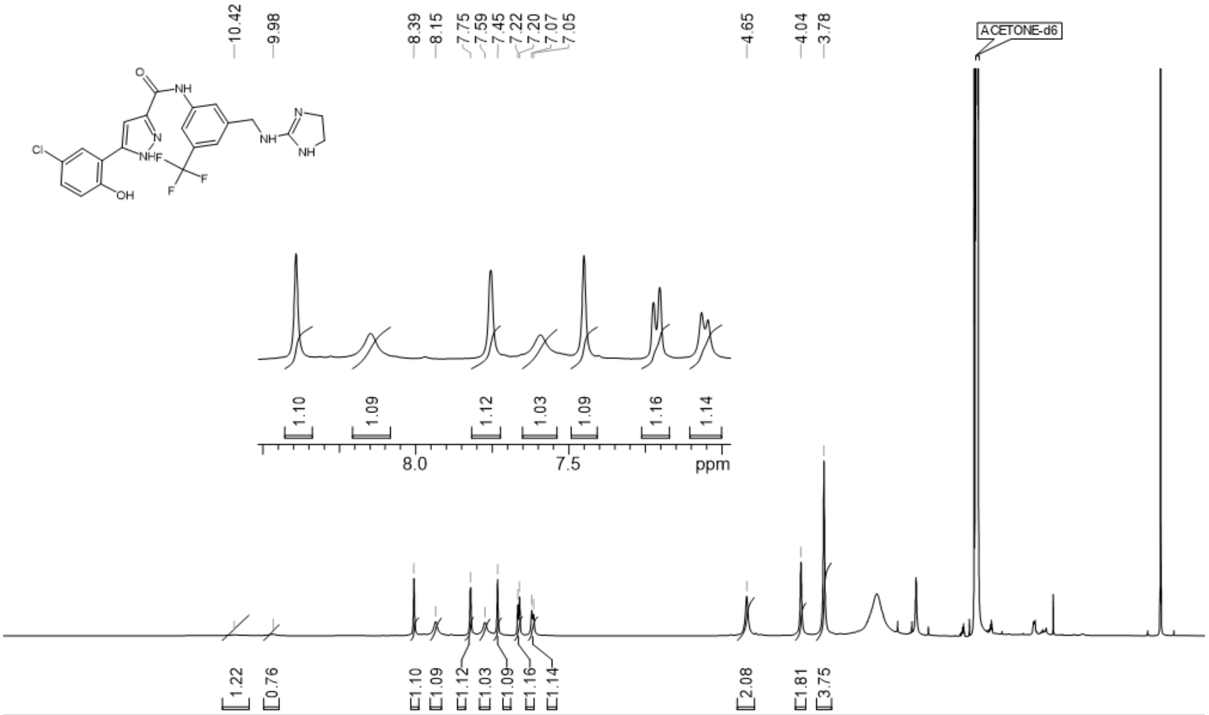

Figure S119. ^{1}H NMR spectrum of **9C**.

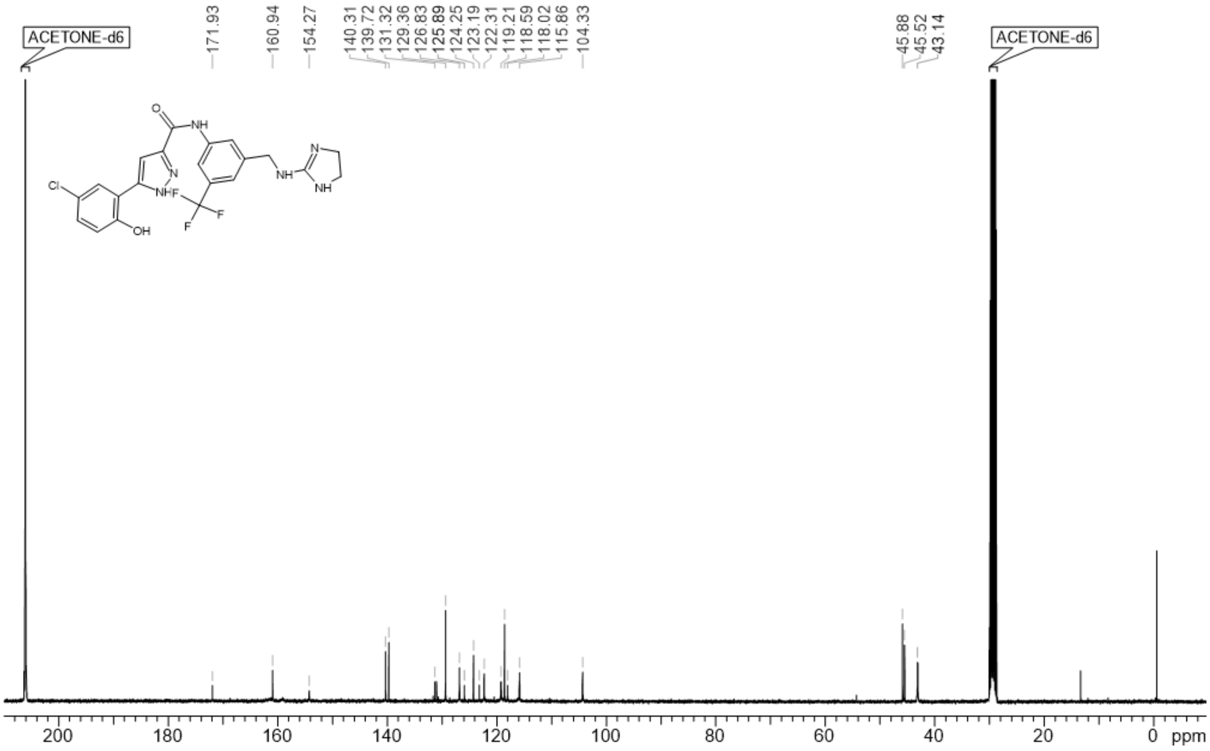

Figure S120. ^{13}C NMR spectrum of **9C**.

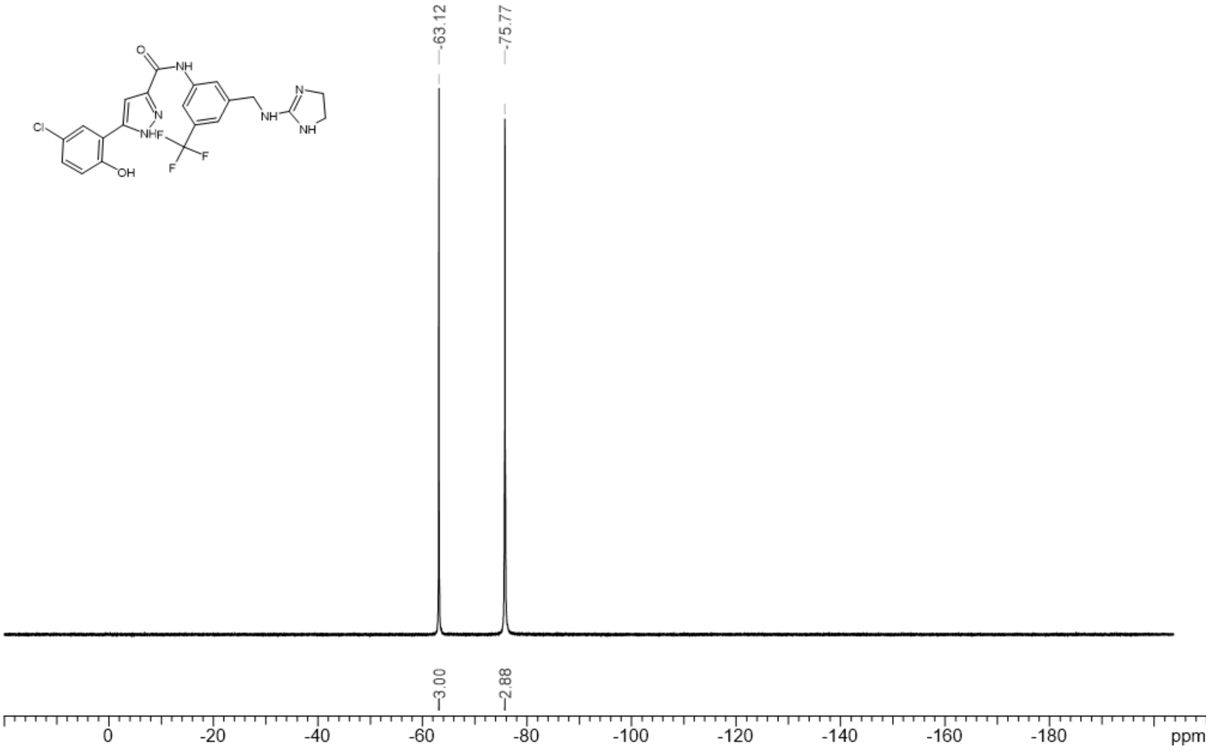

Figure S121. ^{19}F NMR spectrum of **9C.**

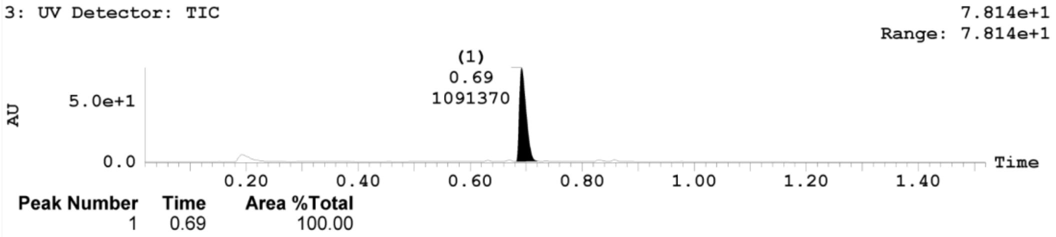

Peak Number	Time	Area %Total
1	0.69	100.00

Figure S122. LCMS purity analysis of **9C.**

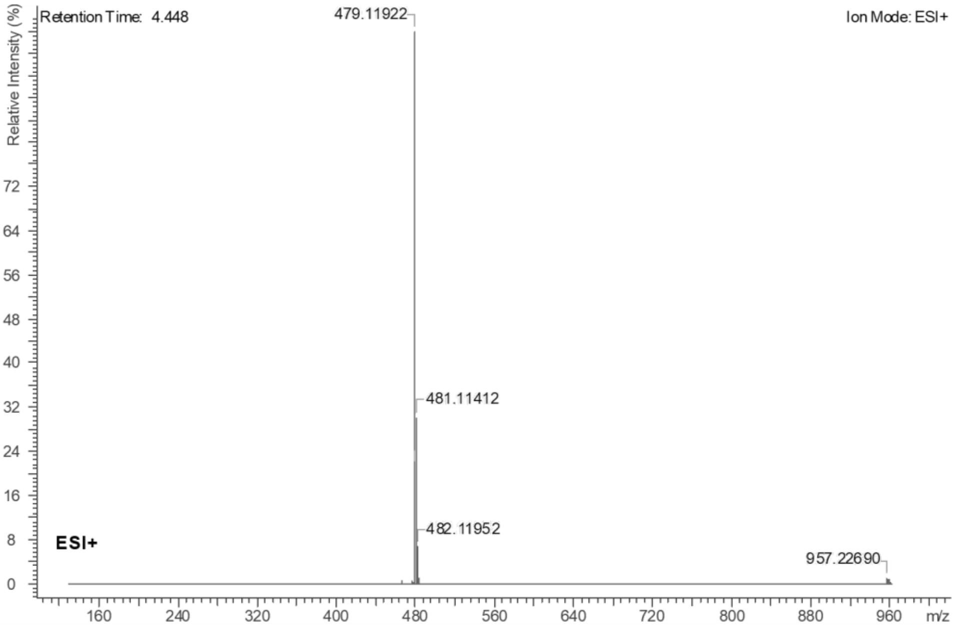

Figure S123. HRMS of **9C.**

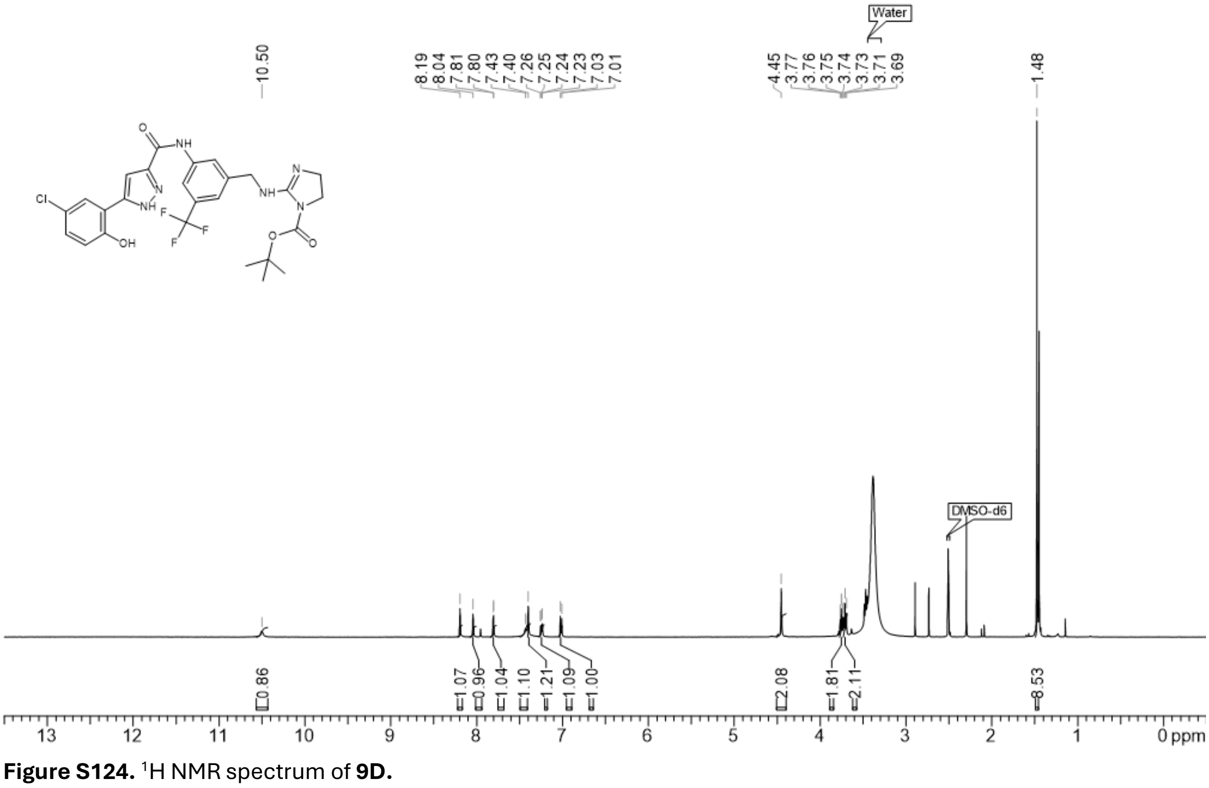

Figure S124. ^{1}H NMR spectrum of **9D.**

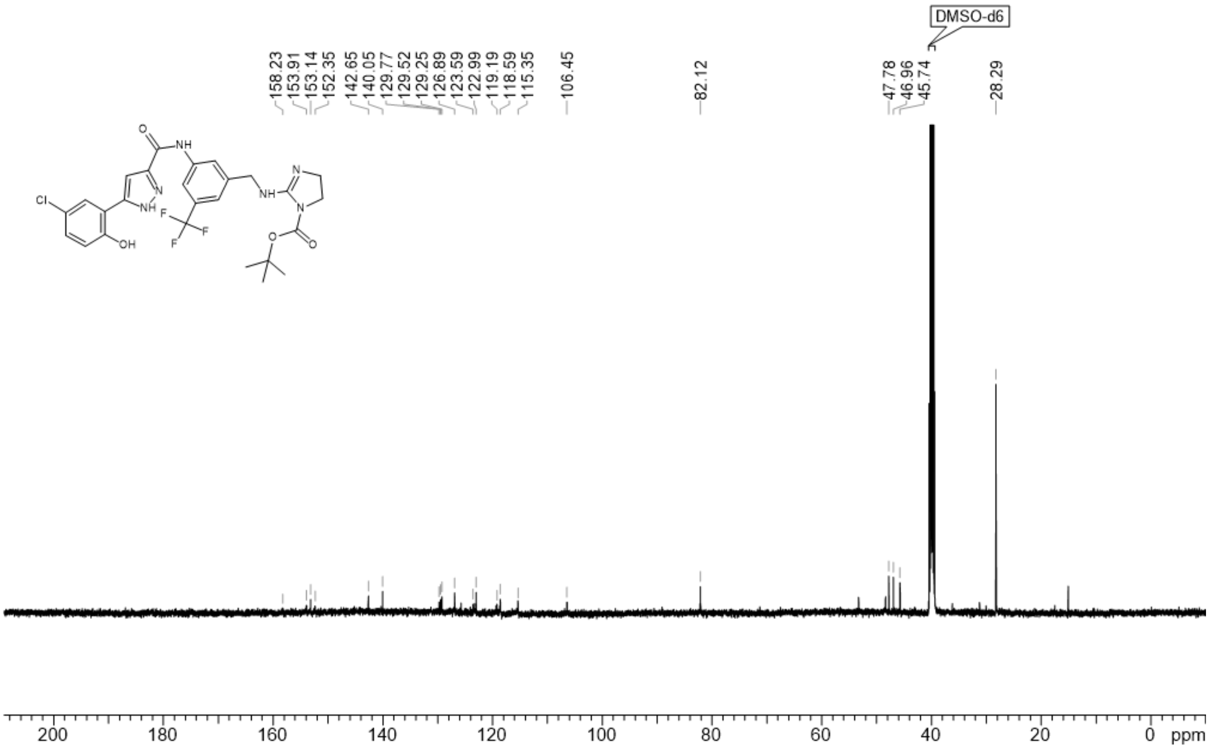

Figure S125. ^{13}C NMR spectrum of **9D.**

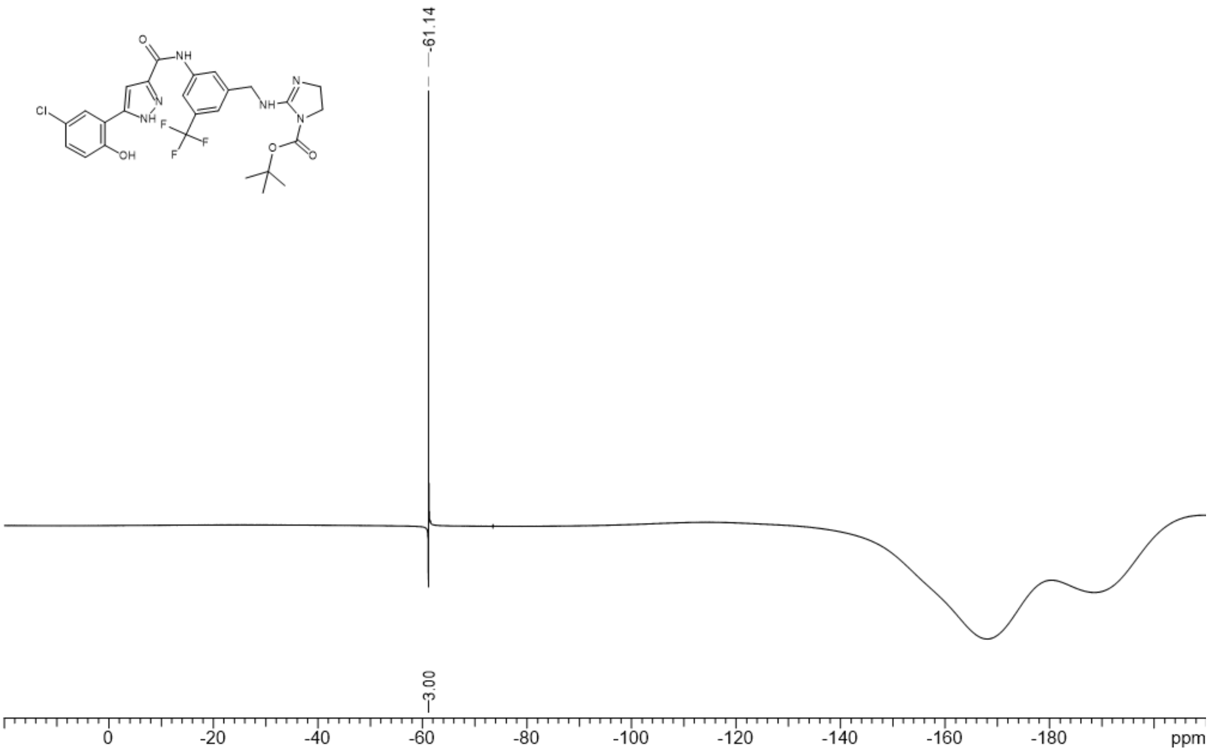

Figure S126. ^{19}F NMR spectrum of **9D.**

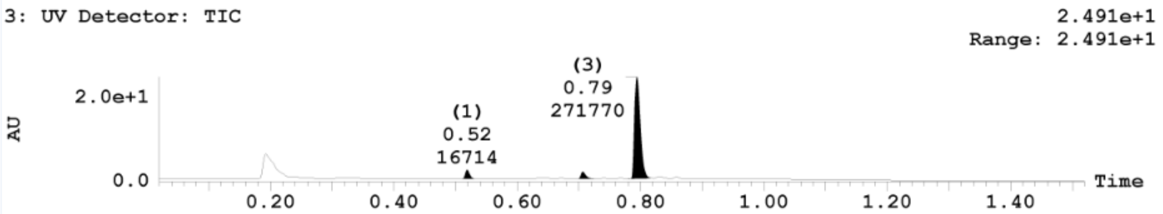

Peak Number	Time	Area %Total
1	0.52	5.52
2	0.71	4.74
3	0.79	89.74

Peak ID	Time	Mass Found	BPM
1	0.52		161.10

Peak ID	Time	Mass Found	BPM
1	0.52		133.98

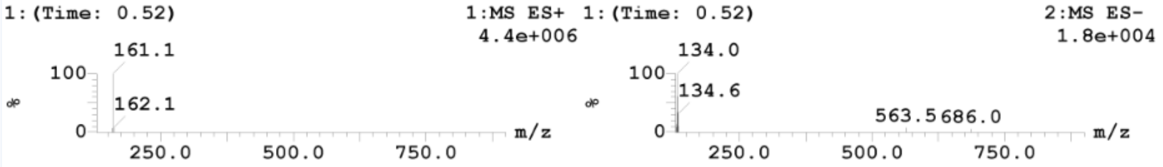

Peak ID	Time	Mass Found	BPM
2	0.71		479.19

Peak ID	Time	Mass Found	BPM
2	0.71		477.03

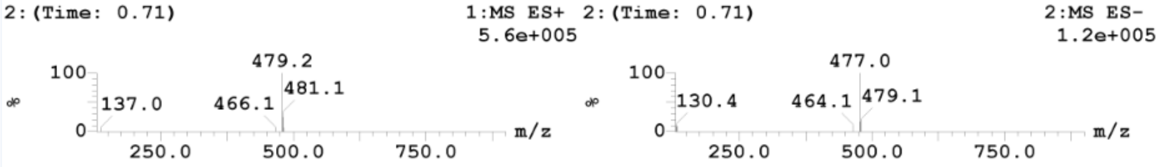

Peak ID	Time	Mass Found	BPM
3	0.79	578.17	579.20

Peak ID	Time	Mass Found	BPM
3	0.79	578.17	577.08

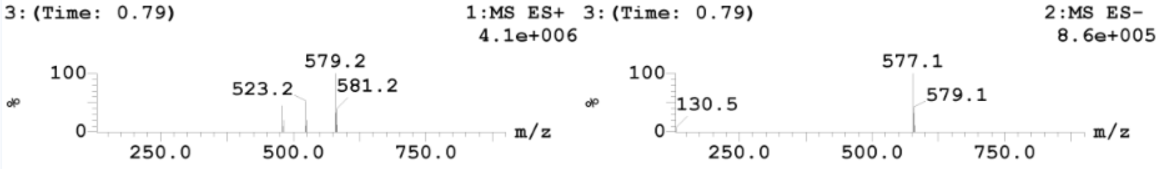

Figure S127. LCMS purity analysis of **9D**, 5% impurity of **9C.**

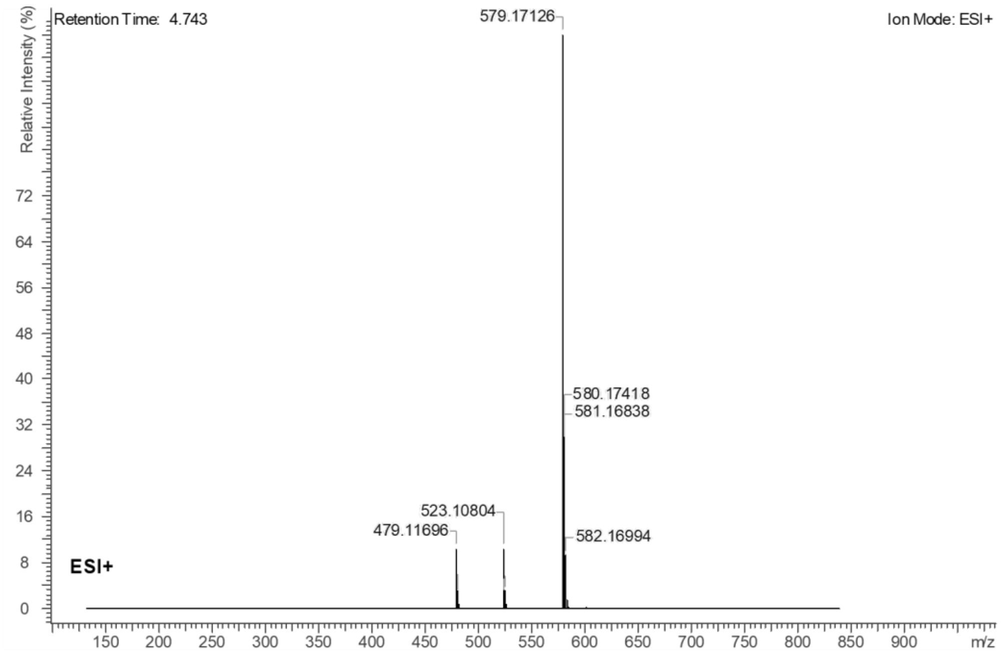

Figure S128. HRMS of **9D.**

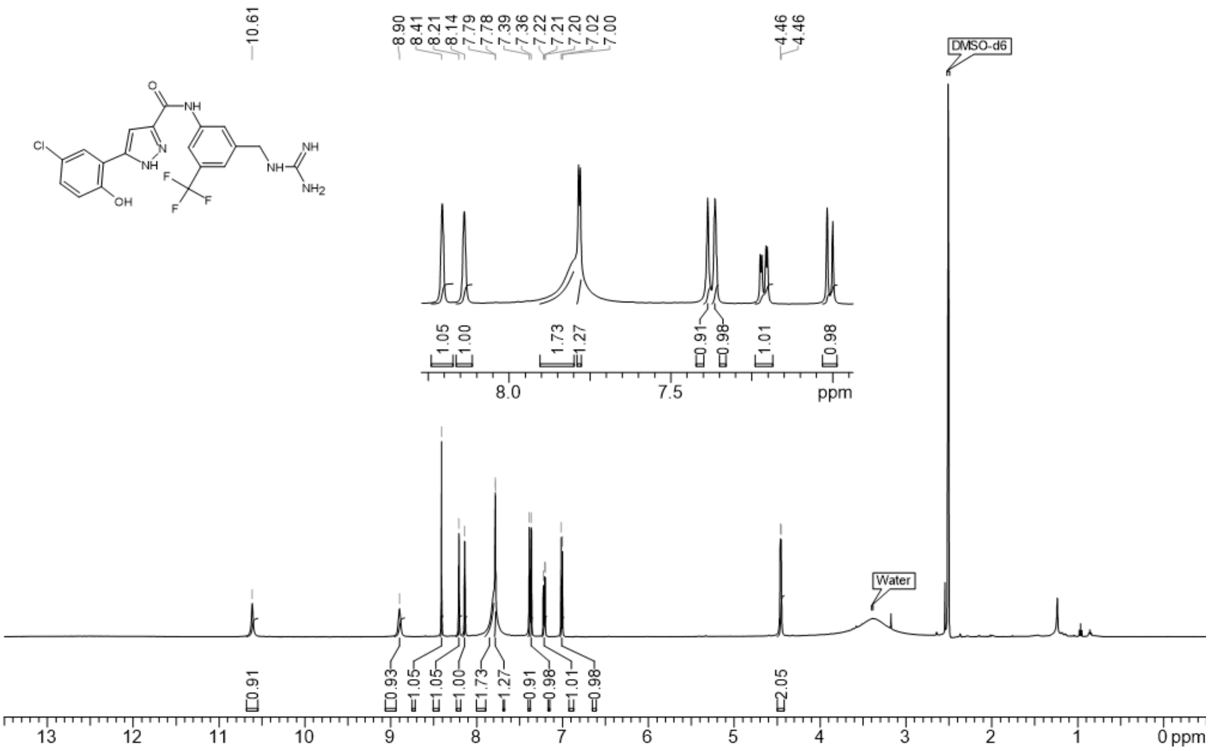

Figure S129. ^{1}H NMR spectrum of **9G.**

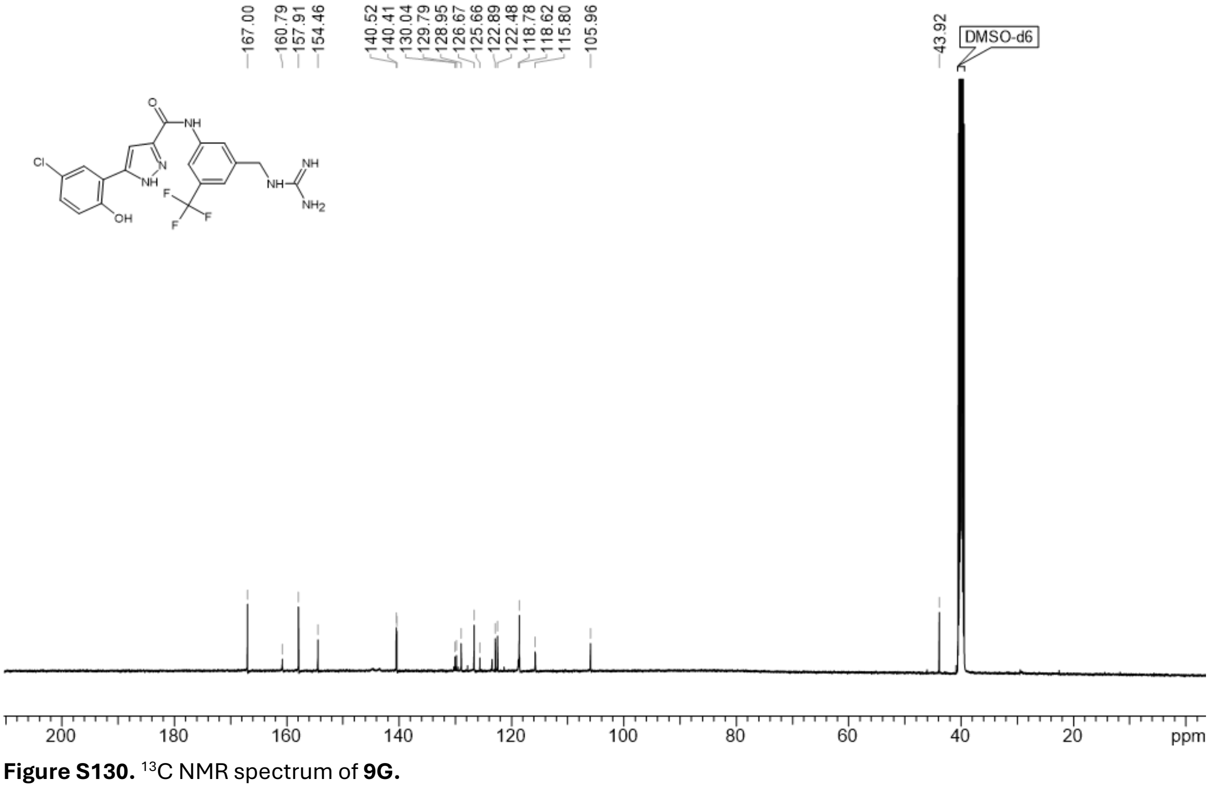

Figure S130. ^{13}C NMR spectrum of **9G.**

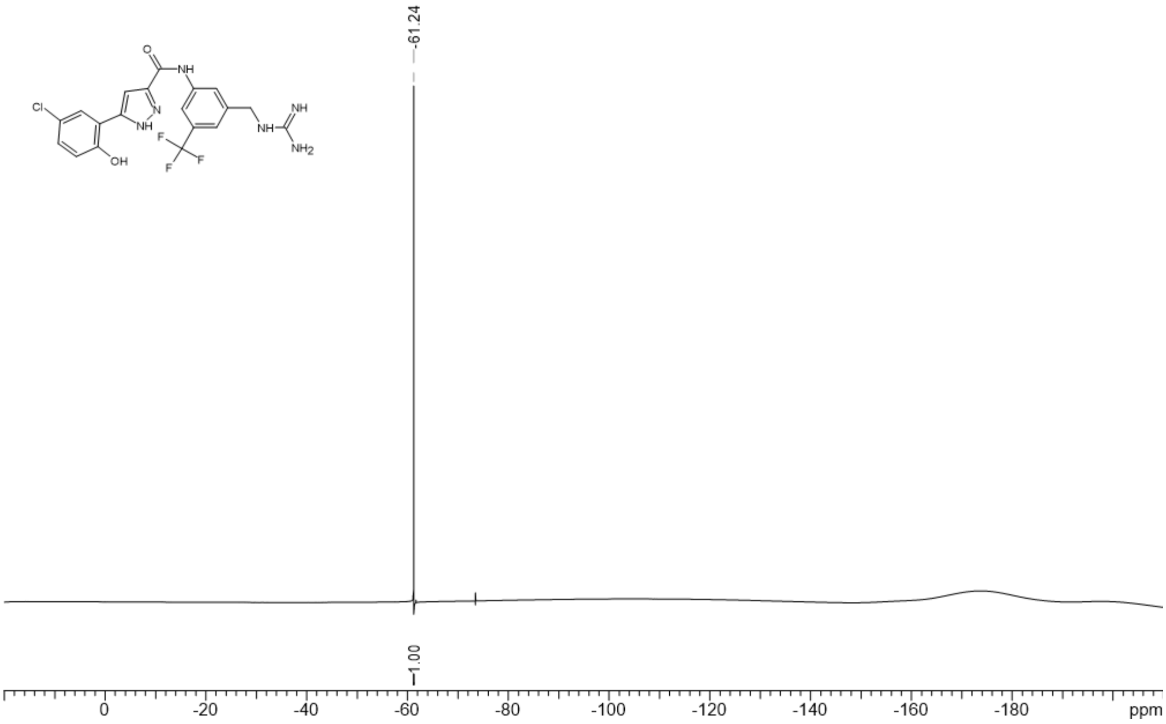

Figure S131. ^{19}F NMR spectrum of **9G.**

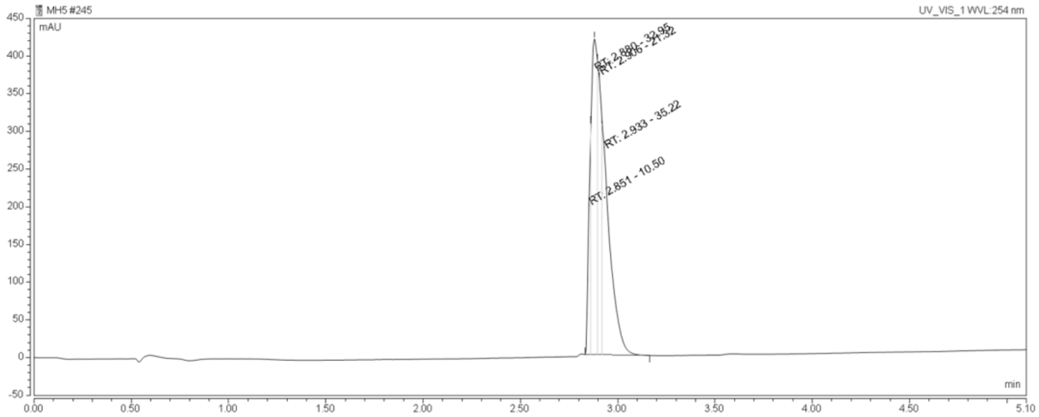

Figure S132. LCMS purity analysis of **9G.**

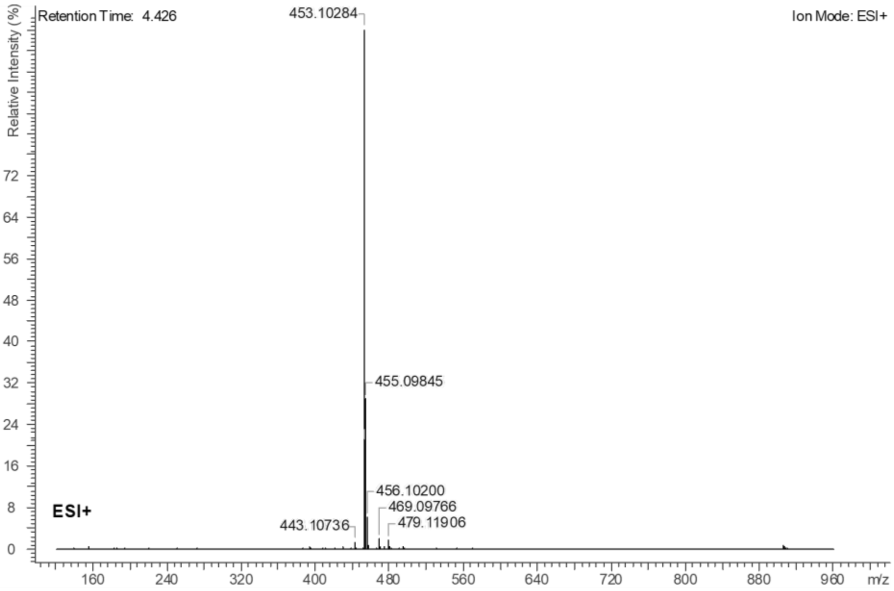

Figure S133. HRMS of **9G.**

Compounds **10A–G**

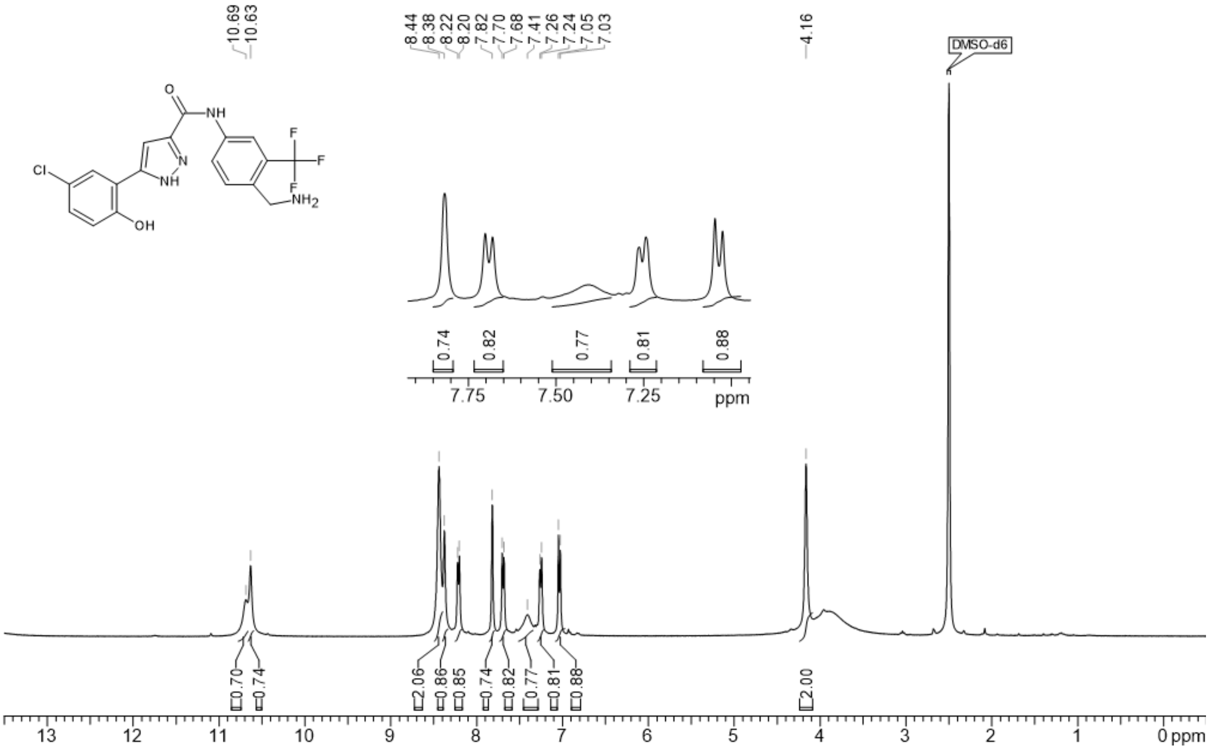

Figure S134. ^{1}H NMR spectrum of **10A.**

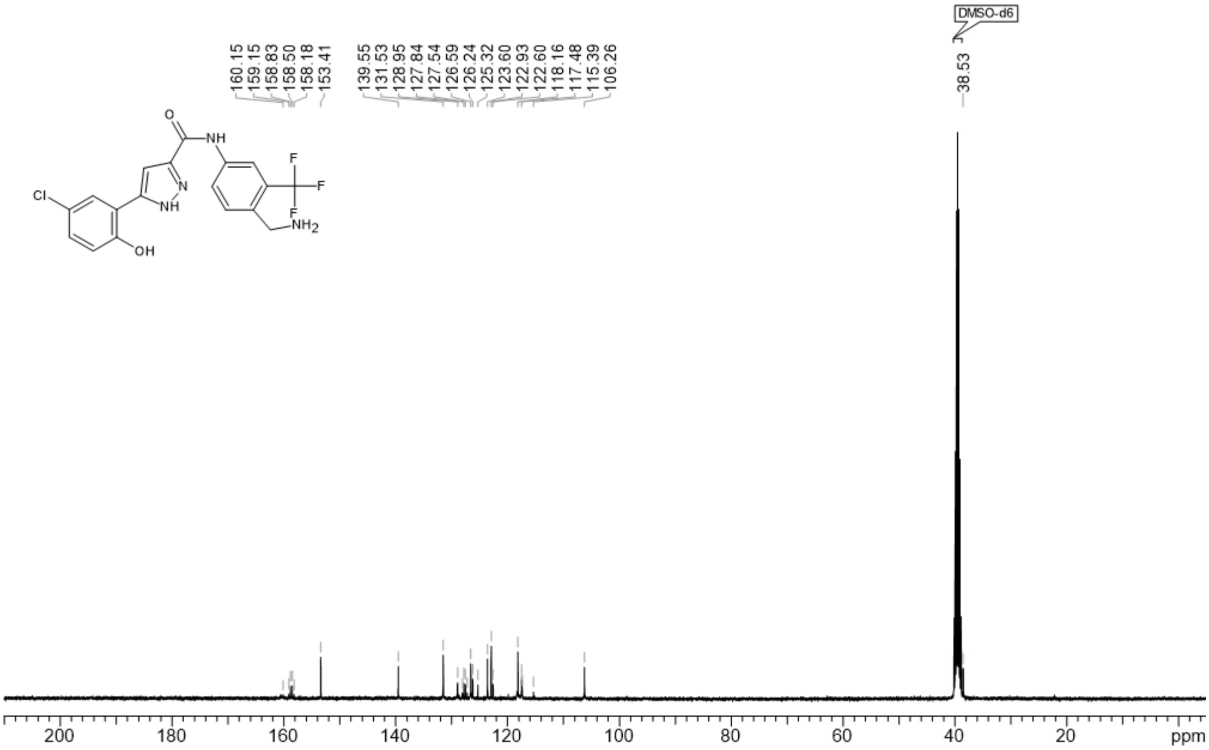

Figure S135. ^{13}C NMR spectrum of **10A.**

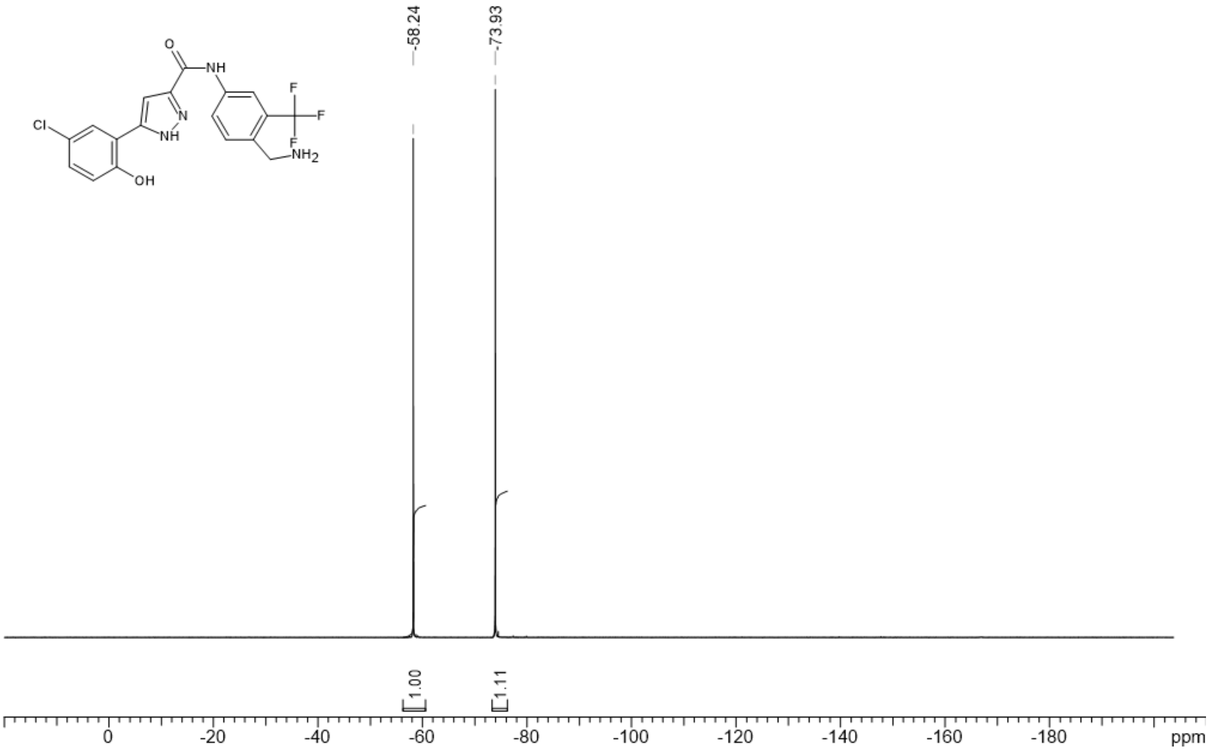

Figure S136. ^{19}F NMR spectrum of **10A.**

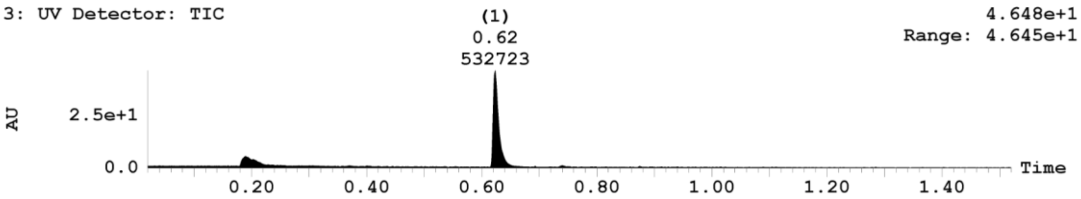

Peak Number	Time	Area %Total
1	0.62	100.00

Figure S137. LCMS purity analysis of **10A.**

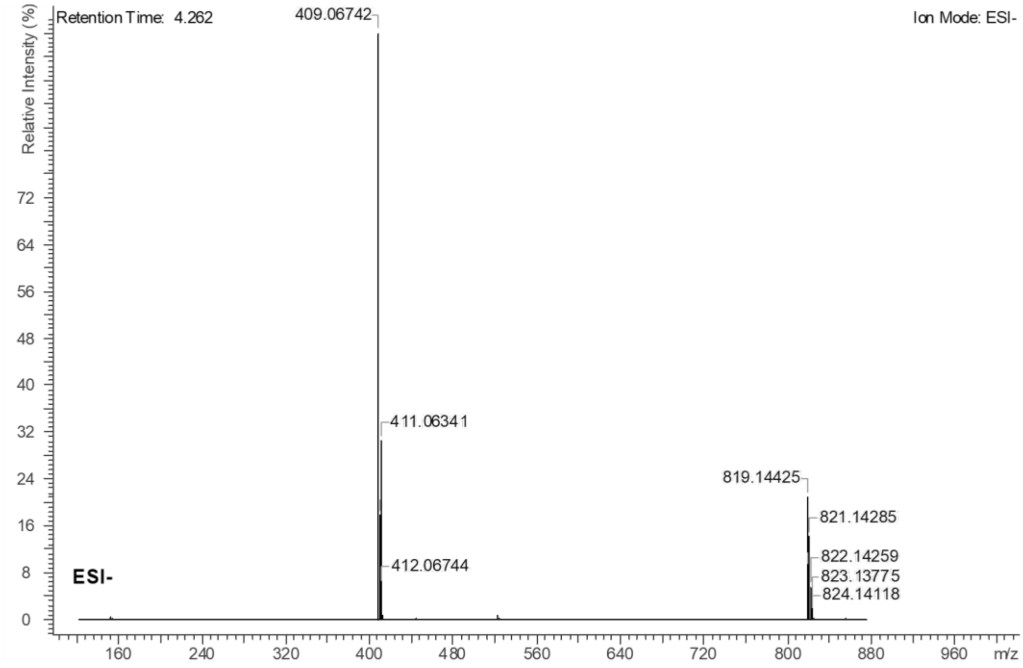

Figure S138. HRMS of **10A.**

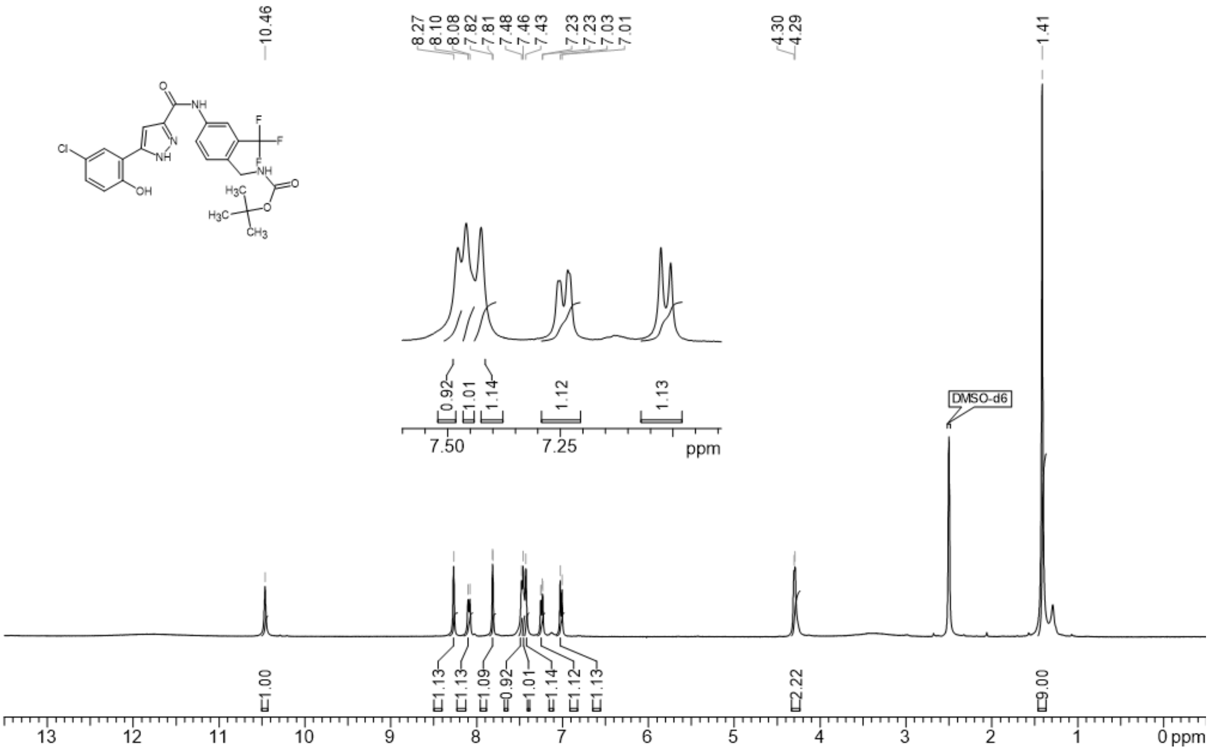

Figure S139. ^{1}H NMR spectrum of **10B.**

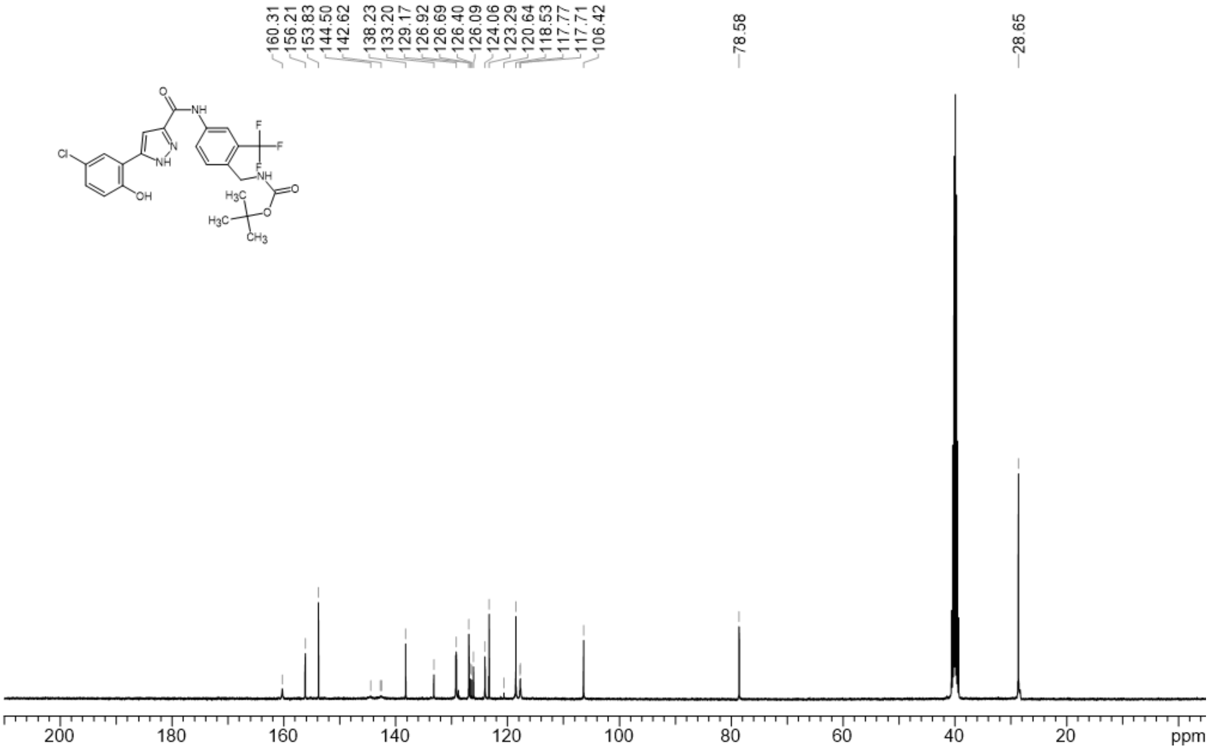

Figure S140. ^{13}C NMR spectrum of **10B.**

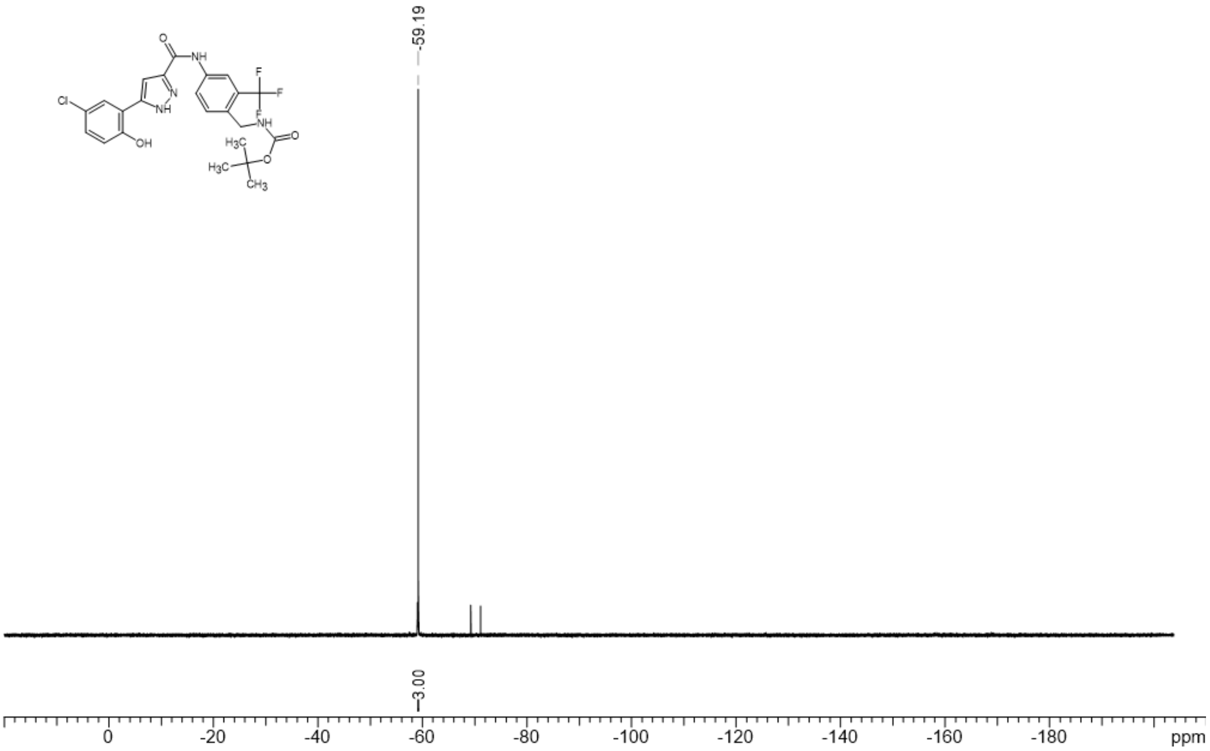

Figure S141. ^{19}F NMR spectrum of **10B.**

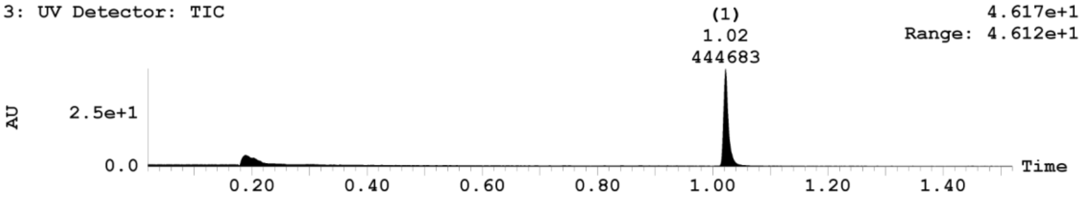

Peak Number	Time	Area %Total
1	1.02	100.00

Figure S142. LCMS purity analysis of **10B.**

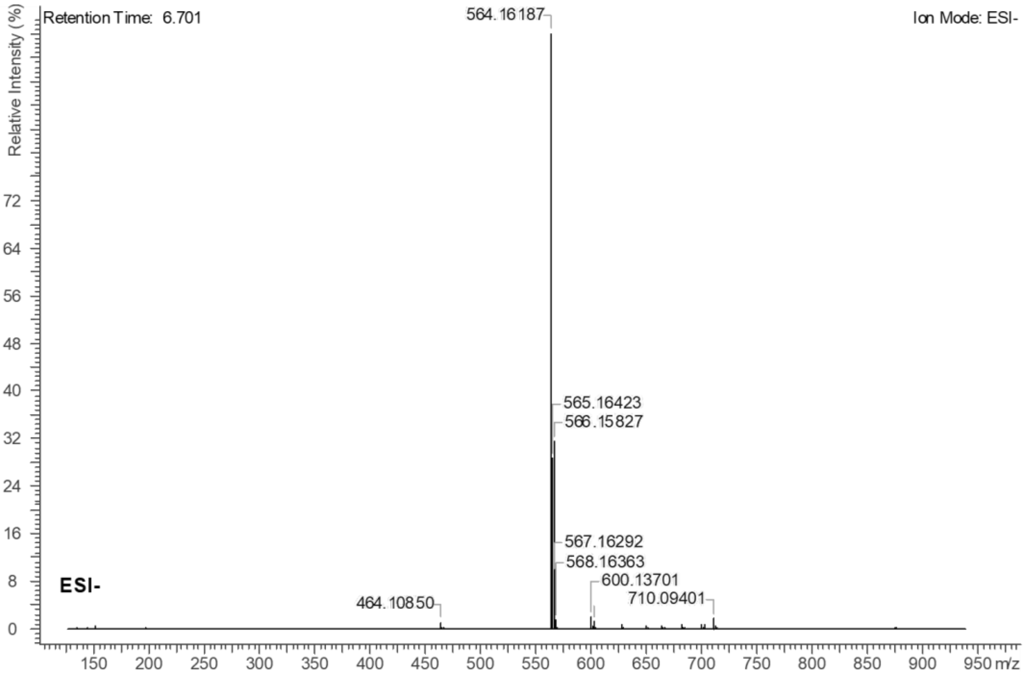

Figure S143. HRMS of **10B.**

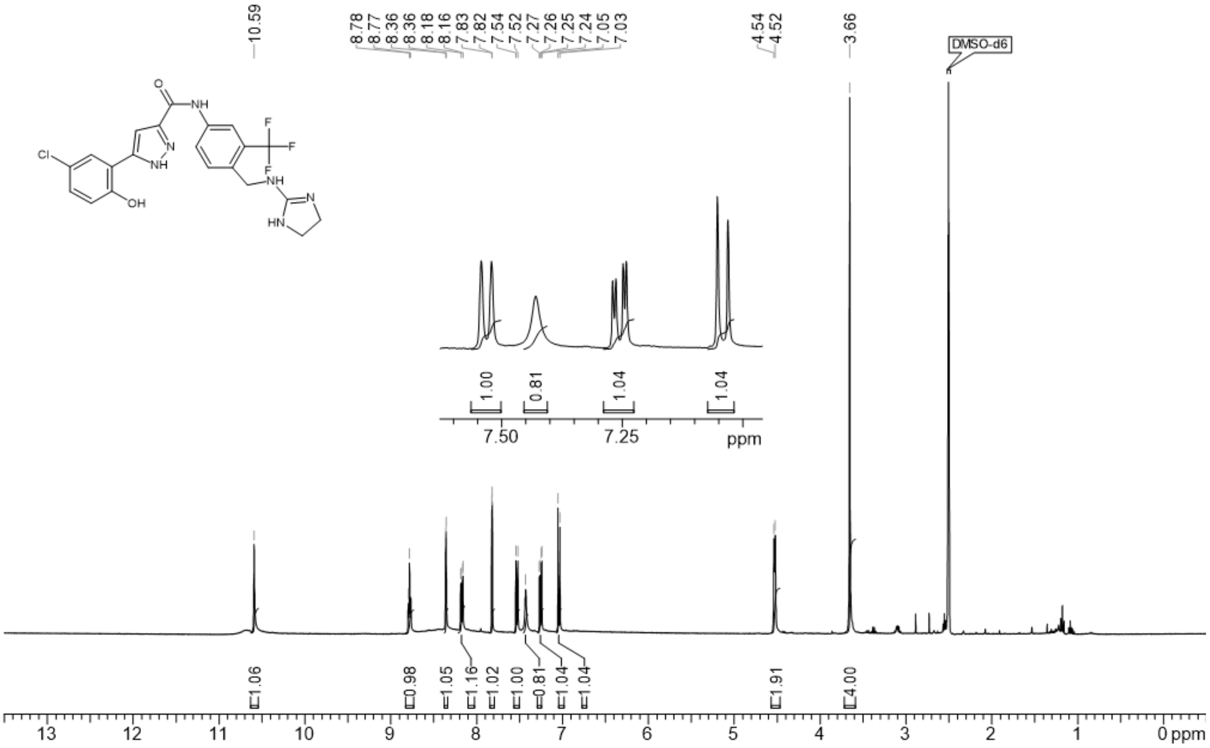

Figure S144. ^{1}H NMR spectrum of **10C.**

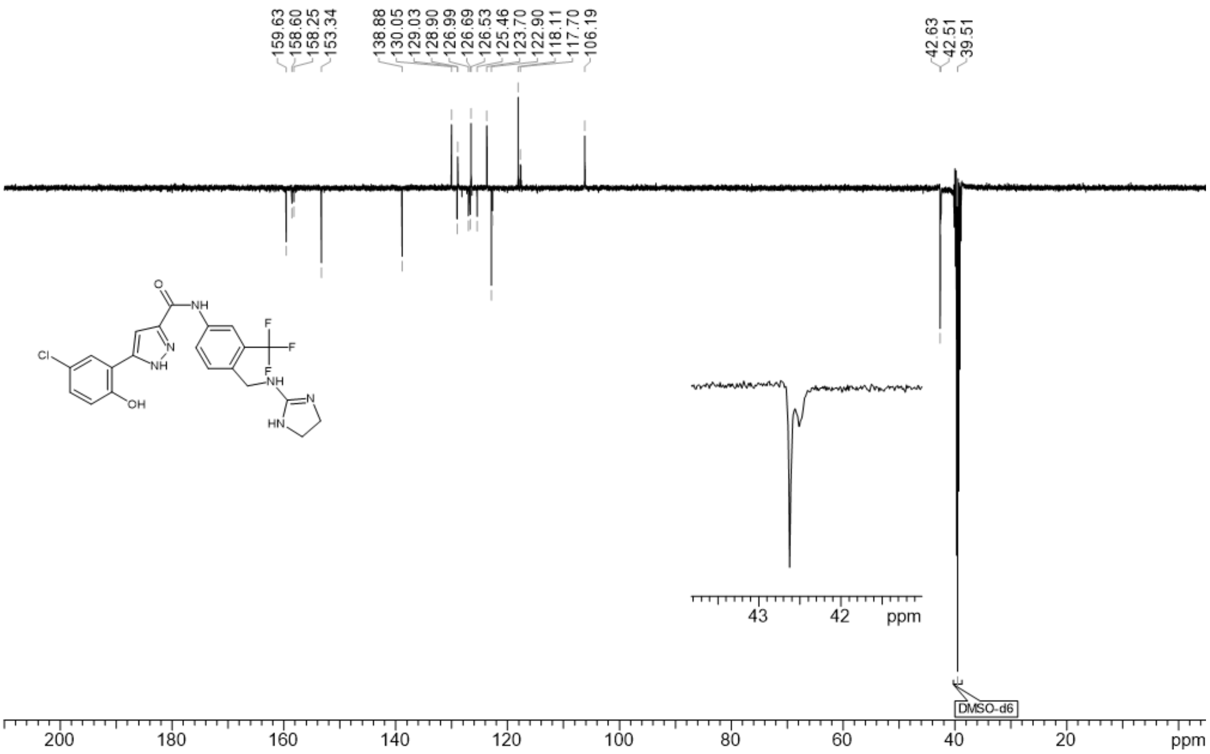

Figure S145. ^{13}C APT NMR spectrum of **10C.**

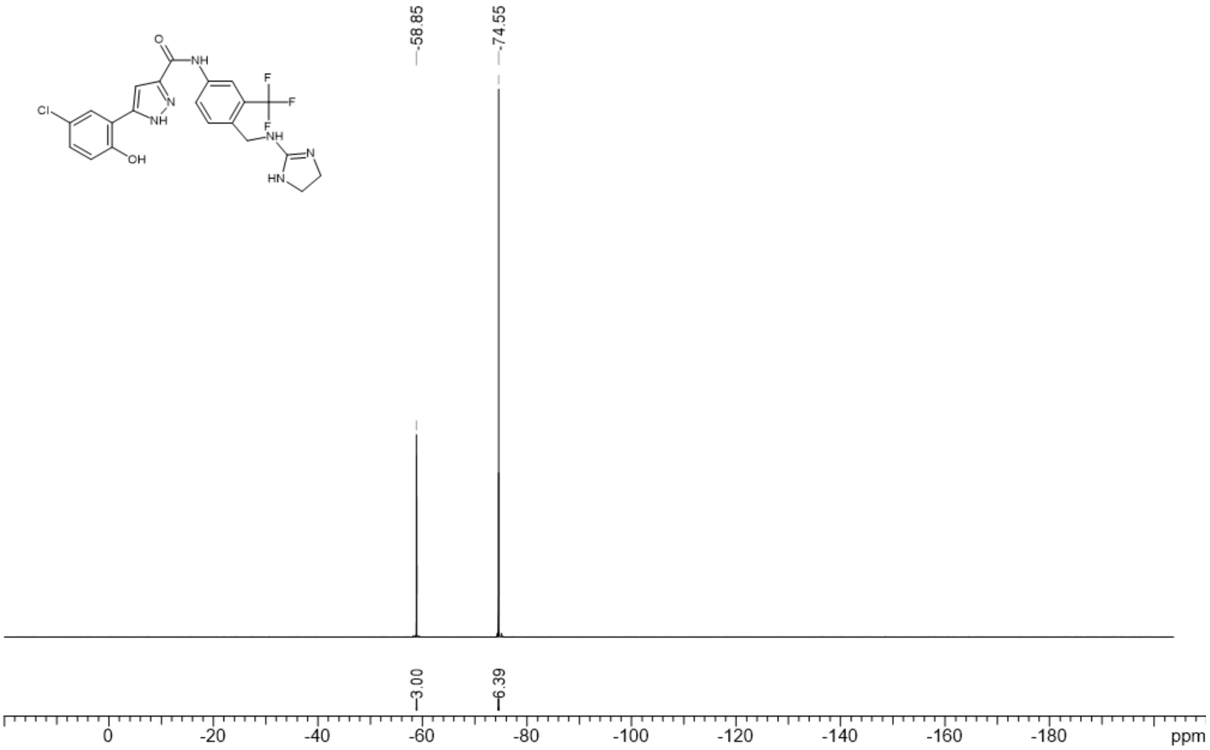

Figure S146. ^{19}F NMR spectrum of **10C.**

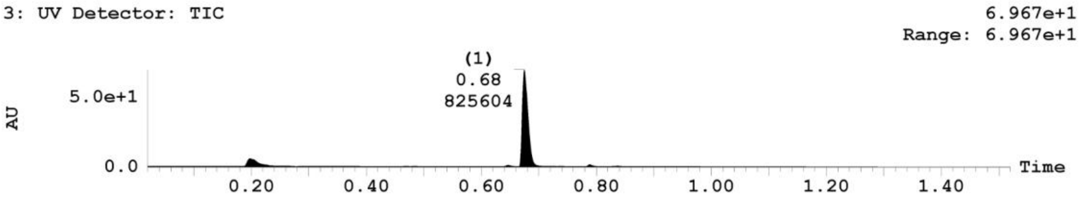

Peak Number	Time	Area %Total
1	0.67	100.00

Figure S147. LCMS purity analysis of **10C.**

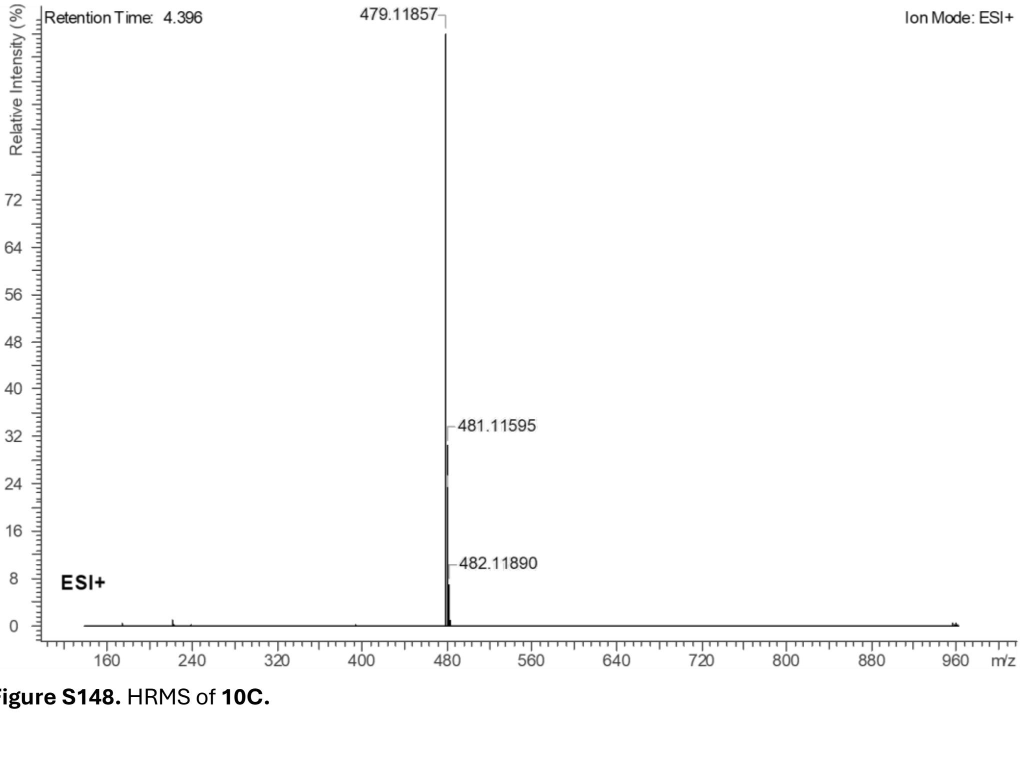

Figure S148. HRMS of **10C.**

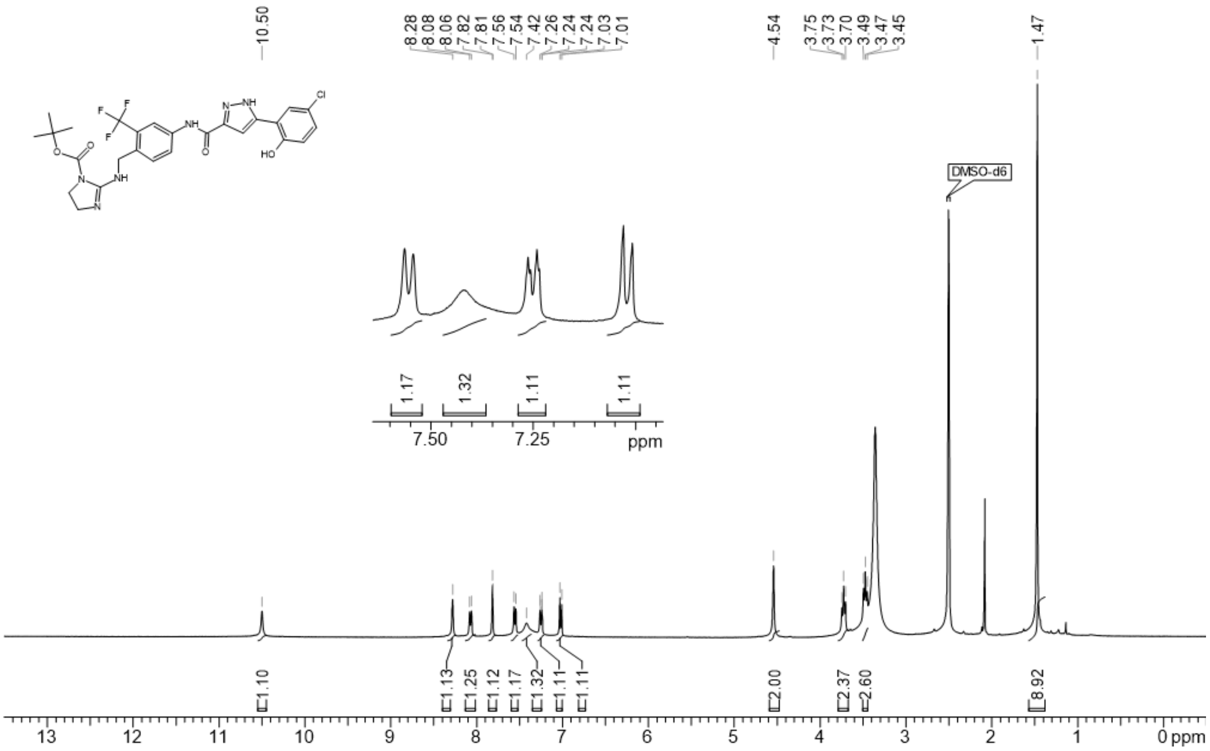

Figure S149. ^{1}H NMR spectrum of **10D.**

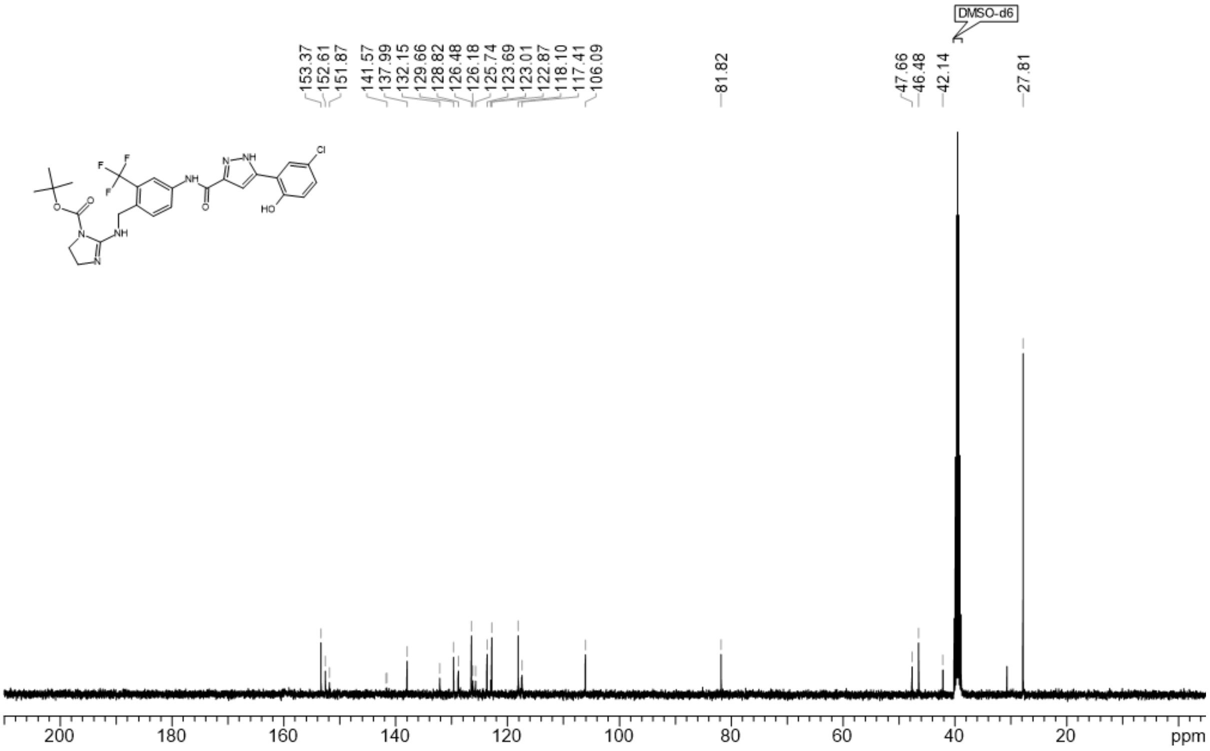

Figure S150. ^{13}C NMR spectrum of **10D.**

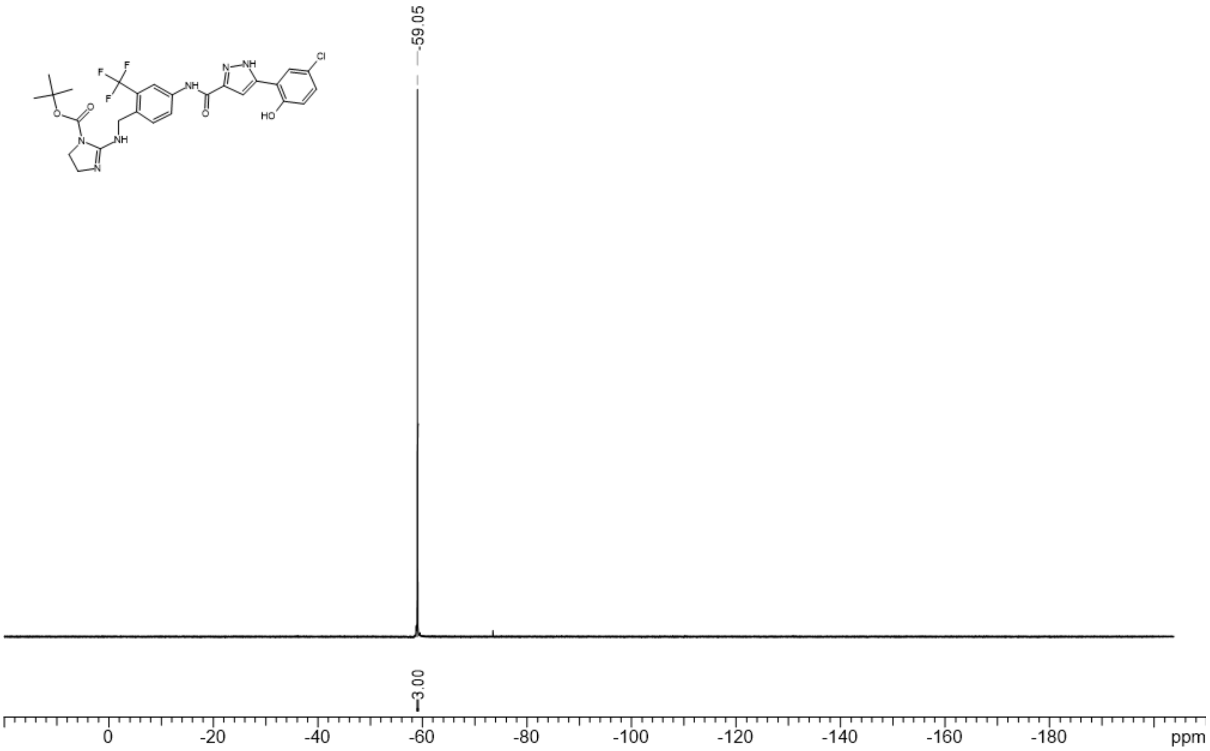

Figure S151. ^{19}F NMR spectrum of **10D.**

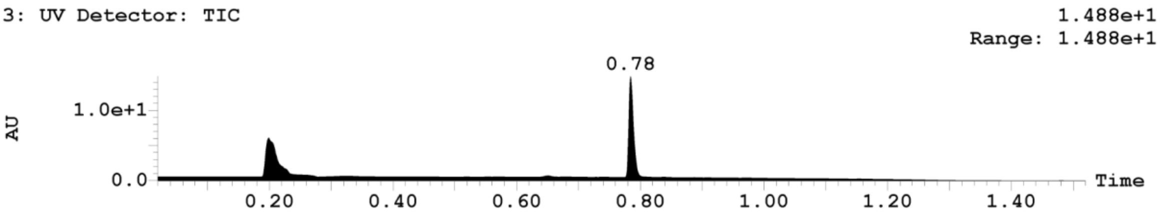

Peak Number	Time	Area %Total
1	0.78	100.00

Figure S152. LCMS purity analysis of **10D.**

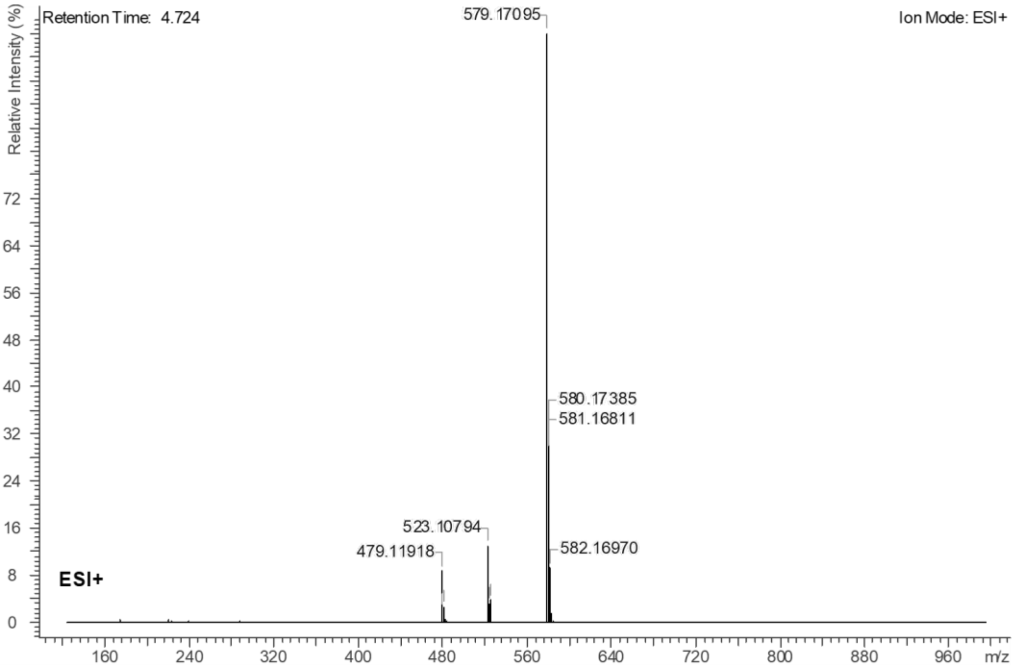

Figure S153. HRMS of **10D.**

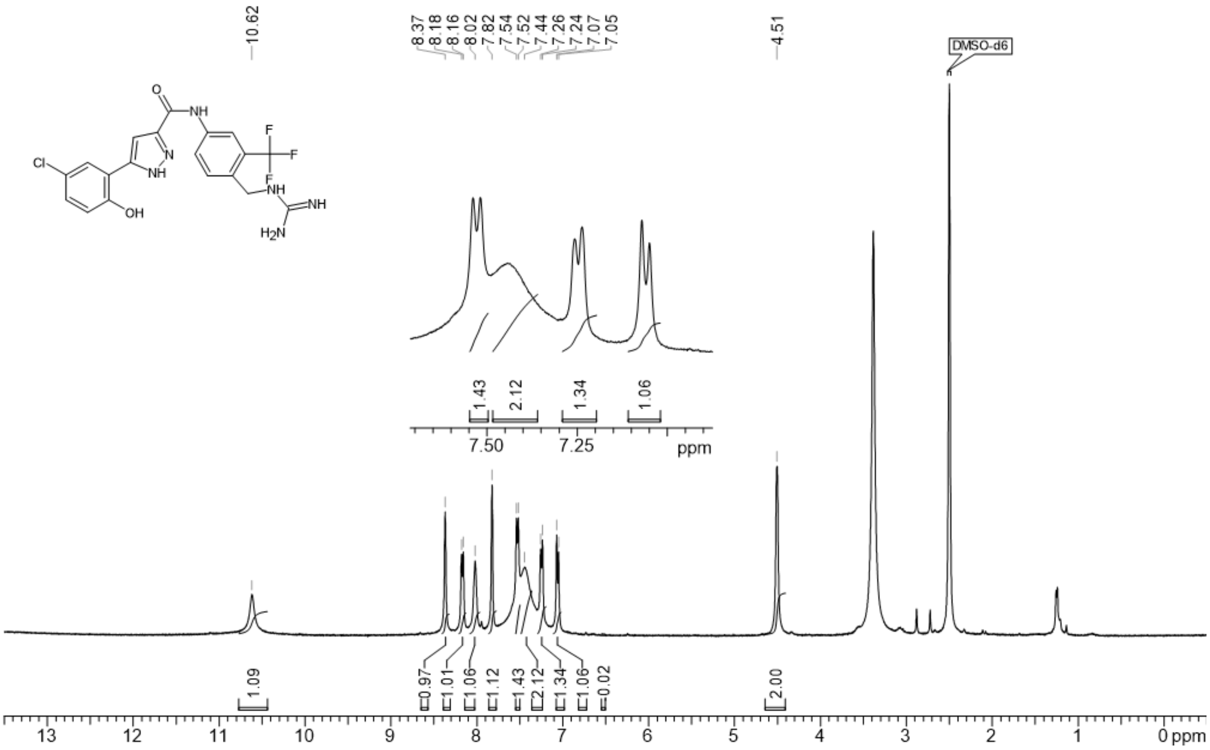

Figure S154. ^{1}H NMR spectrum of **10G.**

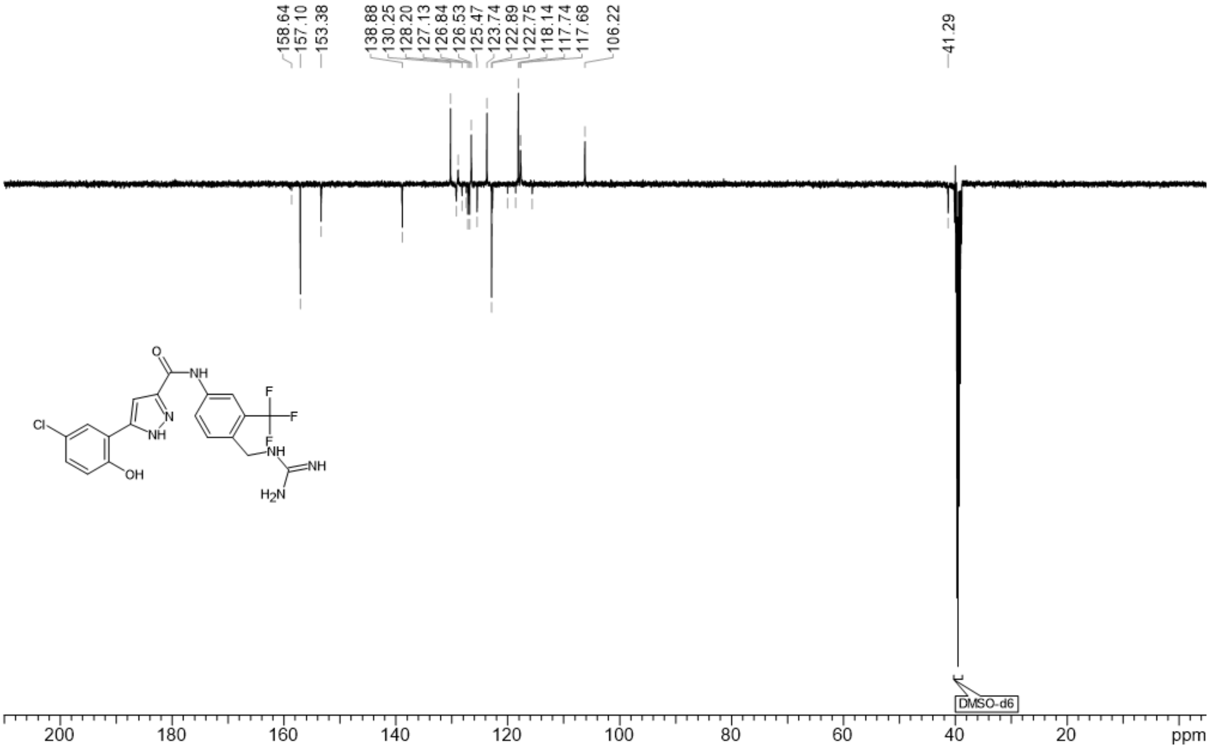

Figure S155. ^{13}C APT NMR spectrum of **10G.**

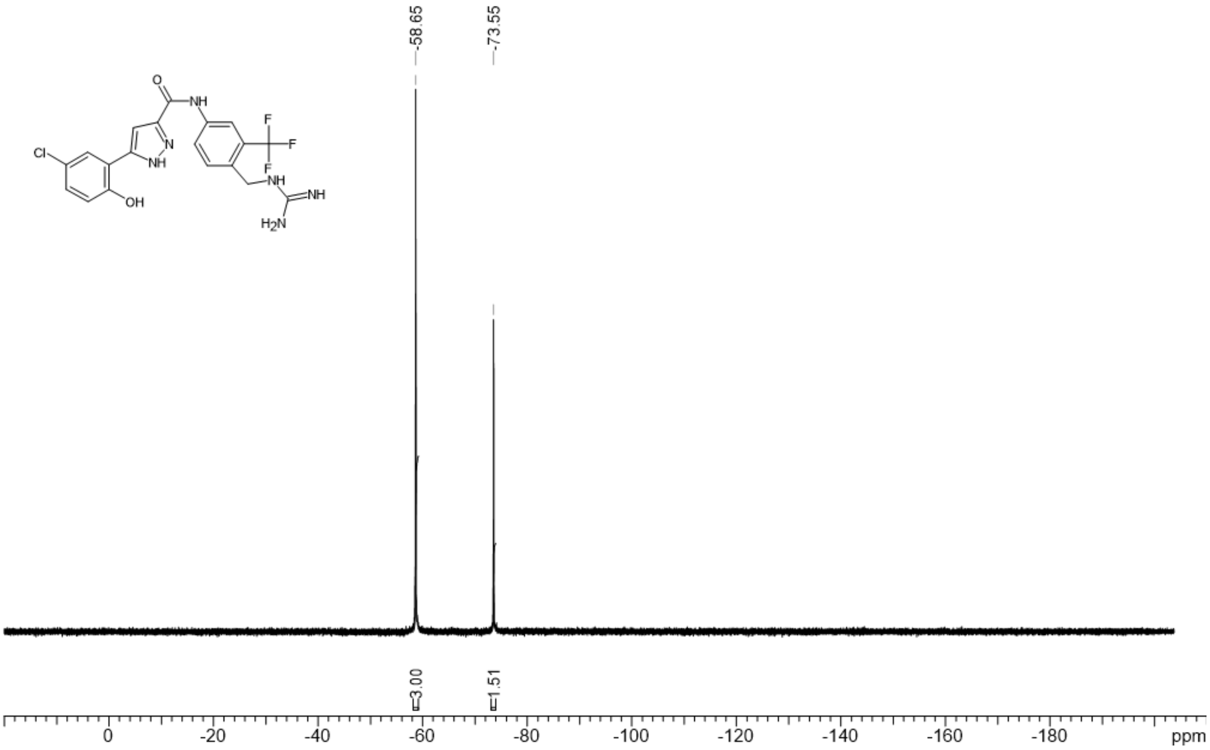

Figure S156. ^{19}F NMR spectrum of **10D.**

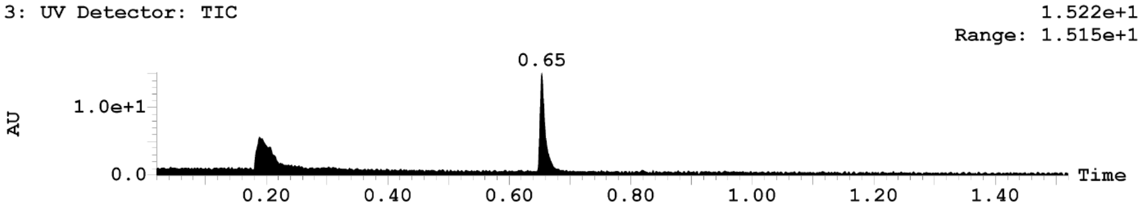

Peak Number	Time	Area %Total
1	0.65	100.00

Figure S157. LCMS purity analysis of **10G.**

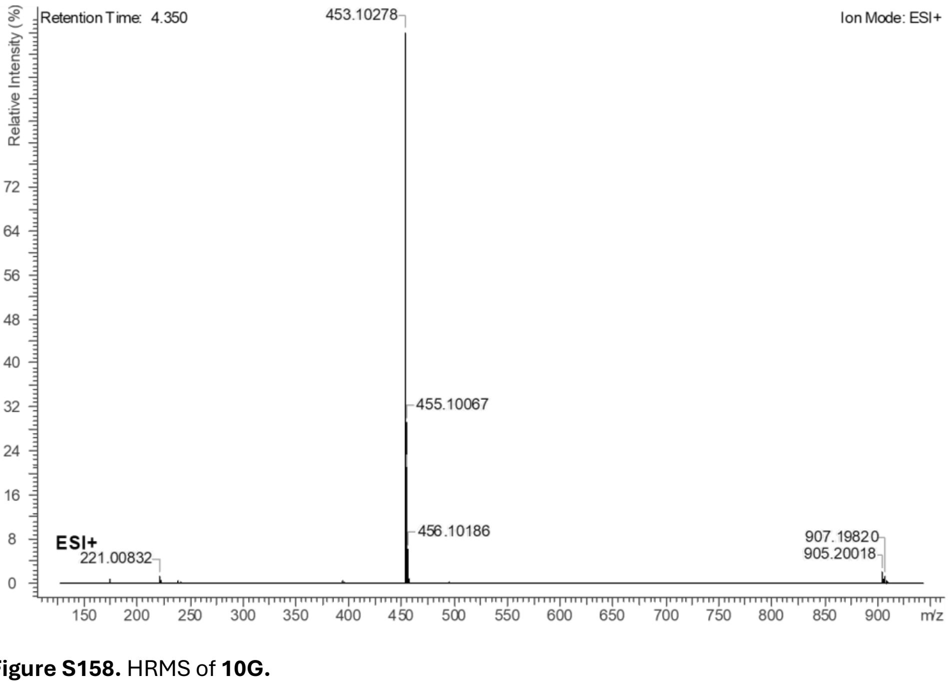

Figure S158. HRMS of **10G.**

Compounds **11A–G**

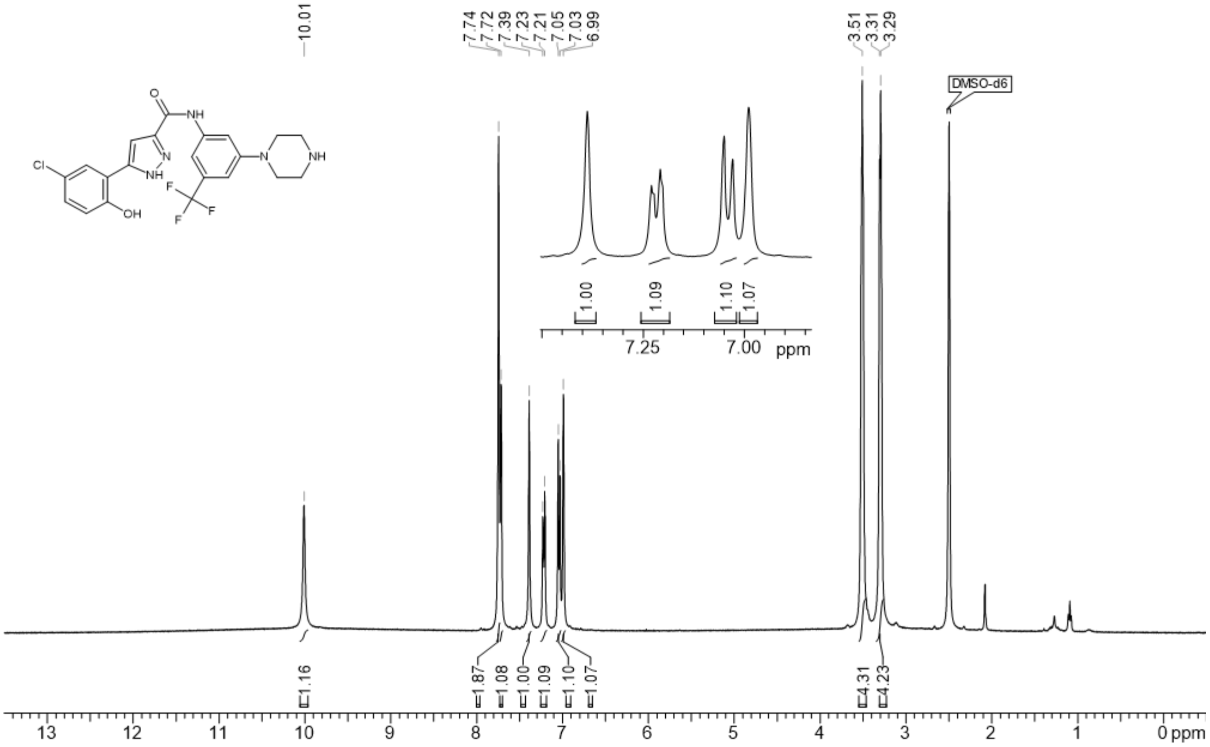

Figure S159. ^{1}H NMR spectrum at 120 °C of **11A.**

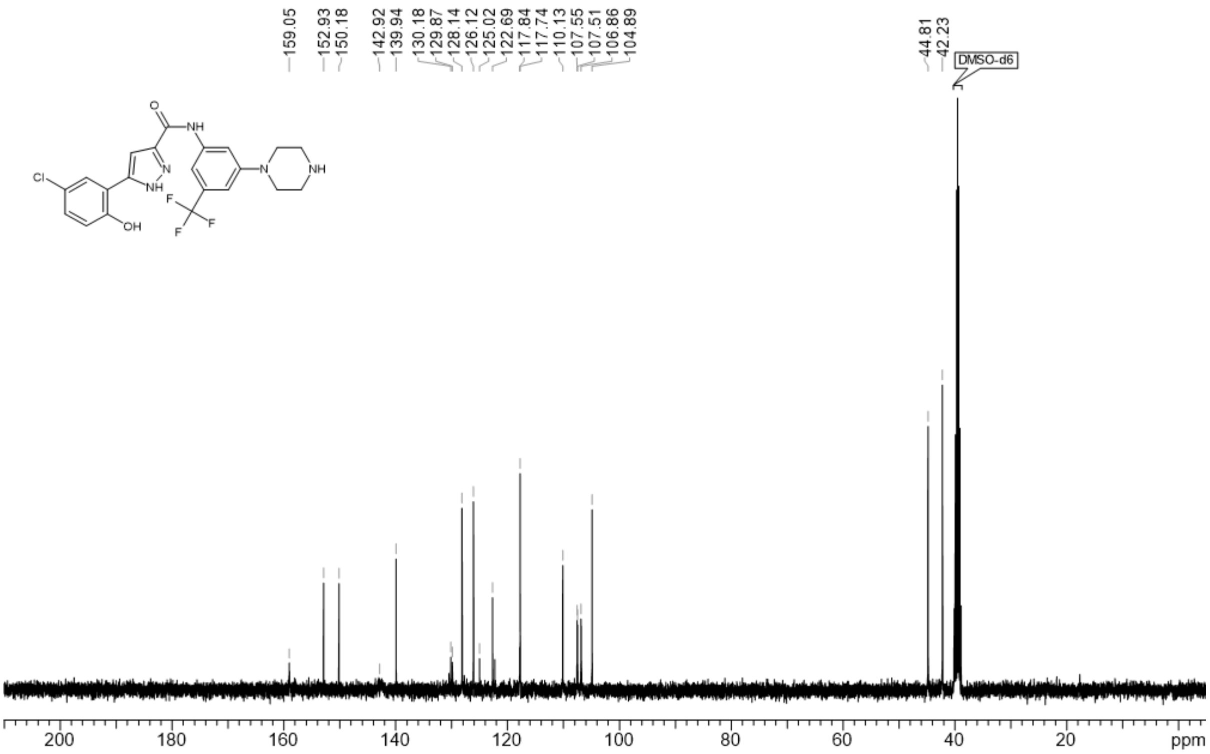

Figure S160. ^{13}C NMR spectrum at 120 °C of **11A.**

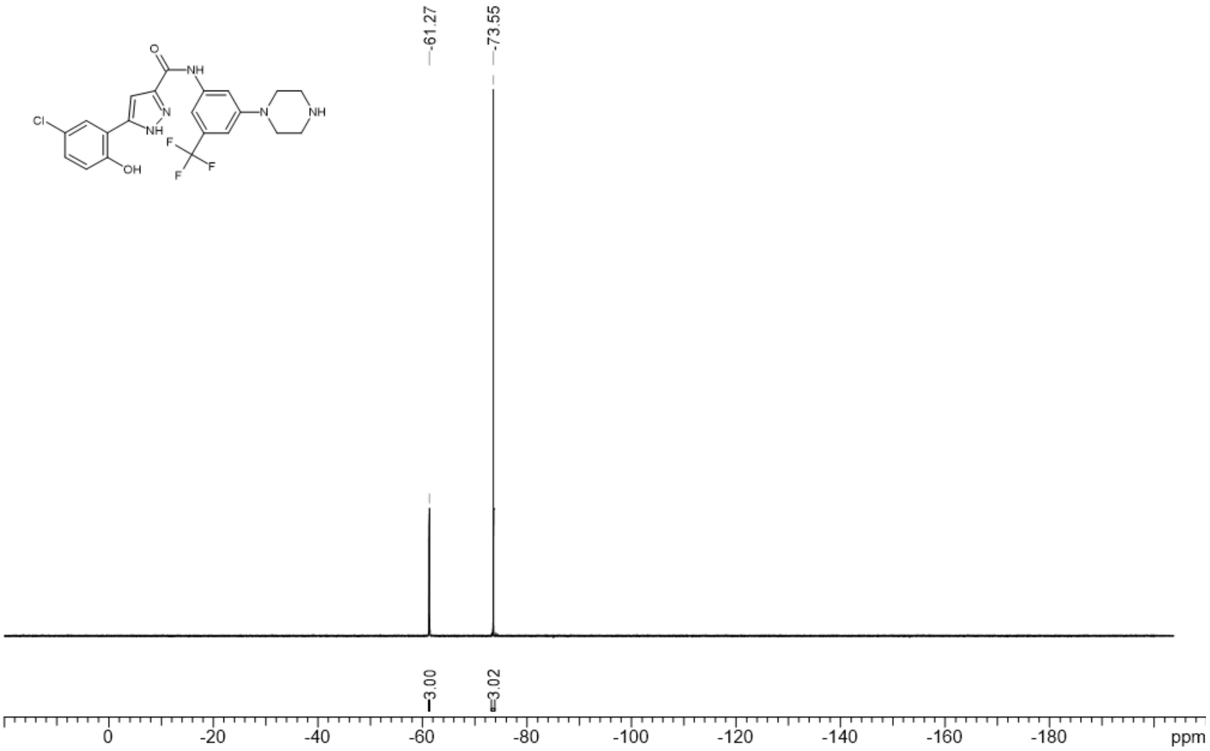

Figure S161. ^{19}F NMR spectrum of **11A.**

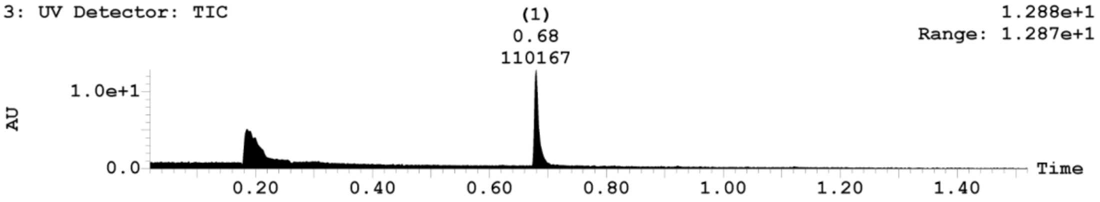

Peak Number	Time	Area %Total
1	0.68	100.00

Figure S162. LCMS purity analysis of **11A.**

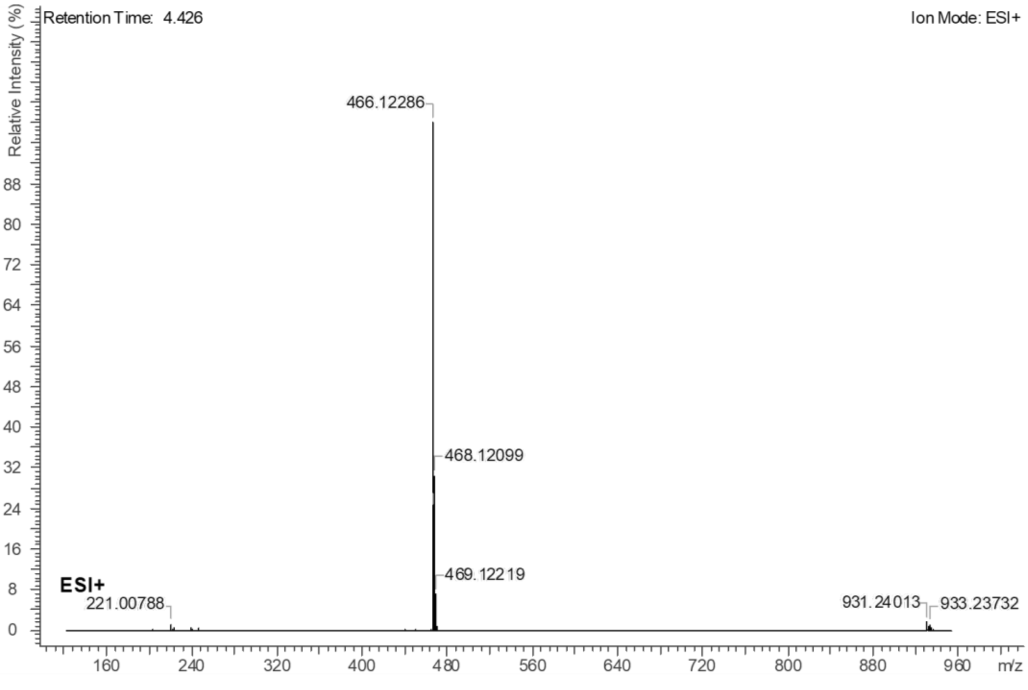

Figure S163. HRMS of **11A.**

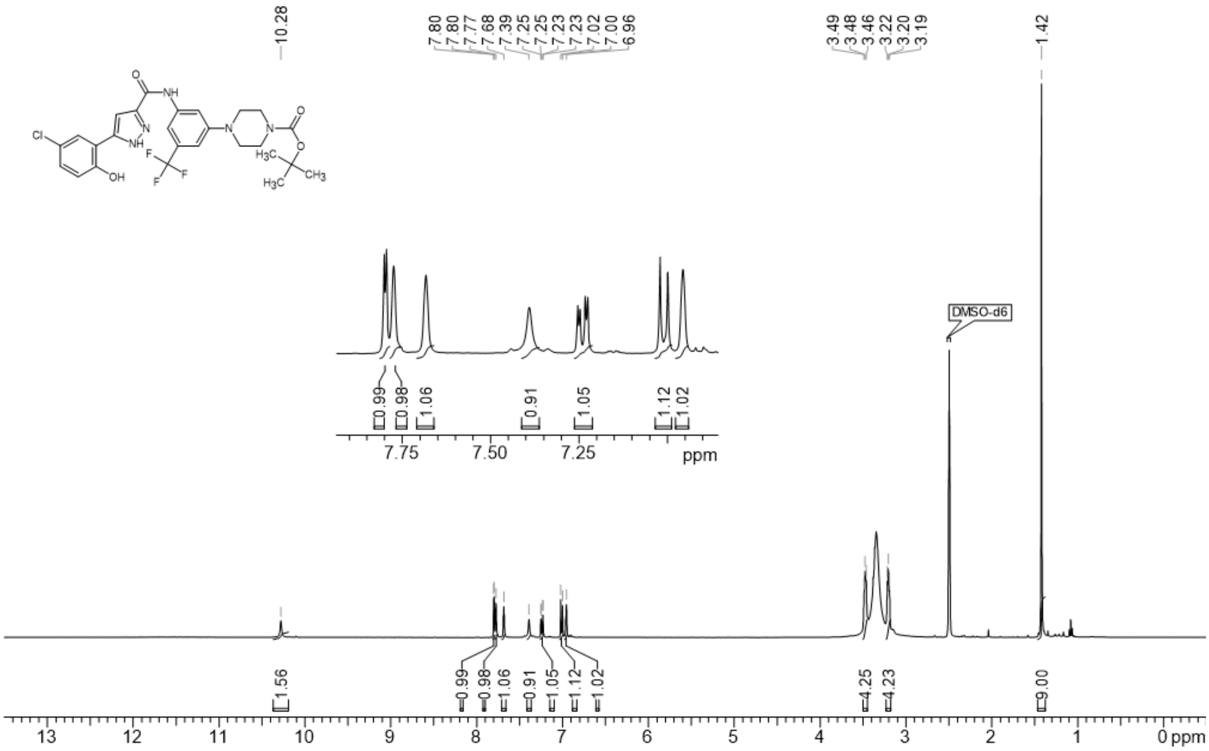

Figure S164. ^{1}H NMR spectrum of **11B.**

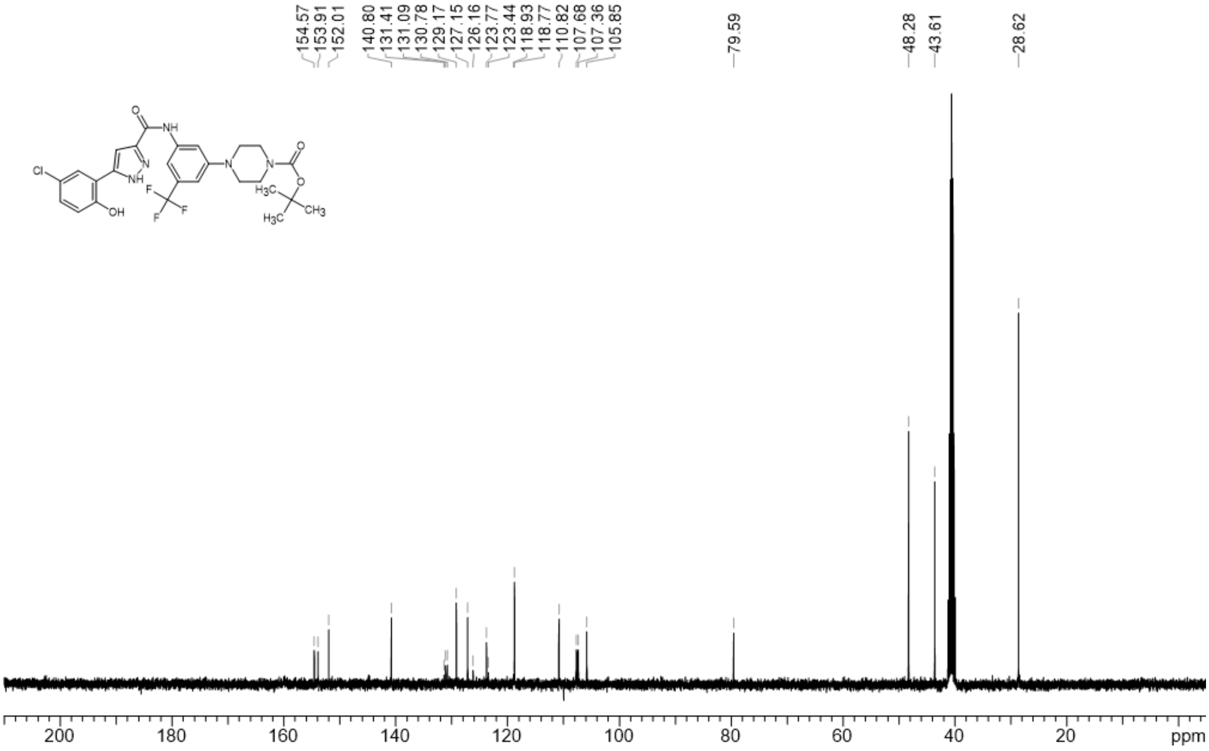

Figure S165. ^{13}C NMR spectrum at 120 °C of **11B.**

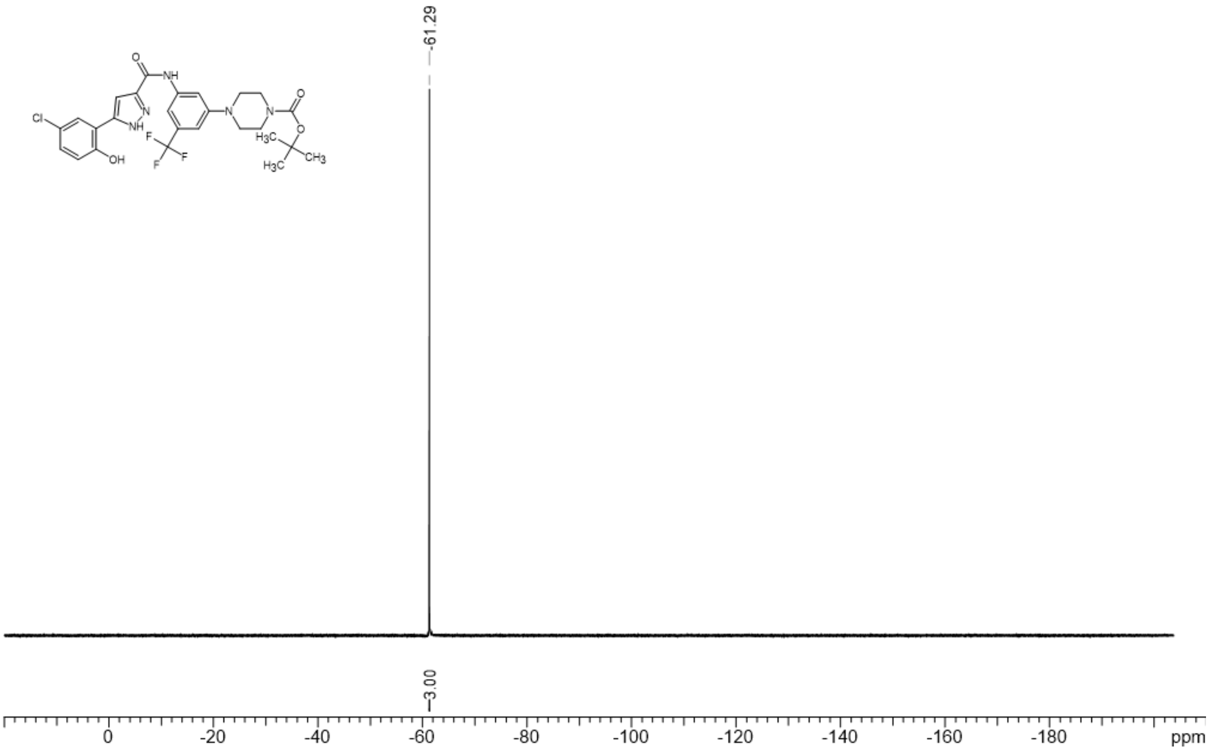

Figure S166. ^{19}F NMR spectrum of **11B.**

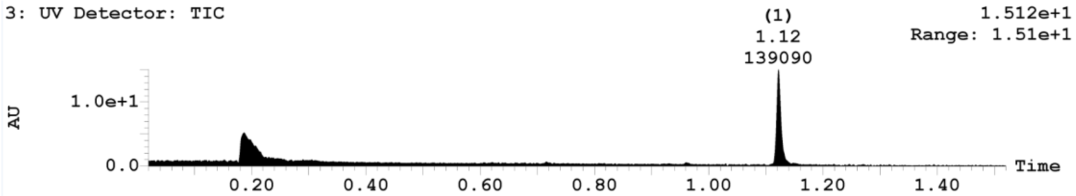

Peak Number	Time	Area %Total
1	1.12	100.00

Figure S167. LCMS purity analysis of **11B.**

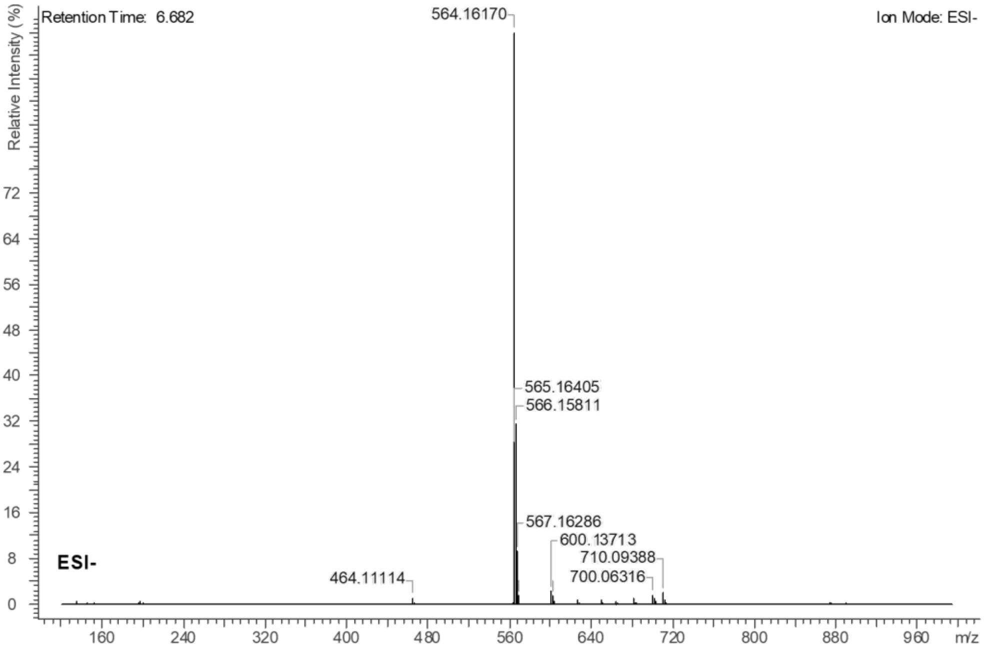

Figure S168. HRMS of **11B.**

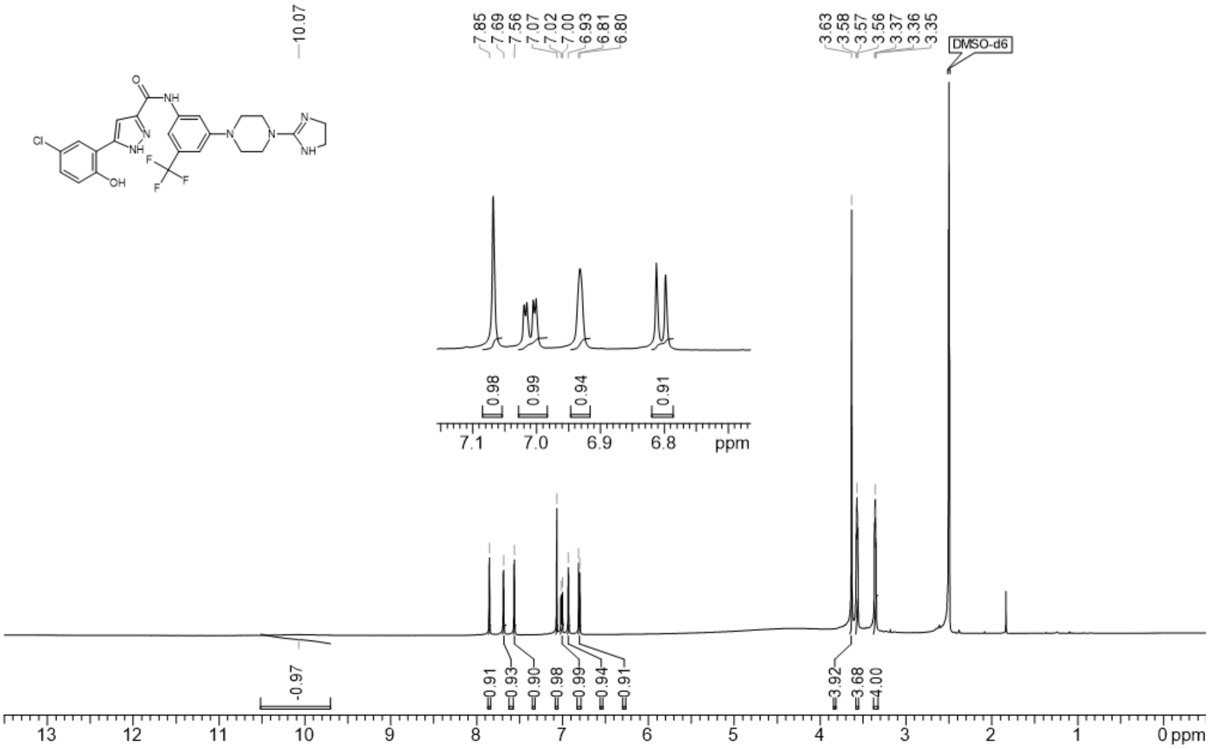

Figure S169. ^{1}H NMR spectrum of **11C.**

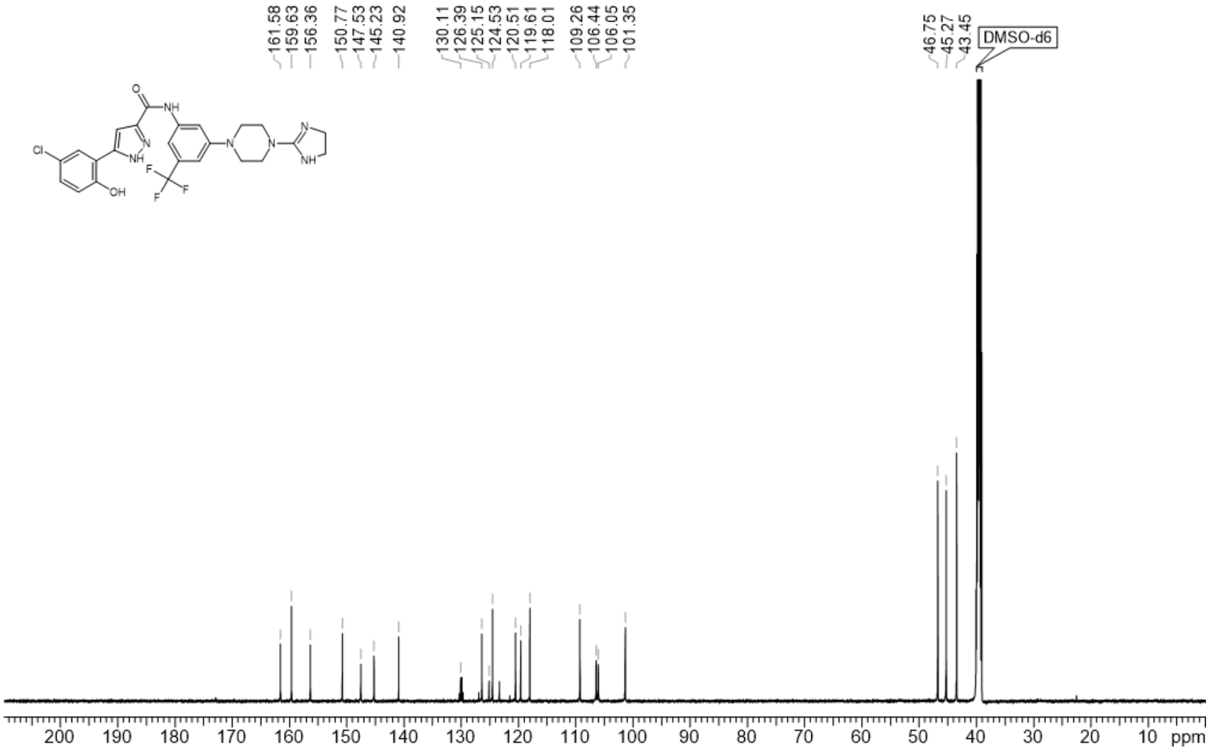

Figure S170. ^{13}C NMR spectrum at 50 °C of **13B.**

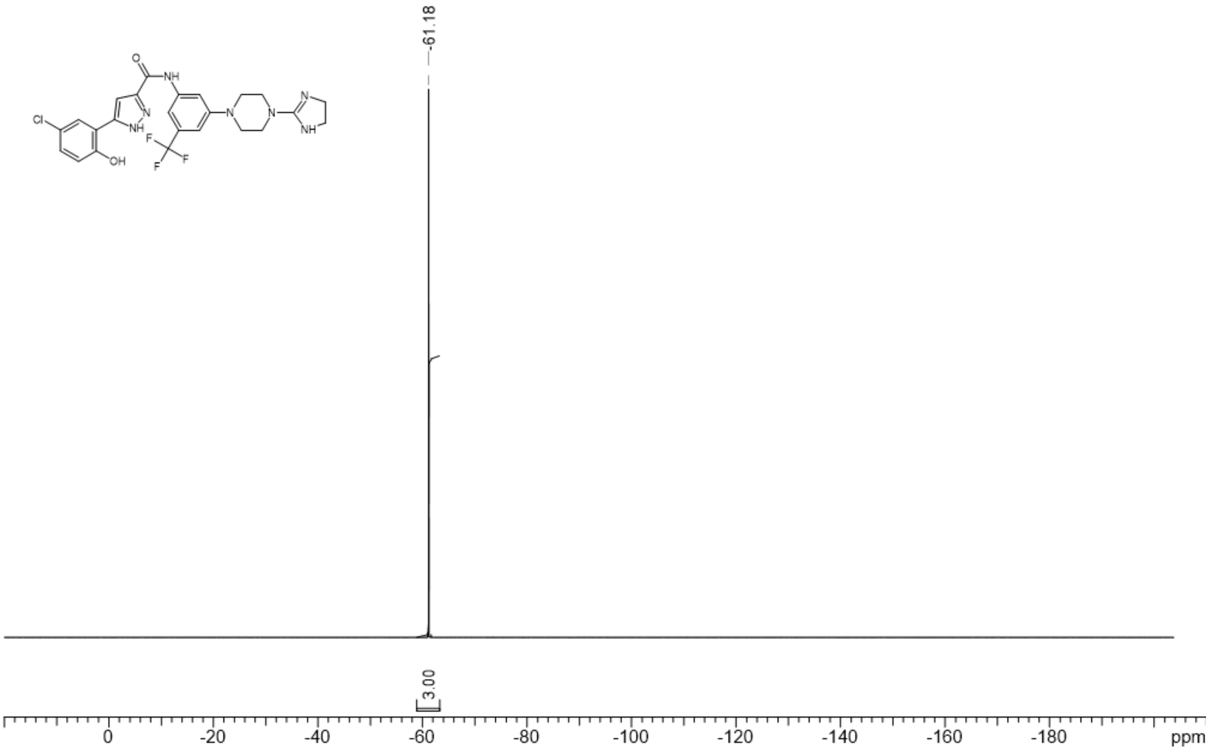

Figure S171. ^{19}F NMR spectrum of **11C.**

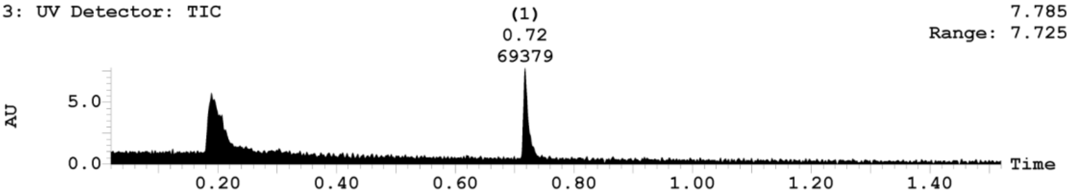

Peak Number	Time	Area %Total
1	0.72	100.00

Figure S172. LCMS purity analysis of **11C.**

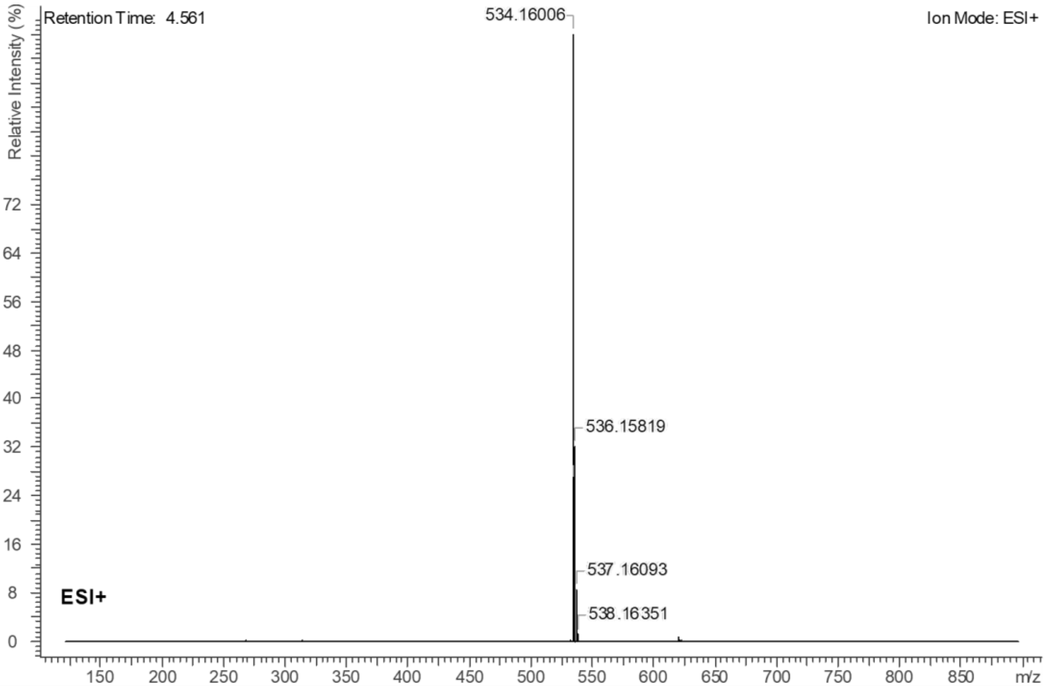

Figure S173. HRMS of **11C.**

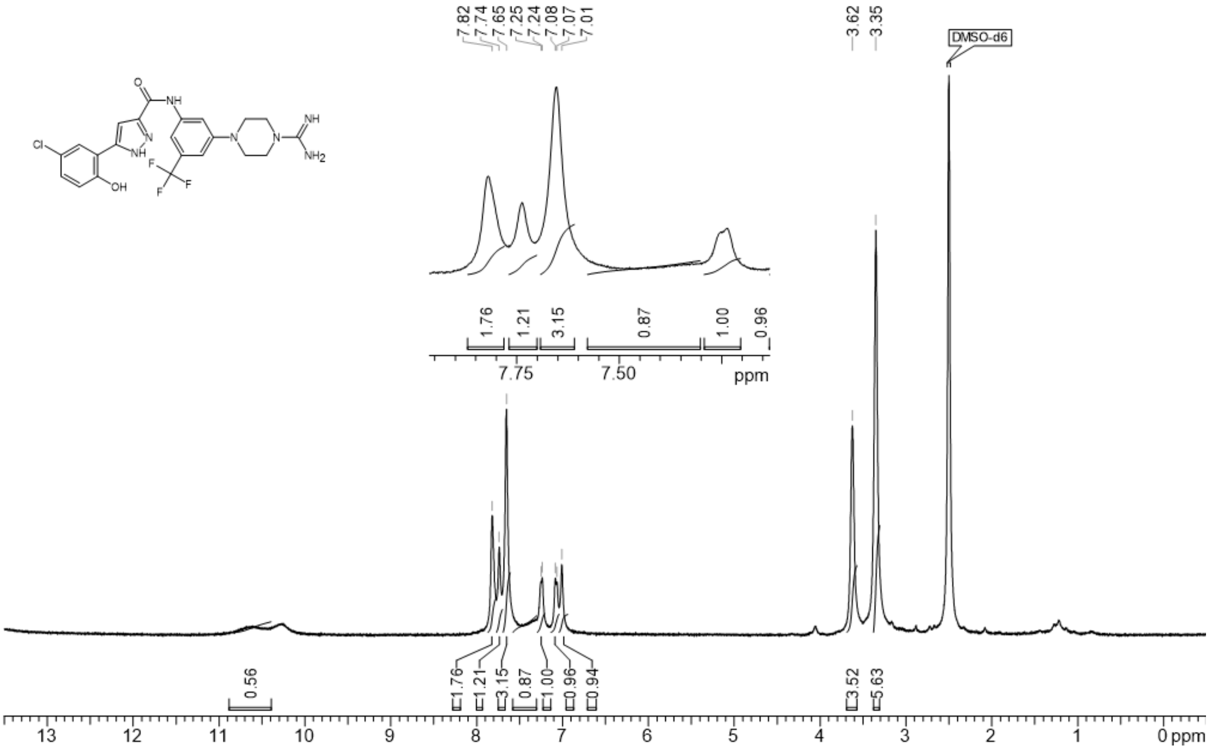

Figure S174. ^{1}H NMR spectrum of **11G.**

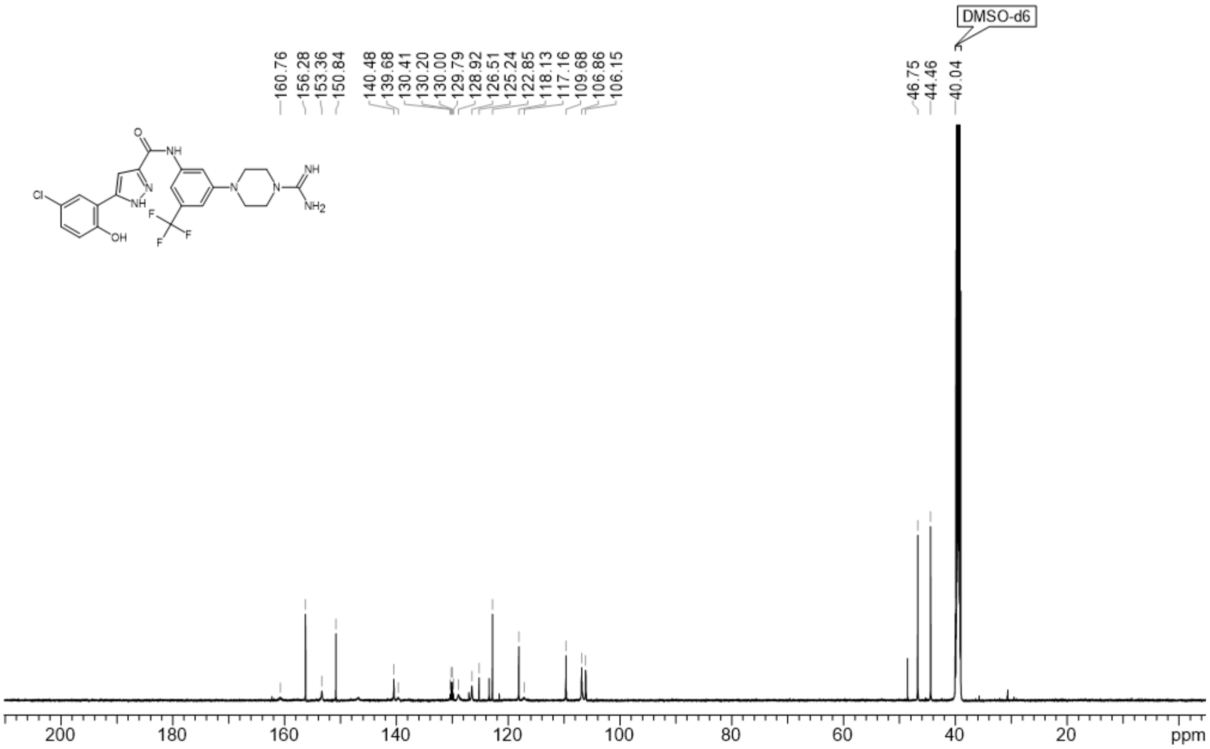

Figure S175. ^{13}C NMR spectrum of **11G.**

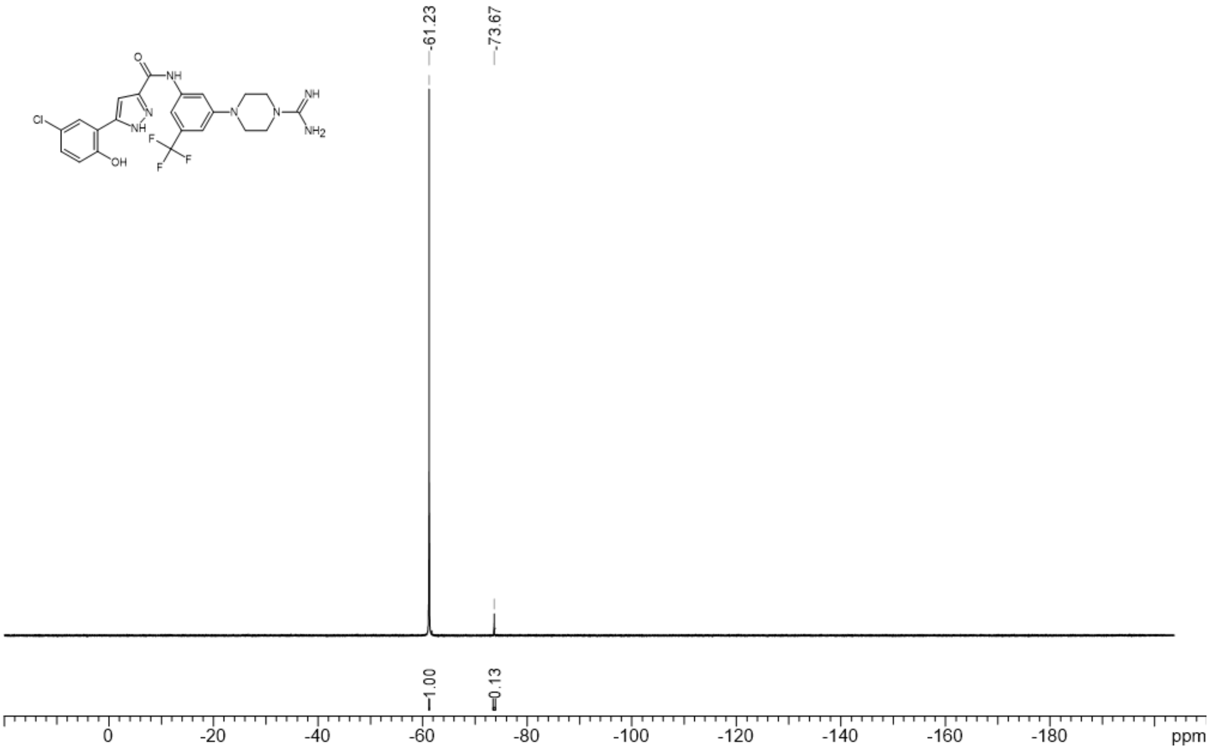

Figure S176. ^{19}F NMR spectrum of **11G.**

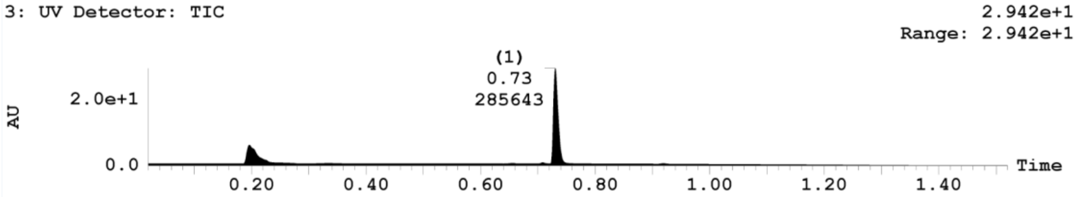

Peak Number	Time	Area %Total
1	0.73	100.00

Figure S177. LCMS purity analysis of **11G.**

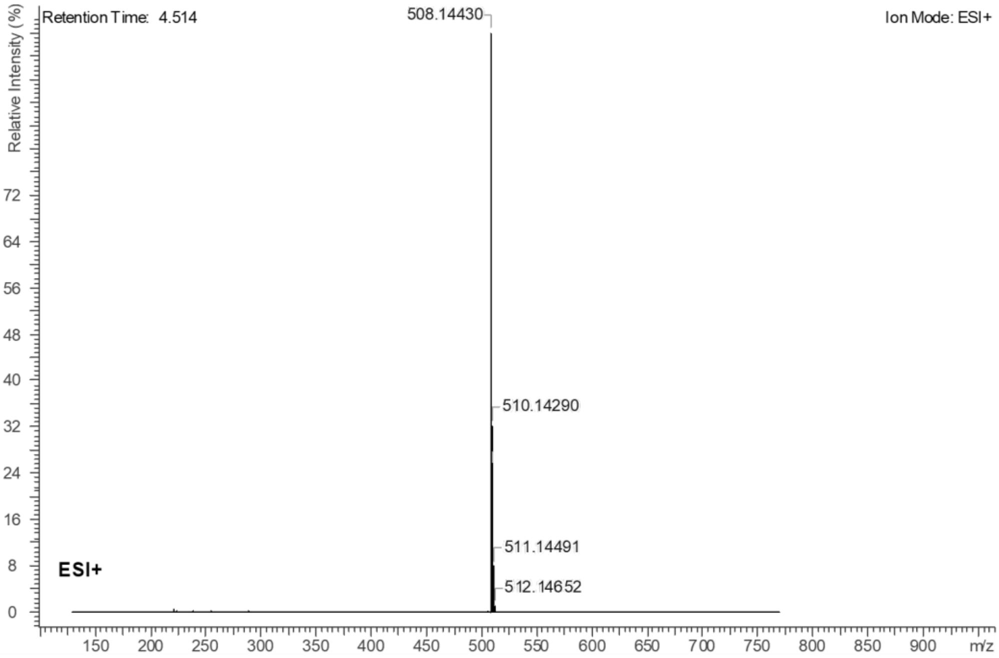

Figure S178. HRMS of **11G.**

Compounds **12A–G**

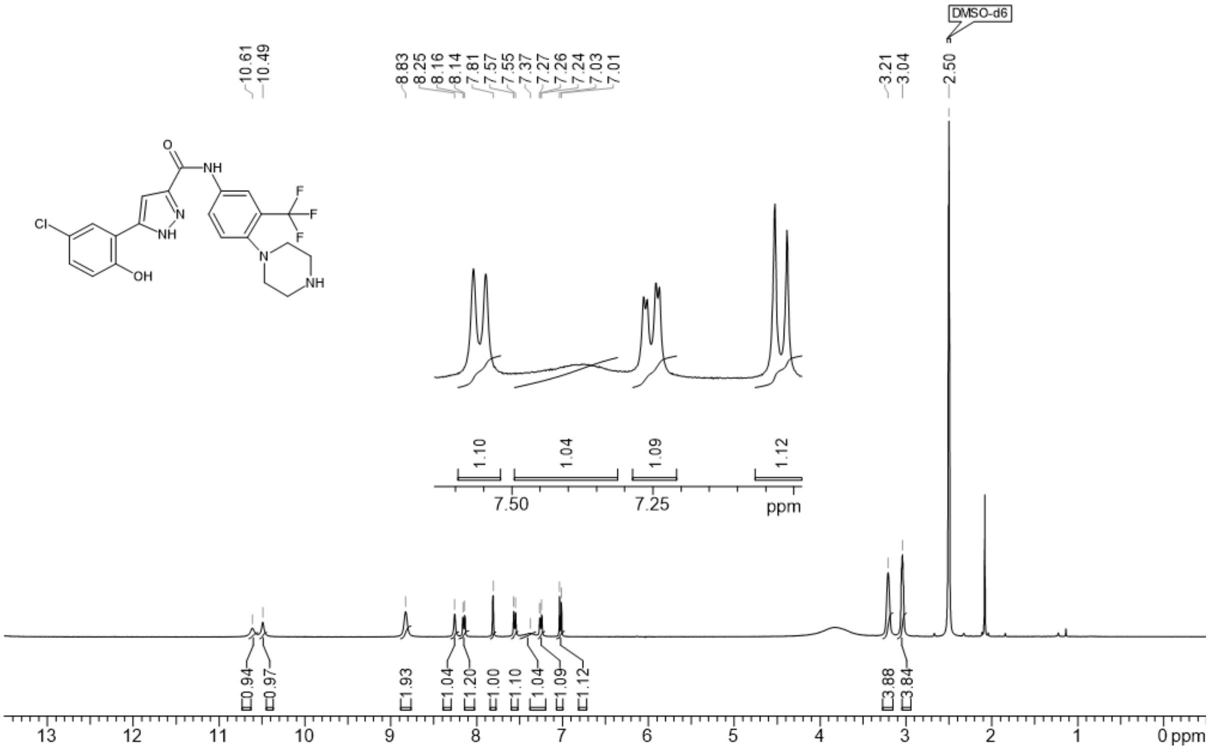

Figure S179. ^{1}H NMR spectrum of **12A.**

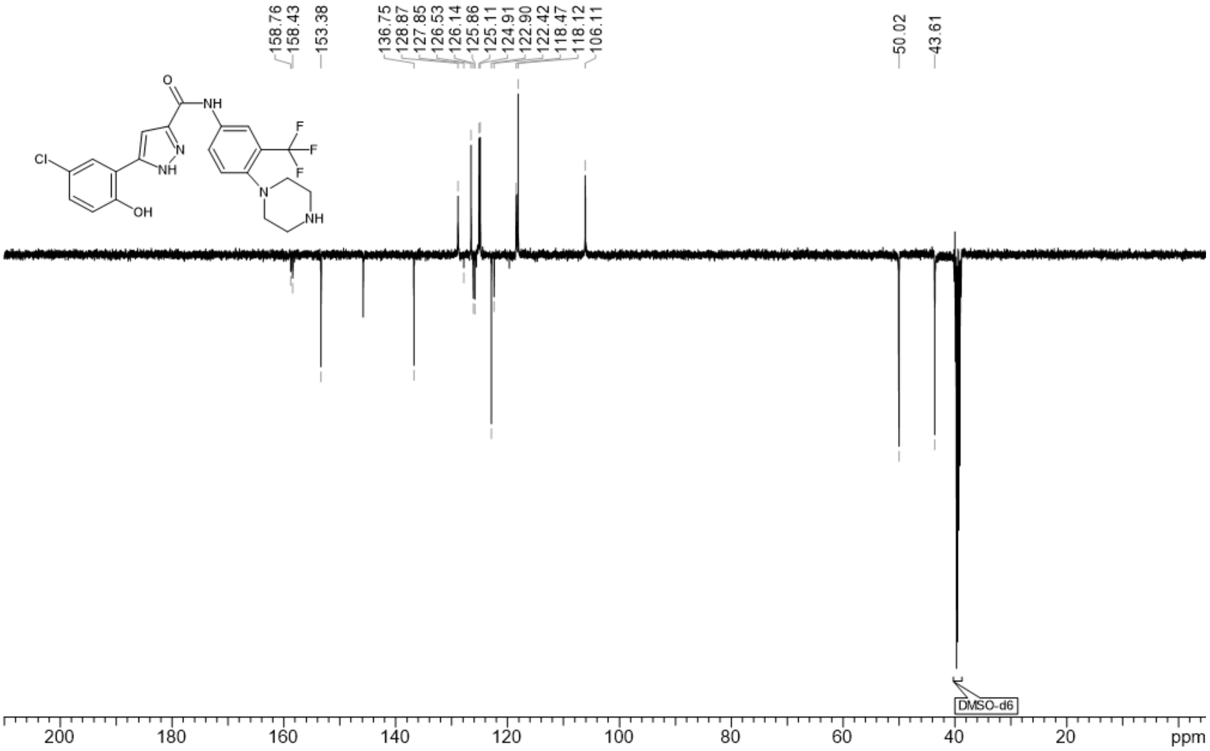

Figure S180. ^{13}C NMR spectrum of **12A.**

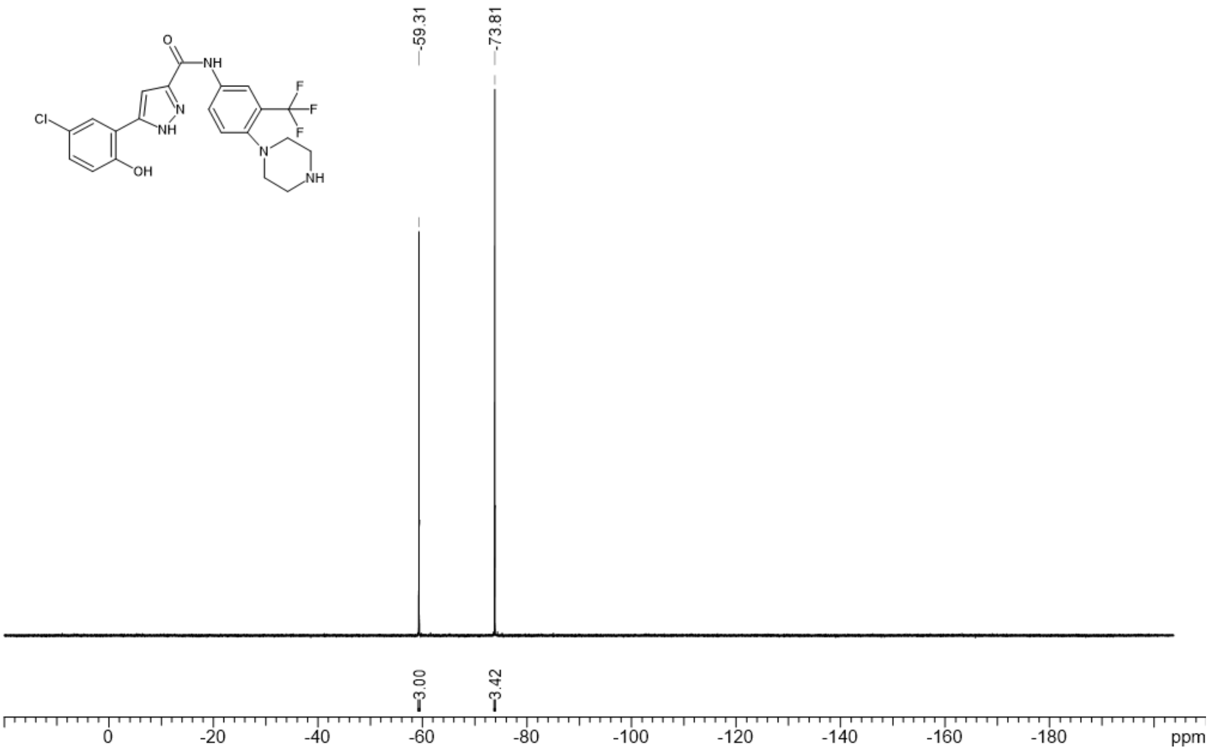

Figure S181. ^{19}F NMR spectrum of **12A.**

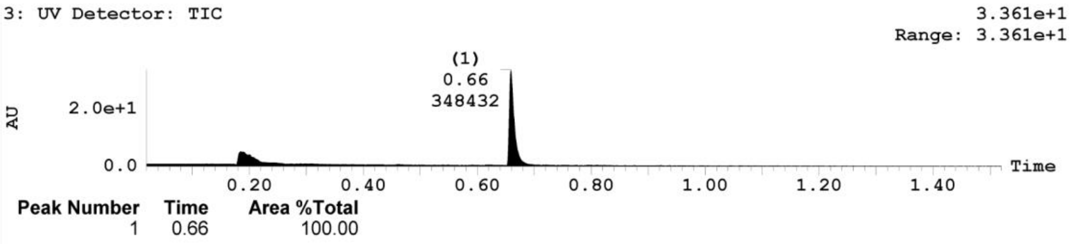

Figure S182. LCMS purity analysis of **12A.**

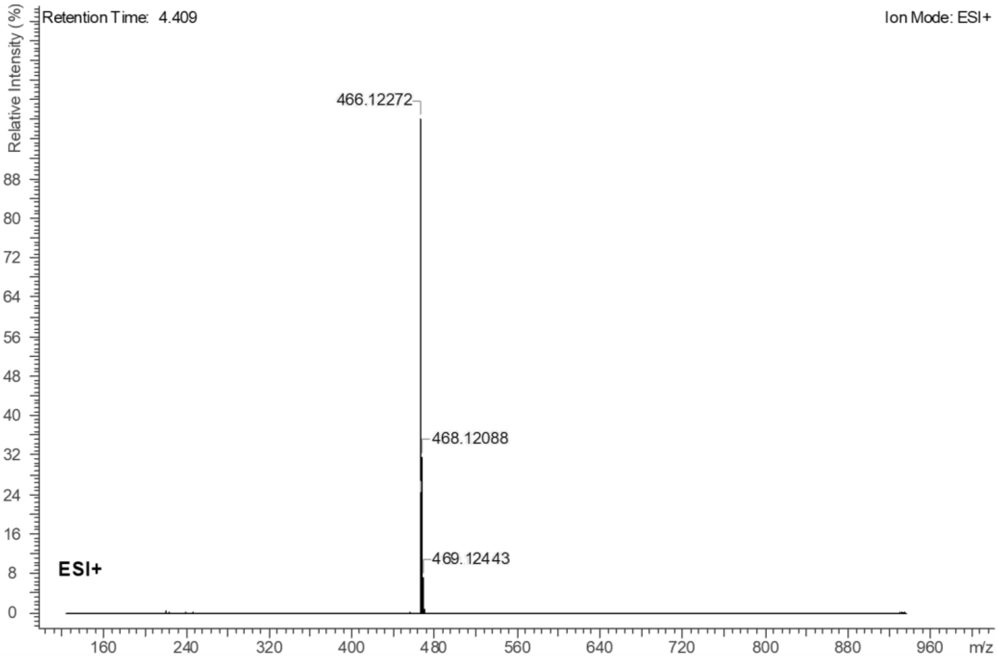

Figure S183. HRMS of **12A.**

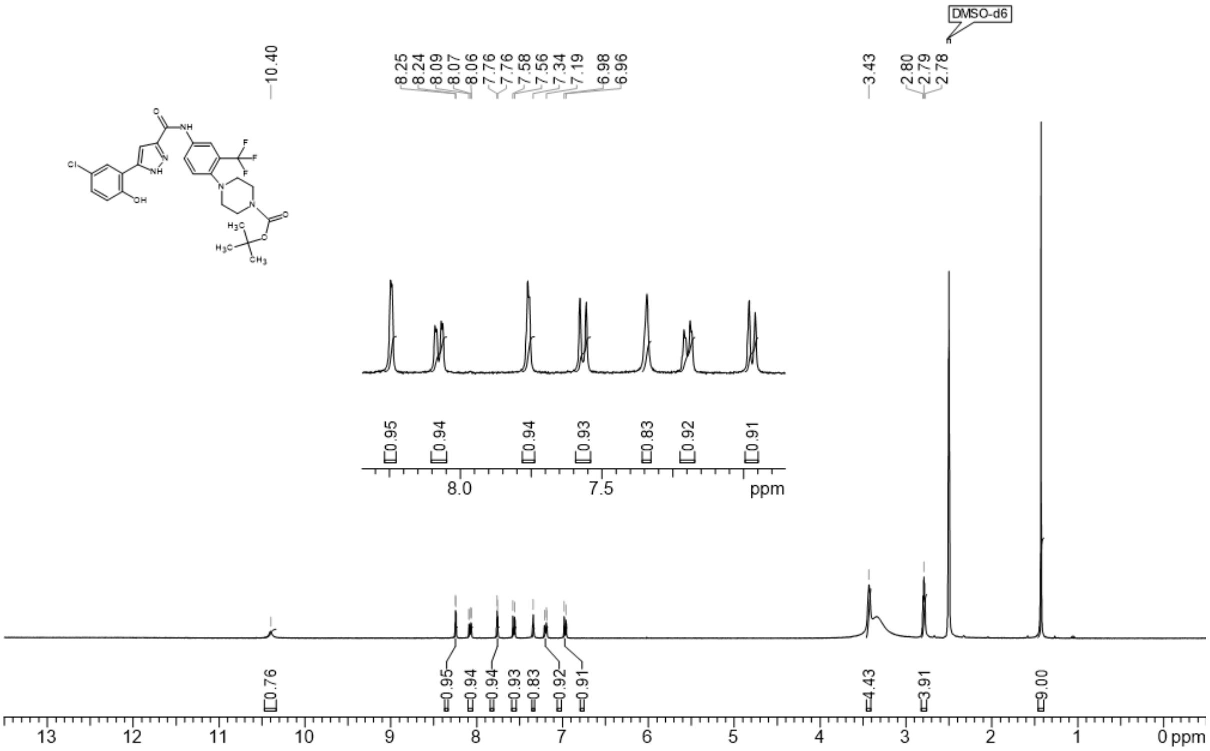

Figure S184. ^{1}H NMR spectrum of **12B.**

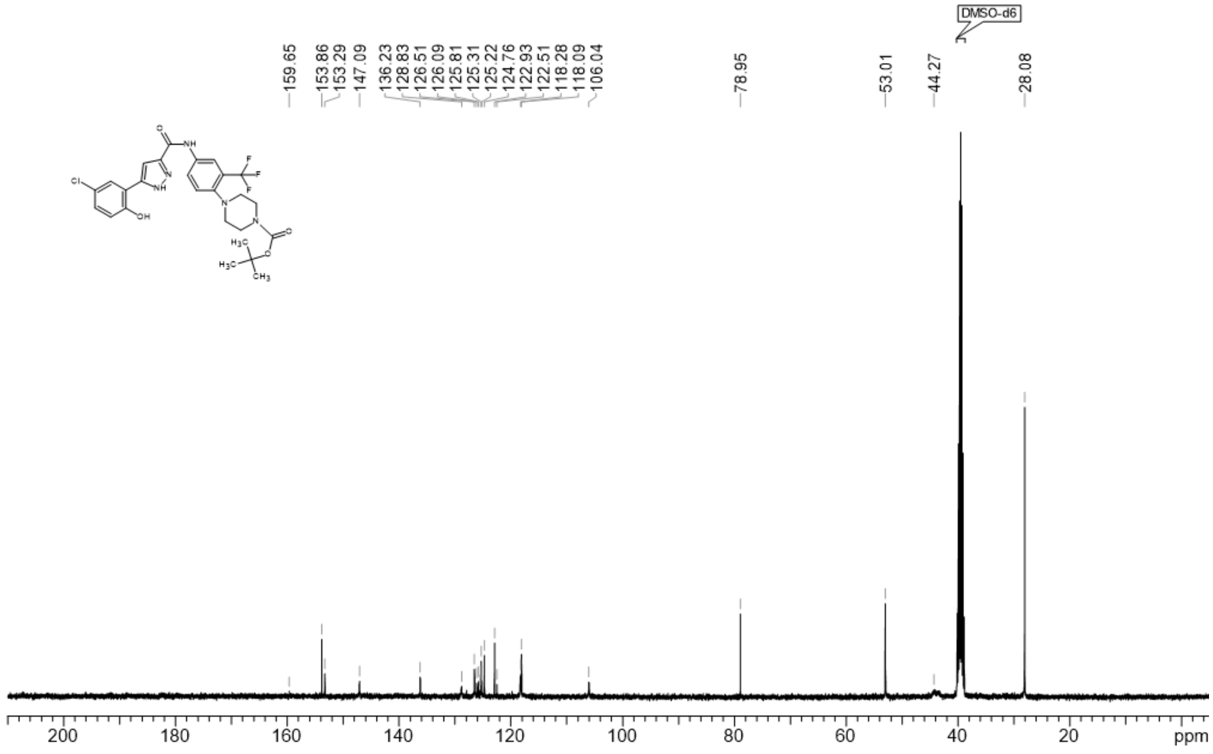

Figure S185. ^{13}C NMR spectrum of **12B.**

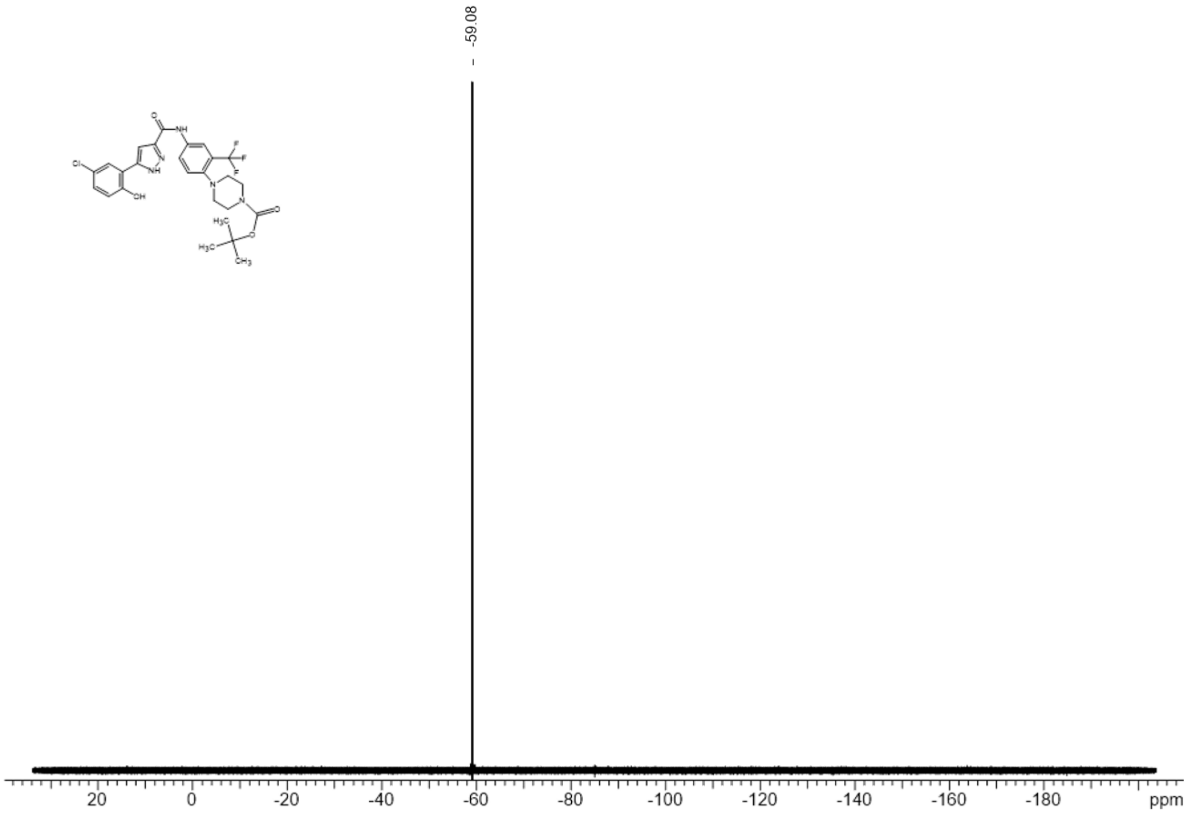

Figure S186. ^{19}F NMR spectrum of **12B.**

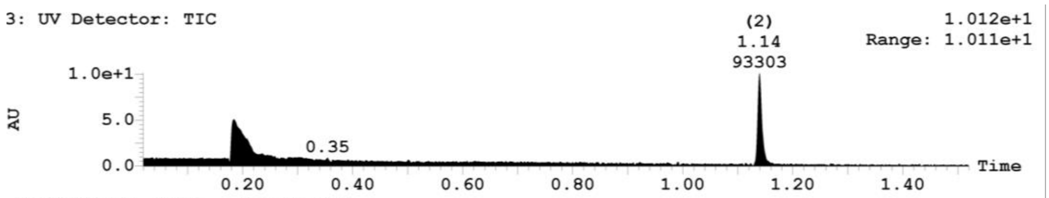

Peak Number	Time	Area %Total
1	0.35	2.08
2	1.14	97.92

Figure S187. LCMS purity analysis of **12B.**

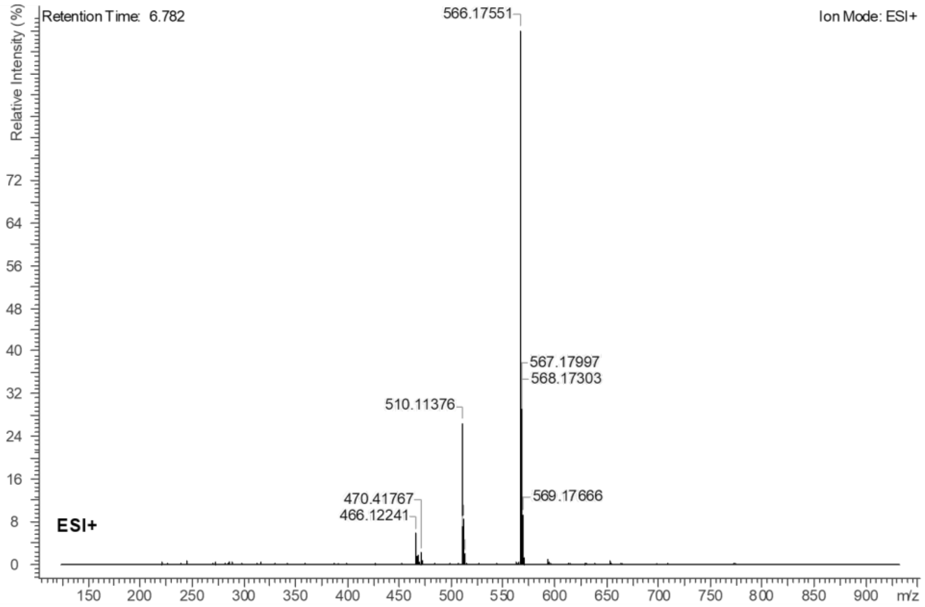

Figure S188. HRMS of **12B.**

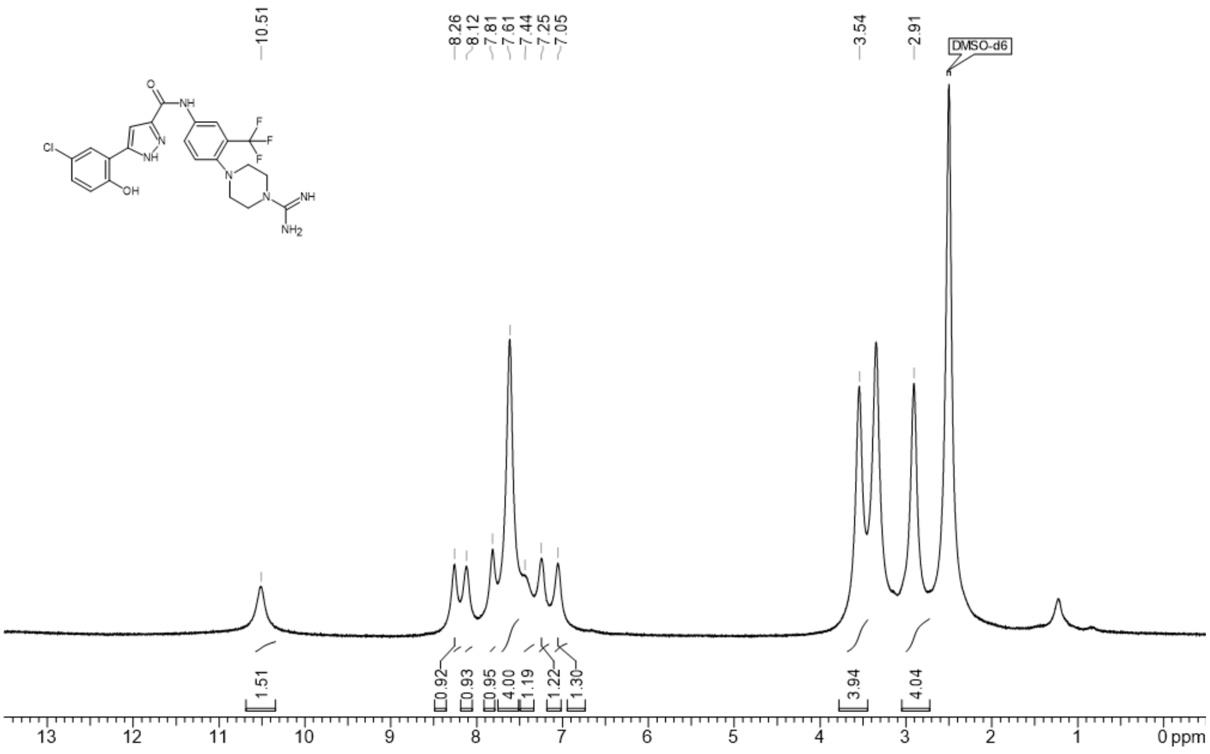

Figure S189. ^{1}H NMR spectrum of **12G** in DMSO-*d6*.

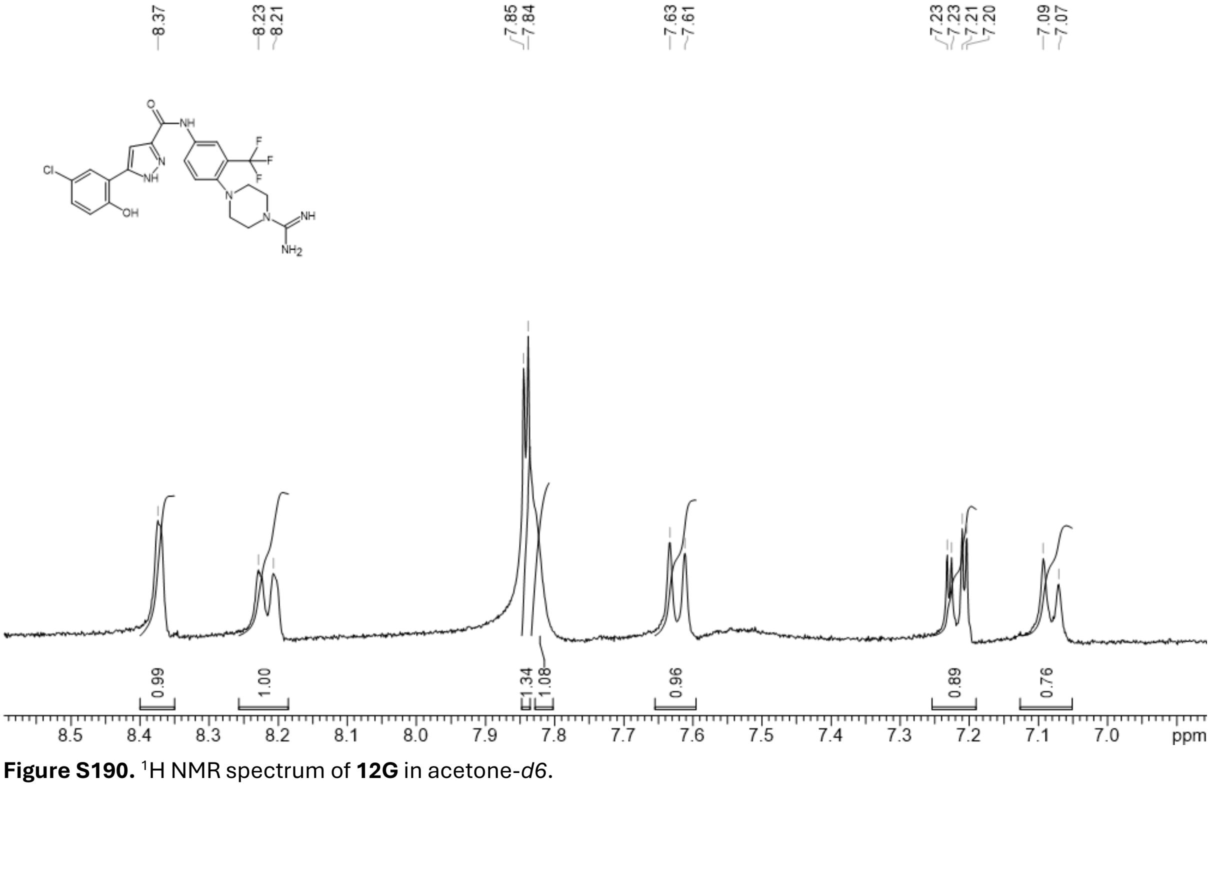

Figure S190. ^{1}H NMR spectrum of **12G** in acetone-*d6*.

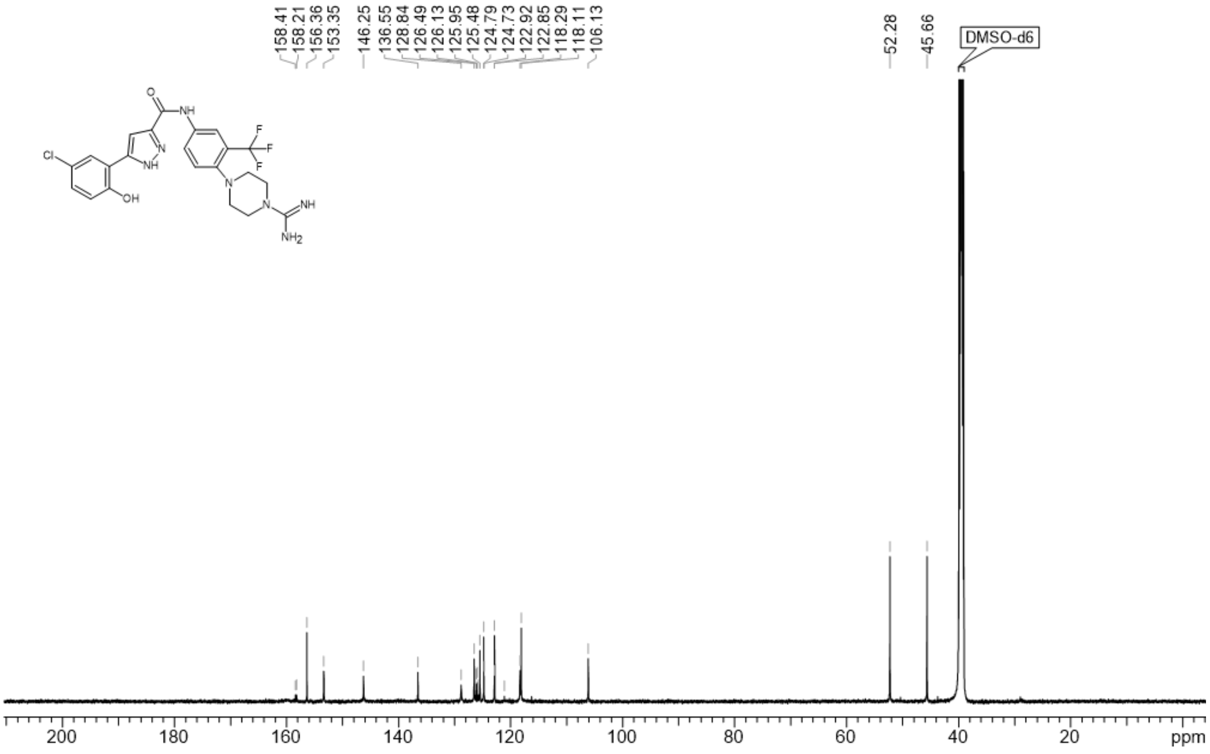

Figure S191. ^{13}C NMR spectrum of **12G.**

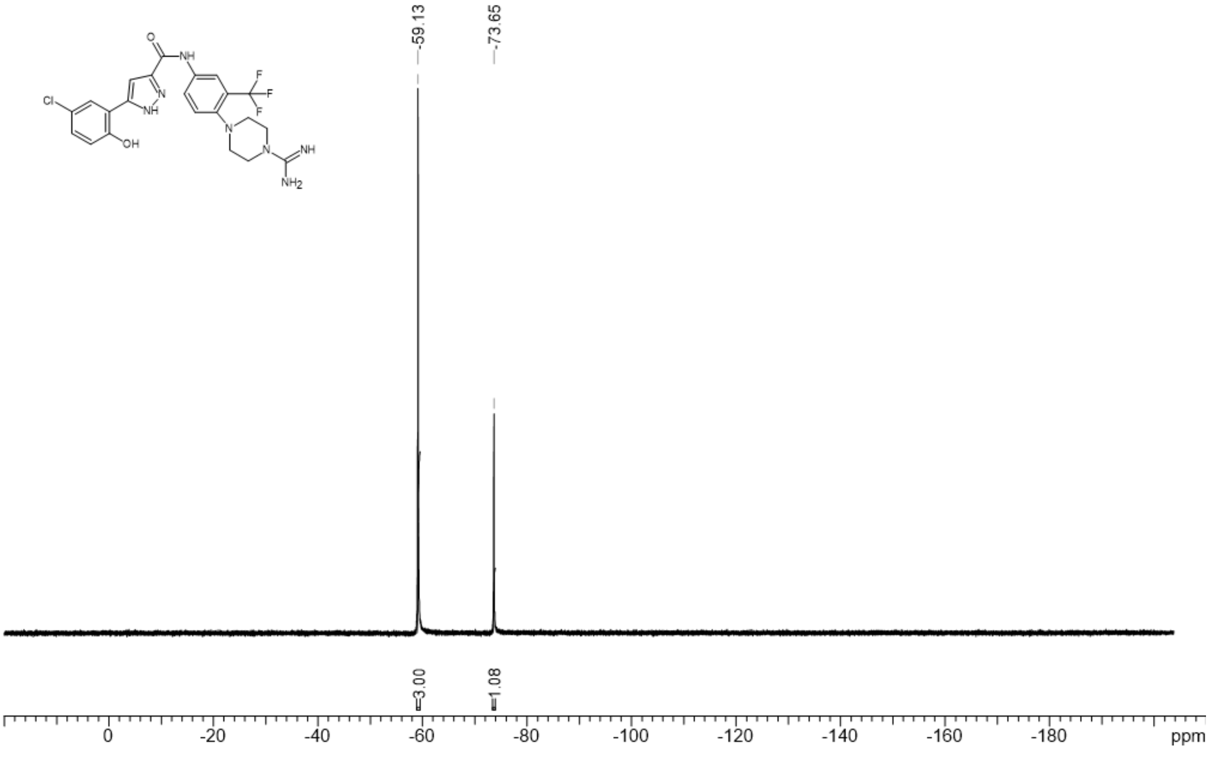

Figure S192. ^{19}F NMR spectrum of **12G.**

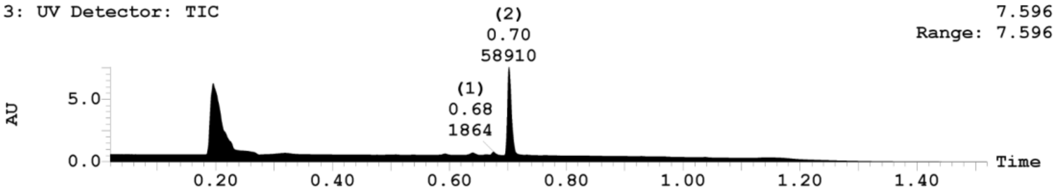

Peak Number	Time	Area %Total
1	0.67	3.07
2	0.70	96.93

Figure S193. LCMS purity analysis of **12G.**

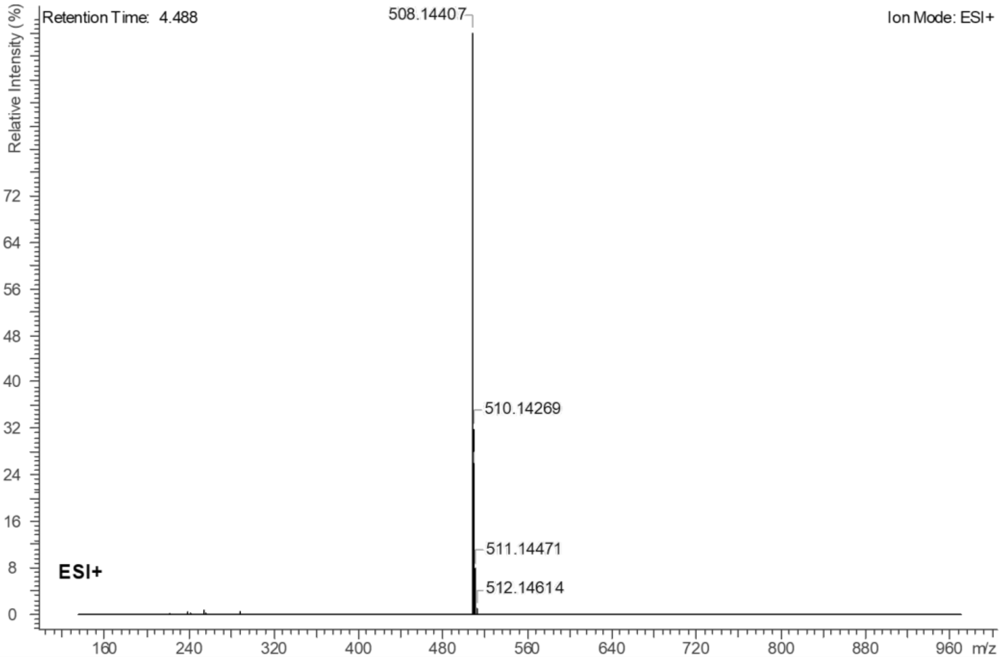

Figure S194. HRMS of **12G.**

Compounds **13A–G**

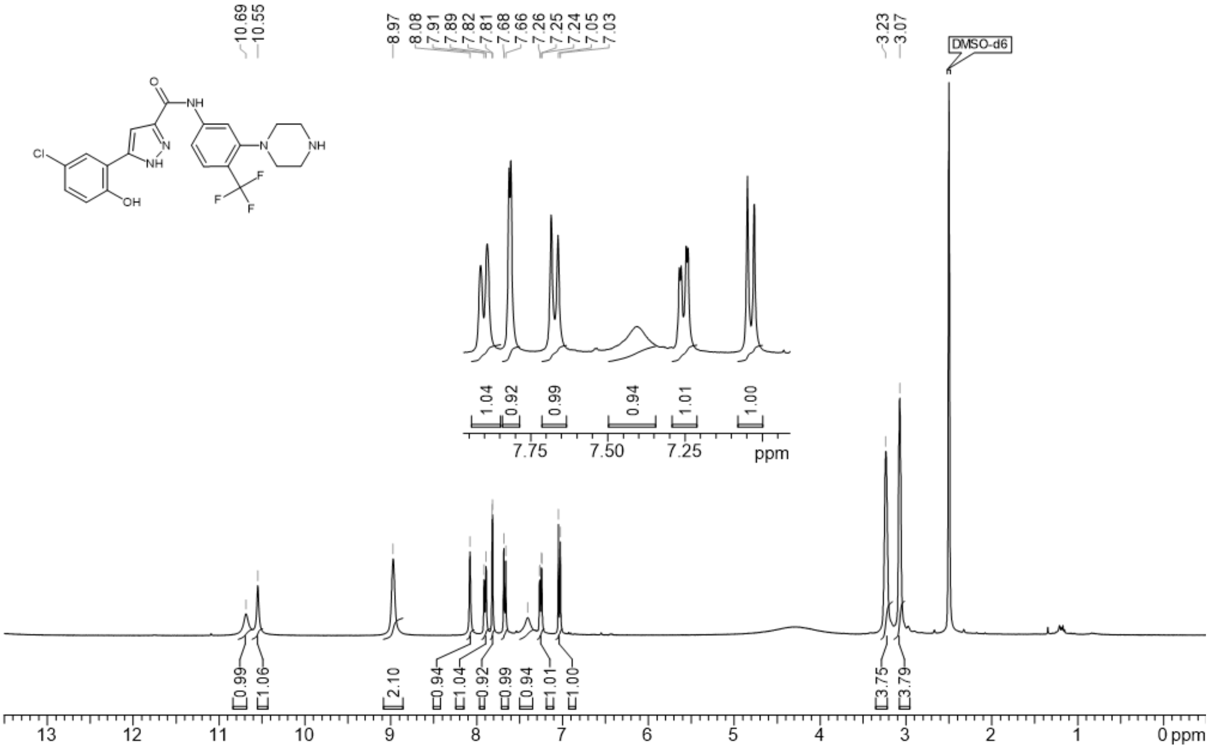

Figure S195. ^{1}H NMR spectrum of **13A.**

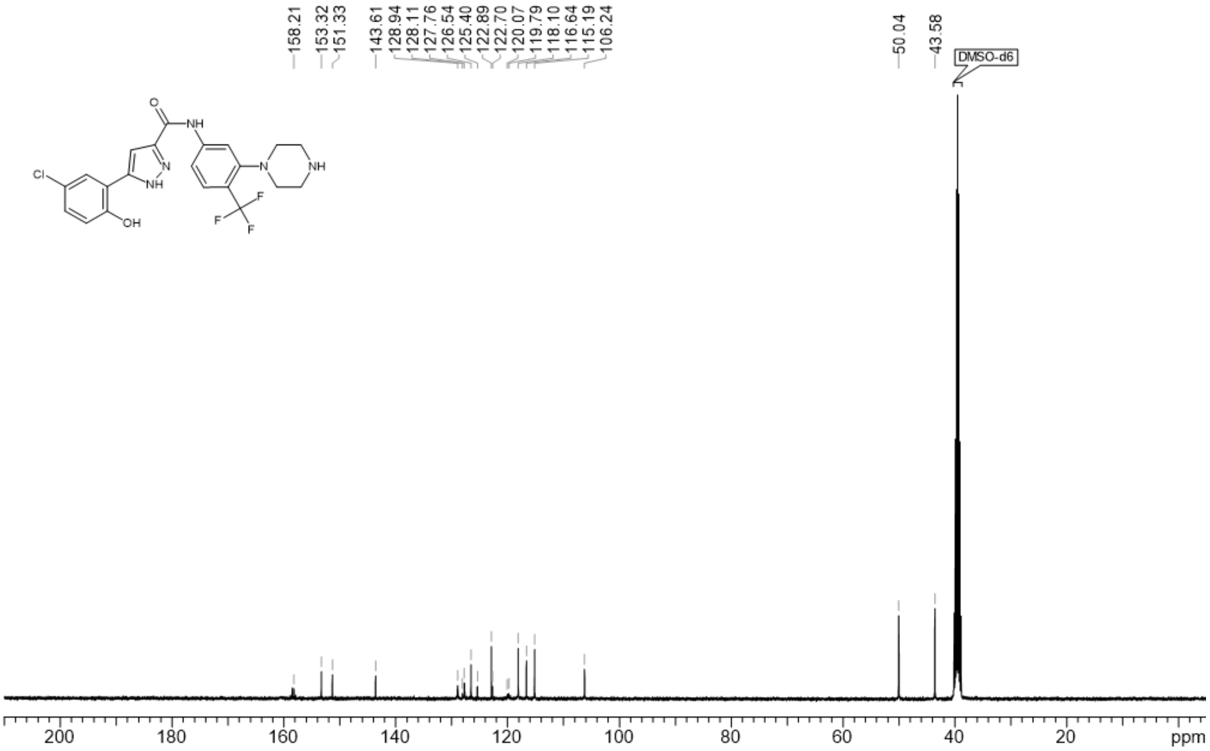

Figure S196. ^{13}C NMR spectrum of **13A.**

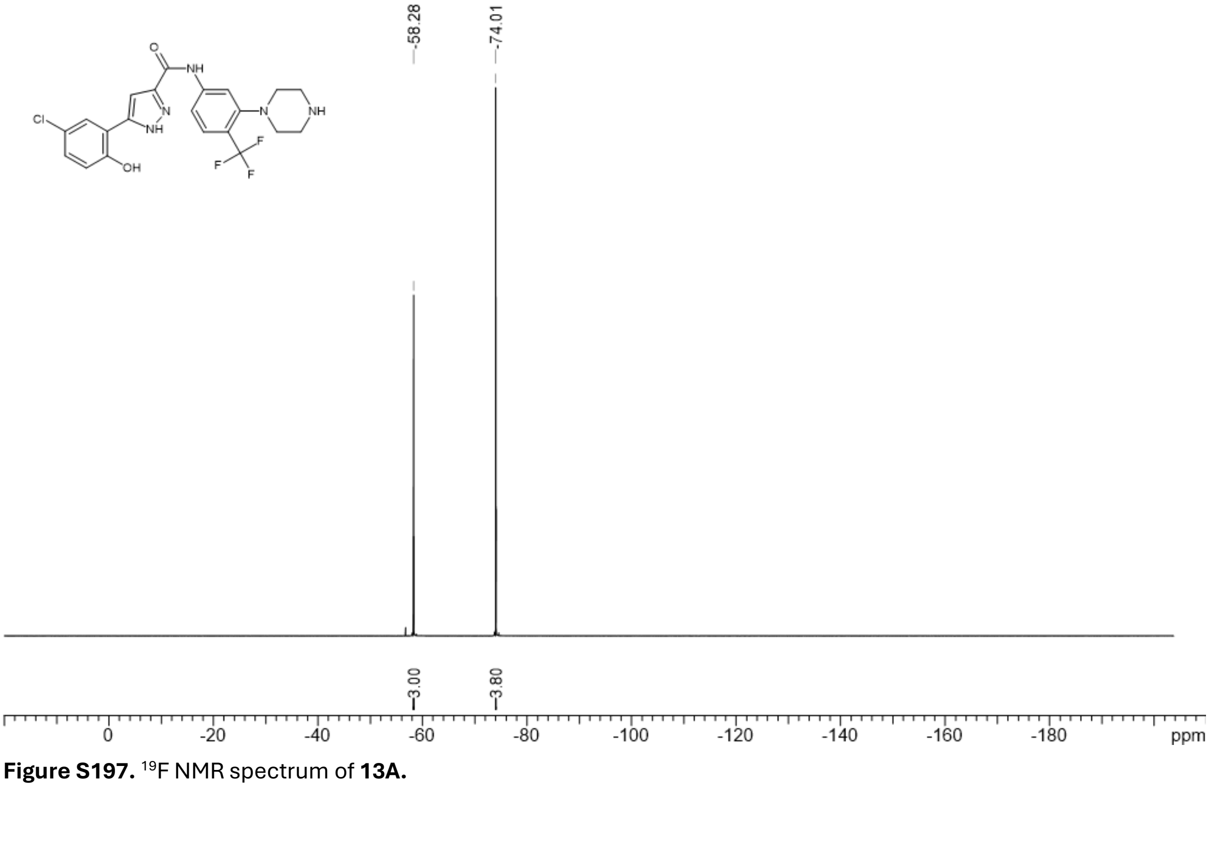

Figure S197. ^{19}F NMR spectrum of **13A.**

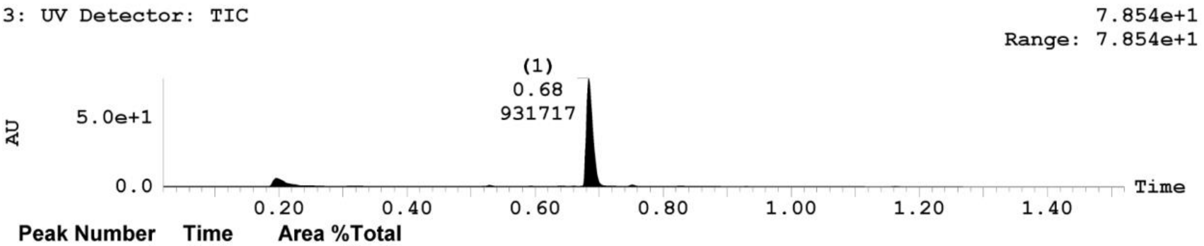

Peak Number	Time	Area %Total
1	0.68	100.00

Figure S198. LCMS purity analysis of **13A.**

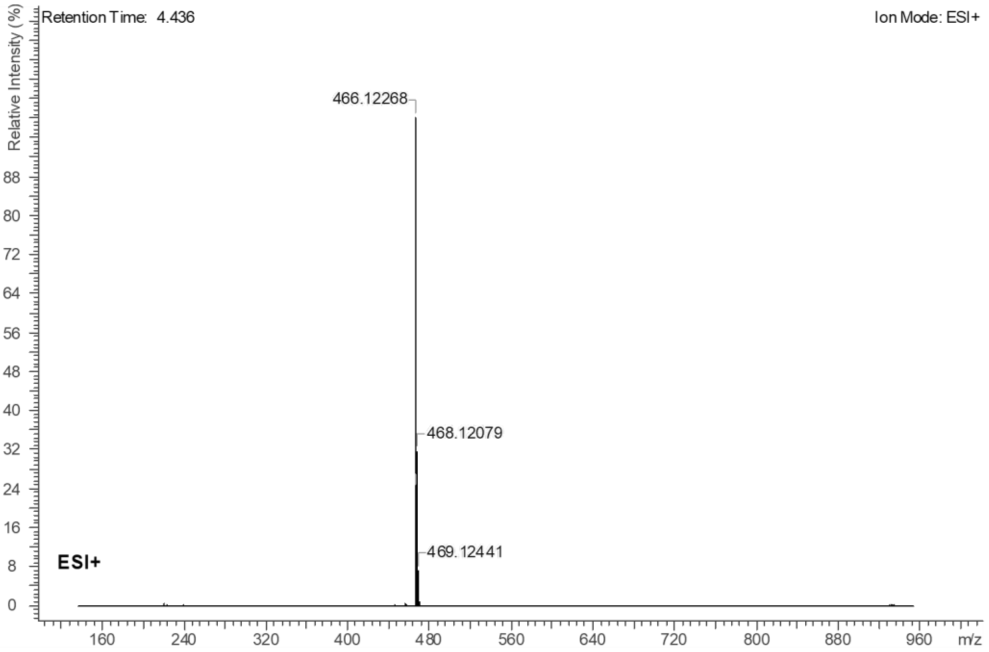

Figure S199. HRMS of **13A.**

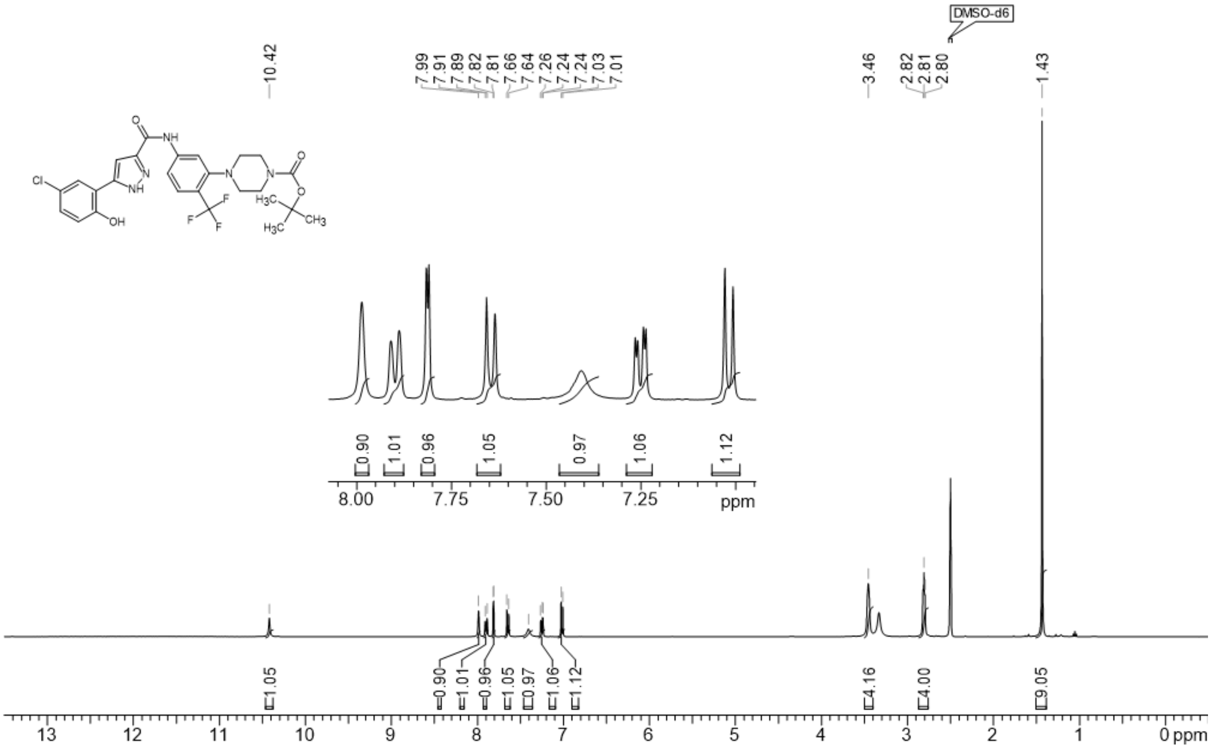

Figure S200. ^{1}H NMR spectrum of **13B.**

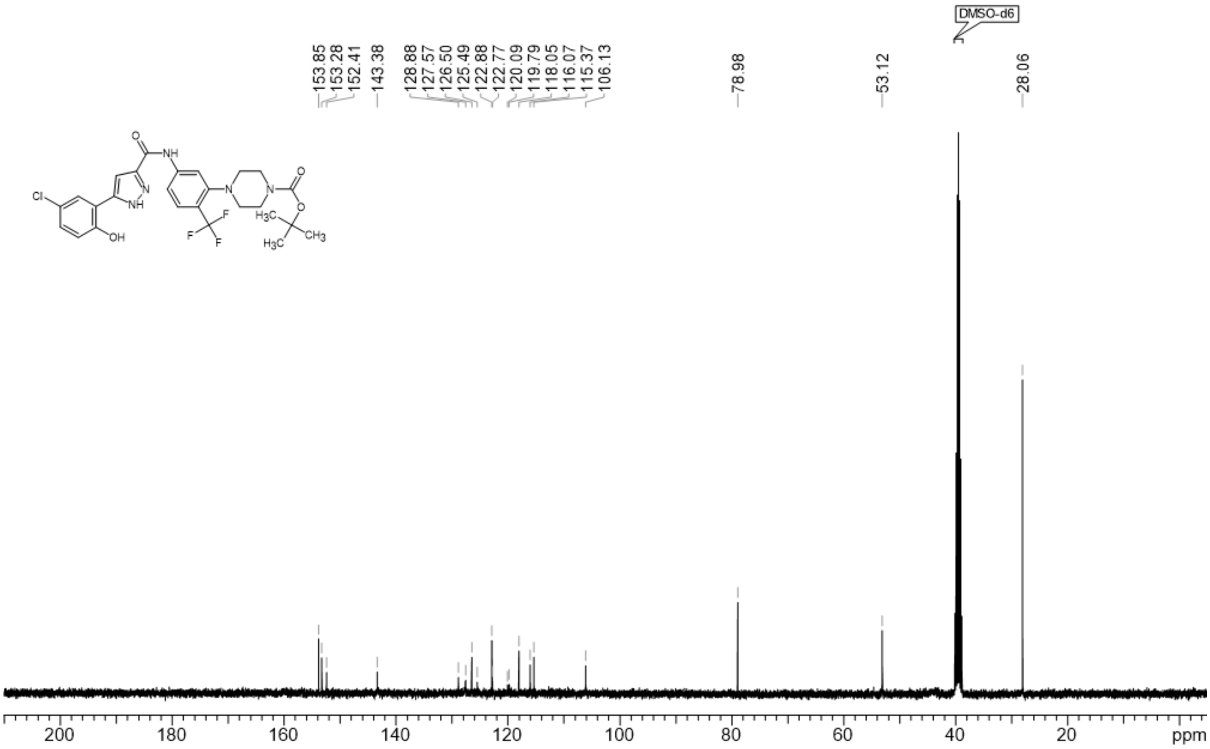

Figure S201. ^{13}C NMR spectrum of **13B.**

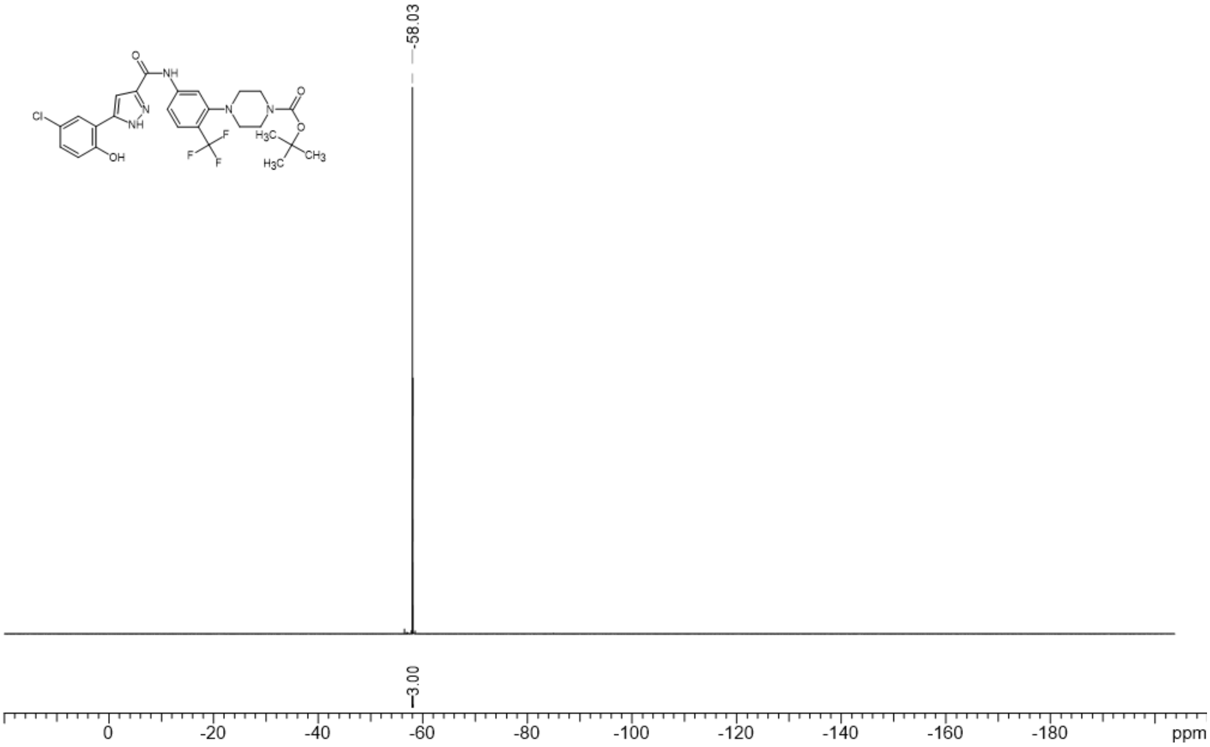

Figure S202. ^{19}F NMR spectrum of **13B.**

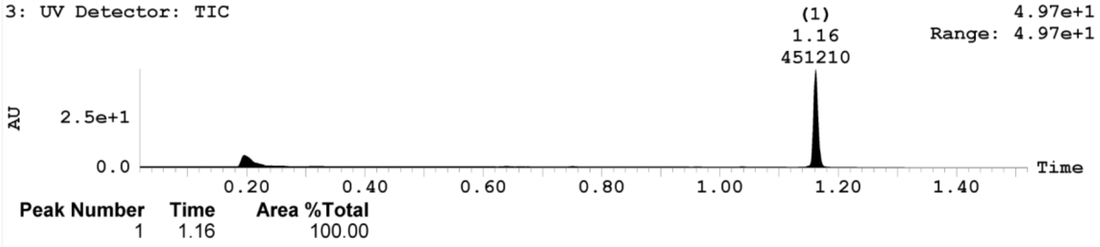

Peak Number	Time	Area %Total
1	1.16	100.00

Figure S203. LCMS purity analysis of **13B.**

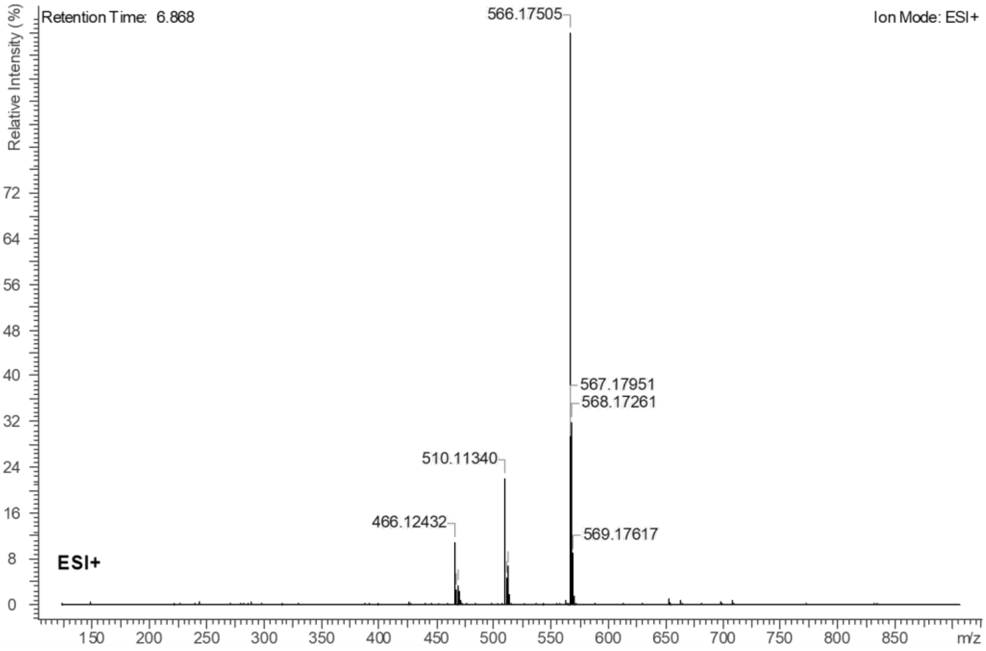

Figure S204. HRMS of **13B.**

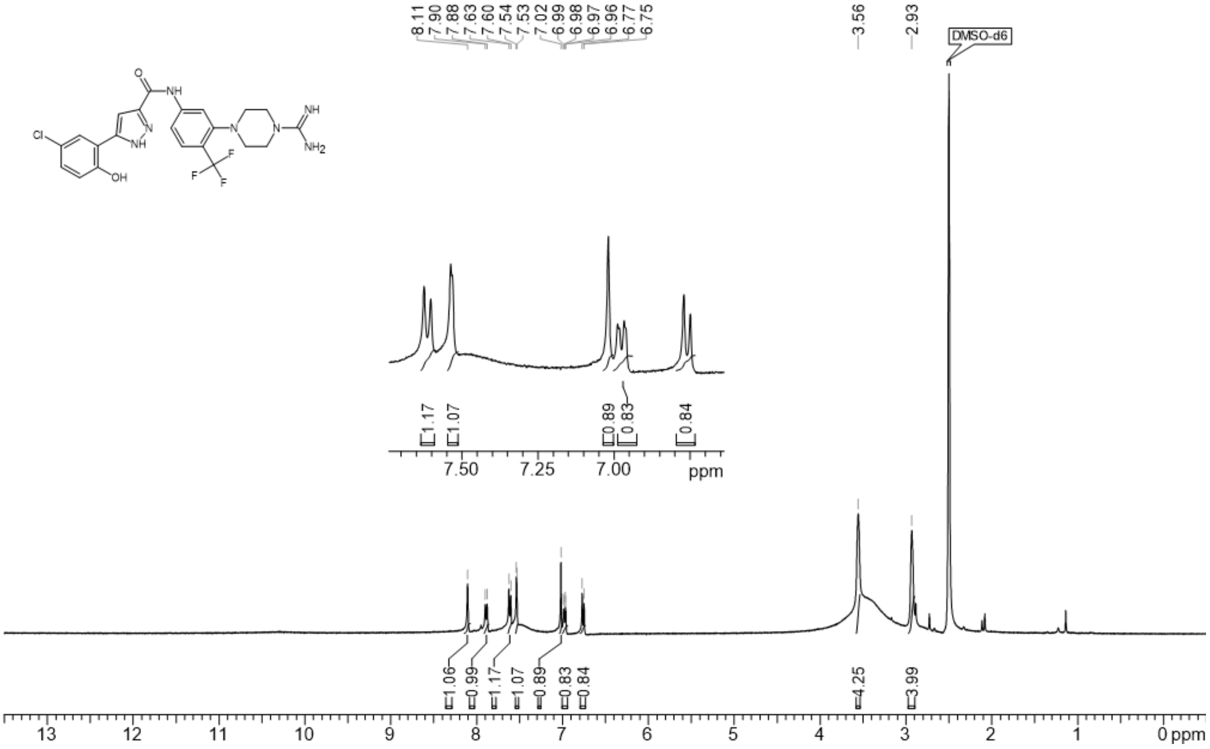

Figure S205. ^{1}H NMR spectrum of **13G.**

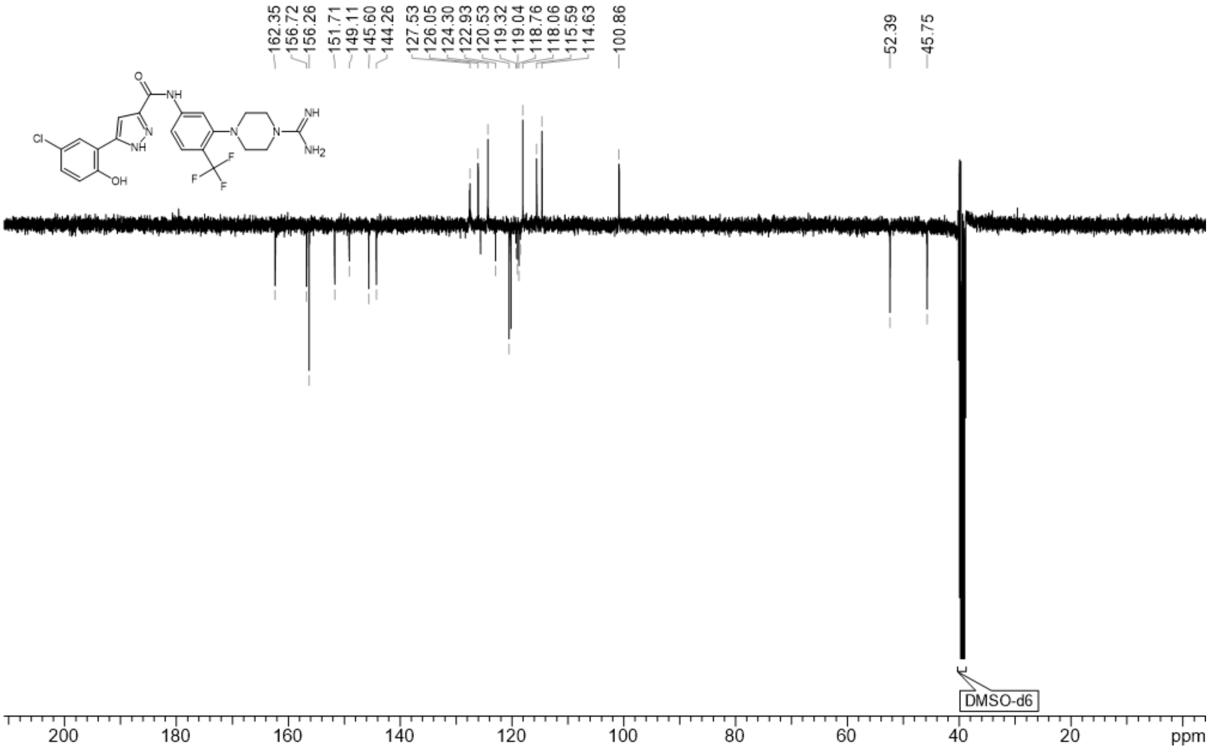

Figure S206. ^{13}C APT NMR spectrum of **13G.**

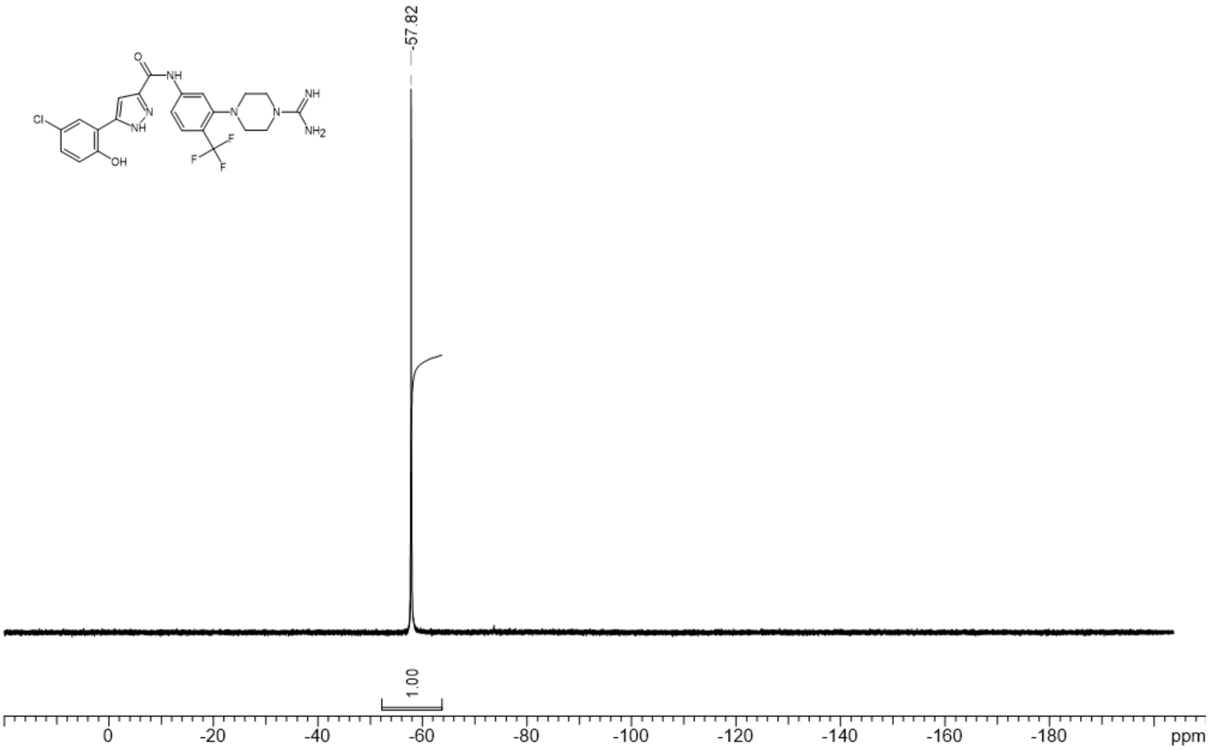

Figure S207. ^{19}F NMR spectrum of **13G.**

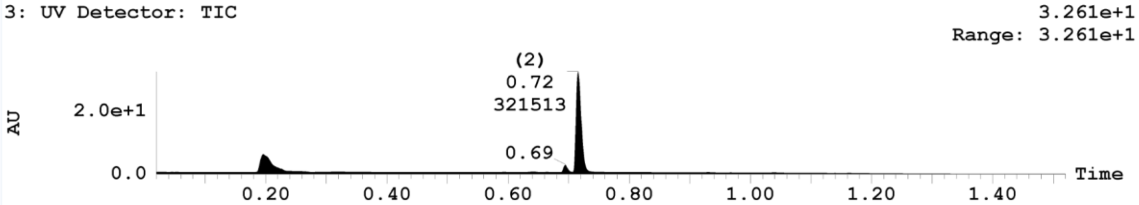

Peak Number	Time	Area %Total
1	0.69	5.12
2	0.72	94.88

Figure S208. LCMS purity analysis of **13G.**

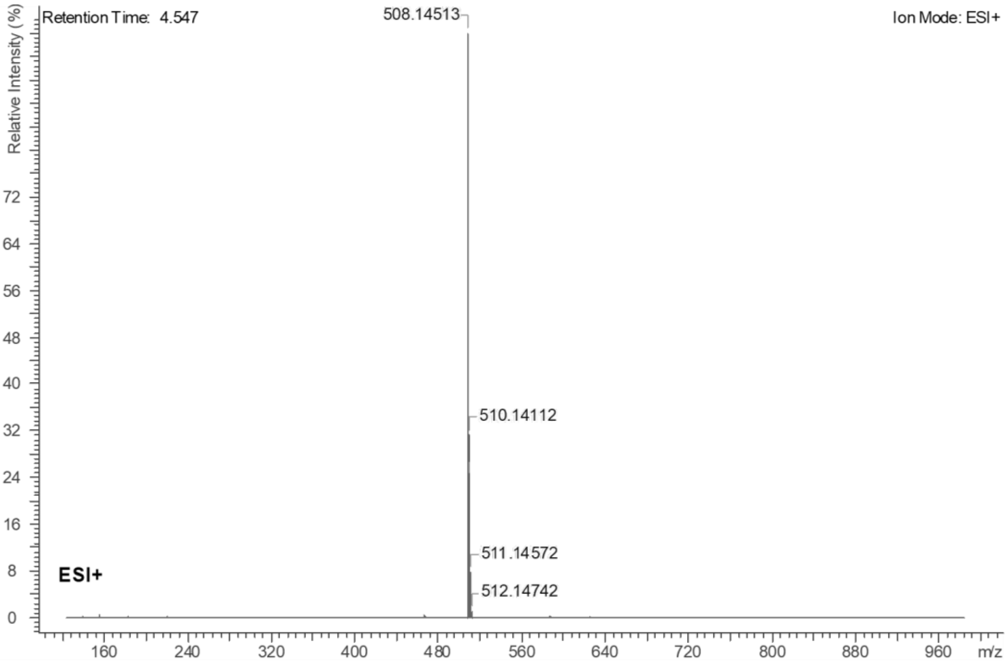

Figure S209. HRMS of **13G.**

Compounds **14A–G**

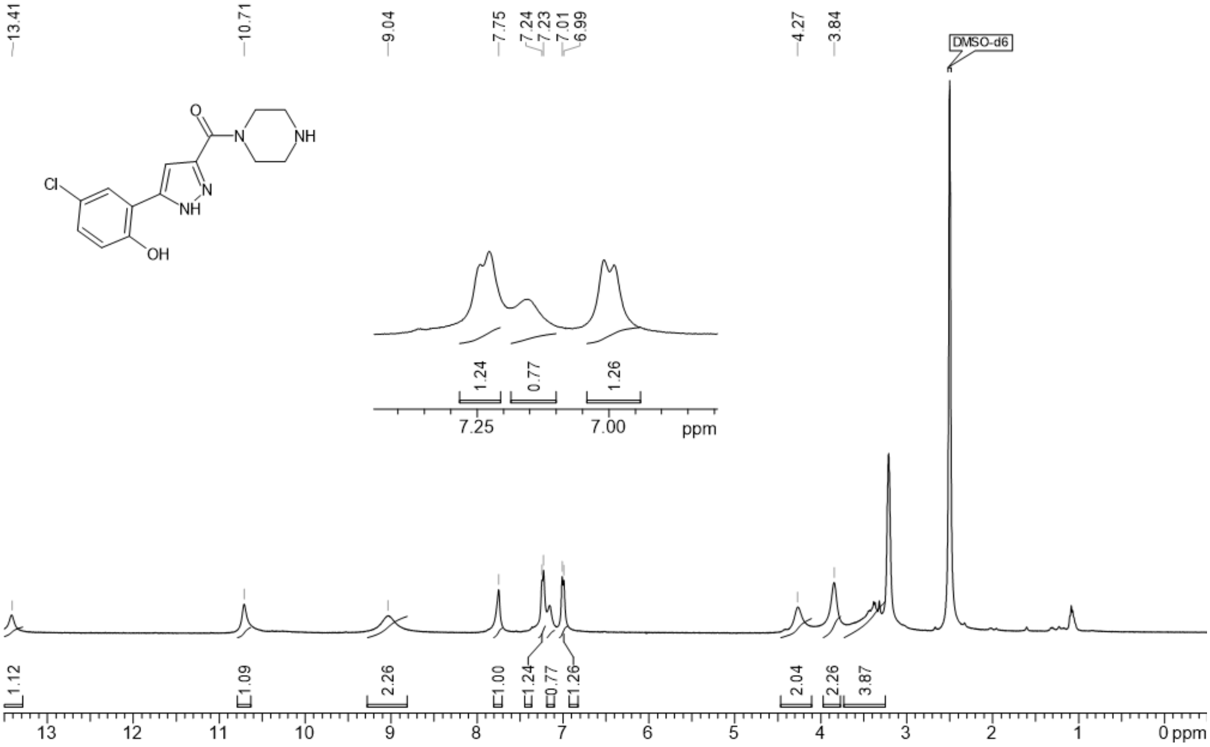

Figure S210. ^{1}H NMR spectrum of **14A.**

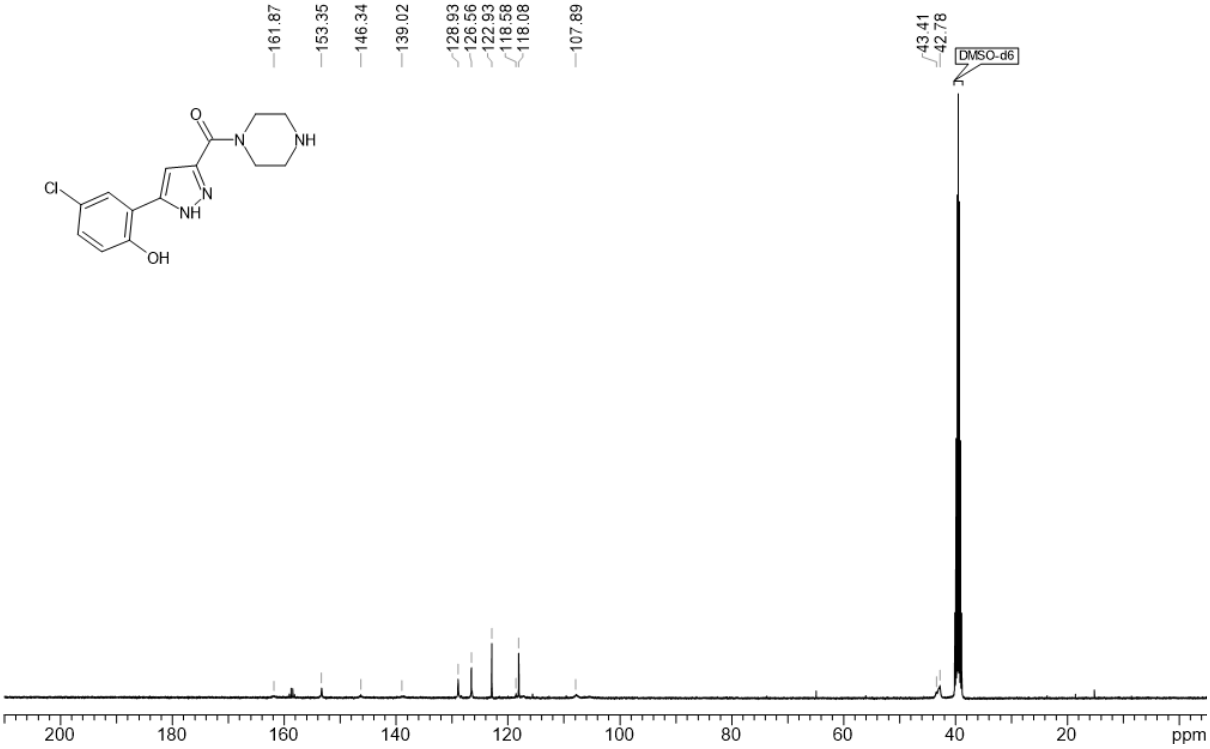

Figure S211. ^{13}C NMR spectrum of **14A.**

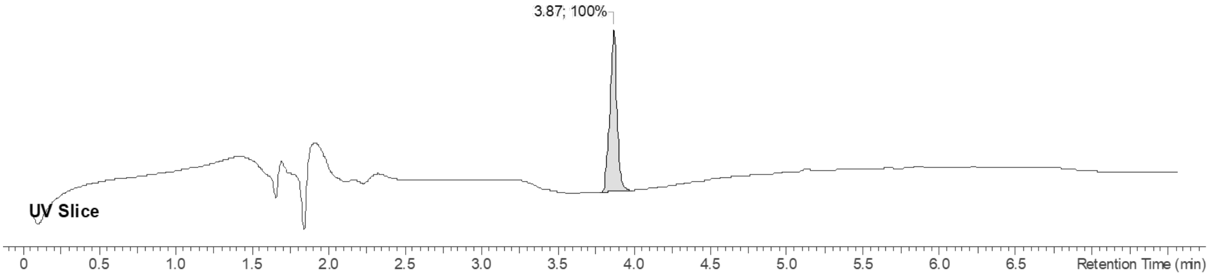

Figure S212. LCMS purity analysis of **14A**.

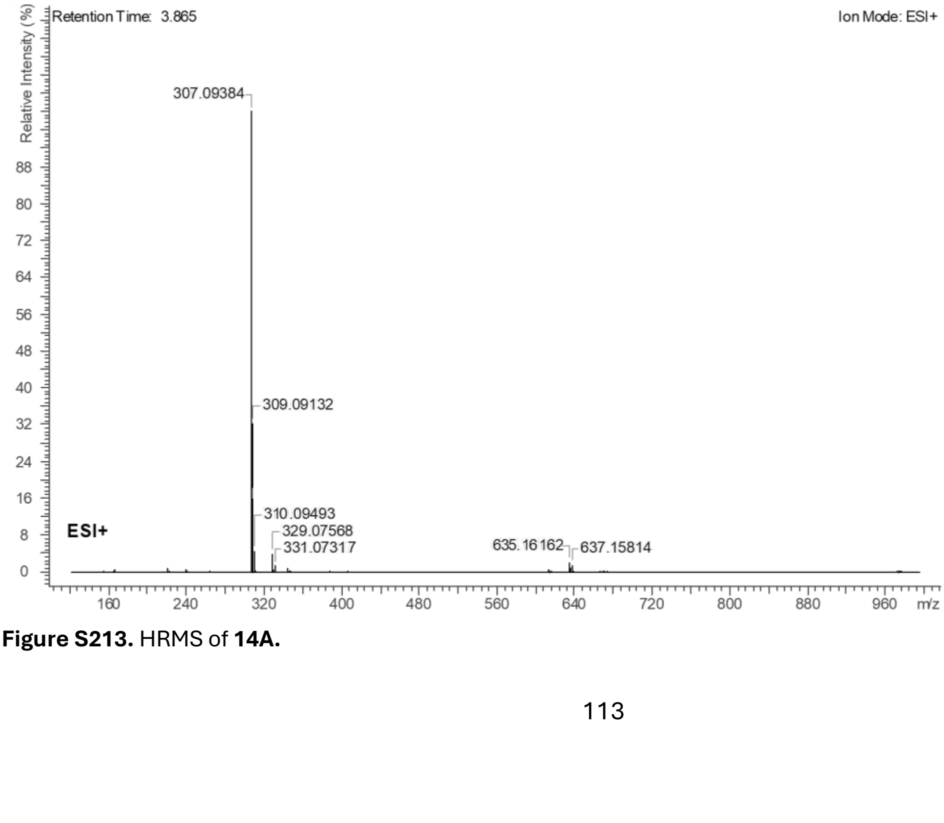

Figure S213. HRMS of **14A.**

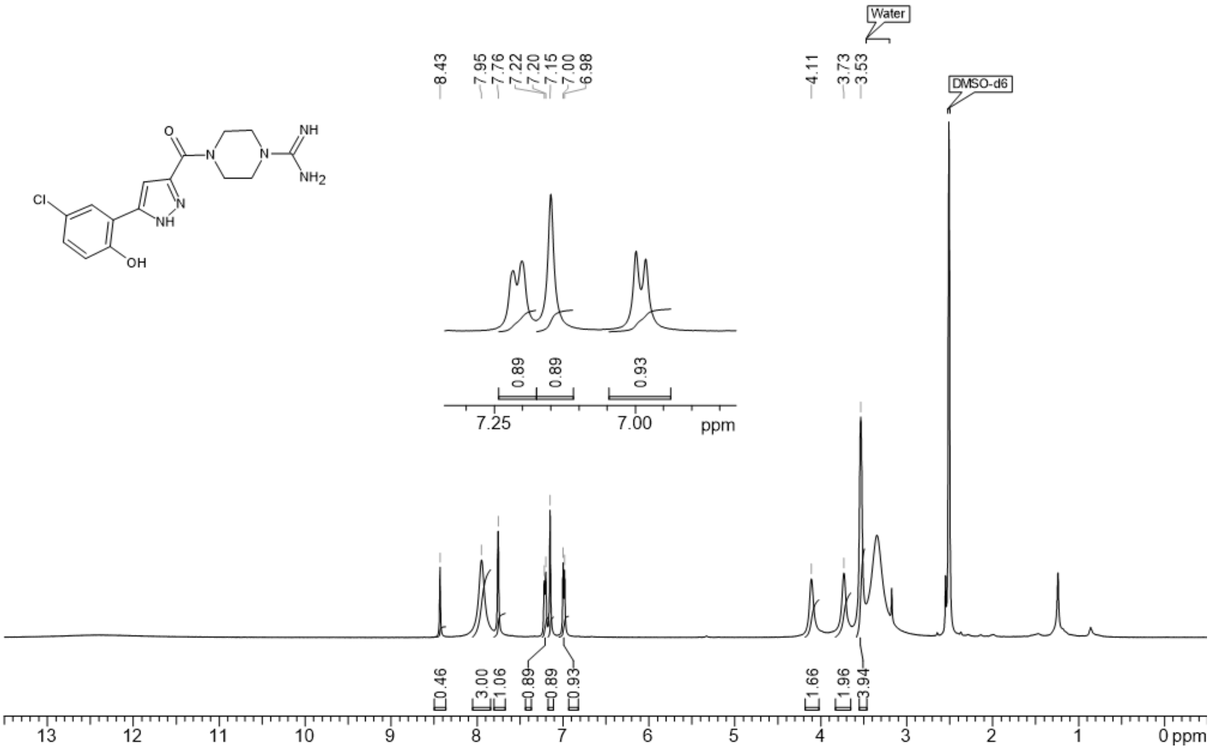

Figure S214. ^{1}H NMR spectrum of **14G.**

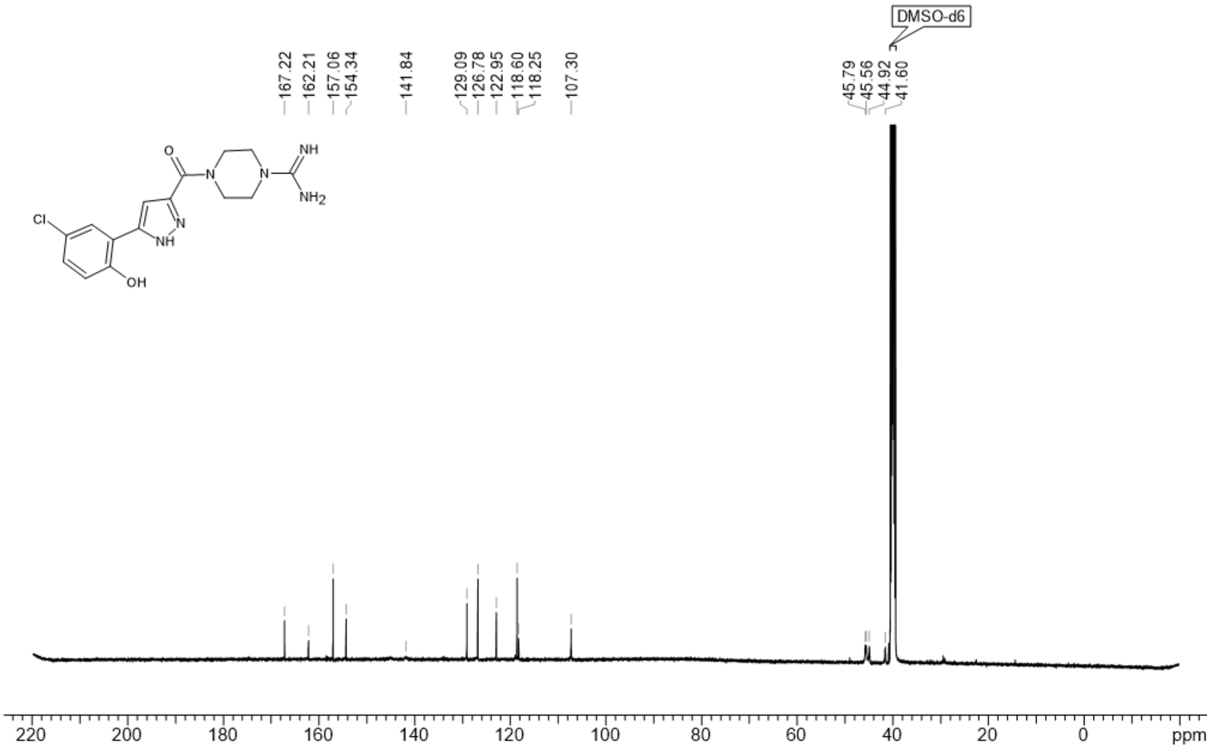

Figure S215. ^{13}C NMR spectrum of **14G.**

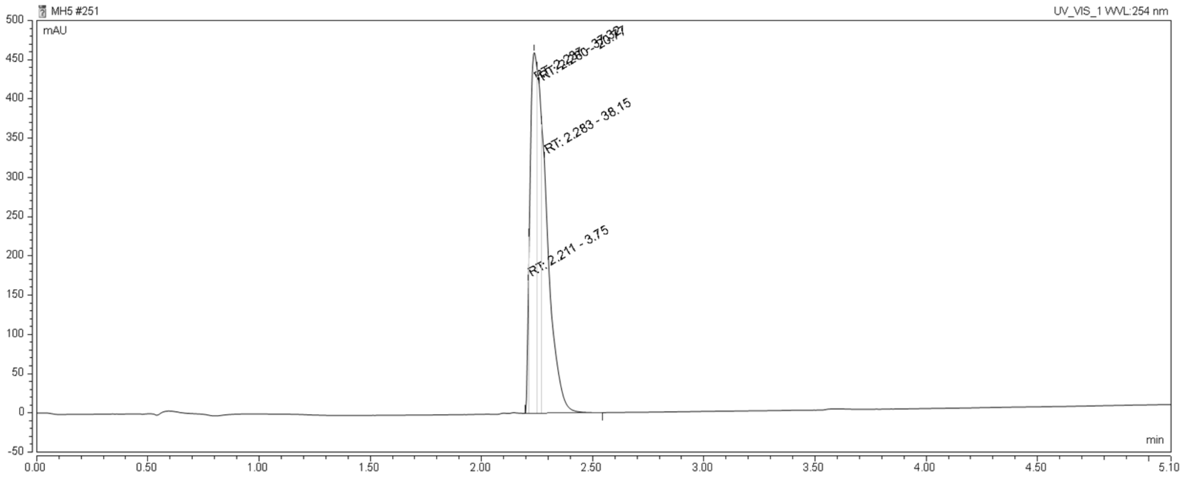

Figure S216. LCMS purity analysis of **14G.**

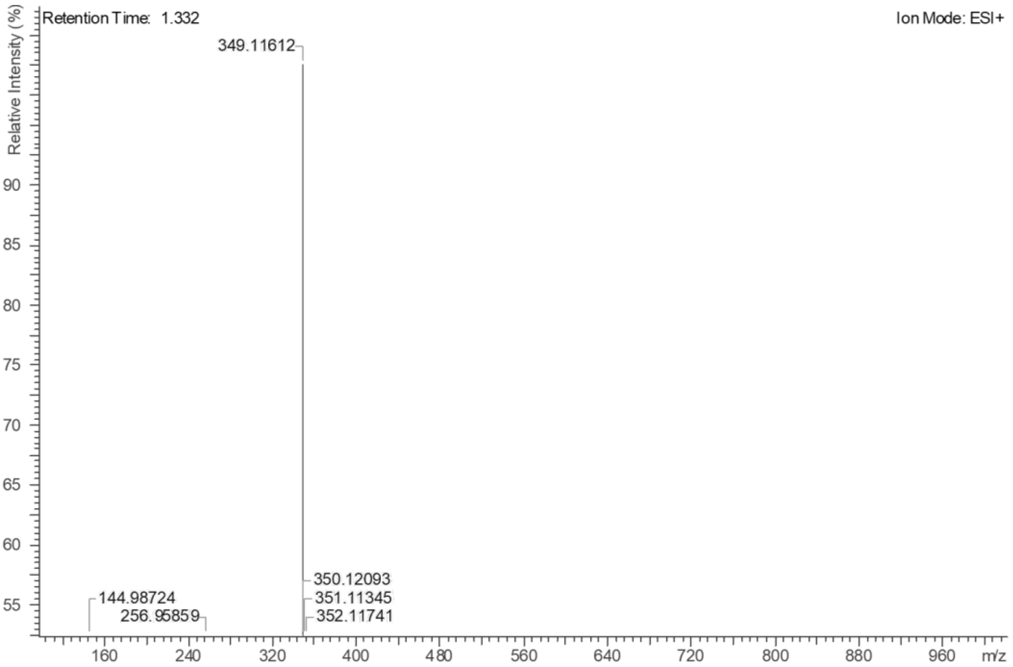

Figure S217. HRMS of **14G.**

Supplementary References

(1) Santos, C. M. M.; Silva, V. L. M.; Silva, A. M. S. Synthesis of Chromone-Related Pyrazole Compounds. *Molecules* **2017**, *22* (10). https://doi.org/10.3390/molecules22101665.

(2) Svenningsen, S. W.; Frederiksen, R. F.; Counil, C.; Ficker, M.; Leisner, J. J.; Christensen, J. B. Synthesis and Antimicrobial Properties of a Ciprofloxacin and PAMAM-Dendrimer Conjugate. *Molecules* **2020**, *25* (6). https://doi.org/10.3390/molecules25061389.

(3) Dardonville, C.; Caine, B. A.; Navarro De La Fuente, M.; Martín Herranz, G.; Corrales Mariblanca, B.; Popelier, P. L. A. Substituent Effects on the Basicity (p: K a) of Aryl Guanidines and 2-(Arylimino)Imidazolidines: Correlations of PH-Metric and UV-Metric Values with Predictions from Gas-Phase Ab Initio Bond Lengths. *New J Chem* **2017**, *41* (19), 11016–11028. https://doi.org/10.1039/c7nj02497e.

(4) Ueno, H.; Yokota, K.; Hoshi, J. I.; Yasue, K.; Hayashi, M.; Hase, Y.; Uchida, I.; Aisaka, K.; Katoh, S.; Cho, H. Synthesis and Structure-Activity Relationships of Novel Selective Factor Xa Inhibitors with a Tetrahydroisoquinoline Ring. *J Med Chem* **2005**, *48* (10), 3586–3604. https://doi.org/10.1021/jm058160e.

(5) Aoyagi, N.; Endo, T. Synthesis of Five- and Six-Membered Cyclic Guanidines by Guanylation with Isothiouronium Iodides and Amines under Mild Conditions. *Synth Commun* **2017**, *47* (5), 442–448. https://doi.org/10.1080/00397911.2016.1269927.

(6) Gaspar, A.; Reis, J.; Matos, M. J.; Uriarte, E.; Borges, F. In Search for New Chemical Entities as Adenosine Receptor Ligands: Development of Agents Based on Benzo-γ-Pyrone Skeleton. *Eur J Med Chem* **2012**, *54*, 914–918. https://doi.org/10.1016/j.ejmech.2012.05.033.

(7) Snyder, C.; Chollet, J.; Santo-Tomas, J.; Scheurer, C.; Wittlin, S. In Vitro and in Vivo Interaction of Synthetic Peroxide RBx11160 (OZ277) with Piperaquine in Plasmodium Models. *Exp Parasitol* **2007**, *115* (3), 296–300. https://doi.org/10.1016/j.exppara.2006.09.016.

(8) Huber, W.; Koella, J. C. A Comparison of Three Methods of Estimating EC50 in Studies of Drug Resistance of Malaria Parasites. *Acta Trop* **1993**, *55* (4), 257–261. https://doi.org/10.1016/0001-706X(93)90083-N.

(9) Jumde, R. P.; Guardigni, M.; Gierse, R. M.; Alhayek, A.; Zhu, D.; Hamid, Z.; Johannsen, S.; Elgaher, W. A. M.; Neusens, P. J.; Nehls, C.; Haupenthal, J.; Reiling, N.; Hirsch, A. K. H. Hit-Optimization Using Target-Directed Dynamic Combinatorial Chemistry: Development of Inhibitors of the Anti-Infective Target 1-Deoxy-D-Xylulose-5-Phosphate Synthase. *Chem Sci* **2021**, *12* (22), 7775–7785. https://doi.org/10.1039/D1SC00330E.

(10) Kolbe, K.; Möckl, L.; Sohst, V.; Brandenburg, J.; Engel, R.; Malm, S.; Bräuchle, C.; Holst, O.; Lindhorst, T. K.; Reiling, N. Azido Pentoses: A New Tool To Efficiently Label *Mycobacterium Tuberculosis* Clinical Isolates. *ChemBioChem* **2017**, *18* (13), 1172–1176. https://doi.org/10.1002/cbic.201600706.

(11) Zelmer, A.; Carroll, P.; Andreu, N.; Hagens, K.; Mahlo, J.; Redinger, N.; Robertson, B. D.; Wiles, S.; Ward, T. H.; Parish, T.; Ripoll, J.; Bancroft, G. J.; Schaible, U. E. A New in Vivo Model to Test Anti-Tuberculosis Drugs Using Fluorescence Imaging. *J. Antimicrob. Chemother.* **2012**, *67* (8), 1948–1960. https://doi.org/10.1093/jac/dks161.

(12) Reiling, N.; Homolka, S.; Walter, K.; Brandenburg, J.; Niwinski, L.; Ernst, M.; Herzmann, C.; Lange, C.; Diel, R.; Ehlers, S.; Niemann, S. Clade-Specific Virulence Patterns of Mycobacterium Tuberculosis Complex Strains in Human Primary Macrophages and Aerogenically Infected Mice. *mBio* **2013**, *4* (4). https://doi.org/10.1128/mBio.00250-13.
